# Comparative Analyses of Cytochrome P450s and Those Associated with Secondary Metabolism in *Bacillus* Species

**DOI:** 10.3390/ijms19113623

**Published:** 2018-11-16

**Authors:** Bongumusa Comfort Mthethwa, Wanping Chen, Mathula Lancelot Ngwenya, Abidemi Paul Kappo, Puleng Rosinah Syed, Rajshekhar Karpoormath, Jae-Hyuk Yu, David R. Nelson, Khajamohiddin Syed

**Affiliations:** 1 Department of Biochemistry and Microbiology, Faculty of Science and Agriculture, University of Zululand, KwaDlangezwa 3886, South Africa; 07bcomfort@gmail.com (B.C.M.); NgwenyaM@unizulu.ac.za (M.L.N.); KappoA@unizulu.ac.za (A.P.K.); 2 College of Food Science and Technology, Huazhong Agricultural University, Wuhan 430070, China; chenwanping@mail.hzau.edu.cn; 3 Department of Pharmaceutical Chemistry, College of Health Sciences, University of KwaZulu-Natal, Durban 4000, South Africa; prosinah@gmail.com (P.R.S.); Karpoormath@ukzn.ac.za (R.K.); 4 Department of Bacteriology, University of Wisconsin-Madison, 3155 MSB, 1550 Linden Drive, Madison, WI 53706, USA; jyu1@wisc.edu; 5 Department of Microbiology, Immunology and Biochemistry, University of Tennessee Health Science Center, Memphis, TN 38163, USA

**Keywords:** Antibiotics, *Bacillus*, biosynthetic gene clusters, comparative analysis, cytochrome P450 monooxygenase, *Mycobacterium*, P450 diversity percentage, P450 profiling, secondary metabolites

## Abstract

Cytochrome P450 monooxygenases (CYPs/P450s) are among the most catalytically-diverse enzymes, capable of performing enzymatic reactions with chemo-, regio-, and stereo-selectivity. Our understanding of P450s’ role in secondary metabolite biosynthesis is becoming broader. Among bacteria, *Bacillus* species are known to produce secondary metabolites, and recent studies have revealed the presence of secondary metabolite biosynthetic gene clusters (BGCs) in these species. However, a comprehensive comparative analysis of P450s and P450s involved in the synthesis of secondary metabolites in *Bacillus* species has not been reported. This study intends to address these two research gaps. *In silico* analysis of P450s in 128 *Bacillus* species revealed the presence of 507 P450s that can be grouped into 13 P450 families and 28 subfamilies. No P450 family was found to be conserved in *Bacillus* species. *Bacillus* species were found to have lower numbers of P450s, P450 families and subfamilies, and a lower P450 diversity percentage compared to mycobacterial species. This study revealed that a large number of P450s (112 P450s) are part of different secondary metabolite BGCs, and also identified an association between a specific P450 family and secondary metabolite BGCs in *Bacillus* species. This study opened new vistas for further characterization of secondary metabolite BGCs, especially P450s in *Bacillus* species.

## 1. Introduction

Cytochrome P450 monooxygenases, also known as CYPs/P450s, are undoubtedly among the most catalytically-diverse enzymes, performing enzymatic reactions with chemo-, regio- and stereo-selectivity [1,2,3,4,5,6]. The catalytic diversity combined with chemo-, regio- and stereo-specific oxidation of substrates exerted by P450s are used in diverse biotechnological applications ranging from drug discovery to bioethanol production and synthesis of different secondary metabolites [7,8,9,10,11,12]. P450s are heme-thiolate proteins ubiquitously found in species belonging to different biological kingdoms, including non-living entities such as viruses [13,14]. In bacteria, P450s have been found to play a key role in enzymatic reactions, leading to the biosynthesis of physiological compounds or the biodegradation of xenobiotics [9,11,15,16].

P450s’ role in the synthesis of a diverse array of secondary metabolites has been thoroughly reviewed [8,12]. Secondary metabolites are natural products that are widely-used in human and veterinary medicine, agriculture, and manufacturing, and are known to mediate a variety of microbe-host and microbe-microbe interactions [17]. P450s were found to play a key role in the synthesis of different secondary metabolites, including terpenes, alkaloids, shikimates, polyketides, and peptides [12]. The coding sequences (genes) of enzymes involved in the synthesis of different secondary metabolites, including P450s, were found to be part of gene clusters named biosynthetic gene clusters (BGCs) [17]. Bacterial species have been found to have more than 1000 different types of BGCs involved in the synthesis of known and unknown secondary metabolites [17].

Among bacteria, species belonging to the genus *Bacillus* are ubiquitously present in the biosphere, and are well known for their distinct features with one common characteristic, i.e., making dormant endospores during unfavorable growth conditions [18,19]. Applications of *Bacillus* species across different spectra have been well explored in the industrial, agricultural, and ecological fields, and by academics, against a backdrop of being a well-known human pathogen [18,19]. Comprehensive *in silico* studies detailing *Bacillus* species’ ability to produce different secondary metabolites and different types of BGCs have frequently been reported [20,21]. Analysis of 1566 *Bacillus* species’ genomes revealed the presence of 20,000 BGCs, most of which were found to produce known secondary metabolites that play a key role in the physiology and development of *Bacillus* species [21]. The study by Grubbs et al. [21] also reported that secondary metabolite alkylpyrones play a key role in inhibiting spore development in *Bacillus* species.

Despite comprehensive analysis of *Bacillus* species’ secondary metabolite BGCs, P450s that are part of different BGCs have not been reported. Analysis of P450s in *Bacillus* species date back to 2009, when the authors performed a comparative analysis of P450s in 29 *Bacillus* species, and identified a few P450s belonging to a limited number of P450 families such as CYP102, CYP106, CYP107, CYP109, CYP134, CYP152, and CYP197 [22]. Among the P450 families identified in *Bacillus* species, the CYP102 P450 family has a special place in P450 research, being one of the most extensively-studied bacterial P450s, for its structural, functional, and biotechnological potential. Even the *Bacillus* species *B. megaterium* has become very famous owing to the identification of CYP102 P450 from this organism [23,24]. *In silico* comparative analysis of P450s in bacterial species is gaining momentum. Recently, a comprehensive comparative analysis of P450s in 60 mycobacterial species has been reported; the authors identified that mycobacteria possess a large number of P450s, and that different mycobacterial categories have characteristic P450 families that can be used as biomarkers to identify different mycobacterial species [25].

The current trend of whole-genome sequencing of organisms resulted in the genome sequencing of a large number of *Bacillus* species genomes. Quite a large number of *Bacillus* species genome sequences are available for public use at Kyoto Encyclopedia of Genes and Genomes—GenomeNet (KEGG) [26]. This gives us the opportunity to perform comprehensive a comparative analysis of P450s in *Bacillus* species as per international P450 nomenclature committee rules [27,28,29], and to identify P450s involved in the synthesis of different secondary metabolites. Here, we report genome data mining, annotation, phylogenetic and comparative analysis of P450s in 128 *Bacillus* species, including identification of P450s involved in the synthesis of different secondary metabolites. This study also reports comparative analysis of P450s between the genera *Bacillus* and *Mycobacterium*. Last but not least, a previous study reporting BGCs in *Bacillus* species did not clearly indicate BGCs on genomic DNA (gDNA) and plasmid DNA [21]; thus, in this study, gDNA and plasmid DNAs were individually subjected to BGC analysis.

## 2. Results and Discussion

### 2.1. Bacillus Species Have the Lowest Number of P450s

**Figure 1:**
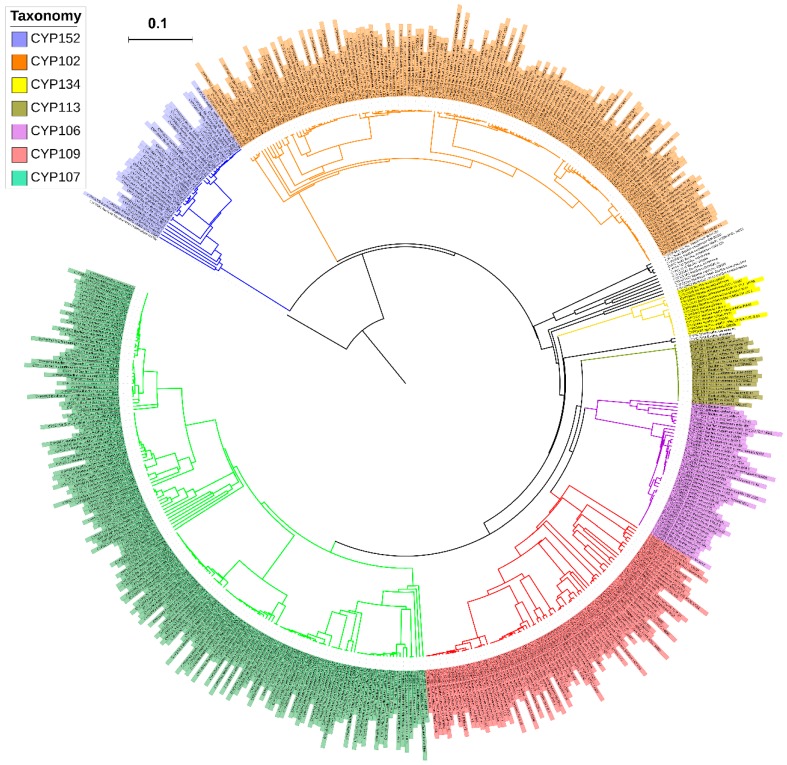
Phylogenetic analysis of *Bacillus* species P450s. Dominant P450 families were indicated in different colors. CYP51B1 from *Mycobacterium tuberculosis* H37Rv is used as an outgroup. A high-resolution phylogenetic tree is provided in the supplementary Figure S1.

**Figure 2:**
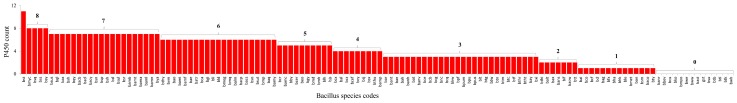
Comparative analysis of P450s in *Bacillus* species. The numbers next to bars indicate the number of P450s in *Bacillus* species. *Bacillus* species’ names with respect to their codes can be found in Supplementary Dataset 1.

Genome data-mining and annotation of P450s in 128 *Bacillus* species revealed the presence of the lowest number of P450s in their genomes (Figure 1 and Figure 2). In total, 507 P450s were found in 114 *Bacillus* species, where 14 species did not have P450s in their genomes (Figure 1 and Figure 2). On average, four P450s were found in *Bacillus* species, with the highest number, 11, found in *Bacillus* subtilis subsp. *spizizenii* TU-B-10. The number of P450s found in *Bacillus* species is very low compared to mycobacterial species (60 species); the latter species have, on average, 35 P450s in their genomes [25]. The P450 count in *Bacillus* species, apart from *B. subtilis* subsp. *spizizenii* TU-B-10, which has 16, is as follows: 9 in 2 species, 8 in 4 species, 7 in 20 species, 6 in 21 species, 5 in 10 species, 4 in 9 species, 3 in 28 species, 2 in 7 species, and a single in 14 species (Figure 1 and Figure 2). This indicates that most *Bacillus* species (28 species) have three P450s in their genomes. P450s identified in each *Bacillus* species and respective P450 sequences were presented in Supplementary datasets 1 and 2.

### 2.2. Bacillus Species Have the Lowest Number of P450 Families and Subfamilies’

**Figure 3:**
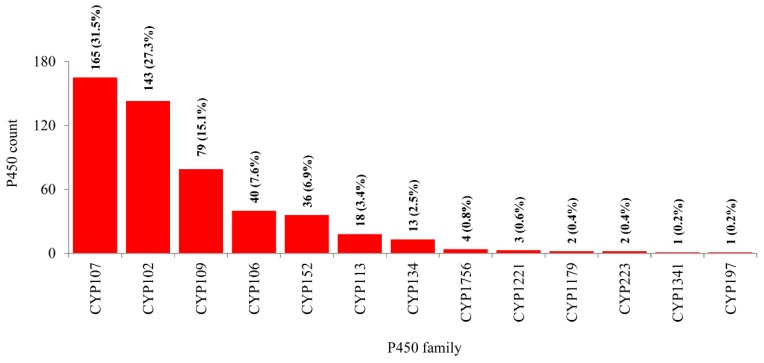
Comparative analysis of P450 families in *Bacillus* species. The numbers next to the family bar indicate the total number of P450s and percentage contribution (parenthesis) by a respective family to the total number of P450s. The data on the number of P450s in each P450 family in *Bacillus* species is presented in Table S1.

**Figure 4:**
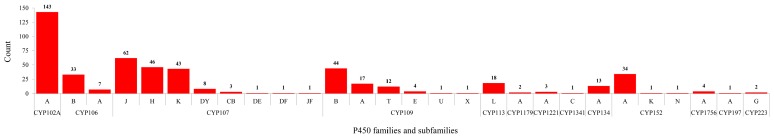
Comparative analysis of P450 subfamilies in *Bacillus* species. The numbers next to bars indicate the total number of members in a particular subfamily. Data on the number of P450s in each P450 subfamily in *Bacillus* species is presented in Table S1.

As per the International P450 Nomenclature Committee rules [27,28,29], all 507 P450s found in 114 *Bacillus* species can be grouped into 13 P450 families and 28 subfamilies (Figure 3 and Figure 4). Phylogenetic analysis of *Bacillus* P450s revealed P450s belonging to the same family grouped together, suggesting that the annotation of P450s in this study is accurate (Figure 1). The number of P450 families and subfamilies found in *Bacillus* species is lower compared to mycobacterial species (60 species), which have 77 P450 families and 132 subfamilies [25]. Because of the presence of the lowest number of P450 families, the P450 diversity percentage in *Bacillus* species was found to be lowest (3.9%) compared to mycobacterial species (72%) [25]. Among 13 P450 families, the CYP107 P450 family has the highest number of P450s (165 P450s) contributing 31.5% of 507 P450s (Figure 3), followed by CYP102 (143 P450s), CYP109 (79 P450s), CYP106 (40 P450s), CYP152 (36 P450s), CYP113 (18 P450s), CYP134 (13 P450s), CYP1756 (4 P450s), CYP1221 (3 P450s), CYP1179 and CYP223 (2 P450s), CYP1341 and CYP197 (single P450s) (Figure 3).

**Figure 5:**
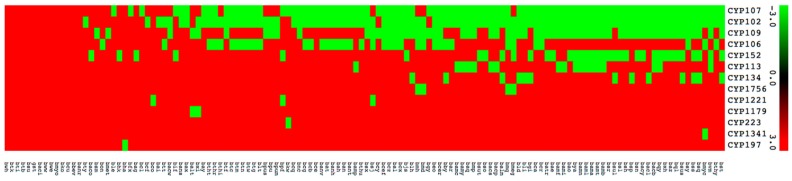
Heatmap of presence or absence of cytochrome P450 families in 128 species of *Bacillus*. The data have been represented as −3 for family presence (green) and 3 for family absence (red). A hundred and twenty-eight *Bacillus* species form the horizontal axis and CYP family numbers form the vertical axis. The respective data used in the generation of Figure 5 is presented in Supplementary Dataset 3.

P450 subfamily analysis revealed that most P450 families have a single subfamily (Figure 4). Among P450 families, CYP107 has the highest number of P450 subfamilies (eight subfamilies) followed by CYP109 (six subfamilies), CYP152 (three subfamilies), and CYP106 (two subfamilies) (Figure 4). The remaining nine P450 families, CYP102, CYP113, CYP1179, CYP1221, CYP1341, CYP134, CYP1756, CYP197, and CYP223, all have a single subfamily (Figure 4). It is interesting to note that the CYP102 P450 family, despite having the second largest number of P450s, has a single subfamily “A”. Analysis of P450 subfamily profiles revealed that specific subfamilies are dominant in a particular family (Figure 4). Subfamily “J” is dominant in the CYP107 family, subfamily “B” is dominant in the CYP109 family and subfamily “A” is dominant in the CYP152 family (Figure 4). Analysis of P450 family profiles revealed that no P450 family is conserved across *Bacillus* species (Figure 5). Most *Bacillus* species have CYP102, CYP107, CYP109, CYP106, and CYP152 P450 families (Figure 5). CYP197, CYP223, and CYP1341 are present in a single *Bacillus* species (Supplementary Dataset 1).

### 2.3. Bacillus Species Have the Lowest Number of Secondary Metabolite BGCs

**Figure 6:**
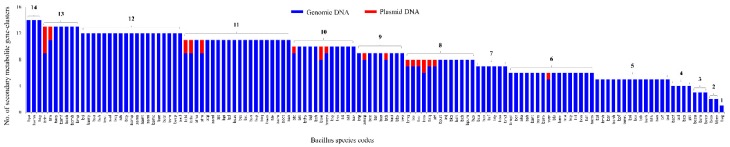
Comparative analysis of secondary metabolite BGCs in 128 *Bacillus* species (gDNA and plasmid DNA). Numbers next to bars indicate the number of secondary metabolite BGCs. Detailed analysis of secondary metabolite BGCs in each species is presented in Table S3.

In order to identify P450s involved in the biosynthesis of secondary metabolites, the gDNA and plasmid DNA of each *Bacillus* species (Table S2) has been subjected to secondary metabolite BGCs analysis using anti-SMASH [30]. In total, 203 plasmids were identified in 60 *Bacillus* species (Table S2). Analysis of 128 *Bacillus* species genomes revealed the presence of 1098 and 26 secondary metabolite BGCs on gDNA and plasmid DNA, respectively (Figure 6 and Table S3).

The number of secondary metabolite BGCs varied from a maximum of 14 to one in *Bacillus* species gDNA. Interestingly, among 203 plasmid DNAs from 60 *Bacillus* species (Table S3), only 21 plasmid DNAs from 18 *Bacillus* species were found to have secondary metabolite BGCs (Figure 6 and Table S3). The number of secondary metabolites BGCs on plasmid DNAs varied from a maximum of four to one (Figure 6 and Table S3).

**Figure 7:**
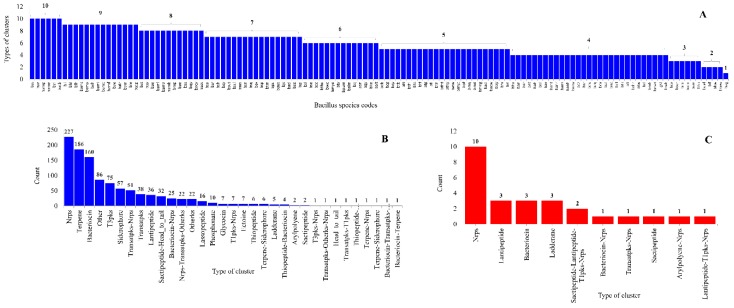
Comparative analysis of types of secondary metabolite BGCs in *Bacillus* species. (**A**) The number of types of secondary metabolite BGCs in *Bacillus* species. (**B**,**C**). Comparative analysis of types of secondary metabolite BGCs on gDNA and plasmid DNAs. Standard abbreviations representing secondary metabolite BGCs as indicated in anti-SMASH [30] were used in the figure.

Analysis of types of secondary metabolite BGCs revealed the presence of 33 and 10 types of BGCs on gDNA and plasmid DNAs in *Bacillus* species (Figure 7 and Supplementary Dataset 4). The types of BGCs in individual *Bacillus* species varied from a maximum of 10 types to one (Figure 7). Among types of BGCs, Nonribosomal peptides secondary metabolite (Nrps) BGCs were dominant in *Bacillus* species, both on gDNA and plasmid DNAs (Figure 7).

Analysis of types of BGCs on gDNA and plasmid DNAs revealed the presence of seven types of common BGCs between gDNA and plasmid DNAs (Figure 7). This suggests that these plasmids might be involved in horizontal gene transfer of different BGCs among *Bacillus* species. It is important to note that horizontal gene transfer of BGCs is a common phenomenon among *Bacillus* species [21]. Interestingly, three distinct types of secondary metabolite gene clusters, namely Sactipeptide-Lantipeptide-T1pks-Nrps, Arylpolyene-Nrps, and Lantipeptide-T1pks-Nrps, were only identified on plasmid DNAs (Figure 7).

### 2.4. Large Number of P450s Found to Be Part of Secondary Metabolites BGCs in Bacillus Species

**Figure 8:**
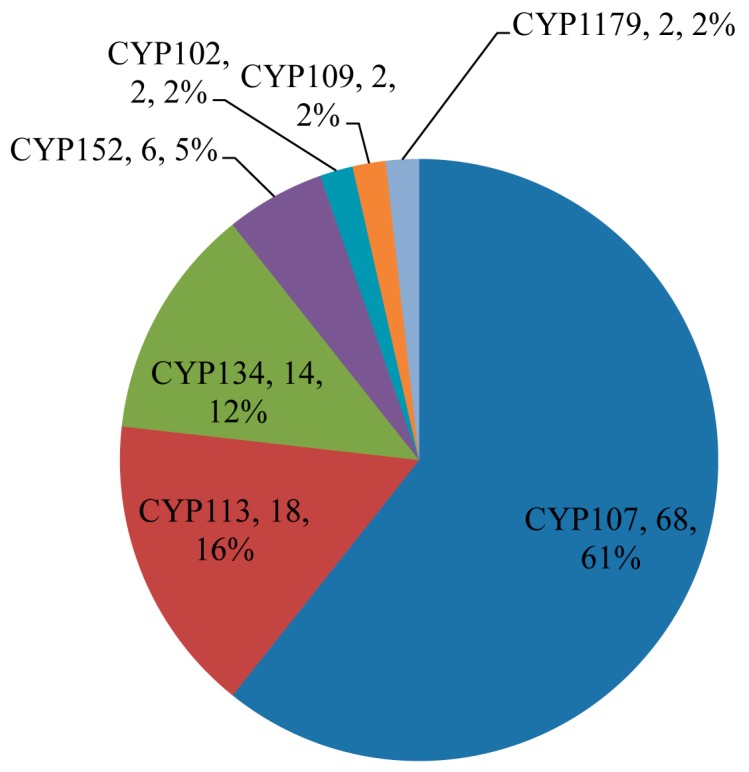
Comparative analysis of P450 families involved in secondary metabolite biosynthesis. The P450 family name, number of P450s and their percentage of the total number of 112 P450s are presented in the figure.

Among 507 P450s identified in 128 *Bacillus* species, 112 P450s (22%) from 50 *Bacillus* species were found to be part of secondary metabolite BGCs (Table 1). Among 13 P450 families, only seven families, namely CYP107, CYP113, CYP134, CYP152, CYP102, CYP109, and CYP1179, were found to be part of different secondary metabolite BGCs (Figure 8). P450 subfamily level analysis revealed that P450s belonging to the subfamilies H and K in the CYP107 family were part of secondary metabolite BGCs, despite subfamily J being dominant in that family (Figure 4). In the CYP152 family, P450s belonging to subfamily A were found to be part of the secondary metabolite BGCs. Analysis of P450s involving secondary metabolite biosynthesis revealed that P450s belonging to the CYP107 family are dominant by 61% (68 P450s) of all P450s (112 P450s) involved in secondary metabolite BGCs, followed by CYP113, CYP152, CYP102, CYP109, and CYP1179 (Figure 8). It is interesting to note that these P450 families are highly-populated in *Bacillus* species (Figure 3 and Figure 5). This further supports the previous hypothesis that species populate specific P450s if they are useful in their adaptation to certain ecological niches or useful in their physiology [31,32,33,34,35]. Considering the large number of P450s, their widespread nature, and part in secondary metabolite BGCs, it can be hypothesized that the P450s belonging to the CYP107, CYP102, CYP109, CYP152, and CYP113 families play a key role in *Bacillus* species’ physiology, including synthesis of different secondary metabolites. Despite secondary metabolite BGCs being found on plasmid DNA, no P450 was found to be part of these clusters. Analysis of association between P450 families and secondary metabolite BGCs revealed that CYP107 family P450s were mostly associated with BGCs Nrps-Transatpks-Otherks and Transatpks-Nrps; CYP113 family P450s are associated with Transatpks BGC, and CYP134 family P450s are associated with other, a putative gene cluster (Table 1).

### 2.5. Bacillus P450s Indeed Involved in the Synthesis of Secondary Metabolites

Based on *in silico* analysis (in this study), seven P450 families, namely CYP107, CYP113, CYP134, CYP152, CYP102, CYP109, and CYP1179, were identified as part of secondary metabolite BGCs in *Bacillus* species (Figure 8). Functional data available for some P450s confirms that the predicted P450s, in this study, are indeed involved in biosynthesis of different secondary metabolites, and some of the P450 families, such as CYP105, CYP107, and CYP109, have been found to display highly-diverse functions [9,12,36]. CYP102A1 from *B. megaterium* [24,37,38] and CYP152A1 from *B. subtilis* [39,40] were found to be fatty acid hydroxylases. P450s belonging to the CYP106, CYP107, CYP109, and CYP134 families were found to hydroxylate different steroids, albeit with different substrate specificities [22]. CYP134A1 is involved in the synthesis of pulcherriminic acid, a natural product [41], and CYP107H1 (P450 biol) is involved in the synthesis of polyketides [42]. Based on functionally characterized homolog P450s from other organisms, CYP105, CYP107, and CYP109 family P450s have been found to be associated with the degradation and biotransformation of a diverse array of xenobiotics and secondary metabolites [36,43,44]. CYP113 P450s are involved in the biosynthesis of secondary metabolites such as erythromycin [45,46] and tylosin [47,48]. Despite CYP102 and CYP152 P450s being found in secondary metabolite BGCs (in this study), their role in secondary metabolites biosynthesis has not been yet elucidated.

## 3. Materials and Methods

### 3.1. Species and Database

In total, 128 *Bacillus* species genomes available for public use at KEGG (https://www.genome.jp/kegg-bin/show_organism?category=Bacillus) were used in this study (Table S4). *Bacillus* species used in this study, along with their names, species codes, and individual genome database links, were presented in Table S4.

### 3.2. Genome Data Mining and Annotation of P450s

P450 mining in *Bacillus* species was carried out following the methods described elsewhere [25]. Briefly, the whole proteome of *Bacillus* species was downloaded from the databases listed in Table S4, and subjected to the NCBI Batch Web CD-Search Tool (http://www.ncbi.nlm.nih.gov/Structure/bwrpsb/bwrpsb.cgi). Proteins that belong to a P450 superfamily were selected and based on the International P450 Nomenclature Committee rule; proteins with >40% identity and >55% identity were grouped under the same family and subfamily, respectively [27,28,29]. Proteins with less than 40% identity were assigned to a new P450 family.

### 3.3. Phylogenetic Analysis of P450s

The phylogenetic tree of *Bacillus* species P450s was built as described elsewhere [25], with slight modifications. Briefly, the *Bacillus* P450s protein sequences along with the outgroup *M. tuberculosis* CYP51B1 (Rv0764c) protein were aligned by MAFFT v6.864 [49], embedded on the Trex web server [50]. Then, the alignments were automatically subjected to tree inferring and optimization by the Trex web server. Finally, the best-inferred trees were visualized, colored, and generated by iTOL (http://itol.embl.de/about.cgi) [51].

### 3.4. P450 Diversity Percentage Analysis

P450 diversity percentage analysis was carried out as described elsewhere [25,34]. Briefly, the P450 diversity percentage in *Bacillus* species was measured as a percentage contribution of the number of P450 families in the total number of P450s.

### 3.5. Generation of P450 Profile Heat-Maps

The presence or absence of P450s in *Bacillus* species was shown with heat-maps generated using P450 family data. The data was represented as −3 for family presence (green) and 3 for family absence (red). A tab-delimited file was imported into Mev (Multi-experiment viewer) [52]. Hierarchical clustering using a Euclidean distance metric was used to cluster the data. A hundred and twenty-eight *Bacillus* species formed the horizontal axis (see Supplementary dataset 3 for codes) and CYP family numbers formed the vertical axis.

### 3.6. Secondary Metabolite BGCs Analysis

Individual *Bacillus* species genome ID and plasmids IDs from the various species databases (Table S2) were submitted to anti-SMASH [30] for identification of secondary metabolite BGCs. Results were downloaded both in the form of gene cluster sequences and Excel spreadsheets representing species-wise cluster information, and finally, P450s that are part of a specific gene cluster were identified. Standard gene cluster abbreviation terminology available at anti-SMASH database [30] was maintained in this study.

### 3.7. Comparative Analysis of P450s

Mycobacterial P450s were retrieved from a published article [25] and used for comparative analysis with *Bacillus* species P450s. P450 families and subfamilies and the P450 diversity percentage were compared between the genera *Mycobacterium* and *Bacillus*.

## 4. Conclusions

Comparative analysis of P450s in bacterial species is gaining momentum and the availability of a large number of bacterial genome sequences is fueling this process. This study is an attempt to perform a comprehensive comparative analysis of P450s and to identify the P450s involved in secondary metabolite synthesis in *Bacillus* species. Future work involves understanding the role of different *Bacillus* P450s, identified in this study, in the synthesis of various secondary metabolites.

## Figures and Tables

**Table 1:** Identification of P450s that are involved in secondary metabolite BGCs in *Bacillus* species. BGCs in each species and P450 identified as part of a particular cluster are presented in Supplementary Dataset 5.

Species Name	Cluster Number	Type of BGCs	P450 Name
*Bacillus subtilis* subsp. *subtilis* 168	4	Nrps-Transatpks-Otherks	CYP134A1
	10	Other	CYP134A1
*Bacillus subtilis* subsp. *subtilis* RO-NN-1	3	Nrps-Transatpks-Otherks	CYP107K1
*Bacillus subtilis* subsp. *subtilis* BSP1	8	Transatpks-Otherks-Nrps	CYP107K1
*Bacillus subtilis* subsp. *subtilis* 6051-HGW	4	Nrps-Transatpks-Otherks	CYP107K1
	10	Other	CYP107K1
*Bacillus subtilis* subsp. *subtilis* BAB-1	4	Nrps-Transatpks-Otherks	CYP107K1
*Bacillus subtilis* subsp. *subtilis* AG1839	4	Nrps-Transatpks-Otherks	CYP107K1
	10	Other	CYP107K1
*Bacillus subtilis* subsp. *subtilis* JH642	4	Nrps-Transatpks-Otherks	CYP107K1
	10	Other	CYP134A1
*Bacillus subtilis* subsp. *subtilis* OH 131.1	1	Lantipeptide	CYP152A1
	4	Nrps-Transatpks-Otherks	CYP107K1
	9	Other	CYP134A1
*Bacillus subtilis* subsp. *spizizenii* W23	1	Phosphonate	CYP152A1
	4	Nrps-Transatpks-Otherks	CYP107K1
	10	Other	CYP134A1
*Bacillus subtilis* subsp. *spizizenii* TU-B-10	3	Nrps-Transatpks-Otherks	CYP107K1
	9	Other	CYP134A1
*Bacillus subtilis* BSn5	4	Other	CYP102A48
	8	Lantipeptide	CYP152A1
	11	Nrps-Transatpks-Otherks	CYP107K1
*Bacillus subtilis* QB928	4	Nrps-Transatpks-Otherks	CYP107K1
	10	Other	CYP134A1
*Bacillus subtilis* XF-1	4	Nrps-Transatpks-Otherks	CYP107K1
*Bacillus subtilis* PY79	4	Nrps-Transatpks-Otherks	CYP107K1
	9	Other	CYP134A1
*Bacillus licheniformis* ATCC 14580	7	Other	CYP134A5
*Bacillus licheniformis* DSM 13 = ATCC 14580	7	Other	CYP134A5
*Bacillus paralicheniformis*	10	Other	CYP134A5
*Bacillus velezensis* FZB42	5	Transatpks-Nrps	CYP107K3
	6	Transatpks-Nrps	CYP107H4
	9	Transatpks	CYP113L1
*Bacillus velezensis* CAU B946	5	Transatpks-Nrps	CYP107K3
	6	Transatpks-Nrps	CYP107H4
	9	Transatpks	CYP113L1
*Bacillus velezensis* YAU B9601-Y2	5	Transatpks	CYP107K3
	6	Transatpks-Nrps	CYP107K3
	7	Transatpks-Nrps	CYP107H4
	10	Transatpks	CYP113L1
*Bacillus velezensis* AS43.3	6	Transatpks	CYP107K3
	7	Transatpks-Nrps	CYP113L1
	10	Transatpks	CYP113L1
*Bacillus velezensis* UCMB5036	5	Transatpks-Nrps	CYP107K3
	6	Transatpks-Nrps	CYP107H4
	9	Bacteriocin-Nrps	CYP113L1
*Bacillus velezensis* UCMB5033	6	Transatpks-Nrps	CYP107K3
	7	Transatpks-Nrps	CYP107H4
	10	Transatpks	CYP113L1
*Bacillus velezensis* UCMB5113	7	Transatpks-Nrps	CYP107K3
	8	Transatpks-Nrps	CYP107H4
	11	Transatpks	CYP113L1
*Bacillus velezensis* NAU-B3	3	Transatpks	CYP113L1
	6	Transatpks-Nrps	CYP107H4
	7	Transatpks-Nrps	CYP107K3
*Bacillus velezensis* TrigoCor1448	5	Transatpks-Nrps	CYP107K3
	6	Transatpks-Nrps	CYP107H4
*Bacillus velezensis* SQR9	6	Transatpks-Nrps	CYP107K3
	7	Transatpks-Nrps	CYP107H4
	10	Transatpks	CYP113L1
*Bacillus velezensis*	6	Transatpks-Nrps	CYP107K3
	7	Transatpks-Nrps	CYP107H4
	10	Transatpks	CYP113L1
*Bacillus amyloliquefaciens* DSM 7	5	Transatpks-Nrps	CYP107K3
	6	Transatpks-Nrps	CYP107H2
*Bacillus amyloliquefaciens* TA208	7	Transatpks-Nrps	CYP107H2
	8	Transatpks-Nrps	CYP107K3
*Bacillus amyloliquefaciens* LL3	5	Transatpks-Nrps	CYP107K3
	6	Transatpks-Nrps	CYP107H2
*Bacillus amyloliquefaciens* XH7	7	Transatpks-Nrps	CYP107H2
	8	Transatpks-Nrps	CYP107K3
*Bacillus amyloliquefaciens* Y2	6	Transatpks-Nrps	CYP107K3
	7	Transatpks-Nrps	CYP107H4
	10	Transatpks	CYP113L1
*Bacillus amyloliquefaciens* IT-45	3	Transatpks	CYP113L1
	6	Transatpks-Nrps	CYP107H4
	7	Transatpks-Nrps	CYP107K3
*Bacillus amyloliquefaciens* CC178	6	Transatpks-Nrps	CYP107H4
	9	Transatpks	CYP113L1
*Bacillus amyloliquefaciens* LFB112	7	Transatpks-Nrps	CYP107K3
	8	Transatpks-Nrps	CYP107H4
	11	Transatpks	CYP113L1
*Bacillus atrophaeus* 1942	3	Nrps-Transatpks-Otherks	CYP107K2
	10	Nrps	CYP152A9
*Bacillus atrophaeus NRS* 1221A	3	Nrps-Transatpks-Otherks	CYP107K2
	10	Nrps	CYP152A9
*Bacillus vallismortis*	6	Transatpks-Nrps	CYP107K3
	7	Transatpks-Nrps	CYP107H4
	10	Transatpks	CYP113L1
*Bacillus pumilus* SH-B9	8	Nrps	CYP109B6
*Bacillus* sp. JS	4	Nrps-Transatpks-Otherks	CYP107K1
*Bacillus* sp. Pc3	1	Bacteriocin-Transatpks-Nrps	CYP107H4
	2	Transatpks-Nrps	CYP107K3
	10	Transatpks	CYP113L1
*Bacillus* sp. BH072	8	Transatpks-Nrps	CYP107K3
	9	Transatpks-Nrps	CYP107H4
	12	Transatpks	CYP107H4
*Bacillus* sp. YP1	4	Nrps-Transatpks-Otherks	CYP107K1
*Bacillus* sp. BS34A	4	Nrps-Transatpks-Otherks	CYP107K1
	10	Other	CYP134A1
*Bacillus* sp. LM 4-2	3	Nrps-Transatpks-Otherks	CYP107K1
	7	Other	CYP102A48
	9	Other	CYP134A1
*Bacillus gibsonii*	1	Nrps-Transatpks-Otherks	CYP107K1
	6	Other	CYP134A1
	9	Lantipeptide	CYP152A1
*Bacillus xiamenensis*	2	Nrps	CYP1179A4
*Bacillus altitudinis*	2	Nrps	CYP1179A4
	8	Nrps	CYP109B5
*Bacillus* sp. SDLI1	3	Transatpks-Nrps	CYP107H4
	4	Transatpks-Nrps	CYP107K3
	11	Transatpks	CYP113L1

## Supplementary Materials

Supplementary materials can be found at http://www.mdpi.com/1422-0067/19/11/3623/s1.
