# Supplementary material for: Comparative Analyses of Cytochrome P450s and Those Associated with Secondary Metabolism in Bacillus Species
Source: Int J Mol Sci. 2018 Nov 16;19(11):3623. doi: 10.3390/ijms19113623 (PMC6275058; doi:10.3390/ijms19113623)
Supplement: Supplementary file 1 [file ijms-19-03623-s001.zip › Supplementary files/Mthethwa et al., 2018 Supplementary Information.docx]

**Comparative analyses of cytochrome P450s and those associated with secondary metabolism in *Bacillus* species**

**Bongumusa Comfort Mthethwa ^1^, Wanping Chen ^2^, Mathula Lancelot Ngwenya ^1^, Abidemi Paul Kappo ^1^, Puleng Rosinah Syed ^3^, Rajshekhar Karpoormath ^3^, Jae-Hyuk Yu ^4^, David R Nelson ^5^* and Khajamohiddin Syed ^1,^***

^1^ Department of Biochemistry and Microbiology, Faculty of Science and Agriculture, University of Zululand, KwaDlangezwa 3886, South Africa; 07bcomfort@gmail.com (B.C.M.); NgwenyaM@unizulu.ac.za (M.L.N.); KappoA@unizulu.ac.za (A.P.K.); khajamohiddinsyed@gmail.com (K.S.)

^2^ College of Food Science and Technology, Huazhong Agricultural University, Wuhan, Hubei Province, China; chenwanping@mail.hzau.edu.cn

^3^ Department of Pharmaceutical Chemistry, College of Health Sciences, University of KwaZulu-Natal, Durban 4000, South Africa; prosinah@gmail.com (P.R.S.); Karpoormath@ukzn.ac.za (R.K.)

^4^ Department of Bacteriology, University of Wisconsin-Madison, 3155 MSB, 1550 Linden Drive, Madison WI 53706, USA; jyu1@wisc.edu

^5^ Department of Microbiology, Immunology and Biochemistry, University of Tennessee Health Science Center, Memphis, TN, 38163; drnelson1@gmail.com

***** Correspondence: drnelson1@gmail.com (D.R.N.) and khajamohiddinsyed@gmail.com (K.S.)

**Table S1.** Comparative analysis of P450s at P450 family and subfamily level in the genus *Bacillus*.

| P450 family | Number of P450 subfamilies | P450 subfamily | Number of P450s | Total number of P450s |
| --- | --- | --- | --- | --- |
| CYP102 | 1 | A | 149 | 149 |
| CYP106 | 2 | A | 7 | 42 |
|  |  | B | 35 |  |
| CYP107 | 8 | CB | 3 | 170 |
|  |  | DE | 1 |  |
|  |  | DF | 1 |  |
|  |  | DY | 8 |  |
|  |  | H | 47 |  |
|  |  | J | 64 |  |
|  |  | JF | 1 |  |
|  |  | K | 45 |  |
| CYP109 | 6 | A | 17 | 80 |
|  |  | B | 45 |  |
|  |  | E | 4 |  |
|  |  | T | 12 |  |
|  |  | U | 1 |  |
|  |  | X | 1 |  |
| CYP113 | 1 | L | 19 | 19 |
| CYP1179 | 1 | A | 2 | 2 |
| CYP1221 | 1 | A | 3 | 3 |
| CYP1341 | 1 | C | 1 | 1 |
| CYP134 | 1 | A | 14 | 14 |
| CYP152 | 3 | A | 34 | 36 |
|  |  | K | 1 |  |
|  |  | N | 1 |  |
| CYP1756 | 1 | A | 4 | 4 |
| CYP197 | 1 | A | 1 | 1 |
| CYP223 | 1 | G | 2 | 2 |
| **13** | **28** | **28** | **523** | **523** |

**Table S2.** *Bacillus* species genome and plasmids IDs used in the study. Plasmids that have gene clusters were highlighted in bold letters.

| **Species Name** | **Species Code** | **Genome ID** | **No. of plasmids** | **Plasmids IDs** |
| --- | --- | --- | --- | --- |
| *Bacillus subtilis* subsp*. subtilis* 168 | bsu | NC_000964 |  |  |
| *Bacillus subtilis* subsp*. subtilis* RO-NN-1 | bsr | CP002906 |  |  |
| *Bacillus subtilis* subsp*. subtilis* BSP1 | bsl | CP003695 |  |  |
| *Bacillus subtilis* subsp. *subtilis* 6051-HGW | bsh | CP003329 |  |  |
| *Bacillus subtilis* subsp*. subtilis* BAB-1 | bsy | CP004405 |  |  |
| *Bacillus subtilis* subsp. *subtilis* AG1839 | bsut | CP008698 |  |  |
| *Bacillus subtilis* subsp. *subtilis* JH642 | bsul | CP007800 |  |  |
| *Bacillus subtilis* subsp. *subtilis* OH 131.1 | bsus | CP007409 |  |  |
| *Bacillus subtilis* subsp. *spizizenii* W23 | bss | CP002183 |  |  |
| *Bacillus subtilis* subsp*. spizizenii* TU-B-10 | bst | CP002905 |  |  |
| *Bacillus subtilis* subsp. *natto* BEST195 | bso | AP011541 |  |  |
| *Bacillus subtilis* BSn5 | bsn | CP002468 |  |  |
| *Bacillus subtilis* QB928 | bsq | CP003783 |  |  |
| *Bacillus subtilis* XF-1 | bsx | CP004019 |  |  |
| *Bacillus subtilis* PY79 | bsp | CP006881 |  |  |
| *Bacillus licheniformis* ATCC 14580 | bli | CP000002 |  |  |
| *Bacillus licheniformis* DSM 13 = ATCC 14580 | bld | AE017333 |  |  |
| *Bacillus paralicheniformis* | blh | CP005965 |  |  |
| *Bacillus velezensis* FZB42 | bay | CP000560 |  |  |
| *Bacillus velezensis* CAU B946 | baq | HE617159 |  |  |
| *Bacillus velezensis* YAU B9601-Y2 | bya | HE774679 |  |  |
| *Bacillus velezensis* AS43.3 | bamp | CP003838 |  |  |
| *Bacillus velezensis* UCMB5036 | baml | HF563562 |  |  |
| *Bacillus velezensis* UCMB5033 | bama | HG328253 |  |  |
| *Bacillus velezensis* UCMB5113 | bamn | HG328254 |  |  |
| *Bacillus velezensis* NAU-B3 | bamb | HG514499 |  |  |
| *Bacillus velezensis TrigoCor*1448 | bamt | CP007244 |  |  |
| *Bacillus velezensis* SQR9 | bamy | CP006890 |  |  |
| *Bacillus velezensis* | bmp | CP009679 | 1 | CP009680 |
| *Bacillus amyloliquefaciens* DSM 7 | bao | FN597644 |  |  |
| *Bacillus amyloliquefaciens* TA208 | baz | CP002627 |  |  |
| *Bacillus amyloliquefaciens* LL3 | bql | CP002634 | 1 | CP002635 |
| *Bacillus amyloliquefaciens* XH7 | bxh | CP002927 |  |  |
| *Bacillus amyloliquefaciens* Y2 | bqy | CP003332 |  |  |
| *Bacillus amyloliquefaciens* IT-45 | bami | CP004065 |  |  |
| *Bacillus amyloliquefaciens* CC178 | bamc | CP006845 |  |  |
| *Bacillus amyloliquefaciens* LFB112 | bamf | CP006952 |  |  |
| *Bacillus atrophaeus* 1942 | bae | CP002207 |  |  |
| *Bacillus atrophaeus* NRS 1221A | batr | CP010778 |  |  |
| *Bacillus vallismortis* | bvm | CP020893 |  |  |
| *Bacillus halodurans* | bha | CP001215 | 4 | CP001216; CP001214; CP001216; CP001214 |
| *Bacillus anthracis Ames* | ban | NC_003997 |  |  |
| *Bacillus anthracis Ames Ancestor* | bar | AE017334 | 2 | AE017336; AE017335 |
| *Bacillus anthracis Sterne* | bat | NC_005945 |  |  |
| *Bacillus anthracis* CDC 684 | bah | CP001215 | 2 | CP001216; CP001214 |
| *Bacillus anthracis* A0248 | bai | CP001598 | 1 | CP001597 |
| *Bacillus anthracis* H9401 | bax | CP002091 | 2 | \| CP002092 \| CP002093 \| \| --- \| --- \| |
| *Bacillus anthracis* A16 | bant | CP001970 | 2 | \| CP001971 \| CP001972 \| \| --- \| --- \| |
| *Bacillus anthracis* A16R | banr | CP001974 | 1 | CP001975 |
| *Bacillus anthracis* SVA11 | bans | CP006742 | 2 | \| CP006743 \| CP006744 \| \| --- \| --- \| |
| *Bacillus anthracis* HYU01 | banh | CP008846 | 2 | \| CP008847 \| CP008848 \| \| --- \| --- \| |
| *Bacillus anthracis Vollum* | banv | CP007666 | 2 | CP007665 CP007664 |
| *Bacillus cereus* ATCC 14579 | bce | NC_004722 | 1 | NC_004721 |
| *Bacillus cereus* ATCC 10987 | bca | AE017194 | 1 | AE017195 |
| *Bacillus cereus* E33L | bcz | CP000001 | 5 | \| CP000040 \| CP000041 \| CP000042 \| CP000043 \| CP000044 \| \| --- \| --- \| --- \| --- \| --- \| |
| *Bacillus cereus* AH187 | bcr | CP001177 | **4** | **CP001179**; CP001180; CP001178; CP001181 |
| *Bacillus cereus* B4264 | bcb | CP001176 |  |  |
| *Bacillus cereus* AH820 | bcu | CP001283 | 3 | \| CP001285 \| CP001286 \| CP001284 \| \| --- \| --- \| --- \| |
| *Bacillus cereus* G9842 | bcg | CP001186 | 2 | \| CP001188 \| CP001187 \| \| --- \| --- \| |
| *Bacillus cereus* Q1 | bcq | CP000227 | **2** | \| **CP000228** \| 19060151 \| \| --- \| --- \| |
| *Bacillus cereus* 03BB102 | bcx | CP001407 | 1 | CP001406 |
| *Bacillus cereus biovar anthracis* CI | bal | CP001746 | 3 | \| CP001747 \| CP001748 \| CP001749 \| \| --- \| --- \| --- \| |
| *Bacillus cereus* NC7401 | bnc | AP007209 | 5 | \| AP007211 \| AP007212 \| AP007213 \| AP007214 \| **AP007210** \| \| --- \| --- \| --- \| --- \| --- \| |
| *Bacillus cereus* F837/76 | bcf | CP003187 | 2 | \| CP003189 \| CP003188 \| \| --- \| --- \| |
| *Bacillus cereus* FRI-35 | bcer | CP003747 | 3 | \| CP003748 \| **CP003749** \| CP003750 \| CP003751 \| \| --- \| --- \| --- \| --- \| |
| *Bacillus cereus* FT9 | bcef | CP008712 |  |  |
| *Bacillus cytotoxicus* | bcy | CP000764 | 1 | CP000765 |
| *Bacillus thuringiensis serovar konkukian* 97-27 | btk | NC_005957 | 1 | NC_006578 |
| *Bacillus thuringiensis Al Hakam* | btl | CP000485 | 1 | CP000486 |
| *Bacillus thuringiensis* BMB171 | btb | CP001903 | **1** | **CP001904** |
| *Bacillus thuringiensis serovar kurstaki* HD73 | btt | CP004069 | 7 | CP004070 CP004071 CP004072 CP004073 CP004074 CP004075  CP004076 |
| *Bacillus thuringiensis serovar kurstaki* YBT-1520 | bthr | CP004858 | **11** | **CP004860**  CP004861  CP004868 CP004869 CP004862 CP004863 CP004864 CP004865 CP004866 CP004867 CP004859 |
| *Bacillus thuringiensis serovar kurstaki* HD-1 | bthi | CP004870 | **13** | **CP004877** CP004876 CP004875 CP004874 CP004873 CP004872 CP004871 CP004882 CP004883 CP004881 CP004880 CP004879 CP004878 |
| *Bacillus thuringiensis serovar chinensis* CT-43 | btc | CP001907 | **10** | **CP001908** CP001909 CP001910 CP001911 CP001912 CP001913 CP001914 CP001915 CP001916 CP001917 |
| *Bacillus thuringiensis serovar finitimus* YBT-020 | btf | CP002508 | 2 | \| CP002509 \| **CP002510** \|  \| \| --- \| --- \| --- \| |
| *Bacillus thuringiensis* MC28 | btm | CP003687 | 7 | CP003688 CP003689 **CP003690** CP003691 CP003692 **CP003693** CP003694 |
| *Bacillus thuringiensis* Bt407 | btg | CP003889 | 9 | CP003897 CP003896 CP003895 CP003894 CP003893 CP003898 CP003892 CP003891 CP003890 |
| *Bacillus thuringiensis* HD-771 | bti | CP003752 | 8 | CP003753 CP003754 CP003755 CP003756 CP003757 CP003758 CP003759 CP003760 |
| *Bacillus thuringiensis* HD-789 | btn | CP003763 | 6 | CP003764 CP003765 CP003766 CP003767 CP003768 **CP003769** |
| *Bacillus thuringiensis serovar thuringiensis* IS5056 | btht | CP004123 | 14 | CP004124 CP004125 CP004126  CP004127 CP004128 CP004129 CP004130 CP004131 CP004132 CP004133  **CP004134** **CP004135** CP004136 **CP004137** |
| *Bacillus thuringiensis* YBT-1518 | bthu | CP005935 | 6 | CP002486 CP005936 CP005937 CP005938 CP005939 CP005940 |
| *Bacillus thuringiensis* HD1011 | btw | CP009335 | **4** | **CP009336** CP009334  **CP009332** CP009333 |
| *Bacillus thuringiensis* YWC2-8 | bthy | CP013055 | 6 | CP013056 CP013057 CP013058 CP013059 CP013060 CP013061 |
| *Bacillus mycoides* KBAB4 | bwe | CP000903 | **4** | **CP000904** CP000905 CP000906 CP000907 |
| *Bacillus mycoides* WSBC 10204 | bww | CP009746 |  |  |
| *Bacillus mycoides* 219298 | bmyc | CP007626 | 4 | CP007621 CP007623 CP007624 CP007625 |
| *Bacillus mycoides* ATCC 6462 | bmyo | CP009692 | **3** | **CP009691**  CP009690 CP009689 |
| *Bacillus toyonensis* | bty | CP006863 | 2 | CP006864 CP006865 |
| *Bacillus clausii* | bcl | AP006627 |  |  |
| *Bacillus pumilus* SAFR-032 | bpu | CP000813 |  |  |
| *Bacillus pumilus* MTCC B6033 | bpum | CP007436 |  |  |
| *Bacillus pumilus* SH-B9 | bpus | CP011007 | 1 | CP011023 |
| *Bacillus pseudofirmus* | bpf | CP001878 | 2 | CP001879 CP001880 |
| *Bacillus megaterium* QM B1551 | bmq | CP001983 | 6 | CP001984  CP001985  CP001986  CP001987 **CP001988** CP001989 |
| *Bacillus megaterium* DSM 319 | bmd | CP001982 |  |  |
| *Bacillus megaterium* WSH-002 | bmh | CP003017 | 2 | CP003018  CP003019  CP003020 |
| *Bacillus megaterium* NBRC 15308 = ATCC 14581 | bmeg | CP009920 | 6 | CP009919  **CP009921** CP009915  CP009918 CP009916 CP009917 |
| *Bacillus cellulosilyticus* | bco | CP002394 |  |  |
| *Bacillus coagulans* 2-6 | bck | CP002472 |  |  |
| *Bacillus coagulans* 36D1 | bag | CP003056 |  |  |
| *Bacillus coagulans* DSM 1 = ATCC 7050 | bcoa | CP009709 |  |  |
| *Bacillus sp. JS* | bjs | CP003492 |  |  |
| *Bacillus sp.* 1NLA3E | baci | CP005586 |  |  |
| *Bacillus infantis* | bif | CP006643 |  |  |
| *Bacillus lehensis* | ble | CP003923 |  |  |
| *Bacillus methanolicus* | bmet | CP007739 | 2 | CP007740  CP007741 |
| *Bacillus sp.* X1(2014) | bsg | CP008855 |  |  |
| *Bacillus sp.* WP8 | bacw | CP010075 |  |  |
| *Bacillus sp*. Pc3 | bacp | CP010406 |  |  |
| *Bacillus sp.* BH072 | bacb | CP009938 |  |  |
| *Bacillus bombysepticus* | bby | CP007512 |  |  |
| *Bacillus sp.* OxB-1 | baco | AP013294 |  |  |
| *Bacillus sp.* YP1 | bacy | CP010014 | 1 | CP010015 |
| *Bacillus sp.* BS34A | bacl | LN680001 |  |  |
| *Bacillus sp.* LM 4-2 | balm | CP011101 |  |  |
| *Bacillus endophyticus* | beo | CP011974 |  |  |
| *Bacillus smithii* | bsm | CP012024 | 1 | CP012025 |
| *Bacillus simplex* | bsj | CP011008 | 1 | CP011009 |
| *Bacillus oceanisediminis* | bon | CP015506 | 1 | CP015507 |
| *Bacillus glycinifermentans* | bgy | LT603683 |  |  |
| *Bacillus flexus* | bfx | CP016790 | 2 | CP016791  CP016792 |
| *Bacillus gibsonii* | bgi | CP017070 |  |  |
| *Bacillus weihaiensis* | bwh | CP016020 | 1 | CP016021 |
| *Bacillus xiamenensis* | bxi | CP017786 |  |  |
| *Bacillus horikoshii* | bhk | CP020880 | 1 | CP020881 |
| *Bacillus krulwichiae* | bkw | CP020814 |  |  |
| *Bacillus beveridgei* | bbev | CP012502 |  |  |
| *Bacillus kochii* | bko | CP022983 | 1 | CP022984 |
| *Bacillus altitudinis* | balt | CP022319 | 1 | CP022320 |
| *Bacillus sp.* SDLI1 | bacs | CP013950 |  |  |

**Table S3.** Secondary metabolite gene-clusters analysis in the genus *Bacillus*. Gene clusters present on the genomic DNA and the plasmid were shown in the table.

| Species Name | Species code | Total no. of gene-clusters | No. of gene-clusters on Genomic DNA | No. of gene-clusters on plasmid(s) |
| --- | --- | --- | --- | --- |
| *Bacillus thuringiensis serovar thuringiensis* IS5056 | btht | 13 | 9 | 4 |
| *Bacillus thuringiensis serovar kurstaki* HD-1 | bthi | 11 | 9 | 2 |
| *Bacillus thuringiensis serovar kurstaki* YBT-1520 | bthr | 11 | 9 | 2 |
| *Bacillus thuringiensis* HD-771 | bti | 12 | 12 |  |
| *Bacillus thuringiensis serovar chinensis* CT-43 | btc | 10 | 9 | 1 |
| *Bacillus thuringiensis* Bt407 | btg | 9 | 9 |  |
| *Bacillus thuringiensis serovar kurstaki* HD73 | btt | 10 | 10 |  |
| *Bacillus thuringiensis* HD-789 | btn | 13 | 11 | 2 |
| *Bacillus thuringiensis* YBT-1518 | bthu | 11 | 11 |  |
| *Bacillus thuringiensis* MC28 | btm | 11 | 9 | 2 |
| *Bacillus thuringiensis* YWC2-8 | bthy | 10 | 10 |  |
| *Bacillus amyloliquefaciens* LL3 | bql | 10 | 10 |  |
| *Bacillus velezensis* YAU B9601-Y2 | bya | 14 | 14 |  |
| *Bacillus velezensis* UCMB5113 | bamn | 14 | 14 |  |
| *Bacillus amyloliquefaciens Y2* | bqy | 14 | 14 |  |
| *Bacillus megaterium* NBRC 15308 = ATCC 14581 | bmeg | 9 | 8 | 1 |
| *Bacillus sp*. YP1 | bacy | 13 | 13 |  |
| *Bacillus velezensis* SQR9 | bamy | 12 | 12 |  |
| *Bacillus amyloliquefaciens* LFB112 | bamf | 13 | 13 |  |
| *Bacillus cereus* G9842 | bcg | 11 | 11 |  |
| *Bacillus megaterium* QM B1551 | bmq | 8 | 7 | 1 |
| *Bacillus sp*. BH072 | bacb | 13 | 13 |  |
| *Bacillus subtilis* subsp. *subtilis* 168 | bsu | 12 | 12 |  |
| *Bacillus subtilis* subsp. *subtilis* 6051-HGW | bsh | 12 | 12 |  |
| *Bacillus subtilis* subsp. *subtilis* AG1839 | bsut | 12 | 12 |  |
| *Bacillus subtilis* subsp. *subtilis* JH642 | bsul | 12 | 12 |  |
| *Bacillus subtilis* QB928 | bsq | 12 | 12 |  |
| *Bacillus paralicheniformis* | blh | 12 | 12 |  |
| *Bacillus velezensis* FZB42 | bay | 12 | 12 |  |
| *Bacillus velezensis* AS43.3 | bamp | 12 | 12 |  |
| *Bacillus velezensis* UCMB5036 | baml | 11 | 11 |  |
| *Bacillus velezensis* UCMB5033 | bama | 12 | 12 |  |
| *Bacillus velezensis* NAU-B3 | bamb | 13 | 13 |  |
| *Bacillus velezensis* TrigoCor1448 | bamt | 12 | 12 |  |
| *Bacillus velezensis* | bmp | 13 | 13 |  |
| *Bacillus amyloliquefaciens* XH7 | bxh | 10 | 10 |  |
| *Bacillus amyloliquefaciens* IT-45 | bami | 12 | 12 |  |
| *Bacillus amyloliquefaciens* CC178 | bamc | 12 | 12 |  |
| *Bacillus atrophaeus* 1942 | bae | 12 | 12 |  |
| *Bacillus atrophaeus* NRS 1221A | batr | 12 | 12 |  |
| *Bacillus vallismortis* | bvm | 12 | 12 |  |
| *Bacillus mycoides* KBAB4 | bwe | 10 | 8 | 2 |
| *Bacillus mycoides* ATCC 6462 | bmyo | 10 | 9 | 1 |
| *Bacillus sp. JS* | bjs | 11 | 11 |  |
| *Bacillus sp.* Pc3 | bacp | 12 | 12 |  |
| *Bacillus sp.* BS34A | bacl | 12 | 12 |  |
| *Bacillus gibsonii* | bgi | 11 | 11 |  |
| *Bacillus subtilis* subsp*. subtilis* BSP1 | bsl | 11 | 11 |  |
| *Bacillus subtilis* subsp*. subtilis* OH 131.1 | bsus | 11 | 11 |  |
| *Bacillus subtilis* subsp*. spizizenii* W23 | bss | 11 | 11 |  |
| *Bacillus subtilis s*ubsp*. spizizenii* TU-B-10 | bst | 11 | 11 |  |
| *Bacillus subtilis* BSn5 | bsn | 11 | 11 |  |
| *Bacillus subtilis* PY79 | bsp | 11 | 11 |  |
| *Bacillus velezensis* CAU B946 | baq | 11 | 11 |  |
| *Bacillus mycoides* WSBC 10204 | bww | 11 | 11 |  |
| *Bacillus lehensis* | ble | 11 | 11 |  |
| *Bacillus sp.* LM 4-2 | balm | 11 | 11 |  |
| *Bacillus sp.* SDLI1 | bacs | 11 | 11 |  |
| *Bacillus subtilis* subsp*. subtilis* BAB-1 | bsy | 10 | 10 |  |
| *Bacillus subtilis* XF-1 | bsx | 10 | 10 |  |
| *Bacillus licheniformis* ATCC 14580 | bli | 10 | 10 |  |
| *Bacillus licheniformis* DSM 13 = ATCC 14580 | bld | 10 | 10 |  |
| *Bacillus amyloliquefaciens* DSM 7 | bao | 11 | 11 |  |
| *Bacillus amyloliquefaciens* TA208 | baz | 10 | 10 |  |
| *Bacillus cereus* ATCC 14579 | bce | 9 | 9 |  |
| *Bacillus cereus* AH187 | bcr | 8 | 7 | 1 |
| *Bacillus cereus A*H820 | bcu | 7 | 7 |  |
| *Bacillus cereus* NC7401 | bnc | 8 | 7 | 1 |
| *Bacillus thuringiensis* HD1011 | btw | 8 | 6 | 2 |
| *Bacillus mycoides* 219298 | bmyc | 6 | 6 |  |
| *Bacillus subtilis subsp. subtilis* RO-NN-1 | bsr | 9 | 9 |  |
| *Bacillus subtilis* subsp*. natto* BEST195 | bso | 9 | 9 |  |
| *Bacillus anthracis* H9401 | bax | 7 | 7 |  |
| *Bacillus cereus* E33L | bcz | 6 | 6 |  |
| *Bacillus cereus* Q1 | bcq | 8 | 7 | 1 |
| *Bacillus cereus* F837/76 | bcf | 7 | 7 |  |
| *Bacillus thuringiensis* BMB171 | btb | 9 | 8 | 1 |
| *Bacillus thuringiensis serovar finitimus* YBT-020 | btf | 8 | 7 | 1 |
| *Bacillus toyonensis* | bty | 7 | 7 |  |
| *Bacillus pumilus* SH-B9 | bpus | 8 | 8 |  |
| *Bacillus coagulans* DSM 1 = ATCC 7050 | bcoa | 3 | 3 |  |
| *Bacillus sp.* 1NLA3E | baci | 9 | 9 |  |
| *Bacillus bombysepticus* | bby | 9 | 9 |  |
| *Bacillus endophyticus* | beo | 9 | 9 |  |
| *Bacillus simplex* | bsj | 8 | 8 |  |
| *Bacillus kochii* | bko | 8 | 8 |  |
| *Bacillus altitudinis* | balt | 8 | 8 |  |
| *Bacillus halodurans* | bha | 6 | 6 |  |
| *Bacillus anthracis* CDC 684 | bah | 6 | 6 |  |
| *Bacillus anthracis* A16 | bant | 6 | 6 |  |
| *Bacillus anthracis* SVA11 | bans | 6 | 6 |  |
| *Bacillus anthracis Vollum* | banv | 6 | 6 |  |
| *Bacillus cereus* B4264 | bcb | 8 | 8 |  |
| *Bacillus cereus biovar anthracis* CI | bal | 5 | 5 |  |
| *Bacillus cereus* FRI-35 | bcer | 6 | 5 | 1 |
| *Bacillus pumilus* MTCC B6033 | bpum | 8 | 8 |  |
| *Bacillus megaterium* WSH-002 | bmh | 5 | 5 |  |
| *Bacillus glycinifermentans* | bgy | 8 | 8 |  |
| *Bacillus flexus* | bfx | 6 | 6 |  |
| *Bacillus anthracis Ames Ancestor* | bar | 5 | 5 |  |
| *Bacillus anthracis* A16R | banr | 6 | 6 |  |
| *Bacillus anthracis* HYU01 | banh | 5 | 5 |  |
| *Bacillus cereus* ATCC 10987 | bca | 6 | 6 |  |
| *Bacillus cytotoxicus* | bcy | 6 | 6 |  |
| *Bacillus thuringiensis Al Hakam* | btl | 6 | 6 |  |
| *Bacillus pumilus* SAFR-032 | bpu | 7 | 7 |  |
| *Bacillus pseudofirmus* | bpf | 5 | 5 |  |
| *Bacillus megaterium* DSM 319 | bmd | 7 | 7 |  |
| *Bacillus methanolicus* | bmet | 5 | 5 |  |
| *Bacillus oceanisediminis* | bon | 6 | 6 |  |
| *Bacillus anthracis Sterne* | bat | 6 | 6 |  |
| *Bacillus anthracis* A0248 | bai | 5 | 5 |  |
| *Bacillus cereus* 03BB102 | bcx | 5 | 5 |  |
| *Bacillus thuringiensis serovar konkukian* 97-27 | btk | 5 | 5 |  |
| *Bacillus sp.* WP8 | bacw | 6 | 6 |  |
| *Bacillus weihaiensis* | bwh | 5 | 5 |  |
| *Bacillus horikoshii* | bhk | 5 | 5 |  |
| *Bacillus anthracis Ames* | ban | 5 | 5 |  |
| *Bacillus infantis* | bif | 5 | 5 |  |
| *Bacillus xiamenensis* | bxi | 5 | 5 |  |
| *Bacillus cereus* FT9 | bcef | 4 | 4 |  |
| *Bacillus clausii* | bcl | 4 | 4 |  |
| *Bacillus cellulosilyticus* | bco | 4 | 4 |  |
| *Bacillus sp.* X1(2014) | gst | 4 | 4 |  |
| *Bacillus smithii* | bsm | 3 | 3 |  |
| *Bacillus sp.* OxB-1 | baco | 3 | 3 |  |
| *Bacillus krulwichiae* | bkw | 2 | 2 |  |
| *Bacillus beveridgei* | bbev | 2 | 2 |  |
| *Bacillus coagulans* 36D1 | bag | 1 | 1 |  |
|  |  | 1124 | 1098 | 26 |

**Table S4.** *Bacillus* species used in the study. Species names, codes and genome database links used in the study were presented in the table.

| Species Name | Species Code | Genomic database link |
| --- | --- | --- |
| *Bacillus subtilis* subsp*. subtilis* 168 | bsu | <https://www.genome.jp/kegg-bin/show_organism?org=bsu> |
| *Bacillus subtilis* subsp*. subtilis* RO-NN-1 | bsr | <https://www.genome.jp/kegg-bin/show_organism?org=bsr> |
| *Bacillus subtilis* subsp*. subtilis BSP1* | bsl | <https://www.genome.jp/kegg-bin/show_organism?org=bsl> |
| *Bacillus subtilis* subsp*. subtilis* 6051-HGW | bsh | <https://www.genome.jp/kegg-bin/show_organism?org=bsh> |
| *Bacillus subtilis* subsp*. subtilis* BAB-1 | bsy | <https://www.genome.jp/kegg-bin/show_organism?org=bsy> |
| *Bacillus subtilis* subsp*. subtilis* AG1839 | bsut | <https://www.genome.jp/kegg-bin/show_organism?org=bsut> |
| *Bacillus subtilis* subsp. *subtilis* JH642 | bsul | <https://www.genome.jp/kegg-bin/show_organism?org=bsul> |
| *Bacillus subtilis* subsp*. subtilis* OH 131.1 | bsus | <https://www.genome.jp/kegg-bin/show_organism?org=bsus> |
| *Bacillus subtilis* subsp*. spizizenii* W23 | bss | <https://www.genome.jp/kegg-bin/show_organism?org=bss> |
| *Bacillus subtilis* subsp*. spizizenii* TU-B-10 | bst | <https://www.genome.jp/kegg-bin/show_organism?org=bst> |
| *Bacillus subtilis* subsp*. natto* BEST195 | bso | <https://www.genome.jp/kegg-bin/show_organism?org=bso> |
| *Bacillus subtilis* BSn5 | bsn | <https://www.genome.jp/kegg-bin/show_organism?org=bsn> |
| *Bacillus subtilis* QB928 | bsq | <https://www.genome.jp/kegg-bin/show_organism?org=bsq> |
| *Bacillus subtilis* XF-1 | bsx | <https://www.genome.jp/kegg-bin/show_organism?org=bsx> |
| *Bacillus subtilis* PY79 | bsp | <https://www.genome.jp/kegg-bin/show_organism?org=bsp> |
| *Bacillus licheniformis* ATCC 14580 | bli | <https://www.genome.jp/kegg-bin/show_organism?org=bli> |
| *Bacillus licheniformis* DSM 13 = ATCC 14580 | bld | <https://www.genome.jp/kegg-bin/show_organism?org=bld> |
| *Bacillus paralicheniformis* | blh | <https://www.genome.jp/kegg-bin/show_organism?org=blh> |
| *Bacillus velezensis* FZB42 | bay | <https://www.genome.jp/kegg-bin/show_organism?org=bay> |
| *Bacillus velezensis* CAU B946 | baq | <https://www.genome.jp/kegg-bin/show_organism?org=baq> |
| *Bacillus velezensis* YAU B9601-Y2 | bya | <https://www.genome.jp/kegg-bin/show_organism?org=bya> |
| *Bacillus velezensis* AS43.3 | bamp | <https://www.genome.jp/kegg-bin/show_organism?org=bamp> |
| *Bacillus velezensis* UCMB5036 | baml | <https://www.genome.jp/kegg-bin/show_organism?org=baml> |
| *Bacillus velezensis* UCMB5033 | bama | <https://www.genome.jp/kegg-bin/show_organism?org=bama> |
| *Bacillus velezensis* UCMB5113 | bamn | <https://www.genome.jp/kegg-bin/show_organism?org=bamn> |
| *Bacillus velezensis* NAU-B3 | bamb | <https://www.genome.jp/kegg-bin/show_organism?org=bamb> |
| *Bacillus velezensis* TrigoCor1448 | bamt | <https://www.genome.jp/kegg-bin/show_organism?org=bamt> |
| *Bacillus velezensis* SQR9 | bamy | <https://www.genome.jp/kegg-bin/show_organism?org=bamy> |
| *Bacillus velezensis* | bmp | <https://www.genome.jp/kegg-bin/show_organism?org=bmp> |
| *Bacillus amyloliquefaciens* DSM 7 | bao | <https://www.genome.jp/kegg-bin/show_organism?org=bao> |
| *Bacillus amyloliquefaciens* TA208 | baz | <https://www.genome.jp/kegg-bin/show_organism?org=baz> |
| *Bacillus amyloliquefaciens* LL3 | bql | <https://www.genome.jp/kegg-bin/show_organism?org=bql> |
| *Bacillus amyloliquefaciens* XH7 | bxh | <https://www.genome.jp/kegg-bin/show_organism?org=bxh> |
| *Bacillus amyloliquefaciens* Y2 | bqy | <https://www.genome.jp/kegg-bin/show_organism?org=bqy> |
| *Bacillus amyloliquefaciens* IT-45 | bami | <https://www.genome.jp/kegg-bin/show_organism?org=bami> |
| *Bacillus amyloliquefaciens* CC178 | bamc | <https://www.genome.jp/kegg-bin/show_organism?org=bamc> |
| *Bacillus amyloliquefaciens* LFB112 | bamf | <https://www.genome.jp/kegg-bin/show_organism?org=bamf> |
| *Bacillus atrophaeus* 1942 | bae | <https://www.genome.jp/kegg-bin/show_organism?org=bae> |
| *Bacillus atrophaeus* NRS 1221A | batr | <https://www.genome.jp/kegg-bin/show_organism?org=batr> |
| *Bacillus vallismortis* | bvm | <https://www.genome.jp/kegg-bin/show_organism?org=bvm> |
| *Bacillus halodurans* | bha | <https://www.genome.jp/kegg-bin/show_organism?org=bha> |
| *Bacillus anthracis Ames* | ban | <https://www.genome.jp/kegg-bin/show_organism?org=ban> |
| *Bacillus anthracis Ames Ancestor* | bar | <https://www.genome.jp/kegg-bin/show_organism?org=bar> |
| *Bacillus anthracis Sterne* | bat | <https://www.genome.jp/kegg-bin/show_organism?org=bat> |
| *Bacillus anthracis* CDC 684 | bah | <https://www.genome.jp/kegg-bin/show_organism?org=bah> |
| *Bacillus anthracis* A0248 | bai | <https://www.genome.jp/kegg-bin/show_organism?org=bai> |
| *Bacillus anthracis* H9401 | bax | <https://www.genome.jp/kegg-bin/show_organism?org=bax> |
| *Bacillus anthracis* A16 | bant | <https://www.genome.jp/kegg-bin/show_organism?org=bant> |
| *Bacillus anthracis* A16R | banr | <https://www.genome.jp/kegg-bin/show_organism?org=banr> |
| *Bacillus anthracis* SVA11 | bans | <https://www.genome.jp/kegg-bin/show_organism?org=bans> |
| *Bacillus anthracis* HYU01 | banh | <https://www.genome.jp/kegg-bin/show_organism?org=banh> |
| *Bacillus anthracis Vollum* | banv | <https://www.genome.jp/kegg-bin/show_organism?org=banv> |
| *Bacillus cereus* ATCC 14579 | bce | <https://www.genome.jp/kegg-bin/show_organism?org=bce> |
| *Bacillus cereus* ATCC 10987 | bca | <https://www.genome.jp/kegg-bin/show_organism?org=bca> |
| *Bacillus cereus* E33L | bcz | <https://www.genome.jp/kegg-bin/show_organism?org=bcz> |
| *Bacillus cereus* AH187 | bcr | <https://www.genome.jp/kegg-bin/show_organism?org=bcr> |
| *Bacillus cereus* B4264 | bcb | <https://www.genome.jp/kegg-bin/show_organism?org=bcb> |
| *Bacillus cereus* AH820 | bcu | <https://www.genome.jp/kegg-bin/show_organism?org=bcu> |
| *Bacillus cereus* G9842 | bcg | <https://www.genome.jp/kegg-bin/show_organism?org=bcg> |
| *Bacillus cereus* Q1 | bcq | <https://www.genome.jp/kegg-bin/show_organism?org=bcq> |
| *Bacillus cereus* 03BB102 | bcx | <https://www.genome.jp/kegg-bin/show_organism?org=bcx> |
| *Bacillus cereus biovar anthracis* CI | bal | <https://www.genome.jp/kegg-bin/show_organism?org=bal> |
| *Bacillus cereus* NC7401 | bnc | <https://www.genome.jp/kegg-bin/show_organism?org=bnc> |
| *Bacillus cereus* F837/76 | bcf | <https://www.genome.jp/kegg-bin/show_organism?org=bcf> |
| *Bacillus cereus* FRI-35 | bcer | <https://www.genome.jp/kegg-bin/show_organism?org=bcer> |
| *Bacillus cereus* FT9 | bcef | <https://www.genome.jp/kegg-bin/show_organism?org=bcef> |
| *Bacillus cytotoxicus* | bcy | <https://www.genome.jp/kegg-bin/show_organism?org=bcy> |
| *Bacillus thuringiensis serovar konkukian* 97-27 | btk | <https://www.genome.jp/kegg-bin/show_organism?org=btk> |
| *Bacillus thuringiensis Al Hakam* | btl | <https://www.genome.jp/kegg-bin/show_organism?org=btl> |
| *Bacillus thuringiensis* BMB171 | btb | <https://www.genome.jp/kegg-bin/show_organism?org=btb> |
| *Bacillus thuringiensis serovar kurstaki* HD73 | btt | <https://www.genome.jp/kegg-bin/show_organism?org=btt> |
| *Bacillus thuringiensis serovar kurstaki* YBT-1520 | bthr | <https://www.genome.jp/kegg-bin/show_organism?org=bthr> |
| *Bacillus thuringiensis serovar kurstaki* HD-1 | bthi | <https://www.genome.jp/kegg-bin/show_organism?org=bthi> |
| *Bacillus thuringiensis serovar chinensis* CT-43 | btc | <https://www.genome.jp/kegg-bin/show_organism?org=btc> |
| *Bacillus thuringiensis serovar finitimus* YBT-020 | btf | <https://www.genome.jp/kegg-bin/show_organism?org=btf> |
| *Bacillus thuringiensis* MC28 | btm | <https://www.genome.jp/kegg-bin/show_organism?org=btm> |
| *Bacillus thuringiensis* Bt407 | btg | <https://www.genome.jp/kegg-bin/show_organism?org=btg> |
| *Bacillus thuringiensis* HD-771 | bti | <https://www.genome.jp/kegg-bin/show_organism?org=bti> |
| *Bacillus thuringiensis* HD-789 | btn | <https://www.genome.jp/kegg-bin/show_organism?org=btn> |
| *Bacillus thuringiensis serovar thuringiensis* IS5056 | btht | <https://www.genome.jp/kegg-bin/show_organism?org=btht> |
| *Bacillus thuringiensis* YBT-1518 | bthu | <https://www.genome.jp/kegg-bin/show_organism?org=bthu> |
| *Bacillus thuringiensis* HD1011 | btw | <https://www.genome.jp/kegg-bin/show_organism?org=btw> |
| *Bacillus thuringiensis* YWC2-8 | bthy | <https://www.genome.jp/kegg-bin/show_organism?org=bthy> |
| *Bacillus mycoides* KBAB4 | bwe | <https://www.genome.jp/kegg-bin/show_organism?org=bwe> |
| *Bacillus mycoides* WSBC 10204 | bww | <https://www.genome.jp/kegg-bin/show_organism?org=bww> |
| *Bacillus mycoides* 219298 | bmyc | <https://www.genome.jp/kegg-bin/show_organism?org=bmyc> |
| *Bacillus mycoides* ATCC 6462 | bmyo | <https://www.genome.jp/kegg-bin/show_organism?org=bmyo> |
| *Bacillus toyonensis* | bty | <https://www.genome.jp/kegg-bin/show_organism?org=bty> |
| *Bacillus clausii* | bcl | <https://www.genome.jp/kegg-bin/show_organism?org=bcl> |
| *Bacillus pumilus* SAFR-032 | bpu | <https://www.genome.jp/kegg-bin/show_organism?org=bpu> |
| *Bacillus pumilus* MTCC B6033 | bpum | <https://www.genome.jp/kegg-bin/show_organism?org=bpum> |
| *Bacillus pumilus* SH-B9 | bpus | <https://www.genome.jp/kegg-bin/show_organism?org=bpus> |
| *Bacillus pseudofirmus* | bpf | <https://www.genome.jp/kegg-bin/show_organism?org=bpf> |
| *Bacillus megaterium* QM B1551 | bmq | <https://www.genome.jp/kegg-bin/show_organism?org=bmq> |
| *Bacillus megaterium* DSM 319 | bmd | <https://www.genome.jp/kegg-bin/show_organism?org=bmd> |
| *Bacillus megaterium* WSH-002 | bmh | <https://www.genome.jp/kegg-bin/show_organism?org=bmh> |
| *Bacillus megaterium* NBRC 15308 = ATCC 14581 | bmeg | <https://www.genome.jp/kegg-bin/show_organism?org=bmeg> |
| *Bacillus cellulosilyticus* | bco | <https://www.genome.jp/kegg-bin/show_organism?org=bco> |
| *Bacillus coagulans* 2-6 | bck | <https://www.genome.jp/kegg-bin/show_organism?org=bck> |
| *Bacillus coagulans* 36D1 | bag | <https://www.genome.jp/kegg-bin/show_organism?org=bag> |
| *Bacillus coagulans* DSM 1 = ATCC 7050 | bcoa | <https://www.genome.jp/kegg-bin/show_organism?org=bcoa> |
| *Bacillus sp*. JS | bjs | <https://www.genome.jp/kegg-bin/show_organism?org=bjs> |
| *Bacillus sp.* 1NLA3E | baci | <https://www.genome.jp/kegg-bin/show_organism?org=baci> |
| *Bacillus infantis* | bif | <https://www.genome.jp/kegg-bin/show_organism?org=bif> |
| *Bacillus lehensis* | ble | <https://www.genome.jp/kegg-bin/show_organism?org=ble> |
| *Bacillus methanolicus* | bmet | <https://www.genome.jp/kegg-bin/show_organism?org=bmet> |
| *Bacillus sp.* X1(2014) | gst | <https://www.genome.jp/kegg-bin/show_organism?org=gst> |
| *Bacillus sp.* WP8 | bacw | <https://www.genome.jp/kegg-bin/show_organism?org=bacw> |
| *Bacillus sp.* Pc3 | bacp | <https://www.genome.jp/kegg-bin/show_organism?org=bacp> |
| *Bacillus sp.* BH072 | bacb | <https://www.genome.jp/kegg-bin/show_organism?org=bacb> |
| *Bacillus bombysepticus* | bby | <https://www.genome.jp/kegg-bin/show_organism?org=bby> |
| *Bacillus sp.* OxB-1 | baco | <https://www.genome.jp/kegg-bin/show_organism?org=baco> |
| *Bacillus sp*. YP1 | bacy | <https://www.genome.jp/kegg-bin/show_organism?org=bacy> |
| *Bacillus sp.* BS34A | bacl | <https://www.genome.jp/kegg-bin/show_organism?org=bacl> |
| *Bacillus sp.* LM 4-2 | balm | <https://www.genome.jp/kegg-bin/show_organism?org=balm> |
| *Bacillus endophyticus* | beo | <https://www.genome.jp/kegg-bin/show_organism?org=beo> |
| *Bacillus smithii* | bsm | <https://www.genome.jp/kegg-bin/show_organism?org=bsm> |
| *Bacillus simplex* | bsj | <https://www.genome.jp/kegg-bin/show_organism?org=bsj> |
| *Bacillus oceanisediminis* | bon | <https://www.genome.jp/kegg-bin/show_organism?org=bon> |
| *Bacillus glycinifermentans* | bgy | <https://www.genome.jp/kegg-bin/show_organism?org=bgy> |
| *Bacillus flexus* | bfx | <https://www.genome.jp/kegg-bin/show_organism?org=bfx> |
| *Bacillus gibsonii* | bgi | <https://www.genome.jp/kegg-bin/show_organism?org=bgi> |
| *Bacillus weihaiensis* | bwh | <https://www.genome.jp/kegg-bin/show_organism?org=bwh> |
| *Bacillus xiamenensis* | bxi | <https://www.genome.jp/kegg-bin/show_organism?org=bxi> |
| *Bacillus horikoshii* | bhk | <https://www.genome.jp/kegg-bin/show_organism?org=bhk> |
| *Bacillus krulwichiae* | bkw | <https://www.genome.jp/kegg-bin/show_organism?org=bkw> |
| *Bacillus beveridgei* | bbev | <https://www.genome.jp/kegg-bin/show_organism?org=bbev> |
| *Bacillus kochii* | bko | <https://www.genome.jp/kegg-bin/show_organism?org=bko> |
| *Bacillus altitudinis* | balt | <https://www.genome.jp/kegg-bin/show_organism?org=balt> |
| *Bacillus* sp. SDLI1 | bacs | <https://www.genome.jp/kegg-bin/show_organism?org=bacs> |
