# Supplementary material for: Comparative Analyses of Cytochrome P450s and Those Associated with Secondary Metabolism in Bacillus Species
Source: Int J Mol Sci. 2018 Nov 16;19(11):3623. doi: 10.3390/ijms19113623 (PMC6275058; doi:10.3390/ijms19113623)
Supplement: Supplementary file 1 [file ijms-19-03623-s001.zip › Supplementary files/Supplementary dataset 2.docx]

Comparative analyses of cytochrome P450s and those associated with secondary metabolism in *Bacillus* species

Bongumusa Comfort Mthethwa ^1^, Wanping Chen ^2^, Mathula Lancelot Ngwenya ^1^, Abidemi Paul Kappo ^1^, Puleng Rosinah Syed ^3^, Rajshekhar Karpoormath ^3^, Jae-Hyuk Yu ^4^, David R Nelson ^5^* and Khajamohiddin Syed ^1,^*

^1^ Department of Biochemistry and Microbiology, Faculty of Science and Agriculture, University of Zululand, KwaDlangezwa 3886, South Africa; 07bcomfort@gmail.com (B.C.M.); NgwenyaM@unizulu.ac.za (M.L.N.); KappoA@unizulu.ac.za (A.P.K.); khajamohiddinsyed@gmail.com (K.S.)

^2^ College of Food Science and Technology, Huazhong Agricultural University, Wuhan, Hubei Province, China; chenwanping@mail.hzau.edu.cn

^3^ Department of Pharmaceutical Chemistry, College of Health Sciences, University of KwaZulu-Natal, Durban 4000, South Africa; prosinah@gmail.com (P.R.S.); Karpoormath@ukzn.ac.za (R.K.)

^4^ Department of Bacteriology, University of Wisconsin-Madison, 3155 MSB, 1550 Linden Drive, Madison WI 53706, USA; jyu1@wisc.edu

^5^ Department of Microbiology, Immunology and Biochemistry, University of Tennessee Health Science Center, Memphis, TN, 38163; drnelson1@gmail.com

***** Correspondence: drnelson1@gmail.com (D.R.N.) and khajamohiddinsyed@gmail.com (K.S.)

**Supplementary dataset 2.** P450s identified in *Bacillus* species. Each P450 is presented with its assigned name, species name and amino acid sequence.

>CYP102A36 *Bacillus subtilis* subsp. *spizizenii* TU-B-10

MKETSPIPQPKTFGPLGNLPLIDKDKPTLSLIKLAEEQGPIFQIHTPAGTTIVVSGHELV

KEVCDEERFDKSIEGALEKVRAFSGDGLFTSWTHEPNWRKAHNILMPTFSQRAMKDYHEK

MVDIAVQLIQKWARLNPNEAVDVPGDMTRLTLDTIGLCGFNYRFNSYYRETPHPFINSMV

RALDEAMHQMQRLDVQDKLMVRTKRQFRYDIQTMFSLVDSIIAERRANGDQDEKDLLARM

LNVEDPETGEKLDDENIRFQIITFLIAGHETTSGLLSFATYFLLKHPDKLKKAYEEVDRV

LTDAAPTYKQVLELTYIRMILNESLRLWPTAPAFSLYPKEDTVIGGKFPITTNDRISVLI

PQLHRDRDAWGKDAEEFRPERFEHQDQVPHHAYKPFGNGQRACIGMQFALHEATLVLGMI

LKYFTLIDHENYELDIKQTLTLKPGDFHISVQSRHQEAIHADVQAAEKAAPDEQKEKTEA

KGASVIGLNNRPLLVLYGSDTGTAEGVARELADTASLHGVRTKTAPLNDRIGKLPKEGAV

VIVTSSYNGKPPSNAGQFVQWLQEIKPGELEGVHYAVFGCGDHNWASTYQYVPRFIDEQL

AEKGATRFSARGEGDVSGDFEGQLDEWKKSMWADAIKAFGLELNENADKERSTLSLQFVR

GLGESPLARSYEASHASIAENRELQSADSDRSTRHIEIALPPDVEYQEGDHLGVLPKNSQ

TNVSRILHRFGLKGTDQVTLSASGRSAGHLPLGRPVSLHDLLSYSVEVQEAATRAQIREL

ASFTVCPPHRRELEELSAEGVYQEQILKKRISMLDLLEKYEACDMPFERFLELLRPLKPR

YYSISSSPRVNPRQASITVGVVRGPAWSGRGEYRGVASNDLAERQAGDDVVMFIRTPESR

FQLPKDPETPIIMVGPGTGVAPFRGFLQARDVLKREGKTLGEAHLYFGCRNDRDFIYRDE

LERFEKDGIVTVHTAFSRKEGMPKTYVQHLMADQADTLISILDRGGRLYVCGDGSKMAPD

VEAALQKAYQAVHGTGEQEAQNWLRHLQDTGMYAKDVWAGI

>CYP102A48 *Bacillus subtilis* subsp. *spizizenii* TU-B-10

MKQASAIPQPKTYGPLKNLPHLEKEQLSQSLWRIADELGPIFRFDFPGVSSVFVSGHNLV

AEVCDEKRFDKNLGKGLQKVREFGGDGLFTSWTHEPNWQKAHRILLPSFSQKAMKGYHSM

MLDIATQLIQKWSRLNPNEEIDVADDMTRLTLDTIGLCGFNYRFNSFYRDSQHPFITSML

RALKEAMNQSKRLGLQDKMMVKTKLQFQKDIEVMNSLVDRMIAERKANPDENIKDLLSLM

LYAKDPVTGETLDDENIRYQIITFLIAGHETTSGLLSFAIYCLLTHPEKLKKAQEEADRV

LTDDTPEYKQIQQLKYIRMVLNETLRLYPTAPAFSLYAKEDTVLGGEYPISKGQPVTVLI

PKLHRDQNAWGPDAEDFRPERFEDPSSIPHHAYKPFGNGQRACIGMQFALQEATMVLGLV

LKHFELINHTGYELKIKEALTIKPDDFKITVKPRKTAAINVQRKEQADIKAETKPKETKP

KHGTPLLVLFGSNLGTAEGIAGELAAQGRQMGFTAETAPLDDYIGKLPEEGAVVIVTASY

NGAPPDNAAGFVEWLKELEEGQLKGVSYAVFGCGNRSWASTYQRIPRLIDDMMKAKGASR

LTAIGEGDAADDFESHRESWENRFWKETMDAFDINEIAQKEDRPSLSITFLSEATETPVA

KAYGAFEGIVLENRELQTAASTRSTRHIELEIPAGKTYKEGDHIGILPKNSRELVQRVLS

RFGLQSNHVIKVSGSAHMAHLPMDRPIKVVDLLSSYVELQEPASRLQLRELASYTVCPPH

QKELEQLVSDDGIYKEQVLAKRLTMLDFLEDYPACEMPFERFLALLPSLKPRYYSISSSP

KVHANIVSMTVGVVKASAWSGRGEYRGVASNYLAELNTGDAAACFIRTPQSGFQMPNDPE

TPMIMVGPGTGIAPFRGFIQARSVLKKEGSTLGEALLYFGCRRPDHDDLYREELDQAEQD

GLVTIRRCYSRVENEPKGYVQHLLKQDTQKLMTLIEKGAHIYVCGDGSQMAPDVERTLRL

AYEAEKAASQEESAVWLQKLQDQRRYVKDVWTGM

>CYP107H1 *Bacillus subtilis* subsp. *spizizenii* TU-B-10

MTIASSTASSEFLKNPYSFYDTLRAVHPIYKGSFLKYPGWYVTGYEETAAILKDARFKVR

TPLPESSTKYQDLSHVQNQMMLFQNQPDHRRLRTLASGAFTPRTTESYQPYIIETVHHLL

DQVQGKKKMEVISDFAFPLASFVIANIIGVPEEDREQLKEWAASLIQTIDFTRSRKALTE

GNIMAVQAMAYFKELIQKRKRHPQQDMISMLLKGREKDKLTEEEAASTCILLAIAGHETT

VNLISNSVLCLLQHPEQLLKLRENPDLIGTAVEECLRYESPTQMTARVASEDIDICGVTI

RQGEQVYLLLGAANRDPSIFTNPDVFDITRSPNPHLSFGHGHHVCLGSSLARLEAQIAIN

TLLQRMPSLNLADFEWRYRPLFGFRALEELPVTFE

>CYP107H3 *Bacillus subtilis* subsp. *spizizenii* TU-B-10

MTIAFSTASSAFLKDPYPFYETLRAVHPIYKGSFLKYPGWYVTGYEETAAILKDARFKVR

TPLPETSIKYQDLKHVQNQMMLFQNQPDHRRLRTLASGAFTPRMAESYRLYIDETVHHLL

DQVQGEKKMEVISDFAFPLASYVIANIIGVPAEDREQLKEWASILIQTIDFTRSRKALTE

GNHISVQAMAYFRELIQRRKRHPQQDMVSMLLKGKEKDKLTEEEVASTCVLLAIAGHETT

VNLISNSVLCLLQHPQQLLKLKENPDLIDTAVEECLRYESPTQMTARVASEDIDISGVTI

RKGEQVYLLLGAANRDPNIFMQADVFDITRSPNPHLSFGQGHHVCLGSSLARLEAQIAIH

TLLQRMPGLKLADSEWRYRPLFGFRALEELPVTFE

>CYP107J1 *Bacillus subtilis* subsp. *spizizenii* TU-B-10

MSSKEKKSVTILTESQLSSRAFKDEAYEFYKELRKSQALYPLSLGALGKGWLISRYDDAI

HLLKNEKLKKNYENVFTAKEKRPALLKNEETLTKHMLNSDPPDHNRLRTLVQKAFTHRMI

LQLEDKIQHIADSLLDKVQPNKFMNLVDDYAFPLPIIVISEMLGIPLEDRQKFRVWSQAI

IDFSDAPERLQENDHLLGEFVEYLESLVRKKRREPAGDLISALIQAESEGTQLSTEELYS

MIMLLIVAGHETTVNLITNMTYALMCHHDQLEKLRQQPDLMNSAIEEALRFHSPVELTTI

RWTAEPFILHGQEIKRKDVIIISLASANRDEKIFPNADIFDIERKNNRHIAFGHGNHFCL

GAQLARLEAKIAISTLLRRCPNIQLKGEKKQMKWKGNFLMRALEELPISF

>CYP107J5 *Bacillus subtilis* subsp. *spizizenii* TU-B-10

MSSKQKKSLTILTESQLCSSAFKDEAYDFYKESRKSHPLYPLSLGKLGEGWLISRYDDAI

HLLKNEKLKKNYENVITAKEEKRPVLLKNEEPLTKHMLNSDPPDHNRLRTLVQKAFTHRM

ILQLEDKIQHIADSLLDKVQPNKFMNLVDDYAFPLPIIVISEMLGIPLEDRQRFRVWSQA

IIDFSDAPERLQENDHLLGEFVEYLEYLVRIKRSEPAGDLISVLIQVESEGTQLSTEELY

SMIMLLIVAGHETTVNLITNMTYALMCHHDQLEKLRQQPGLINSAIEEALRFHSPVELTT

IRWTAEPFILHGQEIKRKDVIIISLASANRDEKIFSNADIFDIERKNNRHIAFGHGSHFC

LGAQLARLESKIAISTLLRRCPNIQIKGEKEQIKWKGNFLMRALEELPLSF

>CYP107K1 *Bacillus subtilis* subsp. *spizizenii* TU-B-10

MEKLMFHPHGKEFHHNPFSVLGRFREEEPIHRFELKRFGATYPAWLITRYDDCMAFLKDN

RITRDVKNVMNQEQIKMLNVSEDIDFVSDHMLAKDTPDHTRLRSLVHQAFTPRTIENLRG

SIEQIAEQLLDEMEKENKADIMKSFASPLPFIVISELMGIPKEDRSQFQIWTNAMVDTSE

GNRELTNQALREFKDYIAKLIHDRRIKPKDDLISKLVHAEENGSKLSEKELYSMLFLLVV

AGLETTVNLLGSGTLALLQHKKECEKLKQQPEMIATAVEELLRYTSPVVMMANRWAIEDF

TYKGHSIKRGDMIFIGIGSANRDPNFFENPEILNINRSPNRHISFGFGIHFCLGAPLARL

EGHIAFKALLKRFPDIELAVAPDDIQWRKNVFLRGLESLPVSLSK

>CYP109A1 *Bacillus subtilis* subsp. *spizizenii* TU-B-10

MTNQTARPSKEERYANLIPMEELHSEKDRLFPFPIYDKLRRESPVRYDQQRDCWDVFKYD

DVQFVLKNPKFFSSKRGIQTESILTMDPPKHTKLRALVNRAFTPKAVKQLETRIKDVTAF

LLQEARQKSTIDIIEDFAGPLPVIIIAEMLGAPIEDRHLIKTYSDVLVAGAKDSSDKAVA

DMLHNRRDGHAFLSDYFRDILSKRRTEPKEDLMTMLLQAEIDGEHLTEEQLIGFCILLLV

AGNETTTNLIANAVRYLTEDSVVQQQVRQNTDNIANVIEETLRYYPPVQAIGRVAAEDTE

LGGVFIKKGSSVISWIASANRDEDKFCEPDCFKIDRPSYPHLSFGFGIHFCLGAPLARLE

ANIALSSLLSMSACIEKADHDEKLEAIPSPFVFGVKRLPVRITFK

>CYP109B1 *Bacillus subtilis* subsp. *spizizenii* TU-B-10

MNVLNRRQALQRALLNGKNKQDAYHPFPWYESMRKDAPVSFDEENQVWSVFLYDDVKKVV

GDKELFSSCMPQQTSSIGNSIINMDPPKHTKIRSVVNKAFTPRVMKQWEPRIQEITDELI

QKFQGRSEFDLVHDFSYPLPVIVISELLGVPSAHMEQFKAWSDLLVSTPKDKSEEAEKAF

LEERDKCEEELAAFFAGIIEEKRNKPEQDIISILVEAEETGEKLSGEELIPFCTLLLVAG

NETTTNLISNAMYSILETPGVYEELRSHPELMPQAVEEALRFRAPAPVLRRIAKRDTEIG

GHLIKEGDMVLAFVASANRDEAKFDRPHMFDIRRHPNPHIAFGHGIHFCLGAPLARLEAN

IALTSLISAFPHMECVSITPIENSVIYGLKSFRVKM

>CYP134A1 Bacillus subtilis subsp. spizizenii TU-B-10

MSQSIKLFSVLSDQFQNNPYAYFSQLREEDPVHYEESIDSYFISRYHDVRYILQHPDIFT

TKSLVERAEPVMRGPVLAQMHGKEHSAKRRIVVRSFIGDALDHLSPLIKQNAENLLAPYL

ERGKSDLVNDFGKTFAVCVTMDMLGLDKRDHEKISEWHSGVADFITSISQSPEARAHSLW

CSEQLSQYLMPVIKERRVNPGSDLISILCTSEYEGMALSDKDILALILNVLLAATEPADK

TLALMIYHLLNNPEQMNDVLADRSLVPRAIAETLRYKPPVQLIPRQLSQDTVVGGMEIKK

DTIVFCMIGAANRDPEAFEQPDVFNIHREDLGIKSAFSGAARHLAFGSGIHNCVGAAFAK

NEIEIVANIVLDKMRNIRLEEDFCYAESGLYTRGPVSLLVAFDGA

>CYP152A1 *Bacillus subtilis* subsp. *spizizenii* TU-B-10

MNEQIPHDKSLDNSLTLMQEGYLFIKNRTERYHSDLFQARLLGKTFICMTGAEAAKLFYD

TERFQRQNALPKRVQKTLFGVNAIQTMDGDAHIHRKLLFLSLMTPPHQKRLAELMTEEWE

AAVTRWEKADQVVLFEEAKEILCRVACYWAGVPLKETEVKERADDFIDMVDAFGAVGPRH

WKGRRARPRAEEWIERMIEDVRAGLLKIPAGTALHEMAFHTQEDGNQLDSRMAAIELINV

LRPIVAISYFLVFSALALHKHPKYKEWLRSGSNREREMFVQEVRRYYPFGPFLGALVKKD

FEWNTCEFKKGTSVLLDVYGTNHDPRLWDNPDEFRPERFEEREENPFDLIPQGGGHAEKG

HRCPGEGITIEVMKASLDFLVNQIEYDVPEQSLHYSLARMPSLPESGFVMSGIRRKS

>CYP102A5 *Bacillus thuringiensis* YWC2-8

MEKKVSAIPQPKTYGPLGNLPLIDKDKPTLSFIKIAEEYGPIFQIQTLSDTIIVVSGHEL

VAEVCDETRFDKSIEGALAKVRAFAGDGLFTSETHEPNWKKAHNILMPTFSQRAMKDYHA

MMVDIAVQLVQKWARLNPNENVDVPEDMTRLTLDTIGLCGFNYRFNSFYRETPHPFITSM

TRALDEAMHQLQRLDIEDKLMWRTKRQFQHDIQSMFSLVDNIIAERKSSGDQEENDLLSR

MLNVQDPETGEKLDDENIRFQIITFLIAGHETTSGLLSFAIYFLLKNPDKLKKAYEEVDR

VLTDPTPTYQQVMKLKYIRMILNESLRLWPTAPAFSLYAKEDTVIGGKYPIKKGEDRISV

LIPQLHRDKDAWGDNVEEFQPERFEELDKVPHHAYKPFGNGQRACIGMQFALHEATLVMG

MLLQHFELIDYQNYQLDVKQTLTLKPGDFKIRILPRKQTISHPTALAPTEDKLKNDEIKQ

HVQKTPSIIGADNLSLLVLYGSDTGVAEGIARELADTASLEGVRTEVVALNDRIGSLPKE

GAVLIVTSSYNGKPPSNAGQFVQWLEELKTDELKGVQYAVFGCGDHNWASTYQRIPRYID

EQMAQKGATRFSKRGEADASGDFEEQLEQWKQSMWSDAMKAFGLELNKNMEKERSTLSLQ

FVSRLGGSPLARTYEAVYASILENRELQSSSSDRSTRHIEVSLPEGATYKEGDHLGVLPV

NSEKNINRILRRFGLNGKDQVILSASGRSINHIPLDSPVSLLDLLSYSVEVQEAATRAQI

REMVTFTACPPHKKELEALLEEGVYHEQILKKRISMLDLLEKYEACEIRFERFLELLPAL

KPRYYSISSSPLVVQNRLSITVGVVNAPAWSGEGTYEGVASNYLAQRHNKDEIICFIRTP

QSNFELPKDPETPIIMVGPGTGIAPFRGFLQARRVQKQKGINLGEAHLYFGCRHPEKDYL

YRTELENDERDGLISLHTAFSRLEGHPKTYVQHLIKQDRINLISLLDNGAHLYICGDGSK

MAPDVEDTLCQAYQEIHEVSEQEARNWLDRVQDEGRYGKDVWAGI

>CYP102A8 *Bacillus thuringiensis* YWC2-8

MDKKVSAIPQPKTYGPLGNLPLIDKDKPTLSFIKLAEEYGPIFQIQTLSDTIIVVSGHEL

VAEVCDETRFDKSIEGALAKVRAFAGDGLFTSETDEPNWKKAHNILMPTFSQRAMKDYHA

MMVDIAVQLVQKWARLNPNENVDVPEDMTRLTLDTIGLCGFNYRFNSFYRETPHPFITSM

TRALDEAMHQLQRLDIEDKLMWRTKRQFQHDIQSMFSLVDNIIAERKSSENQEENDLLSR

MLNVQDPETGEKLDDENIRFQIITFLIAGHETTSGLLSFAIYFLLKNPDKLKKAYEEVDR

VLTDSTPTYQQVMKLKYIRMILNESLRLWPTAPAFSLYAKEDTVIGGKYPIKKGEDRISV

LIPQLHRDKDAWGDDVEEFQPERFEELDKVPHHAYKPFGNGQRACIGMQFALHEATLVMG

MLLQHFEFIDYEDYQLDVKQTLTLKPGDFKIRIVPRNQTISHTTVLAPTEEKLKKHEIKK

QVQKTPSIIGADNLSLLVLYGSDTGVAEGIARELADTASLEGVQTEVVALNDRIGSLPKE

GAVLIVTSSYNGKPPSNAGQFVQWLEELKPDELKGVQYAVFGCGDHNWASTYQRIPRYID

EQMAQKGATRFSTRGEADASGDFEEQLEQWKQSMWSDAMKAFGLELNKNMEKERSTLSLQ

FVSRLGGSPLARTYEAVYASILENRELQSSSSERSTRHIEISLPEGATYKEGDHLGVLPI

NNEKNVNRILKRFGLNGKDQVILSASGRSVNHIPLDSPVRLYDLLSYSVEVQEAATRAQI

REMVTFTACPPHKKELESLLEDGVYQEQILKKRISMLDLLEKYEACEIRFERFLELLPAL

KPRYYSISSSPLVAQDRLSITVGVVNAPAWSGEGTYEGVASNYLAQRHNKDEIICFIRTP

QSNFQLPENPETPIIMVGPGTGIAPFRGFLQARRVQKQKGMKVGEAHLYFGCRHPEKDYL

YRTELENDERDGLISLHTAFSRLEGHPKTYVQHVIKEDRIHLISLLDNGAHLYICGDGSK

MAPDVEDTLCQAYQEIHEVSEQEARNWLDRLQEEGRYGKDVWAGI

>CYP106B1 *Bacillus thuringiensis* YWC2-8

MASPENVILVHEISKLKTKEELWNPYEWYQFMRDNHPVHYDDEQDVWNVFLYDDVNRVLS

DYRLFSSRRERRQFAIPPLETRININSTDPPEHRNVRSIVSKAFTPRSLEQWKPRIQSIA

NELVKDIENCSEVDIVEQFAAPLPVTVISDLLGVPTTDRKKIKAWSDILFMPYSKEKFND

LDAEKGIALNEFKAYLLPIVQEKRYHLTDDIISDLIRAEYEGERLTDEEIVTFSLGLLAA

GNETTTNLIINSFYCFLVDSPATYKEVREKPKLISKAVEEVLRYRFPVTLARRITEDTNI

FGPLMKKDQMVVAWVSAANLDEKKFSQASKFNIHRIGNEKHLTFGKGPHFCLGAPLARLE

AEIALTTFINAFEKIALSPSFNIEQCILENEQTLKFLPIRLKAQ

>CYP106B4 *Bacillus thuringiensis* YWC2-8

MASPENVILVHEISKLKTKEELWNPYEWYRLMRDNHPVHYDEEQDVWNVFLYDDVNRVLS

DYRLFSSRRERRQFSIPPLETRININSTDPPEHRNVRSIVSKAFTPRSLEQWKPRIQAIA

DELVQHIEKCSEVNIVEQFAAPLPVTVISDLLGVPTTDRKKIKEWSDILFMPYSKEKFND

LDAEKGIALNEFKAYLLPIVQEKRYHLTDDIISDLIRAEYEDERLTDEEIVTFSLGLLAA

GNETTTNLIINSFYCFLVDSPGIYEELRKEPNLISKAIEEVLRYRFPVTLARRITEDTNI

FGSLMKKDQMIVAWVSAANLDEKKFSQASQFNVHRTGNEKHLTFGKGPHFCLGAPLARLE

AEIALTTFINAFEKIELSPSFCLEKCILENEQTLKYLPIRLKAK

>CYP107J2 *Bacillus thuringiensis* YWC2-8

MAMKNKVGLRIEDGINLASAQFKEDAYEIYKESRKVQPVLFVNKTELGAEWLITRYEDAL

PLLKDSRLKKDPANVFSQDTLNVFLTVDNGDHLTTHMLNSDPPNHNRLRSLVQKVFTPKM

IAQLEGRIQDITDDLLNEVERKGSLNLVDDYSFPLPIIVISEMLGIPKEDQAKFRIWSHA

VIAYPETPEEIKETEKQLSEFITYLQYLVDMKRKEPKEDLVSALILAESEGHKLSARELY

SMIMLLIVAGHETTVNLITNTVLALLENPNQLQLLKENPKLIDAAIEEGLRYYSPVEVTT

SRWADEPFQIHDQTIEKGDMVVIALASANRDETVFENPEVFDITRENNRHIAFGHGSHFC

LGAPLARLEAKIAITTLFERMPELQIKGNREDIKWQGNYLMRSLEELPLTF

>CYP107J3 *Bacillus thuringiensis* YWC2-8

MSMKNKVGLSIEDGINLASAQFKEDAYEIYKESRKKQPILFVNQVEIGKEWLITRYEDAL

PLLKDNRLKKDWTNVFSQDIKNMYLSVDNSDHLTTHMLNSDPPNHSRLRSLVQKAFTPKM

IAQLDKRIEKIADDLISDIERKGTLNLVDDYSFPLPIIVISEMLGIPKEDQAKFRIWSHA

VIASPETPEEIKETEKQLSEFITYLQYLVDIKRKEPKEDLVSALILAESEGHKLSARELY

SMIMLLIVAGHETTVNLITNTVLALLENPNQLQLLKDNPKLIDSAIEEGLRYYSPVEVTT

ARWAAEPFQIHDRTIEKGDMVVIALASANRDETVFENPEVFDITRENNRHIAFGHGSHFC

LGAPLARLEAKIAITTLFNRMPELQIKGNREEIKWQGNYLMRSLEELPLTF

>CYP107K3 *Bacillus vallismortis*

MEKTMFHPHSPEFHENPFAVLSRFREKDPIHKFELQRFGGTFPAWLITRYDDCMAFLKDG

RITRDVKRVMPKELIAKLNVSEDIDFVSEHMLAKDPPDHSRLRSLVHQGFTPRMIEQLRT

GIEQITEELLDKMETKADPDIMRDFAAPLPFIVISELLGIPKEDRAKFQVWTNAMVDTSE

SGQDATNQALKEFKQYMKTLIEEKRKHPGEDLTSKLIYAEEDGQKLSESELYSMLFLLVV

AGLETTVNLLGSGTLALLLHKDQMEKIKRQPENIQMAVEELLRYTSPVIMMANRWAIEDF

TYKDVSIKKGEMIFIGIGSANRDPEYFDDPDTLNIARTPNRHISFGFGIHFCLGAPLARM

EASIAFTALLKRFPNIELKGAPEDVTWRKNVFLRGLETLPVRF

>CYP107H4 *Bacillus vallismortis*

MTAGLSIAHPSLHADSDFWNDPYPFYDKLRSIDPVYKGTVLKYPGWYVTGYKEAAAILKD

TRFKNRIPLPEASTKYQNLSHIQHDMLLFKNQSDHKRMRMLIGKEFTAKTAESLRPCINE

TVHDLLDQVQIKKNADLVSEFAFPLASLIIAEILGVPKEERYQFRQWTADVIQAIDLTRS

RKTLVRASDTAGRLTSYFRDLIHKRKAHPQQDLISRFIMEEQLSKEEVLATCILLVIAGH

ETTVNLISNGVFTLLKHPEQLSALRENPSLIETAVEECLRYDSPAQLTARTASEDCEING

KTIKKGEQVYILLGAANRDPSIFDQPHKMDIQRKPNPHLAFGKNAHFCIGSSLARIEAQI

AILTLFERMPKLRLAAHRLEYRKLIGFRSLKELPVVIG

>CYP113L1 *Bacillus vallismortis*

MTSLTKIRQQQPYKWYQTMRETSPVHYNEKEDCWEIFTYDEVKRVISDYSHFSSDHKYLS

ADKQEKMIRHINKDSLLKMDPPEHTVFRKLVNQPFMPKSVETLAPRIAAIADDLLQAVRS

KGRMDIIEDYAFPLPIIVIAELLGFPPKDRDIFKSWVDQSQNVKDEKKMNEVQKQMIGYF

MQFILQRRKQPQNDLISHLISADLDGEPLSDKQLIGFCGLLIVAGHVTTENVIGNSFLSL

KEFPHILPRLLENKALLPDFIEEVIRLRPSIQRVTRYTAVESEIGGKTIPAGEKVYAWIG

SANRDEKKFENPDQIDLGRKPNQHLSFGQGSHYCLGAPLARLEAKIALSHFFEQMPAWRF

TKDQEPNLVPSPVFHGVDRLLVEF

>CYP152A4(B9C48_05560)*Bacillus vallismortis*

MDEQIPQEKGLDNSLALLRDGYVFVKNRAENYRSDVFRARLLGKTFICMSGAEAAKLFYD

TERFQRQQALPKRVQKTLFGTGAIQAMDGERHKHRKLLFMSLMTPPRQKRLAEAVEKQWK

ASAEMWKGSNRIVLFDEAKKVLCRAACEWAGVPLKDSEVKERAEDFTDMVDAFGAVGPRH

WKGRRARLKTEKWIEEVIEDVRSGKLQTPEGSALYEMAFHTELDGNRLNSHMAAVELINV

LRPIAAISYFITFSALALHDYPEYRGKLRSRDDQEAERFAHEVRRYYPFAPFLGAVVKKD

FVWKNCEFKKGASVMLDLYGTNHDSRLWENPNEFRPERFQGREENKFDFIPQGGGDPANG

HRCPGEGMTVEVMKTSLAFLANEIEYDVPSQDLSFSLSRMPALPESGFVISDVRRI

>CYP109B7(B9C48_06155)*Bacillus vallismortis*

MSKLRSTRQAIQRTLMKGKNGLDVYNPFPWYEKMRRESPIHFDEETKVWSVFLYDDVKKV

ISDKETFSSLMTDVKSSIAKSMLNMDPPKHTQIRSAVNRAFTPRVLKEWEPRIKDITDHL

LKQAKNKGRIDIVKDLSYPLPVMVISELLGVPSERMDQFKKWSDILVSMPKDASPEAAEK

NQQERDQCEAELAAFFAEIIESKRKKPGQDIISILIKEEEEGEKLTAEDLIPFCNLLLVA

GNETTTNLISNAVYSILETPGLYEELRQDPSLIAQTVEETLRFRAPAPFVRRTVRHDTEL

RGRRLKSGEIVLCYVASANRDENKFEKAGVFDIHRQSNPHLSFGFGVHFCLGAPLARLEA

EVALKGIVKAFSHLEPVRIEPIRNSVMYGLESLEAEINENEGEEKR

>CYP102A49(B9C48_12405)*Bacillus vallismortis*

MKQLSAIPQPKTYGPLKNLPHLEKEKLSQSLWKIAEEYGPIFRFEFPSSVGVFVSGRELA

AEVCDEKRFDKNLSKALLKVREFGGDGLFTSWTHEKNWQKAHRILLPSFSQKAMKGYHSM

MLDIAMQLVQKWSRLNPNEEIDVAEDMTRLTLDTIGLCGFHYRFNSFYRDTQHPFITSML

RALQEAMRQSQRHSLQDKLMIKTRHQFQQDIEEMNSLVDRIIAERRENPDENLSDLLALM

LEAKDPVTGERLDDENIRYQIITFLIAGHETTSGLLSFAIYCLLKNKDKLKKAVQEAERV

LTGETPEYKQIQQLTYIRMVLNETLRLYPTAPAFSLYAKEDTVLGGKYPIAKGQPVTILT

PQLHRDKSAWGEDAELFRPERFSDPAAIPADAYKPFGNGQRACIGMQFALQEATMVLGLV

LKHFELIDHTDYELTIKEALTIKPGDFKIRVKNKDVSNHQPVQNHKAEGSGHKEETKEIP

SHGTPLLILYGSNLGTAEGIAEELADIGRSKGFSTETGPLDDYAGKLPVKGAVVIVTASY

NGAPPDNAAGFVKWMETLEDQELKGVSYAVFGCGDRNWAATYQRIPRLIDKVLEEKGAKR

LTSIGEGDNADDFEYSQEAWEDSFWKDIMKAFHIEAAPKQTNKSQLSIEYVSEAAETPIA

KTYKAFEAEVITNKELHTESSKRSVRHIELRLPETETYQEGDHLGVLPQNSGELISRVIR

RFGLDPNQHFKIKGRQLPHLPMDRPVNAPELLASYVELQEPATRAQLRELAAHTVCPPHQ

KELEHLYSDDTAYKENVLKKRMTMLDLLEDYPACELPFERFLELLPSLKARYYSISSSPK

AASGELSITVGVVTAPAWSGRGEYRGVASNYLAGLQNGDSAVCFIRSPQSGFALPENPKT

PLIMVGAGTGIAPFRGFIQARAAEKMSGNILGEAHLYFGCRHPEQDDLYKDEFDHAEKNG

LVTVHRAYSRLDQDCKVYVQDVLLREAEQIIALLDQGGHLYICGDGSKMAPAVENVLLQA

YEKVHNTDSKVSLEWLEQLQAEGRYAKDVWAGM

>CYP102A43 *Bacillus mycoides* 219298

MREEQTSNIPQPKTYGPLGNYPLFDMSQPTISFCEMAKEYGPIFRLTAPGFKTICISEHK

LVTEVCDINRFDKHVANDMVHVRKFTGDGLFTSKTSEPNWRKAHNILLPTFSQQAMKGYH

TMMLDIASQLIQKWARLNHDSDHIDVPGDMTRLTLDTIGLCGFNYRFNSFYKEKHDPFID

SMVRALDEAMHKIARSKGLDTLMIRKNRQFQDDIQSMFSLVDKIIKERKANGNNGEIDLL

ARMLNSKDPETGEVLDDENIRYQIITFLIAGHETTSGLLSFTTYFLLKHPEVLKKAYEEV

DQVLGDSTPSYKQVLNLKYIRMILNEAIRLWPTAPSFDVYPKEDTIIGGQYHVKKGEGLT

VLLPALHRDKEVWGEDAEEFRPERFADPSKVPNHAYKPFGNGQRACIGMQFALYEATLVL

GMILQKLELIDSENYQLKIKQSLTIKPENFRIRVKLREGKEVAPLLNLPLDDEKENEEKK

KVTSQYSKQEVLEGAENVPLLVLYGSNLGRAEEFARKLADHARLLGFQSDVRTLNDYIGK

LPKEGAVLIVTSSYNGQPPENAAQFVKWLEQASAEECTGIRYAVFGCGDRNWANTYQSVP

KFIDKELELKGAIRYTERGEADAGGDFENDFAQWQKNMWKDVSNVFCLKMKEGTETVSHQ

SSLSVQLISGPHANPIVQKNEAVYATVIASRELQSSESERSTRHIEIALPEGVTYQVGDH

LGVLPSNSRKNVNRILKRFRLNEKDQVILSTSGSSTAHLPLDRPVSLFDLLSHSVEIQEA

ATRAQIRELARFTVCPPHKYELEALLEDGVYQDQILKKHISMLDLLEKYEACEIPFERFL

ELLPALKPRYYSISSSPLVAQDRLSITVGVVSGPAWSGQGEYKGVASNYLAQRHNEDEIT

CFIRTPESRFQLPEDPEKPIIMVGPGTGVAPFRGFLQTRRVQKQKGVNLGEAHLYFGCRH

PEKDYLYRTELENDEKDGLISLHTAFSRSEGHPKTYVQHLMKQDGANLISLLDNGAHLYI

CGDGSRMAPDVEDTLCHAYQEMHGVSEQEARNWLDQLQHEGRYGKDVWAGI

>CYP106B1 *Bacillus mycoides* 219298

MPTTDRKKIKEWSDILFMPYSKEKFNDLDVEKGIALNEFKTYLLPIVQEKRYHLTDDIIS

DLIRAEYEGERLTDEEIVTFSLGLLAAGNETTTNLIINSFYCFLVDSPGIYKELRKEPTL

ISKAIEEVLRYRFPITLARRITADTNIFGPFMKKDQMIVTWVSAANSDEKKFLQASKFNL

HRIGNEKHLTFGKGPHFCLGAPLARLEAEIALTTFINAFEKIELSPSFNLEKCILENEQT

LKYLPILLKTR

>CYP106B6 *Bacillus mycoides* 219298

MNSPENVILVHEISKLKTKEELWNPYEWYKHMRENHPVYYDAEQDVWNVFLYNDVNRVLS

DYRLFSSRRDRRQFSVPPLDTRVNLNSSDPPEHRNVRSIVSKAFTPRSLQQWKPQIQAIA

NELVKDMNNYDEIDIVEQFAAILPVTVISDLLGVPTTDRKKIKMWSDILFMPYSKEKFSD

LDKQKEVALHEFKSYLLPIVQEKRYHLKEDIISDLIRAEYEGERLTDEEIVTFSLGLLAA

GNETTTNLIINSFYCFLVDAPKVYKELSESPELIPKAIEEVLRYRFPVTLARTITEDTTI

FGPKMKKGQMIVTWISAANLDENKFSQADCFDLHRPGNEKHLTFGKGPHFCLGAPLARLE

AEVALTTFIRNFKKLELSPSFHLENCILENEQTLKRFPILLKK

>CYP107J3 *Bacillus mycoides* 219298

MSIKNKVGRKIEDGINLASAQFKEDAYEIYKESRKMQPILFVNEVEIGKEWLITRYEDAL

PLLKDNRLKKDMANVFSQDTKNMYLSVDNSDHLTTHMLNSDPPNHSRLRSLVQKAFTPKM

ISQLDGRIQRVADDLINEIERKGTLNLVDDYSFPLPIIVISEMLGIPKEDQAKFRIWSHA

VIASPETPEEIKETEKQLSEFITYLQYIVDVKRKNPKEDLVSALILAENEGHKLSARELY

SMIMLLIVAGHETTVNLITNTVLALLENPKQLQLLKENPELIDSAIEEGLRYYSPVEVTT

ARWAAEPFQIHDQTIQKGDMVIIALASANRDETVFENPEVFDITRENNRHIAFGHGSHFC

LGAPLARLEAKIAITTLFKRMPSLQIKGEREKINWQGNYLMRSLEELPLSF

>CYP107J7 *Bacillus mycoides* 219298

MFFKNKVGLKIEDGIQLASAGFKEDAYEIYKESRASQPILFVYKSELWTEWLITRYEDAL

PLLKDSRLKKNPENVFSQERLKPFLSLENSDRLTKHMLNSDPPDHSRLRALVQKAFTPKM

ISQLDERIQNIADTLLDRVEYKHSLNLVNDYAFPLPINVISEMLGVPKDDQERFRIWSHA

VIASPETPEEIKENEEKLSEFITYLQYLVDVKRKEPKEDLVSGLIQVESEGSKLSAPELY

SMIMLLIVAGHETTVNLITNTVLALLENPDQLQLLKEKPQLIDSAIEEGLRYYSPVEITT

ARWATEPFMIHDQEIQKGDMVIISLASANRDENVFVNPDVFDITRENNRHIAFGHGSHFC

LGAPLARLEAKIAIATLLKRIPKIQIKGEREKIKWQGNYLMRSLEELPLTF

>CYP107J11 *Bacillus mycoides* 219298

MISKNELNFSLLAETQLASPMFKQKAYDIYKELRAFQPVYPLSLCEQGQGWLITRYEDAM

ALLKDARLMKNFENVFSKKEDVKVPFSLENRELLRNHMLNSDPPDHHRLRSLVQKAFTPQ

MILQLECRIQHIADALLNKVEHTHSINLVNDYAFPLPIMVISEMLGIPLEDQHKFRIWSQ

AIIDTPLRLEDVQKNNQKLEEFAEYIQYLVYKKRKNPTDDLISALIQAESEGIKLNASEL

YSTITLLIVAGHETTVNLIANMTLALLEHPVQLQKLLQNPDLIDSAIEEALRFYSPVELT

TIRWTAEPFTMHGQDIQSKDRIIISLASSNRDEKIFSNADVFDVTRKNNRHIAFGYGSHF

CLGASLARLESKIAISTLLRRMPNLQIQGEREQVKWKESYLMRSLEELPLQL

>CYP107DY2 *Bacillus mycoides* 219298

MKKITLGDLNSPETMRDPIVYYKPFFEQQEPLFRLDDFYGMGGAWIALRYDDVVTILKDP

RFLKDLRKFEPPQDKQEFIEENTSVSKLFEWMMNMPNMLTVDPPDHTRLRRLVSKAFTPR

IIENLHPRIQQIADELLDAVKEKGKMDIIADFAYPLPIIVISEMLGIPVTDRNQFRDWTH

KLMTAAMDPRQGAAVTATLEKFIHYIETLLAEKRVNPSDDLMSALVQAQEKEDKLSKNEL

LSTIWLLIIAGHETTVNLIGNGVLALLQHPEQMDLLRDNPSMLSSAVDELLRYAGPIMIG

NRFAGEDITIHGKIIHKGEMVLFSLAAANIDPQKFSNPEALDITREENDHLAFGNGIHRC

LGAPLARLEGQIAFGTLLQRLPNLRLATKPEQLVYNYSKFRSLATLPVVF

>CYP1341C1 *Bacillus mycoides* 219298

MLQKIELKYDPLDPGTLLNPYPIYKKLRENAPVYWHEGMKSWVLTRYDDCKEVLRNHEVF

TTDRRRVGVEIPDVRHNVQSLDPPDNIPLRNLLTRAFNSQDINNVREKIHVLIKDIFKKH

KSINEFDFMREVSAPLALSMTAITLGVDEPNLDSFLEISEAITRQMDSGLRPENIEPGNQ

AREKLNALVGEWFAAEDRPGIVSYIRKHAQNTNVPEHYIRNTTGTMFNASFGSLYAVFGN

VVLALLEHPEVFDKLNDKSLIDTGVDELIRFDGPAQGTGRIAAKTTKIRDTTIQKGDVIV

VLFAAANRDPEVFPEPDSIILDRSKNPHLGFGWGPHTCVGTFFGKLAIKELILCLLEESS

RLRLLRRPTRRVTATSRGIELLPVSFS

>CYP102A36 *Bacillus subtilis* QB928

MKETSPIPQPKTFGPLGNLPLIDKDKPTLSLIKLAEEQGPIFQIHTPAGTTIVVSGHELV

KEVCDEERFDKSIEGALEKVRAFSGDGLFTSWTHEPNWRKAHNILMPTFSQRAMKDYHEK

MVDIAVQLIQKWARLNPNEAVDVPGDMTRLTLDTIGLCGFNYRFNSYYRETPHPFINSMV

RALDEAMHQMQRLDVQDKLMVRTKRQFRYDIQTMFSLVDSIIAERRANGDQDEKDLLARM

LNVEDPETGEKLDDENIRFQIITFLIAGHETTSGLLSFATYFLLKHPDKLKKAYEEVDRV

LTDAAPTYKQVLELTYIRMILNESLRLWPTAPAFSLYPKEDTVIGGKFPITTNDRISVLI

PQLHRDRDAWGKDAEEFRPERFEHQDQVPHHAYKPFGNGQRACIGMQFALHEATLVLGMI

LKYFTLIDHENYELDIKQTLTLKPGDFHISVQSRHQEAIHADVQAAEKAAPDEQKEKTEA

KGASVIGLNNRPLLVLYGSDTGTAEGVARELADTASLHGVRTKTAPLNDRIGKLPKEGAV

VIVTSSYNGKPPSNAGQFVQWLQEIKPGELEGVHYAVFGCGDHNWASTYQYVPRFIDEQL

AEKGATRFSARGEGDVSGDFEGQLDEWKKSMWADAIKAFGLELNENADKERSTLSLQFVR

GLGESPLARSYEASHASIAENRELQSADSDRSTRHIEIALPPDVEYQEGDHLGVLPKNSQ

TNVSRILHRFGLKGTDQVTLSASGRSAGHLPLGRPVSLHDLLSYSVEVQEAATRAQIREL

ASFTVCPPHRRELEELSAEGVYQEQILKKRISMLDLLEKYEACDMPFERFLELLRPLKPR

YYSISSSPRVNPRQASITVGVVRGPAWSGRGEYRGVASNDLAERQAGDDVVMFIRTPESR

FQLPKDPETPIIMVGPGTGVAPFRGFLQARDVLKREGKTLGEAHLYFGCRNDRDFIYRDE

LERFEKDGIVTVHTAFSRKEGMPKTYVQHLMADQADTLISILDRGGRLYVCGDGSKMAPD

VEAALQKAYQAVHGTGEQEAQNWLRHLQDTGMYAKDVWAGI

>CYP102A48 *Bacillus subtilis* QB928

MKQASAIPQPKTYGPLKNLPHLEKEQLSQSLWRIADELGPIFRFDFPGVSSVFVSGHNLV

AEVCDEKRFDKNLGKGLQKVREFGGDGLFTSWTHEPNWQKAHRILLPSFSQKAMKGYHSM

MLDIATQLIQKWSRLNPNEEIDVADDMTRLTLDTIGLCGFNYRFNSFYRDSQHPFITSML

RALKEAMNQSKRLGLQDKMMVKTKLQFQKDIEVMNSLVDRMIAERKANPDENIKDLLSLM

LYAKDPVTGETLDDENIRYQIITFLIAGHETTSGLLSFAIYCLLTHPEKLKKAQEEADRV

LTDDTPEYKQIQQLKYIRMVLNETLRLYPTAPAFSLYAKEDTVLGGEYPISKGQPVTVLI

PKLHRDQNAWGPDAEDFRPERFEDPSSIPHHAYKPFGNGQRACIGMQFALQEATMVLGLV

LKHFELINHTGYELKIKEALTIKPDDFKITVKPRKTAAINVQRKEQADIKAETKPKETKP

KHGTPLLVLFGSNLGTAEGIAGELAAQGRQMGFTAETAPLDDYIGKLPEEGAVVIVTASY

NGAPPDNAAGFVEWLKELEEGQLKGVSYAVFGCGNRSWASTYQRIPRLIDDMMKAKGASR

LTAIGEGDAADDFESHRESWENRFWKETMDAFDINEIAQKEDRPSLSITFLSEATETPVA

KAYGAFEGIVLENRELQTAASTRSTRHIELEIPAGKTYKEGDHIGILPKNSRELVQRVLS

RFGLQSNHVIKVSGSAHMAHLPMDRPIKVVDLLSSYVELQEPASRLQLRELASYTVCPPH

QKELEQLVSDDGIYKEQVLAKRLTMLDFLEDYPACEMPFERFLALLPSLKPRYYSISSSP

KVHANIVSMTVGVVKASAWSGRGEYRGVASNYLAELNTGDAAACFIRTPQSGFQMPNDPE

TPMIMVGPGTGIAPFRGFIQARSVLKKEGSTLGEALLYFGCRRPDHDDLYREELDQAEQD

GLVTIRRCYSRVENEPKGYVQHLLKQDTQKLMTLIEKGAHIYVCGDGSQMAPDVERTLRL

AYEAEKAASQEESAVWLQKLQDQRRYVKDVWTGM

>CYP107H1 *Bacillus subtilis* QB928

MTIASSTASSEFLKNPYSFYDTLRAVHPIYKGSFLKYPGWYVTGYEETAAILKDARFKVR

TPLPESSTKYQDLSHVQNQMMLFQNQPDHRRLRTLASGAFTPRTTESYQPYIIETVHHLL

DQVQGKKKMEVISDFAFPLASFVIANIIGVPEEDREQLKEWAASLIQTIDFTRSRKALTE

GNIMAVQAMAYFKELIQKRKRHPQQDMISMLLKGREKDKLTEEEAASTCILLAIAGHETT

VNLISNSVLCLLQHPEQLLKLRENPDLIGTAVEECLRYESPTQMTARVASEDIDICGVTI

RQGEQVYLLLGAANRDPSIFTNPDVFDITRSPNPHLSFGHGHHVCLGSSLARLEAQIAIN

TLLQRMPSLNLADFEWRYRPLFGFRALEELPVTFE

>CYP107J1 *Bacillus subtilis* QB928

MSSKEKKSVTILTESQLSSRAFKDEAYEFYKELRKSQALYPLSLGALGKGWLISRYDDAI

HLLKNEKLKKNYENVFTAKEKRPALLKNEETLTKHMLNSDPPDHNRLRTLVQKAFTHRMI

LQLEDKIQHIADSLLDKVQPNKFMNLVDDYAFPLPIIVISEMLGIPLEDRQKFRVWSQAI

IDFSDAPERLQENDHLLGEFVEYLESLVRKKRREPAGDLISALIQAESEGTQLSTEELYS

MIMLLIVAGHETTVNLITNMTYALMCHHDQLEKLRQQPDLMNSAIEEALRFHSPVELTTI

RWTAEPFILHGQEIKRKDVIIISLASANRDEKIFPNADIFDIERKNNRHIAFGHGNHFCL

GAQLARLEAKIAISTLLRRCPNIQLKGEKKQMKWKGNFLMRALEELPISF

>CYP107K1 *Bacillus subtilis* QB928

MEKLMFHPHGKEFHHNPFSVLGRFREEEPIHRFELKRFGATYPAWLITRYDDCMAFLKDN

RITRDVKNVMNQEQIKMLNVSEDIDFVSDHMLAKDTPDHTRLRSLVHQAFTPRTIENLRG

SIEQIAEQLLDEMEKENKADIMKSFASPLPFIVISELMGIPKEDRSQFQIWTNAMVDTSE

GNRELTNQALREFKDYIAKLIHDRRIKPKDDLISKLVHAEENGSKLSEKELYSMLFLLVV

AGLETTVNLLGSGTLALLQHKKECEKLKQQPEMIATAVEELLRYTSPVVMMANRWAIEDF

TYKGHSIKRGDMIFIGIGSANRDPNFFENPEILNINRSPNRHISFGFGIHFCLGAPLARL

EGHIAFKALLKRFPDIELAVAPDDIQWRKNVFLRGLESLPVSLSK

>CYP109B1 *Bacillus subtilis* QB928

MNVLNRRQALQRALLNGKNKQDAYHPFPWYESMRKDAPVSFDEENQVWSVFLYDDVKKVV

GDKELFSSCMPQQTSSIGNSIINMDPPKHTKIRSVVNKAFTPRVMKQWEPRIQEITDELI

QKFQGRSEFDLVHDFSYPLPVIVISELLGVPSAHMEQFKAWSDLLVSTPKDKSEEAEKAF

LEERDKCEEELAAFFAGIIEEKRNKPEQDIISILVEAEETGEKLSGEELIPFCTLLLVAG

NETTTNLISNAMYSILETPGVYEELRSHPELMPQAVEEALRFRAPAPVLRRIAKRDTEIG

GHLIKEGDMVLAFVASANRDEAKFDRPHMFDIRRHPNPHIAFGHGIHFCLGAPLARLEAN

IALTSLISAFPHMECVSITPIENSVIYGLKSFRVKM

>CYP134A1 *Bacillus subtilis* QB928

MSQSIKLFSVLSDQFQNNPYAYFSQLREEDPVHYEESIDSYFISRYHDVRYILQHPDIFT

TKSLVERAEPVMRGPVLAQMHGKEHSAKRRIVVRSFIGDALDHLSPLIKQNAENLLAPYL

ERGKSDLVNDFGKTFAVCVTMDMLGLDKRDHEKISEWHSGVADFITSISQSPEARAHSLW

CSEQLSQYLMPVIKERRVNPGSDLISILCTSEYEGMALSDKDILALILNVLLAATEPADK

TLALMIYHLLNNPEQMNDVLADRSLVPRAIAETLRYKPPVQLIPRQLSQDTVVGGMEIKK

DTIVFCMIGAANRDPEAFEQPDVFNIHREDLGIKSAFSGAARHLAFGSGIHNCVGAAFAK

NEIEIVANIVLDKMRNIRLEEDFCYAESGLYTRGPVSLLVAFDGA

>CYP152A1 *Bacillus subtilis* QB928

MNEQIPHDKSLDNSLTLLKEGYLFIKNRTERYNSDLFQARLLGKNFICMTGAEAAKVFYD

TDRFQRQNALPKRVQKSLFGVNAIQGMDGSAHIHRKMLFLSLMTPPHQKRLAELMTEEWK

AAVTRWEKADEVVLFEEAKEILCRVACYWAGVPLKETEVKERADDFIDMVDAFGAVGPRH

WKGRRARPRAEEWIEVMIEDARAGLLKTTSGTALHEMAFHTQEDGSQLDSRMAAIELINV

LRPIVAISYFLVFSALALHEHPKYKEWLRSGNSREREMFVQEVRRYYPFGPFLGALVKKD

FVWNNCEFKKGTSVLLDLYGTNHDPRLWDHPDEFRPERFAEREENLFDMIPQGGGHAEKG

HRCPGEGITIEVMKASLDFLVHQIEYDVPEQSLHYSLARMPSLPESGFVMSGIRRKS

>CYP102A36 *Bacillus subtilis* subsp. *spizizenii* W23

MKETSPIPQPKTYGPLGNLPLIDKDKPTLSLIKLAEEQGPIFQIHTPAGTTIVVSGHELV

KEVCDEERFDKSIEGALEKVRAFSGDGLFTSWTHEPNWRKAHNILMPTFSQRAMKDYHEK

MVDIAVQLIQKWARLNPNEAVDVPGDMTRLTLDTIGLCGFNYRFNSYYRETPHPFINSMV

RALDEAMHQMQRLDFQDKLMVRTKRQFHHDIQTMFSLVDSIIAERRSNGDQDEKDLLARM

LNVEDPETGEKLDDENIRFQIITFLIAGHETTSGLLSFAIYFLLKHPDKLKKAYEEVDRV

LTGAAPTYKQVLELKYIRMILNESLRLWPTAPAFSLYPKEDTVIGGKYMITTQDRISVLI

PQLHRDQDAWGEDAEEFRPERFEHQDQVPHHAYKPFGNGQRACIGMQFALHEATLVLGMV

LKYFTLIDHENYELDIKQTLTLKPGDFRIRVQTRHQEAIHTDVPAAEKEAPVEQKEETET

KGASVIGLNNRPLLVLYGSDTGTAEGVARELADTASLHGVRTEVAPLNDRIGKLPKEGAV

VIVTASYNGKPPSNAGQFVQWLQEIKPGELEGVHYAVFGCGDHNWASTYQYVPRFIDEQL

AEKGATRFSERGEGDVSGDFEGQLDEWKKSMWTDAIKAFGLELNENADKERSTLSLQFVR

GLGESPLARSYEAAHASIAENRELQSADSDRSTRHIEIALPPDVEYREGDHLGVLPRNSQ

TNVSRILHRFGLKGTDQVTLSASGRSAGHLPLGRPVSLHDLLSYSVEVQEAATRAQIREL

AAFTVCPPHKRELEDMTEEGVYQEQILKKRISMLDLLEQYESCEMPFERFLELLRPLKPR

YYSISSSPRVNPEQASITVGVVRGPAWSGRGEYRGVSSSYLAERQAGDDVVMFVRTPESR

FQLPEDPETPIIMVGPGTGVAPFRGFLQARAALKREGKALGEAHLYFGCRNDHDFIYRDE

LEQFEKDGIVTVHTAFSRKEGMPKTYVQHLMADHAETLISILDRGGRLYVCGDGSKMAPD

VEAGLQKAYQSVHGTGEEEAQNWLKHLQDTGIYAKDVWSGV

>CYP102A48 *Bacillus subtilis* subsp. *spizizenii* W23

MKQASAIPQPKTYGPLKNLPHLEKEQLSQSLWRIADELGPIFRFDFPGVSSVFVSGHNLV

AEVCDESRFDKNLGKGLLKVREFGGDGLFTSWTNEPNWQKAHRILLPSFSQKAMKGYHSM

MLDIATQLIQKWSRLNPNEEIDVADDMTRLTLDTIGLCGFNYRFNSFYRDSQHPFITSML

RALKEAMNQSKRLGLQDKMMVKTKLQFQKDIEVMNSLVDRMIAERKANPDENIKDLLSLM

LYAKDPVTGETLDDENIRYQIITFLIAGHETTSGLLSFAIYCLLTHPEKLKKAQEEADRV

LTDDTPEYRQIQQLKYIRMVLNETLRLYPTAPAFSLYAKEDTVLGGEYPISKGQPVTVLI

PKLHRDQNAWGEDAEDFRPERFEDPSRIPHHAYKPFGNGQRACIGMQFALQEATMVLGLV

LKHFDVMNHTGYELKIKEALTIKPDDFKITVKPRKTAAINVQRKEQADIKTETKPKETKP

KHGTPLLVLYGSNLGTAEGIAGELAAYGRQMGFTAETAPLDDYIGKLLEQGAVVIVTASY

NGAPPDNAAGFVEWLEGLEEGRLNGVSYAVFGCGNRSWASTYQRIPRLIDDMMKAKGASR

LTAIGEGDAADDFESHRESWENRFWKETMEAFAINEISQKEDRPSLSIAFLSEATETPLA

KAYGAFEGNVLENRELQTADSPRSTRHIELQIPDAKTYKEGDHIGILPKNSQKLVQRVLS

RFDLQSNHVIKITGGPHMAHLPMDRPIKVTDLLASYVELQDPASRLQLRELASYTVCPPH

KKELEQLVSDDGIYKEQVLAKRLTMLDLLEDYPACEMPFERFLALLPSLKPRYYSISSSP

KVHANIVSMTVGVVKASAWSGRGEYRGVASNYLAELNTGDAAACFIRTPQSGFQMPDDPE

TPIIMVGPGTGIAPFRGFIQARSVLKKEGSALGEALLYFGCRHPDHDDLYREELDQAEQD

GLVTIRRCYSRVENESKGYVQHLLKQDTQKLMSLIEKGASIYVCGDGSQMAPDVENSLRQ

AYETEKGASEEESADWLQKLQDQKRYVKDVWTGM

>CYP107H3 *Bacillus subtilis* subsp. *spizizenii* W23

MTIASSTASSEFLKNPYPFYETLRAVHPIYKGSFLKYPGWYVTGYEETAAILKDARFKVR

TPLPETSIKYQDLTHVQNQMMLFQNQPDHRRLRMPASGAFTPRMAERYRPYIDETVHHLL

DQVQGEKKMEVISDFAFPLASYVIANIIGVPAEDREQLKDWAASLIQTIDFTRSRKALIE

GNHSAVQAMAYFRELIQKRKRHPQQDMISMLLKGKEKDKMTEEEVASTCILLAIAGHETT

VNLISNSILCLLQHPEQLLELKENPDLIATAVEECLRFESPTQMTARVASEDIDISGVTI

RKGEQVYLLLGAANRDPNIFTRADVFDITRSPNPHLSFGHGHHVCLGSSLARLEAQMAIH

TLLQRMPGLKLAESKWRYRPLFGFRALEELPVTFE

>CYP107K1 *Bacillus subtilis* subsp. *spizizenii* W23

MEKLMFHPHGKEFHLNPFSVLGRFREEDPVHQFELKRFGGTYPAWLITRYDDCMAFLKDN

RITRDVKNVMSQEQIKMLNVSEDIDFVSDHMLAKDTPDHTRLRSLVHQAFTPRTIENLRG

SIEQITEQLLDEMEKENKADIMKSFASPLPFIVISELMGIPKEDRAQFQIWTNAMVDTSE

SNRELTNQALREFKDYIAKLIQDRRIQPKDDLISKLVHAEENGSKLSEKELYSMLFLLVV

AGLETTVNLLGSGTLALLQHKEEFEKLKQYPEMIATAVEELLRYTSPVVMMANRWAIEDF

TYKGHSIKRGDMIFIGIGSANRDPNFFENPETLNLNRSPNRHISFGFGIHFCLGAPLARL

EGHIAFNALLKRFPDIELAAAPDDIHWRKNVFLRGLENLPVSLSK

>CYP109A1 *Bacillus subtilis* subsp. *spizizenii* W23

MTNQTARSSKKERYANLIPMEELHSEKDRLFPFPIYDKLRRESPVRYDPLRDCWDVFKYD

DVQFVLKNPKLFSSKRGIQTESILTMDPPKHTKLRALVSRAFTPKAVKQLETRIKDVTAF

LLQEARQKSTIDIIEDFAGPLPVIIIAEMLGAPIEDRHLIKTYSDVLVAGAKDSSDKAVA

DMVHNRRDGHAFLSDYFRDILSKRRAEPKEDLMTMLLQAEIDGEYLTEEQLIGFCILLLV

AGNETTTNLIANAVRYLTEDSVVQQQVRQNTDNVANVIEETLRYYSPVQAIGRVATEDTE

LGGVFIKKGSSVISWIASANRDEDKFCKPDCFKIDRPSYPHLSFGFGIHFCLGAPLARLE

ANIALSSLLSMSACIEKAAHDEKLEAIPSPFVFGVKRLPVRITFK

>CYP109B1 *Bacillus subtilis* subsp. *spizizenii* W23

MSVLNRRQALQRALLNGKNKQDAYHPFPWYESMRKDAPVSFDEENQVWSVFLYDDVKKVI

GDKELFSSYMPQQSSAIGNSIINMDPPRHTQIRSVVNKAFTPRVMKQWEPRIQEITDELI

QKFQGRSEFDLVHDFSYPLPVIVISELLGVPSEHMDQFKTWSDLLVSTPKDKSEEAEEAF

LEERNKCEEELAAFFANIIEEKRNKPAQDIISILVEAEETGEKLSGEELVPFCTLLLVAG

NETTTNLISNAMYSILETPDVYNELRSHPELTPQAVEEALRFRAPAPVLRRIAKRDTEIG

GHLIKEGDMVLAFVASANRDETKFDRAHLFDIHRHPNPHIAFGHGIHFCLGAPLARLEAK

IALTSLISAFPHMECVSITPIENSVIYGLKSFRVNI

>CYP134A1 *Bacillus subtilis* subsp. *spizizenii* W23

MNQSIKLFSVLSDQFQENPYAYFSQLREEDPVHYEESIDSYFISRYHDVRYILQHPDIFT

TKSLVERAEPVMRGPVLAQMHGKEHSAKRRIVVRSFVGDALDHLSPLIKQNAENLLAPYL

ERGRIDLVNDFGKTFAVCVTMDMLGLDKRDHEKIAEWHSGVADFITSLSQTPEARAHSLW

CSEQLSQYLMPVIEERRVNPGSDLISILCTSEYEGMAMSDKDILALILNVLLAATEPADK

TLALMIYHLLNNPDQMNDVLADRSLVPRAIAETLRYKPPVQLIPRQLSQDTVIGGMEIKK

DTIVFCMIGAANRDPEAFERPDVFHIHREDLGIKSAFSGAARHLAFGSGIHNCVGAAFAK

TEIEIVANIVLDKMRNIRLEEGFRYAESGLYTRGPVSLHVAFDRA

>CYP152A1 *Bacillus subtilis* subsp. *spizizenii* W23

MNEQIPHDKSLDNSVALMREGYLFIKNRKEHYHSDLFQARLLGKTFICMSGAEAAKIFYD

TERFQRQGALPKRVQKSLFGVNAIQTMDGDAHIHRKLLFLSLMTPPHQKRLAELMTEEWE

AAVTRWEKADQVVLFEEAKEILCRVACYWAGVPLKETEVKERADDFIDMVDAFGAVGPRH

WKGRRARPRAEEWIERMIEDVRAGLLKIPAGTALHEMAFHTQEDGNQLDSRMAAIELINV

LRPIVAISYFLVFSALALHKHPKYKEWLRSGSSREREMFVQEVRRYYPFGPFLGALVKKD

FEWNNCEFKKGTSVLLDVYGTNHDPRLWDNPDEFRPERFAEWEENPFDLIPQGGGHAEKG

HRCPGEGITIEVMKASLDFLVNQIEYEVPEQSLHYSLARMPSLPESGFVMSGIRRKR

>CYP102A5 *Bacillus subtilis* subsp. *subtilis* BAB-1

MEKKVSAIPQPKTYGPLGNLPLIDKDKPTLSFIKIAEEYGPIFQIQTLSDTIIVVSGHEL

VAEVCDETRFDKSIEGALAKVRAFAGDGLFTSETHEPNWKKAHNILMPTFSQRAMKDYHA

MMVDIAVQLVQKWARLNPNENVDVPEDMTRLTLDTIGLCGFNYRFNSFYRETPHPFITSM

TRALDEAMHQLQRLDIEDKLMWRTKRQFQHDIQSMFSLVDNIIAERKSSGDQEENDLLSR

MLNVKDPETGEKLDDENIRFQIITFLIAGHETTSGLLSFAIYFLLKNPDKLKKAYEEVDR

VLTDPTPTYQQVMKLKYIRMILNESLRLWPTAPAFSLYAKEDTVIGGKYPIKKGEDRISV

LIPQLHRDKDAWGDNVEEFQPERFEELDKVPHHAYKPFGNGQRACIGMQFALHEATLVMG

MLLQHFEIIDYQNYQLDVKQTLTLKPGDFKIRILPRKQTISHPTVLAPTEDKLKNDEIKQ

HVQKTPSIIGADNLSLLVLYGSDTGVAEGIARELADTASLEGVQTEVVALNDRIGSLPKE

GAVLIVTSSYNGKPPSNAGQFVQWLEELKPDELKGVQYAVFGCGDHNWASTYQRIPRYID

EQMAQKGATRFSKRGEADASGDFEEQLEQWKQNMWSDAMKAFGLELNKNMEKERSTLSLQ

FVSRLGGSPLARTYEAVYASILENRELQSSSSDRSTRHIEVSLPEGATYKEGDHLGVLPV

NSEKNINRILKRFGLNGKDQVILSASGRSINHIPLDSPVSLLDLLSYSVEVQEAATRAQI

REMVTFTACPPHKKELEALLEEGVYHEQILKKRISMLDLLEKYEACEIRFERFLELLPAL

KPRYYSISSSPLVAHNRLSITVGVVNAPAWSGEGTYEGVASNYLAQRHNKDEIICFIRTP

QSNFELPKNPETPIIMVGPGTGIAPFRGFLQARRVQKQKGMNLGQAHLYFGCRHPEKDYL

YRTELENDERDGLISLHTAFSRLEGHPKTYVQHLIKQDRINLISLLDNGAHLYICGDGSK

MAPDVEDTLCQAYQEIHEVSEQEARNWLDRVQDEGRYGKDVWAGI

>CYP102A36 *Bacillus subtilis* subsp. *subtilis* BAB-1

MKETSPIPQPKTFGPLGNLPLIDKDKPTLSLIKLAEEQGPIFQIHTPAGTTIVVSGHELV

KEVCDEERFDKSIEGALEKVRAFSGDGLFTSWTHEPNWRKAHNILMPTFSQRAMKDYHEK

MVDIAVQLIQKWARLNPNEAVDVPGDMTRLTLDTIGLCGFNYRFNSYYRETPHPFINSMV

RALDEAMHQMQRLDVQDKLMVRTKRQFRHDIQTMFSLVDSIIAERRSNGDQDEKDLLARM

LNVEDPETGEKLDDENIRFQIITFLIAGHETTSGLLSFATYFLLKHPDKLKKAYEEVDRV

LTDAAPTYKQVLELTYIRMILNESLRLWPTAPAFSLYPKEDTVIGGKFPITTKDRISVLI

PQLHRDRDAWGKDAEEFRPERFEHQDQVPHHAHKPFGNGQRACIGMQFALHEATLVLGMI

LKYFTLIDHENYELDIKQTLTLKPGDFHIRVQSRNQEAIHADVQAAEKAVSDEQKEKTEA

KGASVIGLNNRPLLLLYGSDTGTAEGVARELADTASLHGVRTETAPLNDRIGKLPKEGAV

VIVTSSYNGKPPSNAGQFVQWLQEIKPGELEGVHYAVFGCGDHNWASTYQYVPRFIDEQL

AEKGATRFSARGEGDVSGDFEGQLDEWKKSMWADAIKAFGLELNENADKERSTLSLQFVR

GLGESPLARSYEASHASIAENRELQSADSDRSTRHIEIALPPDVEYQEGDHLGVLPKNSQ

TNVSRILHRFGLKGTDQVTLSASGRSAGHLPLGRPVSLHDLLSYSVEVQEAATRAQIREL

AAFTVCPPHRRELEELSAEGVYQEQILKKRISMLDLLEKYEACDMPFERFLELLRPLKPR

YYSISSSPRVNPRQASITVGVVRGPAWSGRGEYRGVASNDLAERKAGDDVVMFIRTPESR

FQLPEDPETPIIMVGPGTGVAPFRGFLQAREVLKREGKTLGEAHLYFGCRNDRDFIYRDE

LEQFEKDGIVTVHTAFSRKEGMPKTYVQHLMADHAETLISILDRGGRLYVCGDGSKMAPD

VEAALQKAYQSVHGTGEQEVQNWLRHLQDTGMYAKDVWAGI

>CYP102A48 *Bacillus subtilis* subsp. *subtilis* BAB-1

MKQASAIPQPKTYGPLKNLPHLEKEQLSQSLWRIADELGPIFRFDFPGVSSVFVSGHNLV

AEVCDESRFDKNLGKGLQKVREFGGDGLFTSWTHEPNWQKAHRILLPSFSQKAMKGYHSM

MLDIATQLIQKWSRLNPNEEIDVADDMTRLTLDTIGLCGFNYRFNSFYRDSQHPFITSML

RALKEAMNQSKRLGLQDKMMVKTKLQFQKDIEVMNSLVDRMIAERKANPDEDIKDLLSLM

LYAKDPVTGETLDDENIRYQIITFLIAGHETTSGLLSFAIYCLLTHPEKLKKAQEEADRV

LTDDTPEYKQIQQLKYIRMVLNETLRLYPTAPAFSLYAKEDTVLGGEYPISKGQPVTVLI

PKLHRDQNAWGPDAEDFRPERFEDPSSIPHHAYKPFGNGQRACIGMQFALQEATMVLGLV

LKHFELINHTGYELKIKEALTIKPDDFKITVKPRKTAAINVQRKEQADIKAETKPKETKP

IHGTPLLVLYGSNLGTAEGIAGELAAQGRQMGFAAETAPLDDYIGKLPEEGAVVIVTASY

NGAPPDNAAGFVEWLKELKEGQLKGVSYAVFGCGNRSWASTYQRIPRLIDDMMKAKGASR

LTEIGEGDAADDFESHRESWENRFWKETMDAFDINEIAQKEDRPSLSITFLSETTETPVA

KAYGAFEGVVLENRELQTADSTRSTRHIELEIPAGKTYKEGDHIGIMPKNSRELVQRVLS

RFGLQSNHVIKVSGSAHMAHLPMDRPIKVVDLLSSYVELQEPASRLQLRELASYTVCPPH

QKELEQLVSDDGIYKEQVLAKRLTMLDFLEDYPACEMPFERFLALLPSLKPRYYSISSSP

KVHANIVSMTVGVVKASAWSGRGEYRGVASNYLAELNTGDAAACFIRTPQSGFQMPDESE

TPMIMVGPGTGIAPFRGFIQARSVLKKEGSTLGEALLYFGCRRPDHDDLYREELDQAEQD

GLVTIRRCYSRVENEPKEYVQHLLKQDTQKLMTLIEKGAHIYVCGDGSQMAPDVEKTLRL

AYEAEKGASQEESAEWLQKLQDQKRYVKDVWTGM

>CYP106B4 *Bacillus subtilis* subsp. *subtilis* BAB-1

MASPENVILVHEISKLKTKEELWNPYEWYRLMRDNHPVHYDEEQDVWNVFLYDDVNRVLS

DYRLFSSRRERRQFSIPPLETRININSTDPPEHRNVRSIVSKAFTPRSLEQWKPRIQAIA

DELVQHIEKCSEVNIVEQFAAPLPVTVISDLLGVPTTDRKKMKEWSDILFMPYSKEKFND

LDAEKGIALHEFKAYLLPIVQEKRYHLTDDIISDLIRAEYEGERLTDEEIVTFSLGLLAA

GNETTTNLIINSFYCFLVDSPGIYEELRKEPNLILKAIEEVLRYRFPVTLARRITEDTNI

FGPFMKKNQMIVAWVSAANLDEKKFSQASQFNVHRTGNEKHLTFGKGPHFCLGAPLARLE

AEIALTTFINAFEKIELSPSFCLEKCILENEQTLKYLPIRLKAK

>CYP107J1 *Bacillus subtilis* subsp*. subtilis* BAB-1

MSSKEKNSVTILTESQLSSRAFKDEAYEFYKELRKSQPLYPLSLGALGKGWLISRYDDAI

HLLKNEKLKKNYENVITAKEENRPVLLKNEEPLTKHMLNSDPPDHNRLRTLVQKAFTHRM

ILQLEDKIQHIADSLLDKVQPNKLMNLVDDYAFPLPIIVISEMLGIPLEDRQKFRVWSQV

IIDFSDAPERLQENDHLLGEFVEYLESLVRKKRSEPSGDLISALIQAESEGAQLSTEELY

SMIMLLIVAGHETTVNLITNMTYALMCHGDQFEKLRQQPDLMNSAIEEALRFHSPVELTT

IRWTAEPFILHGQEIKRKDVIIISLASANRDEKIFPNADIFDIERKNNRHIAFGHGNHFC

LGAQLARLEAKIAISTLLRRCPNIQLKGEKEQMKWKGNFLMRALEELPLSF

>CYP107J3 *Bacillus subtilis* subsp. *subtilis* BAB-1

MSMKNKVGLSIEDGINLASAQFKEDAYEIYKESRKKQPILFVNQVEIGKEWIITRYEDAL

PLLKDNRLKKDWTNVFSQDTKNMYLSVDNSDHLTTHMLNSDPPNHSRLRSLVQKAFTPKM

IAQLDGRIQRIADDLISNIERKGTLNLVDDYSFPLPIIVISEMLGIPKEDQAKFRIWSHA

VIASPETPEEIKETEKQLSEFITYLQYLVDIKRKEPKEDLVSALILAESEGHKLSARELY

SMIMLLIVAGHETTVNLITNTVLALLENPNQLQLLKDNPKLIDSAIEEGLRYYSPVEVTT

ARWAAEPFQIHHQTIQKGDMVIIALASANRDETVFENPEIFDITRENNRHIAFGHGSHFC

LGAPLARLEAKIAITTLFNRMPELQIKGNREEIKWQGNYLMRSLEELPLTF

>CYP107K1 *Bacillus subtilis* subsp. *subtilis* BAB-1

MEKLMFHPHGKEFHHNPFSVLGRFREEEPIHRFELKRFGATYPAWLITRYDDCMAFLKDN

RITRDVKNVMDQEQIKMLNVSEDIDFVSDHMLAKDTPDHTRLKSLVHQAFTPRTIENLRG

SIEQIAEQLLDEMEKENKADIMKSFASPLPFIVISELMGIPKEDRSQFQIWTNAMVDTSE

GNRELTNQALREFKDYIAKLIHDRRIKPKDDLISKLVHAEENGSKLSEKELYSMLFLLVV

AGLETTVNLLGSGTLALLQHKKECEKLKQHPEMIATAVEELLRYTSPVVMMANRWAIEDF

TYKGHSIKRGDMIFIGIGSANRDPNFFENPEILNINRSPNRHISFGFGIHFCLGAPLARL

EGHIAFNALLKRFPDIELAVAPEDIQWRKNVFLRGLESLPVSLSK

>CYP109B1 *Bacillus subtilis* subsp. *subtilis* BAB-1

MNVLNRRQALQRALLNGKNKQDAYHPFPWYESMRADAPVSFDEENQVWSVFLYDDVKKVV

GDKELFSSYMPQQTSSIGNSIINMDPPKHTKIRSVVNKAFTPRVMKQWEPRIQEITDELI

QKFQERSEFDLVHDFSYPLPVIVISELLGVPSKHMDQFKAWSDLLVSTPKDKSEEAEKAF

LEERDKCEEELAAFFAGIIEEKRNKPAQDIISILVEAEETGKKLSGEELIPFCTLLLVAG

NETTTNLISNAMYSILETPGVYEELRSHPELMPQAVEEALRFRAPAPVLRRIAKRDTKIG

GHLIKEGDMVLAFVASANRDEAKFDRPHMFDIRRHPNPHIAFGHGIHFCLGAPLARLEAN

IALTSLISAFPHMECVSITPIENSVIYGLKSFRVKM

>CYP134A1 *Bacillus subtilis* subsp. *subtilis* OH 131.1

MSQSIKLFSVLSDQFQNNPYAYFSQLREEDPVHYEESIDSYFISRYHDVRYILQHPDIFT

TKSLVERAEPVMRGPVLAQMHGKEHSAKRRIVVRSFIGDALDHLSPLIKQNAENLLAPYL

ERGKIDLVNDFGKTFAVCVTMDMLGLDKRDHEKISEWHSGVADFITSISQSPEARAHSLW

CSEQLSQYLMPVIKERRVNPGSDLISILCTSEYEGMALSDKDILALILNVLLAATEPADK

TLALMIYHLLNNPEQMNDVLADRSLVPRAIAETLRYKPPVQLIPRQLSQDTVVGGMEIKK

DTIVFCMIGAANRDPEAFEQPDVFNIHREDLGIKSAFSGGARHLAFGSGIHNCVGAAFAK

NEIKIVANIVLDKMRNIRLEEDFCYAESGLYTRGPVSLLVAFDGA

>CYP102A48 *Bacillus subtilis* subsp. *subtilis* OH 131.1

MKQASAIPQPKTYGPLKNLPHLEKEQLSQSLWRIADELGPIFRFDFPGVSSVFVSGHNFV

AEVCDESRFDKNLGKGLQKVREFGGDGLFTSWTHEPNWQKAHRILLPSFSQKAMKGYHSM

MLDIATQLIQKWSRLNPNEEIDVADDMTRLTLDTIGLCGFNYRFNSFYRDSQHPFITSML

RALKEAMNQSKRLGLQDKMMVKTKLQFQKDIEVMNSLVDRMIAERKANPDENIKDLLSLM

LYAKDPVTGETLDDENIRYQIITFLIAGHETTSGLLSFAIYCLLTHPEKLKKAQEEADCV

LTDDTPEYKQIQQLKYIRMVLNETLRLYPTAPAFSLYAKEDTVLGGEYPISKGQPVTVLI

PKLHRDQNAWGPDAEDFRPERFEDPSSIPHHAYKPFGNGQRACIGMQFALQEATMVLGLV

LKHFELINHTGYELKIKEALTIKPDDFKITVKPRKTAAINVQRKEQADIKAETKPKETKP

KHGTPLLVLYGSNLGTAEGIAGELVAQGCQMGFTAETAPLDDYIGKLPEEGAVVIVTASY

NGAPPDNAAGFVEWLKELEEGQLKGVSYAVFGCGNRSWASTYQRIPRLIDDMMKAKGASR

LTAIGEGDAADDFESHRESWENRFWKETMDAFDINELAQKEDRPSLSITFLSEATETPVA

KAYGAFEGIVLENRELQTAASTRSTRHIELEIPAGKTYKEGDHIGILPKNSRELVQRVLS

RFGLQSNHVIKVSGSAHMAHLPMDRPIKVVDLLSSYVELQEPASRLQLRELASYTVCPPH

QKELEQLVSDDGIYKEQVLAKRLTMLDFLEDYPACEMPFERFLALLPSLKPRYYSISSSP

KVHANIVSMTVGVVKASAWSGRGEYRGVASNYLAELNTGDAAACFIRTPQSGFQMPDEPE

TPMIMIGPGTGIAPFRGFIQARSVLKKEGSTLGEALLYFGCRRPDHDDLYREELDQAEQD

GLVTIRRCYSRVENEPKGYVQHLLKQDTQKLMTLIEKGAHIYVCGDGSQMAPDVEKTLRL

AYEAEKGASQEESAEWLQKLQDQRRYVKDVWTGM

>CYP107H1 *Bacillus subtilis* subsp. *subtilis* OH 131.1

MTIASSTASYEFLKNPYSFYDTLRAVHPIYKGSFLKYPGWYVTGYEETAAILKDARFKVR

TPLPESSTKYQDLSHVQNQMMLFQNRPDHRRLRTLASGAFTPRTTESYQPYIIETVHHLL

DQVQGKKKMEVISDFAFPLASFVIANIIGVPEEDREQLKEWAASLIQTIDFTRSRKALTE

GNIMAVQAMAYFKELIQKRKRHPQQDMISMLLKGREKDKLTEEEAASTCILLAIAGHETT

VNLISNSVLCLLQHPEQLLKLRENPDLIGTAVEECLRYESPTQMTARVASEDIDICGVTI

RQGEQVYLLLGAANRDPSIFTNPDVFDITRSPNPHLSFGHGHHVCLGSSLARLEAQIAIN

TLLQRMPSLKLADFEWRYRPLFGFRALEELPVTFE

>CYP107J1 *Bacillus subtilis* subsp. *subtilis* OH 131.1

MSSKEKKSVTILTESQLSSRAFKDEAYEFYKELRKSQPLYPLSLGALGKGWLISRYDDAI

HLLKYEKLKKNYENVFTAKEKRPALLKNEETLTKHMLNSDPPDHNRLRTLVQKAFTHRMI

LQLEDKIQHIADSLLDKVQPNKFMNLVDDYAFPLPIIVISEMLGIPLEDRQKFRVWSQAI

IDFSDAPERLQENDHLLGEFVEYLESLVRKKRREPAGDLISALIQAESEGTQLSTEELYS

MIMLLIVAGHETTVNLITNMTYALMCHHDQLEKLRQQPDLMNSAIEEALRFHSPVELTTI

RWTAEPFILHGQEIKRKDVIIISLASANRDEKIFPNADIFDIERKNNRHIAFGHGNHFCL

GAQLARLEAKIAISTLLRRCPNIQLKGEKKQMKWKGNFLMRALEELPISF

>CYP107K1 *Bacillus subtilis* subsp. *subtilis* OH 131.1

MEKLMFHPHGKEFHHNPFSVLGRFREEEPIHRFELKRFGATYPAWLITRYDDCMAFLKDN

RITRDVKNVMNQEQIKMLNVSEDIDFVSDHMLAKDTPDHTRLRSLVHQAFTPRTIENLRG

SIEQIAEQLLDEMEKENKADIMKSFASPLPFIVISELMGIPKEDRSQFQIWTNAMVDTSE

GNRELTNQALREFKDYIAKLIHDRRIKPKDDLISKLVHAEENGSKLSEKELYSMLFLLVV

AGLETTVNLLGSGTLALLQHKKECEKLKQYPEMIATAVEELLRYTSPVVMMANRWAIEDF

TYKGHSIKRGDMIFIGIVSANRDPNFFENPEILNINRSPNRHISFGFGIHFCLGAPLARL

EGHIAFNALLKRFPDIQLAVAPDDIQWRKNVFLRGLESLPVSLSK

>CYP109B1 *Bacillus subtilis* subsp. *subtilis* OH 131.1

MSVLNRRQALQRALLNGKNKQDAYHPFPWYESMRKDSPVSFDEENQVWSVFLYDDVKKVV

GDKELFSSYMPQQTSSIGNSIINMDPPKHTKIRSVVNKAFTPRVMKQWEPRIQEITDELI

QKCQGRSEFDLVHDFSYPLPVIVISELLGVPSAHMDQFKAWSDLLVSTPKDKSEEAEKAF

LEERDKCEEELAAFFAGIIEEKRNKPAQDIISILVEAEETGEKLSGEELIPFCTLLLVAG

NETTTNLISNAMYSILETPGVYEELRSHPELMPQAVEEALRFRAPAPVLRRIAKRDTEIG

GHLIKEGDMVLAFVASANRDEAKFDRPHMFDIHRHPNPHIAFGHGIHFCLGAPLARLEAN

IALTSLISAFPQMECVSITPIENSVIYGLKSFRVKM

>CYP152A1 *Bacillus subtilis* subsp. *subtilis* OH 131.1

MNEQIPHDKSLDNSLTLLKEGYLFIKNRTERYNSDLFQARLLGKNFICMTGAEAAKVFYD

TDRFQRQNALPKRVQKSLFGVNAIQGMDGSAHIHRKMLFLSLMTPPHQKRLAELMTEEWR

AAVTRWEKTDEVVLFEEAKEILCRVACYWAGVPLKETEVKERADDFIDMVDAFGAVGPRH

WKGRRARPRAEEWIEVMIEDARAGLLKTTPGTALHEMAFHTQEDGSQLDSRMAAIELINV

LRPIVAISYFLVFSALALHEHPKYKEWLRSGNSREREMFVQEVRRYYPFGPFLGALVKKD

FVWNNCEFKKGTSVLLDVYGTNHDPRLWEHPDEFRPERFAEREENPYDMIPQGGGHAEKG

HRCPGEGITIEVMKASLDFLVHQIEYDVPEQSLHYSLARMPSLPESGFVMSGIRRKS

>CYP102A38 *Bacillus amyloliquefaciens* LL3

MKETSPIPKPKTFGPLGNLPLLDKDKPTMSLIKLAKEQGPIFQLHTPAGTIIVVSGHELV

KEVCDEERFDKSIEGALEKVRAFSGDGLFTSWTHEPNWRKAHNILMPTFSKRAMKDYHSM

MTDIAVQLIQKWARLNPDEAVDVPADMTRLTLDTIGLCGFNYRFNSYYRETPHPFINSMV

RALDEAMHQMQRLDVQDKLMIRTKRQFHHDIQAMFSLVDSIIAERRSDGGRDEKDLLARM

LNVEDPETGEKLDDENIRFQIITFLIAGHETTSGLLSFAIYFLLKHPRVLEKAYEEADRV

LTDPVPSYKQVLELTYIRMILQESLRLWPTAPAFSLYAKEDTVIGGKYPITTKDRISVLI

PQLHRDKDAWGDNAEEFYPERFEHPDQVPHHAYKPFGNGQRACIGMQFALHEATLVLGMI

LQHFTFTDHTDYELDIKQTLTLKPNDFHIRVRPRSKEAVPAAFTAAEKAAEDVTKEKQET

KGASIIGLDNRPLLILYGSDTGTAEGVAWELADTAGMHGVRTETAPLNDRIGKLPKEGAL

LIITSSYNGKPPSNAGQFVQWLEEVKQGELEGVRYAVFGCGDHNWAATYQAVPRLIDEKL

AEKGAERFSSRGEGDVSGDFEGKLDEWKKSMWKDAMKAFGLKLNENAEKERSTLGLQFVS

GLGGSPLAQTYEAVYASVAENRELQAPESDRSTRHIEIILPKEAAYNEGDHLGVLPVNSK

EQVSRVLRRYNLNGNDQVLLTASGQSAAHLPLDRPVRLHDLLSSCVELQEAASRAQIREM

AAYTVCPPHKHELEGLLEEGVYQEQILTLRVSMLDLLEKYEACELPFERFLELLRPLKPR

YYSISSSPLKHPGQASITVGVVRGPARSGLGEYRGVASNYLADRSPDDGIVMFVRTPETK

FRLPEDPEKPIIMVGPGTGVAPFRGFLQARAALKGEGKELGEAHLYFGCRNDYDFIYRDE

LEAYEKDGIVTLHTAFSRKEGVPKTYVQHLMAEDAETLISILDRGGHLYVCGDGSKMAPD

VEETLQKAYQSAHGTDERQAQEWLLDLQTKGIYAKDVWAGI

>CYP102A49 *Bacillus amyloliquefaciens* LL3

MKQLSAIPQPKTYGPLKNLPHLEKEKLSQSLWKIAEEYGPIFRFEFPSSVGVFVSGRELA

AEVCDEKRFDKNLGKALLKVREFGGDGLFTSWTNEKNWQKAHRILLPSFSQKAMKGYHSM

MLDIAMQLVQKWSRLNPNEEIDVAEDMTRLTLDTIGLCGFHYRFNSFYRDTQHPFITSML

RALQEAMRQSQRHSLQDKLMIKTRHQFQQDIEEMNALVDRIIAERRENPDENLSDLLALM

LEAKDPVTGERLDDENIRYQIITFLIAGHETTSGLLSFAIYCLLKNKDKLEKAVQEAERV

LTGETPEYKQIQQLTYIRMVLNETLRLYPTAPAFSLYAKEDTVLGGKYPIEKGQPVTILT

PQLHRDKSAWGEDAELFRPERFSDPAAIPADAYKPFGNGQRACIGMQFALQEATMVLGLV

LKHFELIDHSDYELTIKEALTIKPGDFKIRVKNKDVSNRQTAQNHKAEGSDHKEETKEIP

SHGTPLLILYGSNLGTAEGIAEELADIGRSKGFSTETGPLDDYAGKLPVKGAVVIVTASY

NGAPPDNAAGFVKWMETLEDQELKGVSYAVFGCGDRNWAATYQKIPRLIDKVLEEKGATR

LTSIGEGDNADDFEYSQEAWEDSFWQDIIKAFHIEAAPEQQNKSQLSIEYVSEAAETPIA

KTYKAFEAEVITNKELHTESSTRSVRHIELRLPETETYQEGDHLGVLPQNSGELISRVIH

RFALDPNQHFKISGRHLPHLPMDRPVNALELLASYVELQEPATRAQLRELAAHTVCPPHQ

KELEHLYSDETAYKENVLKKRMTMLDLLEDYPACELPFERFLELLPSLKARYYSISSSPK

ANSGELSITVGVVTAPAWSGRGEYRGVASNYLAGLQNGDHAVCFIRSPQSGFALPENTKT

PLIMVGAGTGIAPFRGFIQARAAEKMSGNSLGEAHLYFGCRHPEEDDLYKDEFDQAEKTG

LVTVHRAYSRLDQDCKVYVQDVLLREAAQIIALLDQGGHLYICGDGSKMAPAVENVLLQA

YEKVHNTDSKVSLSWLEQLQAEGRYAKDVWAGV

>CYP107H2 *Bacillus amyloliquefaciens* LL3

MTAGLSIAHPSLHADSDFWNNPYPFYDKLRAVDPVYQGTVLKYPGRYITGYQEAEAVLKD

TRFKNRIPMPEASAKYKHLKNLQKDMLLFTNHSDHKRLRMLIGKAFTLKKAERLKPFITA

TVHDVLDQIDHSKTADLVSDFAFPVASLVIADILGVPKEDRASFREWTADVIQAIDLTRS

KKSLLKAGGTAGKLTAYFKDLIQKRKTEPQKDVITTLISEEQLTEEEVLASCILLIIAGH

ETTVNLICNGVFSLLKHPAELSKLLENPQLIASATEEFLRFESPAQLTARTASEDCVINQ

HLIKKGEQVYILLGAANRDPEVFHRPHQLDITRNPNPHLAFGKGAHVCIGSSLARIEAQT

AILTLLERAPDIRLVKTDVTYRKLFGFRSLSALPVVLS

>CYP107J6 *Bacillus amyloliquefaciens* LL3

MFSKQNESLTVLTESQLSSRAFKDEAYAFYKELRKSRPLCPLSLGSLGEGWLISRYDDAI

QLLKNEKLKKNYENVFTAKEEKRPVLFKNEGPLRKHMLNSDPPDHNRLRTLVQKAFTHRM

ILQLEDKIQHIADSLLDKVQPSKFMNLVDDYAFPLPIIVISEMLGIPLEDRQNFKIWSQA

IIDFSDAPERLQENDHLLGEFVEYLEYLVRKKRSEPAGDLISALIQAESEGTPLSTEELY

SMIMLLIVAGHETTVNLITNMTYALMCHHDQLEKLLRQPDLMNSAIEEALRFHSPVELTT

IRWTAEPFILHGQEIKRKDVIIISLASANRDEKIFPNADMFDIERKNNRHIAFGHGIHFC

LGAQLARLESKIAISTLLRRCPNIQIKGGKEQIKWKGNFLMRALEELPLTF

>CYP107K3 *Bacillus amyloliquefaciens* LL3

MEKIMFHPHSSEFHENPFAVLSRFREQDPIHKFELQRFGGTFPAWLITRYDDCMAFLKDG

RITRDVKRVMPKELIAKLNVSEDIDFVSEHMLAKDPPDHSRLRSLVHQGFTPRMIEQLRT

GIERITEELLDEMETKADPDIMRDFAAPLPFIVISELLGIPKEDRAKFQVWTSAMVDTSE

SGQDATNQALREFKQYMQTLIEEKRNHPGEDLTSKLIYAEEDGQKLSESELYSMLFLLVV

AGLETTVNLLGSGTLALLLHKDQLENIKRQPDTIQTAVEELLRYTSPVIMMANRWAIEDF

TYKDVSIKKGDMIFIGIGSANRDPEYFDEPDTLNVARTPNRHISFGFGIHFCLGAPLARM

EASIAFNALLKRFPNIELNGSSEDLTWRKNVFLRGLETLPVKF

>CYP109B3 *Bacillus amyloliquefaciens* LL3

MKGKDGLDVYNPFPWYEKMRRESPIQFDEETKVWSVFLYDDAKKVISDKETFSSLMSDMK

SSIAKSMLNMDPPKHTQIRSAVNRAFTPRVLKEWEPRIEEITDNLLKQAKNKGRIDIVKD

LSYPLPVIVISELLGVPSEHMDQFKKWSDILVSMPKDASPEEAEKNQKERDQCETELAAF

FAEIIDSKRKQPGQDIISILIKEEEEGEKLSAEDLIPFCNLLLVAGNETTTNLISNAVYS

ILETPGLYEELRHDPSLIAQTVEETLRFRAPAPFVRRTVRHDTELCGRRLKSGDIVLCYI

ASANRDENKFEQADVFDIHRQSNPHLSFGFGVHFCLGAPLARLEAEVALRGIVKAFSHLE

PVRVEPIQNSVMYGLDSLEAEINENRGEEKR

>CYP152A4 *Bacillus amyloliquefaciens* LL3

MTEQIPHEKGLDNSLALLRDGYVFVKNRAENYRSDVFRARLLGKTFICMSGAEAAKLFYD

TERFQRQQALPKRVQKTLFGTGAIQSMDGERHKHRKLLFMSLMTPPRQKRLAEAVAKQWK

ASAEKWEGADRIVLFDEAKKVLCRAACEWAGVPLKDSEVKERAEDFTDMVDAFGAVGPRH

WKGRRARPKTEKWVEEVIEDVRSGKLQTPEGSALYEMAVHTELDGSRLDSHMAAVELINV

LRPIAAISYFIAFSALALHDHPEYRDKLRSGDDQEAERFVHEVRRYYPFAPFLGAVVKKD

FVWKNCEFKKGASVMLDLYGTNHDSRLWENPNEFRPERFQGREENKFDFIPQGGGDPADG

HRCPGEGMTVEVMKASLAFLTNEIEYDVPPQDLSFSLSRMPALPESGFVISDVRRK

>CYP102A38 *Bacillus amyloliquefaciens* TA208

MKETSPIPKPKTFGPLGNLPLLDKDKPTMSLIKLAKEQGPIFQLHTPAGTIIVVSGHELV

KEVCDEERFDKSIEGALEKVRAFSGDGLFTSWTHEPNWRKAHNILMPTFSKRAMKDYHSM

MTDIAVQLIQKWARLNPDEAVDVPADMTRLTLDTIGLCGFNYRFNSYYRETPHPFINSMV

RALDEAMHQMQRLDVQDKLMIRTKRQFHHDIQAMFSLVDSIIAERRSDGGRDEKDLLARM

LNVEDPETGEKLDDENIRFQIITFLIAGHETTSGLLSFAIYFLLKHPRVLEKAYEEADRV

LTDPVPSYKQVLELTYIRMILQESLRLWPTAPAFSLYAKEDTVIGGKYPITTKDRISVLI

PQLHRDKDAWGDNAEEFYPERFEHPDQVPHHAYKPFGNGQRACIGMQFALHEATLVLGMI

LQHFTFTDHTDYELDIKQTLTLKPNDFHIRVRPRSKEAVPAAFTAAEKAAEDVTKEKQET

KGASIIGLDNRPLLILYGSDTGTAEGVAWELADTAGMHGVRTETAPLNDRIGKLPKEGAL

LIITSSYNGKPPSNAGQFVQWLEEVKQGELEGVRYAVFGCGDHNWAATYQAVPRLIDEKL

AEKGAERFSSRGEGDVSGDFEGKLDEWKKSMWKDAMKAFGLKLNENAEKERSTLGLQFVS

GLGGSPLAQTYEAVYASVAENRELQAPESDRSTRHIEIILPKEAAYNEGDHLGVLPVNSK

EQVSRVLRRYNLNGNDQVLLTASGQSAAHLPLDRPVRLHDLLSSCVELQEAASRAQIREM

AAYTVCPPHKHELEGLLEEGVYQEQILTLRVSMLDLLEKYEACELPFERFLELLRPLKPR

YYSISSSPLKHPGQASITVGVVRGPARSGLGEYRGVASNYLADRSPDDGIVMFVRTPETK

FRLPEDPEKPIIMVGPGTGVAPFRGFLQARAALKGEGKELGEAHLYFGCRNDYDFIYRDE

LEAYEKDGIVTLHTAFSRKEGVPKTYVQHLMAEDAETLISILDRGGHLYVCGDGSKMAPD

VEETLQKAYQSAHGTDERQAQEWLLDLQTKGIYAKDVWAGI

>CYP102A49 *Bacillus amyloliquefaciens* TA208

MKQLSAIPQPKTYGPLKNLPHLEKEKLSQSLWKIAEEYGPIFRFEFPSSVGVFVSGRELA

AEVCDEKRFDKNLGKALLKVREFGGDGLFTSWTNEKNWQKAHRILLPSFSQKAMKGYHSM

MLDIAMQLVQKWSRLNPNEEIDVAEDMTRLTLDTIGLCGFHYRFNSFYRDTQHPFITSML

RALQEAMRQSQRHSLQDKLMIKTRHQFQQDIEEMNALVDRIIAERRENPDENLSDLLALM

LEAKDPVTGERLDDENIRYQIITFLIAGHETTSGLLSFAIYCLLKNKDKLEKAVQEAERV

LTGETPEYKQIQQLTYIRMVLNETLRLYPTAPAFSLYAKEDTVLGGKYPIEKGQPVTILT

PQLHRDKSAWGEDAELFRPERFSDPAAIPADAYKPFGNGQRACIGMQFALQEATMVLGLV

LKHFELIDHSDYELTIKEALTIKPGDFKIRVKNKDVSNRQTAQNHKAEGSDHKEETKEIP

SHGTPLLILYGSNLGTAEGIAEELADIGRSKGFSTETGPLDDYAGKLPVKGAVVIVTASY

NGAPPDNAAGFVKWMETLEDQELKGVSYAVFGCGDRNWAATYQKIPRLIDKVLEEKGATR

LTSIGEGDNADDFEYSQEAWEDSFWQDIIKAFHIEAAPEQQNKSQLSIEYVSEAAETPIA

KTYKAFEAEVITNKELHTESSTRSVRHIELRLPETETYQEGDHLGVLPQNSGELISRVIH

RFALDPNQHFKISGRHLPHLPMDRPVNALELLASYVELQEPATRAQLRELAAHTVCPPHQ

KELEHLYSDETAYKENVLKKRMTMLDLLEDYPACELPFERFLELLPSLKARYYSISSSPK

ANSGELSITVGVVTAPAWSGRGEYRGVASNYLAGLQNGDHAVCFIRSPQSGFALPENTKT

PLIMVGAGTGIAPFRGFIQARAAEKMSGNSLGEAHLYFGCRHPEEDDLYKDEFDQAEKTG

LVTVHRAYSRLDQDCKVYVQDVLLREAAQIIALLDQGGHLYICGDGSKMAPAVENVLLQA

YEKVHNTDSKVSLSWLEQLQAEGRYAKDVWAGV

>CYP107H2 *Bacillus amyloliquefaciens* TA208

MTAGLSIAHPSLHADSDFWNNPYPFYDKLRAVDPVYQGTVLKYPGRYITGYQEAEAVLKD

TRFKNRIPMPEASAKYKHLKNLQKDMLLFTNHSDHKRLRMLIGKAFTLKKAERLKPFITA

TVHDVLDQIDHSKTADLVSDFAFPVASLVIADILGVPKEDRASFREWTADVIQAIDLTRS

KKSLLKAGGTAGKLTAYFKDLIQKRKTEPQKDVITTLISEEQLTEEEVLASCILLIIAGH

ETTVNLICNGVFSLLKHPAELSKLLENPQLIASATEEFLRFESPAQLTARTASEDCVINQ

HLIKKGEQVYILLGAANRDPEVFHRPHQLDITRNPNPHLAFGKGAHVCIGSSLARIEAQT

AILTLLERAPDIRLVKTDVTYRKLFGFRSLSALPVVLS

>CYP107J6 *Bacillus amyloliquefaciens* TA208

MFSKQNESLTVLTESQLSSRAFKDEAYAFYKELRKSRPLCPLSLGSLGEGWLISRYDDAI

QLLKNEKLKKNYENVFTAKEEKRPVLFKNEGPLRKHMLNSDPPDHNRLRTLVQKAFTHRM

ILQLEDKIQHIADSLLDKVQPSKFMNLVDDYAFPLPIIVISEMLGIPLEDRQNFKIWSQA

IIDFSDAPERLQENDHLLGEFVEYLEYLVRKKRSEPAGDLISALIQAESEGTPLSTEELY

SMIMLLIVAGHETTVNLITNMTYALMCHHDQLEKLRRQPDLMNSAIEEALRFHSPVELTT

IRWTAEPFILHGQEIKRKDVIIISLASANRDEKIFPNADMFDIERKNNRHIAFGHGIHFC

LGAQLARLESKIAISTLLRRCPNIQIKGGKEQIKWKGNFLMRALEELPLTF

>CYP107K3 *Bacillus amyloliquefaciens* TA208

MEKIMFHPHSSEFHENPFAVLSRFREQDPIHKFELQRFGGTFPAWLITRYDDCMAFLKDG

RITRDVKRVMPKELIAKLNVSEDIDFVSEHMLAKDPPDHSRLRSLVHQGFTPRMIEQLRT

GIERITEELLDEMETKADPDIMRDFAAPLPFIVISELLGIPKEDRAKFQVWTSAMVDTSE

SGQDATNQALREFKQYMQTLIEEKRNHPGEDLTSKLIYAEEDGQKLSESELYSMLFLLVV

AGLETTVNLLGSGTLALLLHKDQLENIKRQPDTIQTAVEELLRYTSPVIMMANRWAIEDF

TYKDVSIKKGDMIFIGIGSANRDPEYFDEPDTLNVARTPNRHISFGFGIHFCLGAPLARM

EASIAFNALLKRFPNIELNGSSEDLTWRKNVFLRGLETLPVKF

>CYP109B3 *Bacillus amyloliquefaciens* TA208

MSNLRSPRQAIQRTLMKGKDGLDVYNPFPWYEKMRRESPIQFDEETKVWSVFLYDDAKKV

ISDKETFSSLMSDMKSSIAKSMLNMDPPKHTQIRSAVNRAFTPRVLKEWEPRIEEITDNL

LKQAKNKGRIDIVKDLSYPLPVIVISELLGVPSEHMDQFKKWSDILVSMPKDASPEEAEK

NQKERDQCETELAAFFAEIIDSKRKQPGQDIISILIKEEEEGEKLSAEDLIPFCNLLLVA

GNETTTNLISNAVYSILETPGLYEELRHDPSLIAQTVEETLRFRAPAPFVRRTVRHDTEL

CGRRLKSGDIVLCYIASANRDENKFEQADVFDIHRQSNPHLSFGFGVHFCLGAPLARLEA

EVALRGIVKAFSHLEPVRVEPIQNSVMYGLDSLEAEINENRGEEKR

>CYP152A4 *Bacillus amyloliquefaciens* TA208

MTEQIPHEKGLDNSLALLRDGYVFVKNRAENYRSDVFRARLLGKTFICMSGAEAAKLFYD

TERFQRQQALPKRVQKTLFGTGAIQSMDGERHKHRKLLFMSLMTPPRQKRLAEAVAKQWK

ASAEKWEGADRIVLFDEAKKVLCRAACEWAGVPLKDSEVKERAEDFTDMVDAFGAVGPRH

WKGRRARPKTEKWVEEVIEDVRSGKLQTPEGSALYEMAVHTELDGSRLDSHMAAVELINV

LRPIAAISYFIAFSALALHDHPEYRDKLRSGDDQEAERFVHEVRRYYPFAPFLGAVVKKD

FVWKNCEFKKGASVMLDLYGTNHDSRLWENPNEFRPERFQGREENKFDFIPQGGGDPADG

HRCPGEGMTVEVMKASLAFLTNEIEYDVPPQDLSFSLSRMPALPESGFVISDVRRK

>CYP102A38 *Bacillus amyloliquefaciens* XH7

MKETSPIPKPKTFGPLGNLPLLDKDKPTMSLIKLAKEQGPIFQLHTPAGTIIVVSGHELV

KEVCDEERFDKSIEGALEKVRAFSGDGLFTSWTHEPNWRKAHNILMPTFSKRAMKDYHSM

MTDIAVQLIQKWARLNPDEAVDVPADMTRLTLDTIGLCGFNYRFNSYYRETPHPFINSMV

RALDEAMHQMQRLDVQDKLMIRTKRQFHHDIQAMFSLVDSIIAERRSDGGRDEKDLLARM

LNVEDPETGEKLDDENIRFQIITFLIAGHETTSGLLSFAIYFLLKHPRVLEKAYEEADRV

LTDPVPSYKQVLELTYIRMILQESLRLWPTAPAFSLYAKEDTVIGGKYPITTKDRISVLI

PQLHRDKDAWGDNAEEFYPERFEHPDQVPHHAYKPFGNGQRACIGMQFALHEATLVLGMI

LQHFTFTDHTDYELDIKQTLTLKPNDFHIRVRPRSKEAVPAAFTAAEKAAEDVTKEKQET

KGASIIGLDNRPLLILYGSDTGTAEGVAWELADTAGMHGVRTETAPLNDRIGKLPKEGAL

LIITSSYNGKPPSNAGQFVQWLEEVKQGELEGVRYAVFGCGDHNWAATYQAVPRLIDEKL

AEKGAERFSSRGEGDVSGDFEGKLDEWKKSMWKDAMKAFGLKLNENAEKERSTLGLQFVS

GLGGSPLAQTYEAVYASVAENRELQAPESDRSTRHIEIILPKEAAYNEGDHLGVLPVNSK

EQVSRVLRRYNLNGNDQVLLTASGQSAAHLPLDRPVRLHDLLSSCVELQEAASRAQIREM

AAYTVCPPHKHELEGLLEEGVYQEQILTLRVSMLDLLEKYEACELPFERFLELLRPLKPR

YYSISSSPLKHPGQASITVGVVRGPARSGLGEYRGVASNYLADRSPDDGIVMFVRTPETK

FRLPEDPEKPIIMVGPGTGVAPFRGFLQARAALKGEGKELGEAHLYFGCRNDYDFIYRDE

LEAYEKDGIVTLHTAFSRKEGVPKTYVQHLMAEDAETLISILDRGGHLYVCGDGSKMAPD

VEETLQKAYQSAHGTDERQAQEWLLDLQTKGIYAKDVWAGI

>CYP102A49 *Bacillus amyloliquefaciens* XH7

MKQLSAIPQPKTYGPLKNLPHLEKEKLSQSLWKIAEEYGPIFRFEFPSSVGVFVSGRELA

AEVCDEKRFDKNLGKALLKVREFGGDGLFTSWTNEKNWQKAHRILLPSFSQKAMKGYHSM

MLDIAMQLVQKWSRLNPNEEIDVAEDMTRLTLDTIGLCGFHYRFNSFYRDTQHPFITSML

RALQEAMRQSQRHSLQDKLMIKTRHQFQQDIEEMNALVDRIIAERRENPDENLSDLLALM

LEAKDPVTGERLDDENIRYQIITFLIAGHETTSGLLSFAIYCLLKNKDKLEKAVQEAERV

LTGETPEYKQIQQLTYIRMVLNETLRLYPTAPAFSLYAKEDTVLGGKYPIEKGQPVTILT

PQLHRDKSAWGEDAELFRPERFSDPAAIPADAYKPFGNGQRACIGMQFALQEATMVLGLV

LKHFELIDHSDYELTIKEALTIKPGDFKIRVKNKDVSNRQTAQNHKAEGSDHKEETKEIP

SHGTPLLILYGSNLGTAEGIAEELADIGRSKGFSTETGPLDDYAGKLPVKGAVVIVTASY

NGAPPDNAAGFVKWMETLEDQELKGVSYAVFGCGDRNWAATYQKIPRLIDKVLEEKGATR

LTSIGEGDNADDFEYSQEAWEDSFWQDIIKAFHIEAAPEQQNKSQLSIEYVSEAAETPIA

KTYKAFEAEVITNKELHTESSTRSVRHIELRLPETETYQEGDHLGVLPQNSGELISRVIH

RFALDPNQHFKISGRHLPHLPMDRPVNALELLASYVELQEPATRAQLRELAAHTVCPPHQ

KELEHLYSDETAYKENVLKKRMTMLDLLEDYPACELPFERFLELLPSLKARYYSISSSPK

ANSGELSITVGVVTAPAWSGRGEYRGVASNYLAGLQNGDHAVCFIRSPQSGFALPENTKT

PLIMVGAGTGIAPFRGFIQARAAEKMSGNSLGEAHLYFGCRHPEEDDLYKDEFDQAEKTG

LVTVHRAYSRLDQDCKVYVQDVLLREAAQIIALLDQGGHLYICGDGSKMAPAVENVLLQA

YEKVHNTDSKVSLSWLEQLQAEGRYAKDVWAGV

>CYP107H2 *Bacillus amyloliquefaciens* XH7

MTAGLSIAHPSLHADSDFWNNPYPFYDKLRAVDPVYQGTVLKYPGRYITGYQEAEAVLKD

TRFKNRIPMPEASAKYKHLKNLQKDMLLFTNHSDHKRLRMLIGKAFTLKKAERLKPFITA

TVHDVLDQIDHSKTADLVSDFAFPVASLVIADILGVPKEDRASFREWTADVIQAIDLTRS

KKSLLKAGGTAGKLTAYFKDLIQKRKTEPQKDVITTLISEEQLTEEEVLASCILLIIAGH

ETTVNLICNGVFSLLKHPAELSKLLENPQLIASATEEFLRFESPAQLTARTASEDCVINQ

HLIKKGEQVYILLGAANRDPEVFHRPHQLDITRNPNPHLAFGKGAHVCIGSSLARIEAQT

AILTLLERAPDIRLVKTDVTYRKLFGFRSLSALPVVLS

>CYP107J6 *Bacillus amyloliquefaciens* XH7

MFSKQNESLTVLTESQLSSRAFKDEAYAFYKELRKSRPLCPLSLGSLGEGWLISRYDDAI

QLLKNEKLKKNYENVFTAKEEKRPVLFKNEGPLRKHMLNSDPPDHNRLRTLVQKAFTHRM

ILQLEDKIQHIADSLLDKVQPSKFMNLVDDYAFPLPIIVISEMLGIPLEDRQNFKIWSQA

IIDFSDAPERLQENDHLLGEFVEYLEYLVRKKRSEPAGDLISALIQAESEGTPLSTEELY

SMIMLLIVAGHETTVNLITNMTYALMCHHDQLEKLRRQPDLMNSAIEEALRFHSPVELTT

IRWTAEPFILHGQEIKRKDVIIISLASANRDEKIFPNADMFDIERKNNRHIAFGHGIHFC

LGAQLARLESKIAISTLLRRCPNIQIKGGKEQIKWKGNFLMRALEELPLTF

>CYP107K3 *Bacillus amyloliquefaciens* XH7

MEKIMFHPHSSEFHENPFAVLSRFREQDPIHKFELQRFGGTFPAWLITRYDDCMAFLKDG

RITRDVKRVMPKELIAKLNVSEDIDFVSEHMLAKDPPDHSRLRSLVHQGFTPRMIEQLRT

GIERITEELLDEMETKADPDIMRDFAAPLPFIVISELLGIPKEDRAKFQVWTSAMVDTSE

SGQDATNQALREFKQYMQTLIEEKRNHPGEDLTSKLIYAEEDGQKLSESELYSMLFLLVV

AGLETTVNLLGSGTLALLLHKDQLENIKRQPDTIQTAGEELLRYTSPVIMMANRWAIEDF

TYKDVSIKKGDMIFIGIGSANRDPEYFDEPDTLNVARTPNRHISFGFGIHFCLGAPLARM

EASIAFNALLKRFPNIELNGSSEDLTWRKNVFLRGLETLPVKF

>CYP109B3 *Bacillus amyloliquefaciens* XH7

MSNLRSPRQAIQRTLMKGKDGLDVYNPFPWYEKMRRESPIQFDEETKVWSVFLYDDAKKV

ISDKETFSSLMSDMKSSIAKSMLNMDPPKHTQIRSAVNRAFTPRVLKEWEPRIEEITDNL

LKQAKNKGRIDIVKDLSYPLPVIVISELLGVPSEHMDQFKKWSDILVSMPKDASPEEAEK

NQKERDQCETELAAFFAEIIDSKRKQPGQDIISILIKEEEEGEKLSAEDLIPFCNLLLVA

GNETTTNLISNAVYSILETPGLYEELRHDPSLIAQTVEETLRFRAPAPFVRRTVRHDTEL

CGRRLKSGDIVLCYIASANRDENKFEQADVFDIHRQSNPHLSFGFGVHFCLGAPLARLEA

EVALRGIVKAFSHLEPVRVEPIQNSVMYGLDSLEAEINENRGEEKR

>CYP152A4 *Bacillus amyloliquefaciens* XH7

MTEQIPHEKGLDNSLALLRDGYVFVKNRAENYRSDVFRARLLGKTFICMSGAEAAKLFYD

TERFQRQQALPKRVQKTLFGTGAIQSMDGERHKHRKLLFMSLMTPPRQKRLAEAVAKQWK

ASAEKWEGADRIVLFDEAKKVLCRAACEWAGVPLKDSEVKERAEDFTDMVDAFGAVGPRH

WKGRRARPKTEKWVEEVIEDVRSGKLQTPEGSALYEMAVHTELDGSRLDSHMAAVELINV

LRPIAAISYFIAFSALALHDHPEYRDKLRSGDDQEAERFVHEVRRYYPFAPFLGAVVKKD

FVWKNCEFKKGASVMLDLYGTNHDSRLWENPNEFRPERFQGREENKFDFIPQGGGDPADG

HRCPGEGMTVEVMKASLAFLTNEIEYDVPPQDLSFSLSRMPALPESGFVISDVRRK

>CYP102A38 *Bacillus amyloliquefaciens* Y2

MLERVLFMKETGPIPQPKTFGPLGNLPLLDKDKPTMSLIKLANEQGPIFQLHTPAGAIIV

VSGHELVKEVCDEERFDKSIEGALEKVRAFSGDGLFTSWTHEPNWRKAHNILMPTFSQRA

MKDYHSMMTDIAVQLIQKWARLNPDEAVDVPADMTRLTLDTIGLCGFNYRFNSYYRETPH

PFINSMVRALDEAMHQMQRLDVQDKLMIRTKRQFHHDIQAMFSLVDSIIAERRSGGRDEK

DLLARMLNVEDPETGEKLDDENIRFQIITFLIAGHETTSGLLSFAIYFLLKHPRVLEKAY

EEADRVLTDPVPSYKQVLDLTYIRMILQESLRLWPTAPAFSLYAKEDTVIGGKYPITPKD

RISVLIPQLHRDKDAWGDNAEEFYPERFEHPDQVPHHAYKPFGNGQRACIGMQFALHEAT

LVLGMILQHFTFIDHTDYELDIKQTLTIKPGDFHIRVRPRNKGAVAAALPAAEKAAEDVE

KEKRETKGASIIGLDNRPLLILYGSDTGTAEGVARELADTAGMHGVRTETAPLNDRIGKL

PKEGALLIITSSYNGKPPSNAGQFVQWLEEVKPGELEGVRYAVFGCGDHNWAATYQAVPR

LIDEKLAEKGAERFSSRGEGDVSGDFEGKLDEWKKSMWTDAMKAFGLKLNENAEKERSSL

GLQFVSGLGGSPLAQTYEAVYASVAENRELQAPESGRSTRHIEITLPKEAAYHEGDHLGV

LPVNSKEQVSRVLRRFNLNGNDQVLLTASGQSAAHLPLDRPVRLHDLLSSCVELQEAASR

AQIREMAAYTVCPPHKRELEDFLEEGVYQEKILTLRVSMLDLLEKYEACELPFERFLELL

RPLKPRYYSISSSPRKHPGQASITVGVVRGPARSGLGEYRGVASNYLADRGPEDGIVMFV

RTPETRFRLPEDPEKPIIMVGPGTGVAPFRGFLQARAALKKEGKELGEAHLYFGCRNDHD

FIYRDELEAYEKDGIVTLHTAFSRKEGVPKTYVQHLMAKDAGALISILGRGGHLYVCGDG

SKMAPDVEATLQKAYQSVHETDERQAQEWLLDLQTKGIYAKDVWAGI

>CYP102A49 *Bacillus amyloliquefaciens* Y2

MKQLSAIPQPKTYGPLKNLPHLEKEKLSQSLWKIAEEYGPIFRFEFPSSVGVFVSGRELA

AEVCDEKRFDKNLSKALLKVREFGGDGLFTSWTHEKNWQKAHRILLPSFSQKAMKGYHSM

MLDIAMQLVQKWSRLNPNEEIDVAEDMTRLTLDTIGLCGFHYRFNSFYRDTQHSFITSML

RALQEAMRQAQRHSLQDKLMIKTRHQFQQDIEEMNSLVDRIIAERRENPDENLSDLLALM

LEAKDPVTGERLDDENIRYQIITFLIAGHETTSGLLSFAIYCLLKNKDKLKKAVQEAERV

LTGETPEYKQIQQLTYIRMVLNETLRLYPTAPAFSLYAKEDTVLGGKYPIAKGQPVTILT

PQLHRDKSAWGEDAESFRPERFSDPAAIPADAYKPFGNGQRACIGMQFALQEATMVLGLV

LKHFELIDHTDYELTIKEALTIKPGDFKIRVKNKDVSNQQSAQNHKAEGSGHKEEMKEIP

SHGTPLLILYGSNLGTAEGIAEELADIGRSKGFSTETGPLDDYAGKLPVKGAVVIVTASY

NGAPPDNAAGFVKWMETLEDQELKGVSYAVFGCGDRNWAATYQRIPRLIDKVLEEKGAKR

LTSIGEGDNADDFEYSQEAWEDSFWKDIMKAFHIEAAPKQTNKSQLSIEYVSEAAETPIA

KTYKAFEAEVITNKELHTESSKRSVRHIELRLPETETYQEGDHLGVLPQNSGELISRVIR

RFGLDPNQHFKIKGRQLPHLPMDRPVNAPELLASYVELQEPATRAQLRELAAHTVCPPHQ

KELEHLYSDDAAYKENVLKKRMTMLDLLEDYPACELPFERFLELLPSLKARYYSISSSPK

AASGELSITVGVVTAPAWSGRGEYRGVASNYLAGLQNGDPAVCFIRSPQSGFALPENPKT

PLIMVGAGTGIAPFRGFIQARAAEKMSGNSLGEAHLYFGCRHPEEDDLYKDEFDDAEKNG

LVTVHRAYSRLDQDCKVYVQDVLLREAAQIIALLDQGGHLYICGDGSKMAPAVENVLLQA

YEKVHNTDSKVSLNWLEQLQAEGRYAKDVWAGM

>CYP107H4 *Bacillus amyloliquefaciens* Y2

MTAGLSIAHPSLHADSDFWNDPYSFYDKLRSIDPVYKGTVLKYPGWYVTGYKEAAAILKD

TRFKNRIPLPEASTKYQNLSHIQHDMLLFKNQSDHKRMRMLIGKEFTAKTAESLRPCIKE

TVHDLLDQVQIKKNADLVSEFAFPLASLIIAEILGVPKEERYQFRQWTADVIQAIDLTRS

RKTLVRASDTAGRLTSYFRDLIHKRKAHPQQDLISRFIMEEQLSKEEVLATCILLVIAGH

ETTVNLMCNGVFTLLKHPEQLSALRENPSLIETAVEECLRYDSPAQLTARTASEDCEING

KTIKKGEQVYILLGAANRDPSIFDQPHKMDIQRKPNPHLAFGKNAHFCIGSSLARIEAQI

AILTLFERMPKLRLAAHRLEYRKLIGFRSLKELPVVIG

>CYP107K3 *Bacillus amyloliquefaciens* Y2

MEKTMFHPHSPEFHENPFAVLSRFREQDPIHKFELQRFGGTFPAWLITRYDDCMAFLKDG

RITRDVKRVMPKELIAKLNVSEDIDFVSEHMLAKDPPDHSRLRSLVHQGFTPRMIEQLRT

GIEQITEELLDEMETKADPDIMRDFAAPLPFIVISELLGIPKEDRAKFQVWTNAMVDTSE

SGQDATNQALKEFKQYMKTLIEEKRKHPAEDLTSKLIYAEEDGQKLSESELYSMLFLLVV

AGLETTVNLLGSGTLALLLHKDQMEKIKRQPENIQTAVEELLRYTSPVIMMANRWAIEDF

TYKDVSIKKGDMIFIGIGSANRDPEYFDDSDTLNIARTPNRHISFGFGIHFCLGAPLARM

EASIAFTALLKRFPNIELKGAPEDVTWRKNVFLRGLETLPVRF

>CYP109B7 *Bacillus amyloliquefaciens* Y2

MKGKNGLDVYNPFPWYEKMRRESPIHFDEETKVWSVFLYDDVKKVISDKETFSSLMTDVK

SSIAKSMLNMDPPKHTQIRSAVNRAFTPRVLKEWEPRIKDITDHLLKQAKNKGRIDIVKD

LSYPLPVMVISELLGVPSEHMDQFKKWSDILVSMPKDASPEAAEKNQQERDQCEAELAAF

FAEIIESKRKKPGQDIISILIKEEEEGEKLTAEDLIPFCNLLLVAGNETTTNLISNAVYS

ILETPGLYEELRQDPSLIAQTVEETLRFRAPAPFVRRTVRHDTELRGRRLKSGEIVLCYV

ASANRDENKFEKAGVFDIHRQSNPHLSFGFGVHFCLGAPLARLEAEAALKGIVKTFSHLE

PVGIEPIRNSVMYGLESLEAEINESEGEEKR

>CYP113L1 *Bacillus amyloliquefaciens* Y2

MKMTSLTKIRQQQPYKWYQTMRETSPVHYNEKEDCWEIFTYDEVKRVISDYSHFSSDHKY

LSADKQEKMIRHINKDSLLKMDPPEHTVFRKLVNQPFMPKSVESLAPRIAAIADDLLQAV

RSKGRMDIIEDYAFPLPIIVIAELLGFPPKDRDIFKSWVDQSQNVKDEKKMNEVQKQMIG

YFMQFILQRRKQPQNDLISHLISADLDGEPLSDKQLIGFCGLLIVAGHVTTENVIGNSFL

SLKEFPHILPRLLENKALLPDFIEEVIRLRPSIQRVTRYTAVESEIGGKTIPAGEKVYAW

IGSANRDEKKFENADQIDLGRKPNQHLSFGQGSHYCLGAPLARLEAKIALSHFFEQMPAW

RFTEDQEPNLVPSPVFHGVDRLLVEF

>CYP152A4 *Bacillus amyloliquefaciens* Y2

MDEQIPHEKGLDNSLALLRDGYVFVKNRAETYRSDVFRARLLGKTFICMSGAEAAKLFYD

TERFQRQQALPKRVQKTLFGTGAIQAMDGERHKHRKLLFMSLMTPPRQKRLAEAVAKQWK

ASAEMWKGSNRIVLFDEAKKVLCRAACEWAGVPLKDSEVKERAEDFTDMVDAFGAVGPRH

WKGRRARLKTEKWIEEVIEDVRSGKLQTPEGSALYEMAFHTELDGNRLDSHMAAVELINV

LRPIAAISYFITFSALALHDYPEYRGKLRSRDDQEAERFAHEVRRYYPFAPFLGAVVKKD

FVWKNCEFKKGASVMLDLYGTNHDSRLWEDPNEFRPERFQGREENKFDFIPQGGGDPANG

HRCPGEGMTVEVMKTSLAFLANEIEYDVPSQDLSFSLSRMPALPESGFVISDVRRI

>CYP102A38 *Bacillus sp.* BH072

MKETGPIPQPKTFGPLGNLPLLDKDKPTMSLIKLANEQGPIFQLHTPAGAIIVVSGHELV

KEVCDEERFDKSIEGALEKVRAFSGDGLFTSWTHEPNWRKAHNILMPTFSQRAMKDYHSM

MTDIAVQLIQKWARLNPDEAVDVPADMTRLTLDTIGLCGFNYRFNSYYRETPHPFINSMV

RALDEAMHQMQRLDVQDKLMIRTKRQFHHDIQAMFSLVDSIIAERRSGGRDEKDLLARML

NVEDPETGEKLDDENIRFQIITFLIAGHETTSGLLSFAIYFLLKHPRVLEKAYEEADRVL

TDPVPSYKQVLDLTYIRMILQESLRLWPTAPAFSLYAKEDTVIGGKYPITPKDRISVLIP

QLHRDKDAWGDNAEEFYPERFEHPDQVPHHAYKPFGNGQRACIGMQFALHEATLVLGMIL

QHFTFIDHTDYELDIKQTLTIKPGDFHIRVRPRNKGAVAAALPAAEKAAEDVEKEKRETK

GASIIGLDNRPLLILYGSDTGTAEGVARELADTAGMHGVRTETAPLNDRIGKLPKEGALL

IITSSYNGKPPSNAGQFVQWLEEVKPGELEGVRYAVFGCGDHNWAATYQAVPRLIDEKLA

EKGAERFSSRGEGDVSGDFEGKLDEWKKSMWTDAMKAFGLKLNENAEKERSSLGLQFVSG

LGGSPLAQTYEAVYASVAENRELQAPESGRSTRHIEITLPKEAAYHEGDHLGVLPVNSKE

QVSRVLRRFNLNGNDQVLLTASGQSAAHLPLDRPVRLHDLLSSCVELQEAASRAQIREMA

AYTVCPPHKRELEDFLEEGVYQEKILTLRVSMLDLLEKYEACELPFERFLELLRPLKPRY

YSISSSPRKHPGQASITVGVVRGPARSGLGEYRGVASNYLADRGPEDGIVMFVRTPETRF

RLPEDPEKPIIMVGPGTGVAPFRGFLQARAALKKEGKELGEAHLYFGCRNDHDFIYRDEL

EAYEKDGIVTLHTAFSRKEGVPKTYVQHLMAKDAGALISILGRGGHLYVCGDGSKMAPDV

EATLQKAYQSVHETDERQAQEWLLDLQTKGIYAKDVWAGI

>CYP102A49 *Bacillus sp*. BH072

MKQLSAIPQPKTYGPLKNLPHLEKEKLSQSLWKIAEEYGPIFRFEFPSSVGVFVSGRELA

AEVCDEKRFDKNLSKALLKVREFGGDGLFTSWTHEKNWQKAHRILLPSFSQKAMKGYHSM

MLDIAMQLVQKWSRLNPNEEIDVAEDMTRLTLDTIGLCGFHYRFNSFYRDTQHSFITSML

RALQEAMRQAQRHSLQDKLMIKTRHQFQQDIEEMNSLVDRIIAERRENPDENLSDLLALM

LEAKDPVTGERLDDENIRYQIITFLIAGHETTSGLLSFAIYCLLKNKDKLKKAVQEAERV

LTGETPEYKQIQQLTYIRMVLNETLRLYPTAPAFSLYAKEDTVLGGKYPIAKGQPVTILT

PQLHRDKSAWGEDAESFRPERFSDPAAIPADAYKPFGNGQRACIGMQFALQEATMVLGLV

LKHFELIDHTDYELTIKEALTIKPGDFKIRVKNKDVSNQQSAQNHKAEGSGHKEEMKEIP

SHGTPLLILYGSNLGTAEGIAEELADIGRSKGFSTETGPLDDYAGKLPVKGAVVIVTASY

NGAPPDNAAGFVKWMETLEDQELKGVSYAVFGCGDRNWAATYQRIPRLIDKVLEEKGAKR

LTSIGEGDNADDFEYSQEAWEDSFWKDIMKAFHIEAAPKQTNKSQLSIEYVSEAAETPIA

KTYKAFEAEVITNKELHTESSKRSVRHIELRLPETETYQEGDHLGVLPQNSGELISRVIR

RFGLDPNQHFKIKGRQLPHLPMDRPVNAPELLASYVELQEPATRAQLRELAAHTVCPPHQ

KELEHLYSDDAAYKENVLKKRMTMLDLLEDYPACELPFERFLELLPSLKARYYSISSSPK

AASGELSITVGVVTAPAWSGRGEYRGVASNYLAGLQNGDPAVCFIRSPQSGFALPENPKT

PLIMVGAGTGIAPFRGFIQARAAEKMSGNSLGEAHLYFGCRHPEEDDLYKDEFDDAEKNG

LVTVHRAYSRLDQDCKVYVQDVLLREAAQIIALLDQGGHLYICGDGSKMAPAVENVLLQA

YEKVHNTDSKVSLNWLEQLQAEGRYAKDVWAGM

>CYP107H4 *Bacillus sp*. BH072

MTAGLSIAHPSLHADSDFWNDPYSFYDKLRSIDPVYKGTVLKYPGWYVTGYKEAAAILKD

TRFKNRIPLPEASTKYQNLSHIQHDMLLFKNQSDHKRMRMLIGKEFTAKTAESLRPCIKE

TVHDLLDQVQIKKNADLVSEFAFPLASLIIAEILGVPKEERYQFRQWTADVIQAIDLTRS

RKTLVRASDTAGRLTSYFRDLIHKRKAHPQQDLISRFIMEEQLSKEEVLATCILLVIAGH

ETTVNLMCNGVFTLLKHPEQLSALRENPSLIETAVEECLRYDSPAQLTARTASEDCEING

KTIKKGEQVYILLGAANRDPSIFDQPHKMDIQRKPNPHLAFGKNAHFCIGSSLARIEAQI

AILTLFERMPKLRLAAHRLEYRKLIGFRSLKELPVVIG

>CYP107K3 *Bacillus sp.* BH072

MEKTMFHPHSPEFHENPFAVLSRFREQDPIHKFELQRFGGTFPAWLITRYDDCMAFLKDG

RITRDVKRVMPKELIAKLNVSEDIDFVSEHMLAKDPPDHSRLRSLVHQGFTPRMIEQLRT

GIEQITEELLDEMETKADPDIMRDFAAPLPFIVISELLGIPKEDRAKFQVWTNAMVDTSE

SGQDATNQALKEFKQYMKTLIEEKRKHPAEDLTSKLIYAEEDGQKLSESELYSMLFLLVV

AGLETTVNLLGSGTLALLLHKDQMEKIKRQPENIQTAVEELLRYTSPVIMMANRWAIEDF

TYKDVSIKKGDMIFIGIGSANRDPEYFDDSDTLNIARTPNRHISFGFGIHFCLGAPLARM

EASIAFTALLKRFPNIELKGAPEDVTWRKNVFLRGLETLPVRF

>CYP109B7 *Bacillus sp*. BH072

MSKLRSTRQAIQRTLMKGKNGLDVYNPFPWYEKMRRESPIHFDEETKVWSVFLYDDVKKV

ISDKETFSSLMTDVKSSIAKSMLNMDPPKHTQIRSAVNRAFTPRVLKEWEPRIKDITDHL

LKQAKNKGRIDIVKDLSYPLPVMVISELLGVPSEHMDQFKKWSDILVSMPKDASPEAAEK

NQQERDQCEAELAAFFAEIIESKRKKPGQDIISILIKEEEEGEKLTAEDLIPFCNLLLVA

GNETTTNLISNAVYSILETPGLYEELRQDPSLIAQTVEETLRFRAPAPFVRRTVRHDTEL

RGRRLKSGEIVLCYVASANRDENKFEKAGVFDIHRQSNPHLSFGFGVHFCLGAPLARLEA

EAALKGIVKTFSHLEPVGIEPIRNSVMYGLESLEAEINESEGEEKR

>CYP113L1 *Bacillus sp*. BH072

MTSLTKIRQQQPYKWYQTMRETSPVHYNEKEDCWEIFTYDEVKRVISDYSHFSSDHKYLS

ADKQEKMIRHINKDSLLKMDPPEHTVFRKLVNQPFMPKSVESLAPRIAAIADDLLQAVRS

KGRMDIIEDYAFPLPIIVIAELLGFPPKDRDIFKSWVDQSQNVKDEKKMNEVQKQMIGYF

MQFILQRRKQPQNDLISHLISADLDGEPLSDKQLIGFCGLLIVAGHVTTENVIGNSFLSL

KEFPHILPRLLENKALLPDFIEEVIRLRPSIQRVTRYTAVESEIGGKTIPAGEKVYAWIG

SANRDEKKFENADQIDLGRKPNQHLSFGQGSHYCLGAPLARLEAKIALSHFFEQMPAWRF

TEDQEPNLVPSPVFHGVDRLLVEF

>CYP152A4 *Bacillus sp*. BH072

MDEQIPHEKGLDNSLALLRDGYVFVKNRAETYRSDVFRARLLGKTFICMSGAEAAKLFYD

TERFQRQQALPKRVQKTLFGTGAIQAMDGERHKHRKLLFMSLMTPPRQKRLAEAVAKQWK

ASAEMWKGSNRIVLFDEAKKVLCRAACEWAGVPLKDSEVKERAEDFTDMVDAFGAVGPRH

WKGRRARLKTEKWIEEVIEDVRSGKLQTPEGSALYEMAFHTELDGNRLDSHMAAVELINV

LRPIAAISYFITFSALALHDYPEYRGKLRSRDDQEAERFAHEVRRYYPFAPFLGAVVKKD

FVWKNCEFKKGASVMLDLYGTNHDSRLWEDPNEFRPERFQGREENKFDFIPQGGGDPANG

HRCPGEGMTVEVMKTSLAFLANEIEYDVPSQDLSFSLSRMPALPESGFVISDVRRI

>CYP102A36 *Bacillus sp*. BS34A

MKETSPIPQPKTFGPLGNLPLIDKDKPTLSLIKLAEEQGPIFQIHTPAGTTIVVSGHELV

KEVCDEERFDKSIEGALEKVRAFSGDGLFTSWTHEPNWRKAHNILMPTFSQRAMKDYHEK

MVDIAVQLIQKWARLNPNEAVDVPGDMTRLTLDTIGLCGFNYRFNSYYRETPHPFINSMV

RALDEAMHQMQRLDVQDKLMVRTKRQFRYDIQTMFSLVDSIIAERRANGDQDEKDLLARM

LNVEDPETGEKLDDENIRFQIITFLIAGHETTSGLLSFATYFLLKHPDKLKKAYEEVDRV

LTDAAPTYKQVLELTYIRMILNESLRLWPTAPAFSLYPKEDTVIGGKFPITTNDRISVLI

PQLHRDRDAWGKDAEEFRPERFEHQDQVPHHAYKPFGNGQRACIGMQFALHEATLVLGMI

LKYFTLIDHENYELDIKQTLTLKPGDFHISVQSRHQEAIHADVQAAEKAAPDEQKEKTEA

KGASVIGLNNRPLLVLYGSDTGTAEGVARELADTASLHGVRTKTAPLNDRIGKLPKEGAV

VIVTSSYNGKPPSNAGQFVQWLQEIKPGELEGVHYAVFGCGDHNWASTYQYVPRFIDEQL

AEKGATRFSARGEGDVSGDFEGQLDEWKKSMWADAIKAFGLELNENADKERSTLSLQFVR

GLGESPLARSYEASHASIAENRELQSADSDRSTRHIEIALPPDVEYQEGDHLGVLPKNSQ

TNVSRILHRFGLKGTDQVTLSASGRSAGHLPLGRPVSLHDLLSYSVEVQEAATRAQIREL

ASFTVCPPHRRELEELSAEGVYQEQILKKRISMLDLLEKYEACDMPFERFLELLRPLKPR

YYSISSSPRVNPRQASITVGVVRGPAWSGRGEYRGVASNDLAERQAGDDVVMFIRTPESR

FQLPKDPETPIIMVGPGTGVAPFRGFLQARDVLKREGKTLGEAHLYFGCRNDRDFIYRDE

LERFEKDGIVTVHTAFSRKEGMPKTYVQHLMADQADTLISILDRGGRLYVCGDGSKMAPD

VEAALQKAYQAVHGTGEQEAQNWLRHLQDTGMYAKDVWAGI

>CYP102A48 *Bacillus sp*. BS34A

MKQASAIPQPKTYGPLKNLPHLEKEQLSQSLWRIADELGPIFRFDFPGVSSVFVSGHNLV

AEVCDEKRFDKNLGKGLQKVREFGGDGLFTSWTHEPNWQKAHRILLPSFSQKAMKGYHSM

MLDIATQLIQKWSRLNPNEEIDVADDMTRLTLDTIGLCGFNYRFNSFYRDSQHPFITSML

RALKEAMNQSKRLGLQDKMMVKTKLQFQKDIEVMNSLVDRMIAERKANPDENIKDLLSLM

LYAKDPVTGETLDDENIRYQIITFLIAGHETTSGLLSFAIYCLLTHPEKLKKAQEEADRV

LTDDTPEYKQIQQLKYIRMVLNETLRLYPTAPAFSLYAKEDTVLGGEYPISKGQPVTVLI

PKLHRDQNAWGPDAEDFRPERFEDPSSIPHHAYKPFGNGQRACIGMQFALQEATMVLGLV

LKHFELINHTGYELKIKEALTIKPDDFKITVKPRKTAAINVQRKEQADIKAETKPKETKP

KHGTPLLVLFGSNLGTAEGIAGELAAQGRQMGFTAETAPLDDYIGKLPEEGAVVIVTASY

NGAPPDNAAGFVEWLKELEEGQLKGVSYAVFGCGNRSWASTYQRIPRLIDDMMKAKGASR

LTAIGEGDAADDFESHRESWENRFWKETMDAFDINEIAQKEDRPSLSITFLSEATETPVA

KAYGAFEGIVLENRELQTAASTRSTRHIELEIPAGKTYKEGDHIGILPKNSRELVQRVLS

RFGLQSNHVIKVSGSAHMAHLPMDRPIKVVDLLSSYVELQEPASRLQLRELASYTVCPPH

QKELEQLVSDDGIYKEQVLAKRLTMLDFLEDYPACEMPFERFLALLPSLKPRYYSISSSP

KVHANIVSMTVGVVKASAWSGRGEYRGVASNYLAELNTGDAAACFIRTPQSGFQMPNDPE

TPMIMVGPGTGIAPFRGFIQARSVLKKEGSTLGEALLYFGCRRPDHDDLYREELDQAEQD

GLVTIRRCYSRVENEPKGYVQHLLKQDTQKLMTLIEKGAHIYVCGDGSQMAPDVERTLRL

AYEAEKAASQEESAVWLQKLQDQRRYVKDVWTGM

>CYP107H1 *Bacillus sp*. BS34A

MTIASSTASSEFLKNPYSFYDTLRAVHPIYKGSFLKYPGWYVTGYEETAAILKDARFKVR

TPLPESSTKYQDLSHVQNQMMLFQNQPDHRRLRTLASGAFTPRTTESYQPYIIETVHHLL

DQVQGKKKMEVISDFAFPLASFVIANIIGVPEEDREQLKEWAASLIQTIDFTRSRKALTE

GNIMAVQAMAYFKELIQKRKRHPQQDMISMLLKGREKDKLTEEEAASTCILLAIAGHETT

VNLISNSVLCLLQHPEQLLKLRENPDLIGTAVEECLRYESPTQMTARVASEDIDICGVTI

RQGEQVYLLLGAANRDPSIFTNPDVFDITRSPNPHLSFGHGHHVCLGSSLARLEAQIAIN

TLLQRMPSLNLADFEWRYRPLFGFRALEELPVTFE

>CYP107J1 *Bacillus sp*. BS34A

MSSKEKKSVTILTESQLSSRAFKDEAYEFYKELRKSQALYPLSLGALGKGWLISRYDDAI

HLLKNEKLKKNYENVFTAKEKRPALLKNEETLTKHMLNSDPPDHNRLRTLVQKAFTHRMI

LQLEDKIQHIADSLLDKVQPNKFMNLVDDYAFPLPIIVISEMLGIPLEDRQKFRVWSQAI

IDFSDAPERLQENDHLLGEFVEYLESLVRKKRREPAGDLISALIQAESEGTQLSTEELYS

MIMLLIVAGHETTVNLITNMTYALMCHHDQLEKLRQQPDLMNSAIEEALRFHSPVELTTI

RWTAEPFILHGQEIKRKDVIIISLASANRDEKIFPNADIFDIERKNNRHIAFGHGNHFCL

GAQLARLEAKIAISTLLRRCPNIQLKGEKKQMKWKGNFLMRALEELPISF

>CYP107K1 *Bacillus sp.* BS34A

MEKLMFHPHGKEFHHNPFSVLGRFREEEPIHRFELKRFGATYPAWLITRYDDCMAFLKDN

RITRDVKNVMNQEQIKMLNVSEDIDFVSDHMLAKDTPDHTRLRSLVHQAFTPRTIENLRG

SIEQIAEQLLDEMEKENKADIMKSFASPLPFIVISELMGIPKEDRSQFQIWTNAMVDTSE

GNRELTNQALREFKDYIAKLIHDRRIKPKDDLISKLVHAEENGSKLSEKELYSMLFLLVV

AGLETTVNLLGSGTLALLQHKKECEKLKQQPEMIATAVEELLRYTSPVVMMANRWAIEDF

TYKGHSIKRGDMIFIGIGSANRDPNFFENPEILNINRSPNRHISFGFGIHFCLGAPLARL

EGHIAFKALLKRFPDIELAVAPDDIQWRKNVFLRGLESLPVSLSK

>CYP109B1 *Bacillus sp*. BS34A

MNVLNRRQALQRALLNGKNKQDAYHPFPWYESMRKDAPVSFDEENQVWSVFLYDDVKKVV

GDKELFSSCMPQQTSSIGNSIINMDPPKHTKIRSVVNKAFTPRVMKQWEPRIQEITDELI

QKFQGRSEFDLVHDFSYPLPVIVISELLGVPSAHMEQFKAWSDLLVSTPKDKSEEAEKAF

LEERDKCEEELAAFFAGIIEEKRNKPEQDIISILVEAEETGEKLSGEELIPFCTLLLVAG

NETTTNLISNAMYSILETPGVYEELRSHPELMPQAVEEALRFRAPAPVLRRIAKRDTEIG

GHLIKEGDMVLAFVASANRDEAKFDRPHMFDIRRHPNPHIAFGHGIHFCLGAPLARLEAN

IALTSLISAFPHMECVSITPIENSVIYGLKSFRVKM

>CYP134A1 *Bacillus sp*. BS34A

MSQSIKLFSVLSDQFQNNPYAYFSQLREEDPVHYEESIDSYFISRYHDVRYILQHPDIFT

TKSLVERAEPVMRGPVLAQMHGKEHSAKRRIVVRSFIGDALDHLSPLIKQNAENLLAPYL

ERGKSDLVNDFGKTFAVCVTMDMLGLDKRDHEKISEWHSGVADFITSISQSPEARAHSLW

CSEQLSQYLMPVIKERRVNPGSDLISILCTSEYEGMALSDKDILALILNVLLAATEPADK

TLALMIYHLLNNPEQMNDVLADRSLVPRAIAETLRYKPPVQLIPRQLSQDTVVGGMEIKK

DTIVFCMIGAANRDPEAFEQPDVFNIHREDLGIKSAFSGAARHLAFGSGIHNCVGAAFAK

NEIEIVANIVLDKMRNIRLEEDFCYAESGLYTRGPVSLLVAFDGA

>CYP102A36 *Bacillus sp*. YP1

MKETSPIPQPKTFGPLGNLPLIDKDKPTLSLIKLAEEQGPIFQIHTPAGTTIVVSGHELV

KEVCDEERFDKSIEGALEKVRAFSGDGLFTSWTHEPNWRKAHNILMPTFSQRAMKDYHEK

MVDIAVQLIQKWARLNPNEAVDVPGDMTRLTLDTIGLCGFNYRFNSYYRETPHPFINSMV

RALDEAMHQMQRLDVQDKLMVRTKRQFRHDIQTMFSLVDSIIAERRANGDQDEKDLLARM

LNVEDPETGEKLDDENIRFQIITFLIAGHETTSGLLSFATYFLLKHPDKLKKAYEEVDRV

LTDAAPTYKQVLELKYIRMILNESLRLWPTAPAFSLYPKEDTVIGGKFPITTKDRISVLI

PQLHRDRDAWGKDAEEFRPERFEHQDQVPHHAYKPFGNGQRACIGMQFALHEATLVLGMI

LKYFTLIDHENYELDIKQTLTLKPGDFHIRVQSRHQEAIHADAQADEKAAPYEQKEKTEA

KGASVIGLNNRPLLVLYGSDTGTAEGVARELADTASLHGVRTETAPLNDRIGKLPKEGAV

VIVTSSYNGKMPSNAGQFVQWLQEIKPGELEGVHYAVFGCGDHNWASTYQYVPRFIDEQL

AEKGATRFSGRGEGDVSGDFEGQLDEWKKSMWADAIKAFGLELNENADKERSTLSLQFVR

GLGESPLARSYEASHASIAENRELQSADSDRSTRHIEITLPPDVEYQEGDHLGVLPKNSQ

TNVSRILHRFGLKGTDQVTLSASGRSAGHLPLGRPVSLHDLLSYSVEVQEAATRAQIREL

AAFTVCPPHRRELEELSAEGVYQEQILKKRISMLDLLEKYEACDMPFERFLELLRPLKPR

YYSISSSPRVNPRLASITVGVVRGPAWSGRGEYRGVASNDLAERQAGDDLVMFVRTPESR

FQLPEDPETPIIMVGPGTGVAPFRGFLQAREVLKREGKTLGEAHLYFGCRNDRDFIYRDE

LEQFEKDGIVTVHTAFSRKEGMPKTYVQHVMADHAETLISILDRGGRLYVCGDGSKMAPD

VEAALQKAYQSVHGTGEQEAQNWLRHLQDTGMYAKDVWAGI

>CYP102A48 *Bacillus sp*. YP1

MKQASAIPQPKTYGPLKNLPHLEKEQLSQSLWRIADELGPIFRFDFPGVSSVFVSGHNLV

AEVCDESRFDKNLGKGLQKVREFGGDGLFTSWTHEPNWQKAHRILLPSFSQKAMKGYHSM

MLDIATQLIQKWSRLNPNEEIDVADDMTRLTLDTIGLCGFNYRFNSFYRDSQHPFITSML

RALKEAMNQSKRLGLQDKMMVKTKLQFQKDIEVMNSLVDRMIAERKANPDDNIKDLLSLM

LYAKDPVTGETLDDENIRYQIITFLIAGHETTSGLLSFAIYCLLTHPEKLKKAQEEADRV

LTDDTPEYKQIQQLKYTRMVLNETLRLYPTAPAFSLYAKEDTVLGGEYPISKGQPVTVLI

PKLHRDQNAWGPDAEDFRPERFEDPSSIPHHAYKPFGNGQRACIGMQFALQEATMVLGLV

LKHFELINHTGYELKIKEALTIKPDDFKITVKPRKTAAINVQRKEQADIKAETKPKETKP

KHGTPLLVLYGSNLGTAEGIAGELAAQGRQMGFTAETAPLDDYIGKLPEEGAVVIVTASY

NGSPPDNAAGFVEWLKELEEGQLKGVSYAVFGCGNRSWASTYQRIPRLIDDMMKAKGASR

LTEIGEGDAADDFESHRESWENRFWKETMDAFDINEIAQKEDRPSLSIAFLSEATETPVA

KAYGAFEGVVLENRELQTADSTRSTRHIELEIPAGKTYKEGDHIGILPKNSRELVQRVLS

RFGLQSNHVIKVSGSAHMAHLPMDRPIKVVDLLSSYVELQEPASRLQLRELASYTVCPPH

QKELEQLVSDDGIYKEQVLAKRLTMLDFLEDYPACEMPFERFLALLPSLKPRYYSISSSP

KVHANIVSMTVGVVKASAWSGRGEYRGVASNYLAELNTGDAAACFIRTPQSGFQMPDEPE

TPMIMVGPGTGIAPFRGFIQARSVLKKEGSTLGEALLYFGCRRPDHDDLYREELDQAEQD

GLVTIRRCYSRVENEPKEYVQHLLKQDTQKLMTLIEKGAHIYVCGDGSQMAPDVEKTLRL

AYEAEKGASQEESAEWLQKLQDQKRYVKDVWTGM

>CYP107H1 *Bacillus sp.* YP1

MTIASSTASSEFLKNPYSFYDTLRAVHPIYKGSFLKYPGWYVTGYEETATILKDARFKVR

TPLPESSTKYQDLSHVQNQMMLFQNQPDHRRLRTLASGAFTPRATESYQPYIIETVHHLL

DQVQGEKKMEVISDFAFPLASFVIANIVGVPEEDREQLKEWAASLIQTIDFTRSRKALTE

GNHMAVQAMAYFKELIQKRKRHPQQDMISMLLKGKENDKLTEEEAASTCILLAIAGHETT

VNLISNSVLCLLQHPEQLLKLRENPDLIGTAVEECLRYESPTQMTARVASEDIDISGVTI

RQGEQVYLLLGAANRDPSIFTNPDVFDITRSPNQHLSFGHGHHVCLGSSLARLEAQIAIN

TLLQRMPSLKLADFEWRYRPLFGFRALEELPVTFE

>CYP107J1 *Bacillus sp*. YP1

MSSKEKKSVTILTESQLSSRAFKDEAYEFYKELRKSQALYPLSLGALGKGWLISRYDDAI

HLLKNEKLKKNYENVFTAKEKRPALLKNEETLTKHMLNSDPPDHNRLRTLVQKAFTHRMI

LQLEDKIQHIADSLLDKVQPNKFMNLVDDYAFPLPIIVISEMLGIPLEDRQKFRVWSQAI

IDFSDAPERLQENDHLLGEFVEYLESLVRKKRREPAGDLISALIQAESEGTQLSTEELYS

MIMLLIVAGHETTVNLITNMTYALMCHHDQLEKLRQQPDLMNSAIEEALRFHSPVELTTI

RWTAEPFILHGQEIKRKDVIIISLASANRDEKIFPNADIFDIERKNNRHIAFGHGNHFCL

GAQLARLEAKIAISTLLRRCPNIQLKGEKKQMKWKGNFLMRALEELPISF

>CYP107K1 *Bacillus sp.* YP1

MEKLMFHPHGKEFHHNPFSVLGRFREEEPIHRFELKRFGATYPAWLITRYDDCMAFLKDN

RITRDVKNVMNQEQIKMLNVSEDIDFVSDHMLAKDTPDHTRLRSLVHQAFTPRTIENLRC

SIEQIAEQLLDEMEKENKADIMKSFASPLPFIVISELMGIPKEDRSQFQIWTNAMVDTSE

GNRELTNQALREFKDYIAKLIHDRRIKPKDDLISKLVHAEENGSKLSEKELYSMLFLLVV

AGLETTVNLLGSGTLALLQHMKECEKLKQQPEMIATAVEELLRYTSPVVMMANRWAIEDF

RYKGHSIKRGDMIFIGIGSANRDPNFFENPEILNINRSPNRHISFGFGIHFCLGAPLARL

EGHIAFNALLKRFPDIELAVAPDDIQWRKNVFLRGLESLPVSLSK

>CYP109B1 *Bacillus sp*. YP1

MNVLNRRQALQRALLNGKNKQDAYHPFPWYESMRKDAPVSFDEENQVWSVFLYDDVKKVV

GDKELFSSCMPQQTSSIGNSIINMDPPKHTKIRSVVNKAFTPRVMKQWEPRIQEITDELI

QKFQGRSEFDLVHDFSYPLPVIVISELLGVPSAHMDQFKAWSDLLVSTPKDKSEEAEKAF

LEERDKCEEELAAFFAGIIEEKRNKPAQDIISILVEAEETGEKLSGEELIPFCTLLLVAG

NETTTNLISNAMYSILETPGVYEELRSHPELMPQAVEEALRFRAPAPVLRRIAKRDTEIG

GHLIKEGDMILAFVASANRDEAKFDRPHMFDIHRHPNPHIAFGHGIHFCLGAPLARLEAN

IALTSLISAFPHMECVSITPIENSVIYGLKSFRVKM

>CYP152A1 *Bacillus sp*. YP1

MNEQIPHDKSLDNSLTLLKEGYLFIKNRTERYNSDLFQARLLGKNFICMTGAEAAKVFYD

TDRFQRQNALPKRVQKSLFGVNAIQGMDGSAHIHRKMLFLSLMTPPHQKRLAELMTEEWK

AAVTRWEKADEVVLFEEAKEILCRVACYWAGVPLKETEVKERADDFIDMVDAFGAVGPRH

WKGRRARPRAEEWIEVMIEDARAGLLKTTPGTALHEMAFHTQEDGSQLDSRMAAIELINV

LRPIVAISYFLVFSALALHEHPKYKEWLRSGNSREREMFVQEVRRYYPFGPFLGALVKKD

FVWNNCEFKKGTSVLLDVYGTNHDPRLWDHPDEFRPERFAEREENPFDMIPQGGGHAEKG

HRCPGEGITIEVMKASLDFLVHQIEYDVPEQSLHYSLARMPSLPESGFVMSGIRRKS

>CYP102A36 *Bacillus subtilis* BSn5

MKETSPIPQPKTFGPLGNLPLIDKDKPTLSLIKLAEEQGPIFQIHTPAGTTIVVSGHELV

KEVCDEERFDKSIEGALEKVRAFSGDGLFTSWTHEPNWRKAHNILMPTFSQRAMKDYHEK

MVDIAVQLIQKWARLNPNEAVDVPGDMTRLTLDTIGLCGFNYRFNSYYRETPHPFINSMV

RALDEAMHQMQRLDVQDKLMVRTKRQFRHDIQTMFSLVDSIIAERRANGDQDEKDLLARM

LNVEDPETGEKLDDENIRFQIITFLIAGHETTSGLLSFATYFLLKHPDKLKKAYEEVDRV

LTDAAPTYKQVLELTYIRMILNESLRLWPTAPAFSLYPKEDTVIGGKFPITTNDRISVLI

PQLHRDRDAWGKDAEEFRPERFEHQDQVPHHAYKPFGNGQRACIGMQFALHEATLVLGMI

LKYFTLIDHENYELDIKQTLTLKPGDFHIRVQSRNQDAIHADVQAVEKAASDEQKEKTEA

KGTSVIGLNNRPLLVLYGSDTGTAEGVARELADTASLHGVRTETAPLNDRIGKLPKEGAV

VIVTSSYNGKPPSNAGQFVQWLQEIKPGELEGVHYAVFGCGDHNWASTYQYVPRFIDEQL

AEKGATRFSARGEGDVSGDFEGQLDEWKKSMWADAIKAFGLELNENADKERSTLSLQFVR

GLGESPLARSYEASHASIAENRELQSADSDRSTRHIEIALPPDVEYQEGDHLGVLPKNSQ

TNVSRILHRFGLKGTDQVTLSASGRSAGHLPLGRPVSLHDLLSYSVEVQEAATRAQIREL

AAFTVCPPHRRELEELSAEGVYQEQILKKRISMLDLLEKYEACDMPFERFLELLRPLKPR

YYSISSSPRVNPRQASITVGVVRGPAWSGRGEYRGVASNDLAERQAGDDVVMFIRTPESR

FQLPKDPETPIIMVGPGTGVAPFRGFLQARDVLKREGKTLGEAHLYFGCRNDRDFIYRDE

LERFEKDGIVTVHTAFSRKEGMPKTYVQHLMADQADTLISILDRGGRLYVCGDGSKMAPD

VEAALQKAYQAVHGTGEQEAQNWLRHLQDTGMYAKDVWAGI

>CYP102A48 *Bacillus subtilis* BSn5

MKQASAIPQPKTYGPLKNLPHLEKEQLSQSLWRIADELGPIFRFDFPGVSSVFVSGHNLV

AEVCDESRFDKNLGKGLQKVREFGGDGLFTSWTHEPNWQKAHRILLPSFSQKAMKGYHSM

MLDIATQLIQKWSRLNPNEEIDVADDMTRLTLDTIGLCGFNYRFNSFYRDSQHPFITSML

RALKEAMNQSKRLGLQDKMMVKTKLQFQKDIEVMNSLVDRMIAERKANPDDNIKDLLSLM

LYAKDPVTGETLDDENIRYQIITFLIAGHETTSGLLSFAIYCLLTHPEKLKKAQEEADRV

LTDDTPEYKQIQQLKYTRMVLNETLRLYPTAPAFSLYAKEDTVLGGEYPISKGQPVTVLI

PKLHRDQNAWGPDAEDFRPERFEDPSSIPHHAYKPFGNGQRACIGMQFALQEATMVLGLV

LKHFELINHTGYELKIKEALTIKPDDFKITVKPRKTAAINVQRKEQADIKAETKPKETKP

KHGTPLLVLYGSNLGTAEGIAGELAAQGRQMGFTAETAPLDDYIGKLPEEGAVVIVTASY

NGSPPDNAAGFVEWLKELEEGQLKGVSYAVFGCGNRSWASTYQRIPRLIDDMMKAKGASR

LTEIGEGDAADDFESHRESWENRFWKETMDAFDINEIAQKEDRPSLSIAFLSEATETPVA

KAYGAFEGVVLENRELQTADSTRSTRHIELEIPAGKTYKEGDHIGIMPKNSRELVQRVLS

RFGLQSNHVIKVSGSAHMSHLPMDRPIKVADLLSSYVELQEPASRLQLRELASYTVCPPH

QKELEQLVLDDGIYKEQVLAKRLTMLDFLEDYPACEMPFERFLALLPSLKPRYYSISSSP

KVHANIVSMTVGVVKASAWSGRGEYRGVASNYLAELNTGDAAACFIRTPQSGFQMPDEPE

TPMIMVGPGTGIAPFRGFIQARSVLKKEGSTLGEALLYFGCRRPDHDDLYREELDQAEQE

GLVTIRRCYSRVENESKGYVQHLLKQDSQKLMTLIEKGAHIYVCGDGSQMAPDVEKTLRW

AYETEKGASQEESADWLQKLQDQKRYIKDVWTGN

>CYP107H1 *Bacillus subtilis* BSn5

MMSIVFIFEQKGENHVTIASSTASSEFLKNPYSFYDTLRAVHPIYKGSFLKYPGWYVTGY

EETAAILKDARFKVRTPLPESSTKYQDLSHVQNQMMLFQNPPDHRRLRTLASGAFTPRTT

ESYQPYIIETVHHLLDQVQGKKKMEVISDFAFPLASFVIANIIGVPEEDREQLKEWAASL

IQTIDFTRSRKALTEGNIMAVQAMAYFKELIQKRKRHPQQDMISMLLKGREKDKLTEEEA

ASTCILLAIAGHETTVNLISNSVLCLLQHPEQLLKLRENPDLIGTAVEECLRYESPTQMT

ARVASEDIDICGVTIRQGEQVYLLLGAANRDPSIFTNPDVFDITRSPNPHLSFGHGHHVC

LGSSLARLEAQIAINTLLQRMPSLNLADFEWRYRPLFGFRALEELPVTFE

>CYP107J1 *Bacillus subtilis* BSn5

MSSKEKKSVTILTESQLSSRAFKDEAYEFYKELRKSQPLYPLSLGALGKGWLISRYDDAI

HLLKNEKLKKNYENVFTAKEKRPALLKNEETLTKHMLNSDPPDHNRLRTLVQKAFTHRMI

LQLEDKIQHIADSLLDKVQPNKFMNLVDDYAFPLPIIVISEMLGIPLEDRQKFRVWSQAI

IDFSDAPERLQENDHLLGEFVEYLESLVRKKRREPAGDLISALIQAESEGTQLSTEELYS

MIMLLIVAGHETTVNLITNMTYALMCHHDQLEKLRQQPDLMNSAIEEALRFHSPVELTTI

RWTAEPFILHGQEIKRKDVIIISLASANRDEKIFPNADIFDIERKNNRHIAFGHGNHFCL

GAQLARLEAKIAISTLLRRCPNIQLKGEKKQMKWKGNFLMRALEELPISF

>CYP107K1 *Bacillus subtilis* BSn5

MEKLMFHPHGKEFHHNPFSVLGRFREEEPIHRFELKRFGATYPAWLITRYDDCMAFLKDN

RITRDVKNVMNQEQIKMLNVSEDIDFVSDHMLAKDTPDHTRLRSLVHQAFTPRTIENLRG

SIEQIAEQLLDEMEKENKADIMKSFASPLPFIVISELMGIPKEDRSQFQIWTNAMVDTSE

GNRELTNQALREFKDYIAKLIHDRRIKPKDDLISKLVHAEENGSKLSEKELYSMLFLLIV

AGLETTVNLLGSGTLALLQHMKECEKLKQHPEMIATAVEELLRYTSPVVMMANRWAIEDF

TYKGHSIKRGDMIFIGIGSANRDPNFFENPEILNINRSPNRHISFGFGIHFCLGAPLARL

EGHIAFNALLKRFPDIELAVAPDDIQWRKNVFLRGLESLPVSLSK

>CYP109B1 *Bacillus subtilis* BSn5

MNVLNRRQALQRALLNGKNKQDAYHPFPWYESMRKDAPVSFDEENQVWSVFLYDDVKKVV

GDKELFSSYMPQQTSSIGNSIINMDPPKHTKIRSVVNKAFTPRVMKQWEPRIQEITDELI

QKCQGRSEFDLVHDFSYPLPVIVISELLGVPSAHMDQFKAWSDLLVSTPKDKSEEAEKAF

LEERDKCEEELAAFFAGIIEEKRNKPAQDIISILVKAEETGEKLSGEELIPFCTLLLVAG

NETTTNLISNAMYSILETPGVYEELRSHPELMPQAVEEALRFRAPAPVLRRIAKRDTEIG

GHLIKEGDMVLAFVASANRDEVKFDRPHMFDIRRHPNPHIAFGHGIHFCLGAPLARLEAN

IALTSLISAFPQMECVSITPIENSVIYGLKSFRVKM

>CYP152A1 *Bacillus subtilis* BSn5

MNEQIPHDKSLDNSLTLLKEGYLFIKNRTERYNSDLFQARLLGKNFICMTGAEAAKVFYD

TDRFQRQNALPKRVQKSLFGVNAIQGMDGSAHIHRKMLFLSLMTPPHQKRLAELMTEEWK

AAVTRWEKADEVVLFEEAKEILCRVACYWADVPLKETEVKERADDFIDMVDAFGAVGPRH

WKGRRARPRAEEWIEVMIEDARAGLLKTTPGTALHEMAFHKQEDGSELDSRMAAIELINV

LRPIVAISYFLVFSALALHEHPKYKEWLRSGSSREREMFVQEVRRYYPFGPFLGALVKKD

FVWNNCEFKKGTSVLLDVYGTNHDPRLWDHPDEFRPERFAEREENPFDLIPQGGGHAEKG

HRCPGEGITIEVMKASLDFLVNQIEYDVPEQSLYYSLARMPSLPESGFVMSGIRRKR

>CYP102A36 *Bacillus subtilis* PY79

MKETSPIPQPKTFGPLGNLPLIDKDKPTLSLIKLAEEQGPIFQIHTPAGTTIVVSGHELV

KEVCDEERFDKSIEGALEKVRAFSGDGLFTSWTHEPNWRKAHNILMPTFSQRAMKDYHEK

MVDIAVQLIQKWARLNPNEAVDVPGDMTRLTLDTIGLCGFNYRFNSYYRETPHPFINSMV

RALDEAMHQMQRLDVQDKLMVRTKRQFRYDIQTMFSLVDSIIAERRANGDQDEKDLLARM

LNVEDPETGEKLDDENIRFQIITFLIAGHETTSGLLSFATYFLLKHPDKLKKAYEEVDRV

LTDAAPTYKQVLELTYIRMILNESLRLWPTAPAFSLYPKEDTVIGGKFPITTNDRISVLI

PQLHRDRDAWGKDAEEFRPERFEHQDQVPHHAYKPFGNGQRACIGMQFALHEATLVLGMI

LKYFTLIDHENYELDIKQTLTLKPGDFHISVQSRHQEAIHADVQAAEKAAPDEQKEKTEA

KGASVIGLNNRPLLVLYGSDTGTAEGVARELADTASLHGVRTKTAPLNDRIGKLPKEGAV

VIVTSSYNGKPPSNAGQFVQWLQEIKPGELEGVHYAVFGCGDHNWASTYQYVPRFIDEQL

AEKGATRFSARGEGDVSGDFEGQLDEWKKSMWADAIKAFGLELNENADKERSTLSLQFVR

GLGESPLARSYEASHASIAENRELQSADSDRSTRHIEIALPPDVEYQEGDHLGVLPKNSQ

TNVSRILHRFGLKGTDQVTLSASGRSAGHLPLGRPVSLHDLLSYSVEVQEAATRAQIREL

ASFTVCPPHRRELEELSAEGVYQEQILKKRISMLDLLEKYEACDMPFERFLELLRPLKPR

YYSISSSPRVNPRQASITVGVVRGPAWSGRGEYRGVASNDLAERQAGDDVVMFIRTPESR

FQLPKDPETPIIMVGPGTGVAPFRGFLQARDVLKREGKTLGEAHLYFGCRNDRDFIYRDE

LERFEKDGIVTVHTAFSRKEGMPKTYVQHLMADQADTLISILDRGGRLYVCGDGSKMAPD

VEAALQKAYQAVHGTGEQEAQNWLRHLQDTGMYAKDVWAGI

>CYP102A48 *Bacillus subtilis* PY79

MKQASAIPQPKTYGPLKNLPHLEKEQLSQSLWRIADELGPIFRFDFPGVSSVFVSGHNLV

AEVCDEKRFDKNLGKGLQKVREFGGDGLFTSWTHEPNWQKAHRILLPSFSQKAMKGYHSM

MLDIATQLIQKWSRLNPNEEIDVADDMTRLTLDTIGLCGFNYRFNSFYRDSQHPFITSML

RALKEAMNQSKRLGLQDKMMVKTKLQFQKDIEVMNSLVDRMIAERKANPDENIKDLLSLM

LYAKDPVTGETLDDENIRYQIITFLIAGHETTSGLLSFAIYCLLTHPEKLKKAQEEADRV

LTDDTPEYKQIQQLKYIRMVLNETLRLYPTAPAFSLYAKEDTVLGGEYPISKGQPVTVLI

PKLHRDQNAWGPDAEDFRPERFEDPSSIPHHAYKPFGNGQRACIGMQFALQEATMVLGLV

LKHFELINHTGYELKIKEALTIKPDDFKITVKPRKTAAINVQRKEQADIKAETKPKETKP

KHGTPLLVLFGSNLGTAEGIAGELAAQGRQMGFTAETAPLDDYIGKLPEEGAVVIVTASY

NGAPPDNAAGFVEWLKELEEGQLKGVSYAVFGCGNRSWASTYQRIPRLIDDMMKAKGASR

LTAIGEGDAADDFESHRESWENRFWKETMDAFDINEIAQKEDRPSLSITFLSEATETPVA

KAYGAFEGIVLENRELQTAASTRSTRHIELEIPAGKTYKEGDHIGILPKNSRELVQRVLS

RFGLQSNHVIKVSGSAHMAHLPMDRPIKVVDLLSSYVELQEPASRLQLRELASYTVCPPH

QKELEQLVSDDGIYKEQVLAKRLTMLDFLEDYPACEMPFERFLALLPSLKPRYYSISSSP

KVHANIVSMTVGVVKASAWSGRGEYRGVASNYLAELNTGDAAACFIRTPQSGFQMPNDPE

TPMIMVGPGTGIAPFRGFIQARSVLKKEGSTLGEALLYFGCRRPDHDDLYREELDQAEQD

GLVTIRRCYSRVENEPKGYVQHLLKQDTQKLMTLIEKGAHIYVCGDGSQMAPDVERTLRL

AYEAEKAASQEESAVWLQKLQDQRRYVKDVWTGM

>CYP107H1 *Bacillus subtilis* PY79

MMSIVFIFEQKGENHVTIASSTASSEFLKNPYSFYDTLRAVHPIYKGSFLKYPGWYVTGY

EETAAILKDARFKVRTPLPESSTKYQDLSHVQNQMMLFQNQPDHRRLRTLASGAFTPRTT

ESYQPYIIETVHHLLDQVQGKKKMEVISDFAFPLASFVIANIIGVPEEDREQLKEWAASL

IQTIDFTRSRKALTEGNIMAVQAMAYFKELIQKRKRHPQQDMISMLLKGREKDKLTEEEA

ASTCILLAIAGHETTVNLISNSVLCLLQHPEQLLKLRENPDLIGTAVEECLRYESPTQMT

ARVASEDIDICGVTIRQGEQVYLLLGAANRDPSIFTNPDVFDITRSPNPHLSFGHGHHVC

LGSSLARLEAQIAINTLLQRMPSLNLADFEWRYRPLFGFRALEELPVTFE

>CYP107J1 *Bacillus subtilis* PY79

MSSKEKKSVTILTESQLSSRAFKDEAYEFYKELRKSQALYPLSLGALGKGWLISRYDDAI

HLLKNEKLKKNYENVFTAKEKRPALLKNEETLTKHMLNSDPPDHNRLRTLVQKAFTHRMI

LQLEDKIQHIADSLLDKVQPNKFMNLVDDYAFPLPIIVISEMLGIPLEDRQKFRVWSQAI

IDFSDAPERLQENDHLLGEFVEYLESLVRKKRREPAGDLISALIQAESEGTQLSTEELYS

MIMLLIVAGHETTVNLITNMTYALMCHHDQLEKLRQQPDLMNSAIEEALRFHSPVELTTI

RWTAEPFILHGQEIKRKDVIIISLASANRDEKIFPNADIFDIERKNNRHIAFGHGNHFCL

GAQLARLEAKIAISTLLRRCPNIQLKGEKKQMKWKGNFLMRALEELPISF

>CYP107K1 *Bacillus subtilis* PY79

MEKLMFHPHGKEFHHNPFSVLGRFREEEPIHRFELKRFGATYPAWLITRYDDCMAFLKDN

RITRDVKNVMNQEQIKMLNVSEDIDFVSDHMLAKDTPDHTRLRSLVHQAFTPRTIENLRG

SIEQIAEQLLDEMEKENKADIMKSFASPLPFIVISELMGIPKEDRSQFQIWTNAMVDTSE

GNRELTNQALREFKDYIAKLIHDRRIKPKDDLISKLVHAEENGSKLSEKELYSMLFLLVV

AGLETTVNLLGSGTLALLQHKKECEKLKQQPEMIATAVEELLRYTSPVVMMANRWAIEDF

TYKGHSIKRGDMIFIGIGSANRDPNFFENPEILNINRSPNRHISFGFGIHFCLGAPLARL

EGHIAFKALLKRFPDIELAVAPDDIQWRKNVFLRGLESLPVSLSK

>CYP109B1 *Bacillus subtilis* PY79

MNVLNRRQALQRALLNGKNKQDAYHPFPWYESMRKDAPVSFDEENQVWSVFLYDDVKKVV

GDKELFSSCMPQQTSSIGNSIINMDPPKHTKIRSVVNKAFTPRVMKQWEPRIQEITDELI

QKFQGRSEFDLVHDFSYPLPVIVISELLGVPSAHMEQFKAWSDLLVSTPKDKSEEAEKAF

LEERDKCEEELAAFFAGIIEEKRNKPEQDIISILVEAEETGEKLSGEELIPFCTLLLVAG

NETTTNLISNAMYSILETPGVYEELRSHPELMPQAVEEALRFRAPAPVLRRIAKRDTEIG

GHLIKEGDMVLAFVASANRDEAKFDRPHMFDIRRHPNPHIAFGHGIHFCLGAPLARLEAN

IALTSLISAFPHMECVSITPIENSVIYGLKSFRVKM

>CYP134A1 *Bacillus subtilis* PY79

MSQSIKLFSVLSDQFQNNPYAYFSQLREEDPVHYEESIDSYFISRYHDVRYILQHPDIFT

TKSLVERAEPVMRGPVLAQMHGKEHSAKRRIVVRSFIGDALDHLSPLIKQNAENLLAPYL

ERGKSDLVNDFGKTFAVCVTMDMLGLDKRDHEKISEWHSGVADFITSISQSPEARAHSLW

CSEQLSQYLMPVIKERRVNPGSDLISILCTSEYEGMALSDKDILALILNVLLAATEPADK

TLALMIYHLLNNPEQMNDVLADRSLVPRAIAETLRYKPPVQLIPRQLSQDTVVGGMEIKK

DTIVFCMIGAANRDPEAFEQPDVFNIHREDLGIKSAFSGAARHLAFGSGIHNCVGAAFAK

NEIEIVANIVLDKMRNIRLEEDFCYAESGLYTRGPVSLLVAFDGA

>CYP102A36 *Bacillus subtilis* subsp*. subtilis* 6051-HGW

MKETSPIPQPKTFGPLGNLPLIDKDKPTLSLIKLAEEQGPIFQIHTPAGTTIVVSGHELV

KEVCDEERFDKSIEGALEKVRAFSGDGLFTSWTHEPNWRKAHNILMPTFSQRAMKDYHEK

MVDIAVQLIQKWARLNPNEAVDVPGDMTRLTLDTIGLCGFNYRFNSYYRETPHPFINSMV

RALDEAMHQMQRLDVQDKLMVRTKRQFRYDIQTMFSLVDSIIAERRANGDQDEKDLLARM

LNVEDPETGEKLDDENIRFQIITFLIAGHETTSGLLSFATYFLLKHPDKLKKAYEEVDRV

LTDAAPTYKQVLELTYIRMILNESLRLWPTAPAFSLYPKEDTVIGGKFPITTNDRISVLI

PQLHRDRDAWGKDAEEFRPERFEHQDQVPHHAYKPFGNGQRACIGMQFALHEATLVLGMI

LKYFTLIDHENYELDIKQTLTLKPGDFHISVQSRHQEAIHADVQAAEKAAPDEQKEKTEA

KGASVIGLNNRPLLVLYGSDTGTAEGVARELADTASLHGVRTKTAPLNDRIGKLPKEGAV

VIVTSSYNGKPPSNAGQFVQWLQEIKPGELEGVHYAVFGCGDHNWASTYQYVPRFIDEQL

AEKGATRFSARGEGDVSGDFEGQLDEWKKSMWADAIKAFGLELNENADKERSTLSLQFVR

GLGESPLARSYEASHASIAENRELQSADSDRSTRHIEIALPPDVEYQEGDHLGVLPKNSQ

TNVSRILHRFGLKGTDQVTLSASGRSAGHLPLGRPVSLHDLLSYSVEVQEAATRAQIREL

ASFTVCPPHRRELEELSAEGVYQEQILKKRISMLDLLEKYEACDMPFERFLELLRPLKPR

YYSISSSPRVNPRQASITVGVVRGPAWSGRGEYRGVASNDLAERQAGDDVVMFIRTPESR

FQLPKDPETPIIMVGPGTGVAPFRGFLQARDVLKREGKTLGEAHLYFGCRNDRDFIYRDE

LERFEKDGIVTVHTAFSRKEGMPKTYVQHLMADQADTLISILDRGGRLYVCGDGSKMAPD

VEAALQKAYQAVHGTGEQEAQNWLRHLQDTGMYAKDVWAGI

>CYP102A48 *Bacillus subtilis* subsp. subtilis 6051-HGW

MKQASAIPQPKTYGPLKNLPHLEKEQLSQSLWRIADELGPIFRFDFPGVSSVFVSGHNLV

AEVCDEKRFDKNLGKGLQKVREFGGDGLFTSWTHEPNWQKAHRILLPSFSQKAMKGYHSM

MLDIATQLIQKWSRLNPNEEIDVADDMTRLTLDTIGLCGFNYRFNSFYRDSQHPFITSML

RALKEAMNQSKRLGLQDKMMVKTKLQFQKDIEVMNSLVDRMIAERKANPDENIKDLLSLM

LYAKDPVTGETLDDENIRYQIITFLIAGHETTSGLLSFAIYCLLTHPEKLKKAQEEADRV

LTDDTPEYKQIQQLKYIRMVLNETLRLYPTAPAFSLYAKEDTVLGGEYPISKGQPVTVLI

PKLHRDQNAWGPDAEDFRPERFEDPSSIPHHAYKPFGNGQRACIGMQFALQEATMVLGLV

LKHFELINHTGYELKIKEALTIKPDDFKITVKPRKTAAINVQRKEQADIKAETKPKETKP

KHGTPLLVLFGSNLGTAEGIAGELAAQGRQMGFTAETAPLDDYIGKLPEEGAVVIVTASY

NGAPPDNAAGFVEWLKELEEGQLKGVSYAVFGCGNRSWASTYQRIPRLIDDMMKAKGASR

LTAIGEGDAADDFESHRESWENRFWKETMDAFDINEIAQKEDRPSLSITFLSEATETPVA

KAYGAFEGIVLENRELQTAASTRSTRHIELEIPAGKTYKEGDHIGILPKNSRELVQRVLS

RFGLQSNHVIKVSGSAHMAHLPMDRPIKVVDLLSSYVELQEPASRLQLRELASYTVCPPH

QKELEQLVSDDGIYKEQVLAKRLTMLDFLEDYPACEMPFERFLALLPSLKPRYYSISSSP

KVHANIVSMTVGVVKASAWSGRGEYRGVASNYLAELNTGDAAACFIRTPQSGFQMPNDPE

TPMIMVGPGTGIAPFRGFIQARSVLKKEGSTLGEALLYFGCRRPDHDDLYREELDQAEQD

GLVTIRRCYSRVENEPKGYVQHLLKQDTQKLMTLIEKGAHIYVCGDGSQMAPDVERTLRL

AYEAEKAASQEESAVWLQKLQDQRRYVKDVWTGM

>CYP107H1 *Bacillus subtilis* subsp. *subtilis* 6051-HGW

MTIASSTASSEFLKNPYSFYDTLRAVHPIYKGSFLKYPGWYVTGYEETAAILKDARFKVR

TPLPESSTKYQDLSHVQNQMMLFQNQPDHRRLRTLASGAFTPRTTESYQPYIIETVHHLL

DQVQGKKKMEVISDFAFPLASFVIANIIGVPEEDREQLKEWAASLIQTIDFTRSRKALTE

GNIMAVQAMAYFKELIQKRKRHPQQDMISMLLKGREKDKLTEEEAASTCILLAIAGHETT

VNLISNSVLCLLQHPEQLLKLRENPDLIGTAVEECLRYESPTQMTARVASEDIDICGVTI

RQGEQVYLLLGAANRDPSIFTNPDVFDITRSPNPHLSFGHGHHVCLGSSLARLEAQIAIN

TLLQRMPSLNLADFEWRYRPLFGFRALEELPVTFE

>CYP107J1 *Bacillus subtilis* subsp. *subtilis* 6051-HGW

MSSKEKKSVTILTESQLSSRAFKDEAYEFYKELRKSQALYPLSLGALGKGWLISRYDDAI

HLLKNEKLKKNYENVFTAKEKRPALLKNEETLTKHMLNSDPPDHNRLRTLVQKAFTHRMI

LQLEDKIQHIADSLLDKVQPNKFMNLVDDYAFPLPIIVISEMLGIPLEDRQKFRVWSQAI

IDFSDAPERLQENDHLLGEFVEYLESLVRKKRREPAGDLISALIQAESEGTQLSTEELYS

MIMLLIVAGHETTVNLITNMTYALMCHHDQLEKLRQQPDLMNSAIEEALRFHSPVELTTI

RWTAEPFILHGQEIKRKDVIIISLASANRDEKIFPNADIFDIERKNNRHIAFGHGNHFCL

GAQLARLEAKIAISTLLRRCPNIQLKGEKKQMKWKGNFLMRALEELPISF

>CYP107K1 *Bacillus subtilis* subsp. *subtilis* 6051-HGW

MEKLMFHPHGKEFHHNPFSVLGRFREEEPIHRFELKRFGATYPAWLITRYDDCMAFLKDN

RITRDVKNVMNQEQIKMLNVSEDIDFVSDHMLAKDTPDHTRLRSLVHQAFTPRTIENLRG

SIEQIAEQLLDEMEKENKADIMKSFASPLPFIVISELMGIPKEDRSQFQIWTNAMVDTSE

GNRELTNQALREFKDYIAKLIHDRRIKPKDDLISKLVHAEENGSKLSEKELYSMLFLLVV

AGLETTVNLLGSGTLALLQHKKECEKLKQQPEMIATAVEELLRYTSPVVMMANRWAIEDF

TYKGHSIKRGDMIFIGIGSANRDPNFFENPEILNINRSPNRHISFGFGIHFCLGAPLARL

EGHIAFKALLKRFPDIELAVAPDDIQWRKNVFLRGLESLPVSLSK

>CYP109B1 *Bacillus subtilis* subsp. *subtilis* 6051-HGW

MNVLNRRQALQRALLNGKNKQDAYHPFPWYESMRKDAPVSFDEENQVWSVFLYDDVKKVV

GDKELFSSCMPQQTSSIGNSIINMDPPKHTKIRSVVNKAFTPRVMKQWEPRIQEITDELI

QKFQGRSEFDLVHDFSYPLPVIVISELLGVPSAHMEQFKAWSDLLVSTPKDKSEEAEKAF

LEERDKCEEELAAFFAGIIEEKRNKPEQDIISILVEAEETGEKLSGEELIPFCTLLLVAG

NETTTNLISNAMYSILETPGVYEELRSHPELMPQAVEEALRFRAPAPVLRRIAKRDTEIG

GHLIKEGDMVLAFVASANRDEAKFDRPHMFDIRRHPNPHIAFGHGIHFCLGAPLARLEAN

IALTSLISAFPHMECVSITPIENSVIYGLKSFRVKM

>CYP152A1 *Bacillus subtilis* subsp. *subtilis* 6051-HGW

MNEQIPHDKSLDNSLTLLKEGYLFIKNRTERYNSDLFQARLLGKNFICMTGAEAAKVFYD

TDRFQRQNALPKRVQKSLFGVNAIQGMDGSAHIHRKMLFLSLMTPPHQKRLAELMTEEWK

AAVTRWEKADEVVLFEEAKEILCRVACYWAGVPLKETEVKERADDFIDMVDAFGAVGPRH

WKGRRARPRAEEWIEVMIEDARAGLLKTTSGTALHEMAFHTQEDGSQLDSRMAAIELINV

LRPIVAISYFLVFSALALHEHPKYKEWLRSGNSREREMFVQEVRRYYPFGPFLGALVKKD

FVWNNCEFKKGTSVLLDLYGTNHDPRLWDHPDEFRPERFAEREENLFDMIPQGGGHAEKG

HRCPGEGITIEVMKASLDFLVHQIEYDVPEQSLHYSLARMPSLPESGFVMSGIRRKS

>CYP102A36 *Bacillus subtilis* subsp. *subtilis* BSP1

MKETSPIPQPKTFGPLGNLPLIDKDKPTLSLIKLAEEQGPIFQIHTPAGTTIVVSGHELV

KEVCDEERFDKSIEGALEKVRAFSGDGLFTSWTHEPNWRKAHNILMPTFSQRAMKDYHEK

MVDIAVQLIQKWARLNPNEAVDVPGDMTRLTLDTIGLCGFNYRFNSYYRETPHPFINSMV

RALDEAMHQMQRLDVQDKLMVRTKRQFHHDIQTMFSLVDSIIAERRSNGDQDEKDLLARM

LNVEDPETGEKLDDENIRFQIITFLIAGHETTSGLLSFATYFLLKHPDKLKKAYEEVDRV

LTDAAPTYKQVLELTYIRMILNESLRLWPTAPAFSLYPKEDTVIGGKFPITTNDRISVLI

PQLHRDRDAWGKDAEEFRPERFEHQDQVPHHAYKPFGNGQRACIGMQFALHEATLVLGMI

LKYFTLIDHENYELDIKQTLTLKPGDFHIRVQSRNQDAIHADVQAAEKAASDEQKEKTEA

KGTSVIGLNNRPLLVLYGSDTGTAEGVARELADTASLHGVRTETAPLNDRIGKLPKEGAV

VIVTSSYNGKPPSNAGQFVQWLQEIKPGELEGVHYAVFGCGDHNWASTYQYVPRFIDEQL

AEKGATRFSARGEGDVSGDFEGQLDEWKKSMWADAIKAFGLELNENADKERSTLSLQFVR

GLGESPLARSYEASHASIAENRELQSADSDRSTRHIEIALPPDVEYQEGDHLGVLPKNSQ

TNVSRILHRFGLKGTDQVTLSASGHSAGHLPLGRPVSLHDLLSYSVEVQEAATRAQIREL

AAFTVCPPHRRELEELSAEGVYQEQILKKRISMLDLLEKYEACDMPFERFLELLRPLKPR

YYSISSSPRVNPRLASITVGVVRGPAWSGRGEYRGVASNDLAERQAGDDLVMFVRTPESR

FQLPEDPETPIIMVGPGTGVAPFRGFLQAREVLKREGKTLGEAHLYFGCRNDRDFIYRDE

LEQFEKDGIVTVHTAFSRKEGIPKTYVQHVMADHAETLISILDRGGRLYVCGDGSKMAPD

VEAALQKAYQSVHGTGEQEAQNWLRHLQDTGMYAKDVWAGI

>CYP102A48 *Bacillus subtilis* subsp. *subtilis* BSP1

KQASAIPQPKTYGPLKNLPHLEKEQLSQSLWRIADELGPIFRFDFPGVSSVFVSGHNFVA

EVCDESRFDKNLGKGLQKVREFGGDGLFTSWTHEPNWQKAHRILLPSFSQKAMKGYHSMM

LDIATQLIQKWSRLNPNEEIDVADDMTRLTLDTIGLCGFNYRFNSFYRDSQHPFITSMLR

ALKEAMNQSKRLGLQDKMMVKTKLQFQKDIEVMNSLVDRMIAERKANPDENIKDLLSLML

YAKDPVTGETLDDENIRYQIITFLIAGHETTSGLLSFAIYCLLTHPEKLKKAQEEADCVL

TDDTPEYKQIQQLKYIRMVLNETLRLYPTAPAFSLYAKEDTVLGGEYPISKGQPVTVLIP

KLHRDQNAWGPDAEDFRPERFEDPSSIPHHAYKPFGNGQRACIGMQFALQEATMVLGLVL

KHFELINHTGYELKIKEALTIKPDDFKITVKPRKTAAINVQRKEQADIKAETKPKETKPK

HGTPLLVLYGSNLGTAEGIAGELVAQGCQMGFTAETAPLDDYIGKLPEEGAVVIVTASYN

GAPPDNAAGFVEWLKELEEGQLKGVSYAVFGCGNRSWASTYQRIPRLIDDMMKAKGASRL

TAIGEGDAADDFESHRESWENRFWKETMDAFDINELAQKEDRPSLSITFLSEATETPVAK

AYGAFEGIVLENRELQTAASTRSTRHIELEIPAGKTYKEGDHIGILPKNSRELVQRVLSR

FGLQSNHVIKVSGSAHMAHLPMDRPIKVVDLLSSYVELQEPASRLQLRELASYTVCPPHQ

KELEQLVSDDGIYKEQVLAKRLTMLDFLEDYPACEMPFERFLALLPSLKPRYYSISSSPK

VHANIVSMTVGVVKASAWSGRGEYRGVASNYLAELNTGDAAACFIRTPQSGFQMPDEPET

PMIMIGPGTGIAPFRGFIQARSVLKKEGSTLGEALLYFGCRRPDHDDLYREELDQAEQDG

LVTIRRCYSRVENEPKGYVQHLLKQDTQKLMTLIEKGAHIYVCGDGSQMAPDVEKTLRLA

YEAEKGASQEESAEWLQKLQDQRRYVKDVWTGM

>CYP107H1 *Bacillus subtilis* subsp. *subtilis* BSP1

MTIASSTASSEFLKNPYSFYDTLRAVHPIYKGSFLKYPGWYVTGYEETAAILKDARFKVR

TPLPESSTKYQDLSHVQNQMMLFQNQPDHRRLRTLASGAFTPRATESYQPYIIETVHHLL

DQVQGKKKMEVISDFAFPLASFVIANIIGVPEEDREQLKEWAGSLIQTIDFTRSRKALTE

GNIMAVQAMAYFKELIQKRKRHPQQDMISMLLKGREKDKLTEEEAASTCILLAIAGHETT

VNLISNSVLCLLQHPEQILKLRENPDLIGTAVEECLRYESPTQMTARVASEDIDICGVTI

RQGEQIYLLLGAANRDPSIFTNPDVFDITRSPNPHLSFGHGHHVCLGSSLARLEAQIAIN

TLLQRMPSLKLADFEWRYRPLFGFRALEELPVTFE

>CYP107J1 *Bacillus subtilis* subsp. *subtilis* BSP1

MSSKEKKSVTILTESQLSSRAFKDEAYEFYKELRKSQPLYPLSLGALGKGWLISRYDDAI

HLLKNEKLKKNYENVFTAKEKRPALLKNEETLTKHMLNSDPPDHNRLRTLVQKAFTHRMI

LQLEDKIQHIADSLLDKVQPNKFMNLVDDYAFPLPIIVISEMLGIPLEDRQKFRVWSQAI

IDFSDAPERLQENDHLLGEFVEYLESLVRKKRREPAGDLISALIQAESEGTQLSTEELYS

MIMLLIVAGHETTVNLITNMTYALMCHHDQLEKLRQQPDLMNSAIEEALRFHSPVELTTI

RWTAEPFILHGQEIKRKDVIIISLASANRDEKIFPNADIFDIERKNNRHIAFGNGNHFCL

GAQLARLEAKIAISTLLRRCPNIQLKGEKKQMKWKGNFLMRALEELPISF

>CYP107K1 *Bacillus subtilis* subsp*. subtilis* BSP1

MQMEKLMFHPHGKEFHHNPFSVLGRFREEEPIHRFELKRFGATYPAWLITRYDDCMAFLK

DNRITRDVKNVMNQEQIKMLNVSEDIDFVSDHMLAKDTPDHTRLRSLVHQAFTPRTIENL

RGSIEQIAEQLLDEMEKENKADIMKSFASPLPFIVISELMGIPKEDRSQFQIWTNAMVDT

SEGNRELTNQALREFKDYISKLIHDRRIKPKDDLISKLVHAEENGSKLSEKELYSMLFLL

VVAGLETTVNLLGSGTLALLQHKKECEKLKQQPEMIATAVEELLRYTSPVVMMANRWAIE

DFTYKGHSIKRGDMIFIGIGSANRDPNFFENPEILNINRSPNRHISFGFGIHFCLGAPLA

RLEGHIAFNAASEEIS

>CYP109B1 *Bacillus subtilis* subsp. *subtilis* BSP1

MNVLNRRQALQRALLNGKNKQDAYHPFPWYESMRKDAPVSFDEENQVWSVFLYDDVKKVV

GDKELFSSYMPQQTSSIGNSIINMDPPKHTKIRSVVNKAFTPRVMKQWEPRIQEITDELI

QKCQGRSEFDLVHDFSYPLPVIVISELLGVPSAHMDQFKAWSDLLVSTPKDKSEEAEKAF

LEERDKCEEELAAFFAGIIEEKRNKPAQDIISILVKAEETGEKLSGEELIPFCTLLLVAG

NETTTNLISNAMYSILETPGVYEELRSHPELMPQAVEEALRFRAPAPVLRRIAKRDTEIG

GHLIKEGDMVLAFVASANRDEAKFDRPHMFDIRRHPNPHIAFGHGIHFCLGAPLARLEAN

IALTSLISAFPHMECVSITPIENSVIYGLKSFRVKM

>CYP152A1 *Bacillus subtilis* subsp. *subtilis* BSP1

MNEQIPHDKSLDNSLTLLKEGYLFIKNRTERYNSDLFQARLLGKNFICMTGAEAAKVFYD

TDRFQRQNALPKRVQKSLFGVNAIQGMDGSAHIHRKMLFLSLMTPPHQKRLAELMTEEWK

AAVTRWEKADEVVLFEEAKEILCRVACYWAGVPLKETEVKERADDFIDMVDAFGAVGPRH

WKGRRARPRAEEWIEVMIEDARAGLLKTTPGTALHEMAFHTQEDGSQLDSRMAAIELINV

LRPIVAISYFLVFSALALHEHPKYKEWLRSGNSREREMFVQEVRRYYPFGPFLGALVKKD

FVWNNCEFKKGTSVLLDLYGTNHDPRLWDHPNEFRPERFAEREENLFDMIPQGGGHAEKG

HRCPGEGITIEVMKASLDLLVHQIEYDVPEQSLHYSLARMPSLPESGFVMSGIRRKR

>CYP102A36 *Bacillus subtilis* subsp. *subtilis* JH642

MKETSPIPQPKTFGPLGNLPLIDKDKPTLSLIKLAEEQGPIFQIHTPAGTTIVVSGHELV

KEVCDEERFDKSIEGALEKVRAFSGDGLFTSWTHEPNWRKAHNILMPTFSQRAMKDYHEK

MVDIAVQLIQKWARLNPNEAVDVPGDMTRLTLDTIGLCGFNYRFNSYYRETPHPFINSMV

RALDEAMHQMQRLDVQDKLMVRTKRQFRYDIQTMFSLVDSIIAERRANGDQDEKDLLARM

LNVEDPETGEKLDDENIRFQIITFLIAGHETTSGLLSFATYFLLKHPDKLKKAYEEVDRV

LTDAAPTYKQVLELTYIRMILNESLRLWPTAPAFSLYPKEDTVIGGKFPITTNDRISVLI

PQLHRDRDAWGKDAEEFRPERFEHQDQVPHHAYKPFGNGQRACIGMQFALHEATLVLGMI

LKYFTLIDHENYELDIKQTLTLKPGDFHISVQSRHQEAIHADVQAAEKAAPDEQKEKTEA

KGASVIGLNNRPLLVLYGSDTGTAEGVARELADTASLHGVRTKTAPLNDRIGKLPKEGAV

VIVTSSYNGKPPSNAGQFVQWLQEIKPGELEGVHYAVFGCGDHNWASTYQYVPRFIDEQL

AEKGATRFSARGEGDVSGDFEGQLDEWKKSMWADAIKAFGLELNENADKERSTLSLQFVR

GLGESPLARSYEASHASIAENRELQSADSDRSTRHIEIALPPDVEYQEGDHLGVLPKNSQ

TNVSRILHRFGLKGTDQVTLSASGRSAGHLPLGRPVSLHDLLSYSVEVQEAATRAQIREL

ASFTVCPPHRRELEELSAEGVYQEQILKKRISMLDLLEKYEACDMPFERFLELLRPLKPR

YYSISSSPRVNPRQASITVGVVRGPAWSGRGEYRGVASNDLAERQAGDDVVMFIRTPESR

FQLPKDPETPIIMVGPGTGVAPFRGFLQARDVLKREGKTLGEAHLYFGCRNDRDFIYRDE

LERFEKDGIVTVHTAFSRKEGMPKTYVQHLMADQADTLISILDRGGRLYVCGDGSKMAPD

VEAALQKAYQAVHGTGEQEAQNWLRHLQDTGMYAKDVWAGI

>CYP102A48 *Bacillus subtilis* subsp. *subtilis* JH642

MKQASAIPQPKTYGPLKNLPHLEKEQLSQSLWRIADELGPIFRFDFPGVSSVFVSGHNLV

AEVCDEKRFDKNLGKGLQKVREFGGDGLFTSWTHEPNWQKAHRILLPSFSQKAMKGYHSM

MLDIATQLIQKWSRLNPNEEIDVADDMTRLTLDTIGLCGFNYRFNSFYRDSQHPFITSML

RALKEAMNQSKRLGLQDKMMVKTKLQFQKDIEVMNSLVDRMIAERKANPDENIKDLLSLM

LYAKDPVTGETLDDENIRYQIITFLIAGHETTSGLLSFAIYCLLTHPEKLKKAQEEADRV

LTDDTPEYKQIQQLKYIRMVLNETLRLYPTAPAFSLYAKEDTVLGGEYPISKGQPVTVLI

PKLHRDQNAWGPDAEDFRPERFEDPSSIPHHAYKPFGNGQRACIGMQFALQEATMVLGLV

LKHFELINHTGYELKIKEALTIKPDDFKITVKPRKTAAINVQRKEQADIKAETKPKETKP

KHGTPLLVLFGSNLGTAEGIAGELAAQGRQMGFTAETAPLDDYIGKLPEEGAVVIVTASY

NGAPPDNAAGFVEWLKELEEGQLKGVSYAVFGCGNRSWASTYQRIPRLIDDMMKAKGASR

LTAIGEGDAADDFESHRESWENRFWKETMDAFDINEIAQKEDRPSLSITFLSEATETPVA

KAYGAFEGIVLENRELQTAASTRSTRHIELEIPAGKTYKEGDHIGILPKNSRELVQRVLS

RFGLQSNHVIKVSGSAHMAHLPMDRPIKVVDLLSSYVELQEPASRLQLRELASYTVCPPH

QKELEQLVSDDGIYKEQVLAKRLTMLDFLEDYPACEMPFERFLALLPSLKPRYYSISSSP

KVHANIVSMTVGVVKASAWSGRGEYRGVASNYLAELNTGDAAACFIRTPQSGFQMPNDPE

TPMIMVGPGTGIAPFRGFIQARSVLKKEGSTLGEALLYFGCRRPDHDDLYREELDQAEQD

GLVTIRRCYSRVENEPKGYVQHLLKQDTQKLMTLIEKGAHIYVCGDGSQMAPDVERTLRL

AYEAEKAASQEESAVWLQKLQDQRRYVKDVWTGM

>CYP107H1 *Bacillus subtilis* subsp. *subtilis* JH642

MTIASSTASSEFLKNPYSFYDTLRAVHPIYKGSFLKYPGWYVTGYEETAAILKDARFKVR

TPLPESSTKYQDLSHVQNQMMLFQNQPDHRRLRTLASGAFTPRTTESYQPYIIETVHHLL

DQVQGKKKMEVISDFAFPLASFVIANIIGVPEEDREQLKEWAASLIQTIDFTRSRKALTE

GNIMAVQAMAYFKELIQKRKRHPQQDMISMLLKGREKDKLTEEEAASTCILLAIAGHETT

VNLISNSVLCLLQHPEQLLKLRENPDLIGTAVEECLRYESPTQMTARVASEDIDICGVTI

RQGEQVYLLLGAANRDPSIFTNPDVFDITRSPNPHLSFGHGHHVCLGSSLARLEAQIAIN

TLLQRMPSLNLADFEWRYRPLFGFRALEELPVTFE

>CYP107J1 *Bacillus subtilis* subsp. *subtilis* JH642

MSSKEKKSVTILTESQLSSRAFKDEAYEFYKELRKSQALYPLSLGALGKGWLISRYDDAI

HLLKNEKLKKNYENVFTAKEKRPALLKNEETLTKHMLNSDPPDHNRLRTLVQKAFTHRMI

LQLEDKIQHIADSLLDKVQPNKFMNLVDDYAFPLPIIVISEMLGIPLEDRQKFRVWSQAI

IDFSDAPERLQENDHLLGEFVEYLESLVRKKRREPAGDLISALIQAESEGTQLSTEELYS

MIMLLIVAGHETTVNLITNMTYALMCHHDQLEKLRQQPDLMNSAIEEALRFHSPVELTTI

RWTAEPFILHGQEIKRKDVIIISLASANRDEKIFPNADIFDIERKNNRHIAFGHGNHFCL

GAQLARLEAKIAISTLLRRCPNIQLKGEKKQMKWKGNFLMRALEELPISF

>CYP107K1 *Bacillus subtilis* subsp. *subtilis* JH642

MEKLMFHPHGKEFHHNPFSVLGRFREEEPIHRFELKRFGATYPAWLITRYDDCMAFLKDN

RITRDVKNVMNQEQIKMLNVSEDIDFVSDHMLAKDTPDHTRLRSLVHQAFTPRTIENLRG

SIEQIAEQLLDEMEKENKADIMKSFASPLPFIVISELMGIPKEDRSQFQIWTNAMVDTSE

GNRELTNQALREFKDYIAKLIHDRRIKPKDDLISKLVHAEENGSKLSEKELYSMLFLLVV

AGLETTVNLLGSGTLALLQHKKECEKLKQQPEMIATAVEELLRYTSPVVMMANRWAIEDF

TYKGHSIKRGDMIFIGIGSANRDPNFFENPEILNINRSPNRHISFGFGIHFCLGAPLARL

EGHIAFKALLKRFPDIELAVAPDDIQWRKNVFLRGLESLPVSLSK

>CYP152A1 *Bacillus subtilis* subsp. *subtilis* JH642

MNEQIPHDKSLDNSLTLLKEGYLFIKNRTERYNSDLFQARLLGKNFICMTGAEAAKVFYD

TDRFQRQNALPKRVQKSLFGVNAIQGMDGSAHIHRKMLFLSLMTPPHQKRLAELMTEEWK

AAVTRWEKADEVVLFEEAKEILCRVACYWAGVPLKETEVKERADDFIDMVDAFGAVGPRH

WKGRRARPRAEEWIEVMIEDARAGLLKTTSGTALHEMAFHTQEDGSQLDSRMAAIELINV

LRPIVAISYFLVFSALALHEHPKYKEWLRSGNSREREMFVQEVRRYYPFGPFLGALVKKD

FVWNNCEFKKGTSVLLDLYGTNHDPRLWDHPDEFRPERFAEREENLFDMIPQGGGHAEKG

HRCPGEGITIEVMKASLDFLVHQIEYDVPEQSLHYSLARMPSLPESGFVMSGIRRKS

>CYP134A1 *Bacillus subtilis* subsp. *subtilis* JH642

MSQSIKLFSVLSDQFQNNPYAYFSQLREEDPVHYEESIDSYFISRYHDVRYILQHPDIFT

TKSLVERAEPVMRGPVLAQMHGKEHSAKRRIVVRSFIGDALDHLSPLIKQNAENLLAPYL

ERGKSDLVNDFGKTFAVCVTMDMLGLDKRDHEKISEWHSGVADFITSISQSPEARAHSLW

CSEQLSQYLMPVIKERRVNPGSDLISILCTSEYEGMALSDKDILALILNVLLAATEPADK

TLALMIYHLLNNPEQMNDVLADRSLVPRAIAETLRYKPPVQLIPRQLSQDTVVGGMEIKK

DTIVFCMIGAANRDPEAFEQPDVFNIHREDLGIKSAFSGAARHLAFGSGIHNCVGAAFAK

NEIEIVANIVLDKMRNIRLEEDFCYAESGLYTRGPVSLLVAFDGA

>CYP102A36 *Bacillus subtilis* subsp. *subtilis* RO-NN-1

MKETSPIPQPKTFGPLGNLPLIDKDKPTLSLIKLAEEQGPIFQIHTPAGTTIVVSGHELV

KEVCDEERFDKSIEGALEKVRAFSGDGLFTSWTHEPNWRKAHNILMPTFSQRAMKDYHEK

MVDIAVQLIQKWARLNPNEAVDVPGDMTRLTLDTIGLCGFNYRFNSYYRETPHPFINSMV

RALDEAMHQMQRLDFQDKLMVRTKRQFHHDIQTMFSLVDSIIAERRSNGDQDEKDLLARM

LNVEDPETGEKLDDENIRFQIITFLIAGHETTSGLLSFATYFLLKHPDKLKKAYEEVDRV

LTDAAPTYKQVLELKYIRMILNESLRLWPTAPAFSLYPKEDTVIGGKFPITTKDRISVLI

PQLHRDRDAWGKDAEEFRPERFEHQDQVPHHAYKPFGNGQRACIGMQFALHEATLVLGMI

LKYFTLIDHENYELDIKQTLTLKPGDFHIRVQSRHQEAIHADVQAAEKAAPDEQKEKTEA

KDASVIGLNNRLLLVLYGSDTGTAEGVARELADTASLHGVRTETAPLNDRIGKLPKEGAV

VIVTSSYNGKPPSNAGQFVQWLQEIKLGELEGVHYAVFGCGDHNWASTYQDVPRFIDEQL

AEKGATRFSARGEGDVSGDFEGQLDEWKKSMWADAIKAFGLELNENADKERSTLSLQFVR

GLGESPLARSYEASHASIVENRELQSADSDRSTRHIEIALPPDVEYQEGDHLGILPKNSQ

TNVSRILHRFGLKGTDQVTLSASGRSAGHLPLGRPVSLHDLLSYSVEVQEAATRAQIREL

AAFTVCPPHRRELEELTAEGVYQEQILKKRISMLDLLEKYEACDMPFERFLELLRPLKPR

YYSISSSPRVNPRQASITVGVVRGPAWSGRGEYRGVASNDLAERQAGDDVVMFIRTPESR

FQLPEDPETPIIMVGPGTGVAPFRGFLQAREVLKREGKKLGEAHLYFGCRNDRDFIYRDE

LEQFKKDGIVTVHTAFSRKEGMPKTYVQHLMADHAETLISILDRGGRLYVCGDGSKMAPD

VEAALQKAYQSVHGTGEQEAQNWLRHLQDTGMYAKDVWAGI

>CYP102A48 *Bacillus subtilis* subsp. *subtilis* RO-NN-1

MKQASAIPQPKTYGPLKNLPHLEKEQLSQSLWRIADELGPIFRFDFPGVSSVFVSGHNLV

AEVCDESRFDKNLGKGLQKVREFGGDGLFTSWTHEPNWQKAHRILLPSFSQKAMKGYHSM

MLDIATQLIQKWSRLNPNEEIDVADDMTRLTLDTIGLCGFNYRFNSFYRDSQHPFITSML

RALKEAMNQSKRLGLQDKMMVKTKLQFQKDIEVMNSLVDRMIAERKANPDDNIKDLLSLM

LYAKDPVTGETLDDENIRYQIITFLIAGHETTSGLLSFAIYCLLTHPEKLKKAQEEADRV

LTDDTPEYKQIQQLKYIRMVLNETLRLYPTAPAFSLYAKEDTVLGGEYPISKGQPVTVLI

PKLHRDQNAWGPDAEDFRPERFEDPSSIPHHAYKPFGNGQRACIGMQFALQEATMVLGLV

LKHFELINHTGYELKIKEALTIKPDDFKITVKPRKTAAINVQRKEQADIKAETKPKETKP

KHGTPLLVLYGSNLGTAEGIAGELAAQGRQMGFTAETAPLDDYIGKLPEEGAVVIVTASY

NGAPPDNAAGFVEWLKELEEGQLKGVSYAVFGCGNRSWASTYQRIPRLIDDMMKAKGASR

LTAIGEGDAADDFESHRESWENRFWKETMDAFDINEIAQKEDRPSLSITFLSEATETPVA

KAYGAFEGIVLENRELQTADSTRSTRHIELEIPDGKTYKEGDHIGILPKNSRELVQRVLS

RFGLQSNHVIKVSGSAHMAHLPMDRPIRVVDLLSSYVELQEPASRLQLRELASYTVCPPH

QKELEQLVSDDGIYKEHVLAKRLTMLDFLEDYPACEMPFERFLALLPSLKPRYYSISSSP

KVHANIVSMTVGVVKASAWSGRGEYRGVASNYLAELNTGDAAACFIRTPQSGFQMPDELE

TPMIMVGPGTGIAPFRGFIQARSVLKKEGSTLGEALLYFGCRRPDHDDLYREELDQAEQD

GLVTIRRCYSRVENEPKEYVQHLLKQDTQKLMTLIEKGAHIYVCGDGSQMAPDVEKTLRL

AYEAEKGASQEESAEWLQKLQDQRRYVKDVWTGM

>CYP107H1 *Bacillus subtilis* subsp. *subtilis* RO-NN-1

MTIASSAASSEFLKNPYSFYDTLRAVHPIYKGSFLKYPGWYVTGYEETTAILKDARFKVR

TPLPDSSTKYQDLSNVQNQMMLFQNQPDHRRLRTLASGALTPRATESYQPYIIETVHHLL

DQVQGKKKMEVISDFAFPLASFVIANIIGVPEEDREQLKEWAASLIQTIDFTRSRKALTE

GNHMAVQAMAYFKELIQKRKRHPQQDMISMLLKGKEKDKLTEEEAASTCILLAIAGHETT

VNLISNSVLCLLQHPEQLLKLRENPDLIGTAVEECLRYESPTQMTARVASEDIDISGVTI

RQGEQVYLLLGAANRDPSTFTNPDVFDITRSPNPHLSFGNGHHVCLGSSLARLEAQIAIN

TLLQRMPSLKLADFEWRYRPLFGFRALEELPVTFE

>CYP107J1 *Bacillus subtilis* subsp. *subtilis* RO-NN-1

MSSKEKKSVTILTESHLSSRAFKDEAYKFYKELRKSQPLYPLSLGTLGKGWLITRYDDAI

HLLKNEKLKKNYENVISAKEENRPVLLKNEEPLTKHMLNSDPPDHNRLRTLVQKAFTHRM

ILQLEDKIQHIADSLLDKVQPNKLMNLVDDYAFPLPIIVISEMLGIPLEDRQKFRVWSQA

IIDFSDAPERLQENDHLLGEFVEYLESLVRKKRSEPSGDLISAFIQAESEGTQLSTEELY

SMIMLLIVAGHETTVNLITNMTYALMCHGDQLEKLRHQPDLMNSAIEEALRFHSPVELTT

IRWTAEPFILHGQEIKRKDVIIISLASANRDEKIFPNADLFDIERKSNRHIAFGHGNHFC

LGAQLARLEAKIAISTLLRRCPNIQLKGEKEQMKWKGNFLMRALEELPLSF

>CYP107K1 *Bacillus subtilis* subsp. *subtilis* RO-NN-1

MEKLMFHPHGKEFHHNPFSVLGRFREEEPIHRFELKRFGATYPAWLITRYDDCMAFLKDN

RITRDVKNVMNQEQIKMLNVSEDIDFVSDHMLAKDTPDHTRLRSLVHQAFTPRTIENLRG

SIEQIAEQLLDEMEKENKADIMKSFASPLPFIVISELMGIPKEDRSQFQIWTNAMVDTSE

GNRELTNQALREFKDYIAKLIHDRRIKPKDDLISKLVHAEENGSKLSEKELYSMLFLLIV

AGLETTVNLLGSGTLALLQHKKECEKLKQHPEMIATAVEELLRYTSPVVMMANRWAIEDF

TYKGHSIKRGDMIFIGIGSANRDPNFFENPEILNINRSPNRHISFGFGIHFCLGAPLARL

EGHIAFNALLKRFPDIELAAAPDDIQWRKNVFLRGLESLPVSLSK

>CYP109B1 *Bacillus subtilis* subsp. *subtilis* RO-NN-1

MEHSLLNFFKGVTIMSVLNRRQALQRALLNGKNKQDAYHPFPWYESMRKDAPVSFDEENQ

VWSVFLYDDVKKVVGDKELFSSYMPQQTSSIGNSIINMDPPKHTKIRSVVNKAFTPRVMK

QWEPRIQEITDELIQKFQGRSEFDLVHDFSYPLPVIVISELLGVPSKHMDQFKAWSDLLV

STPKDKTEEAEKAFLEERDKCEEELAAFFAGIIEEKRNKPAQDIISILVEAEETGEKLSG

EELIPFCTLLLVAGNETTTNLISNAMYSILETPGVYEELRSHPELVPQAVEEALRFRAPA

PVLRRIAKRDTKIGGHLMKEGDMVLAFVASANRDEAKFDRPHMFDIRRHPNPHIAFGHGI

HFCLGAPLARLEANIALTSLISAFPHMECVSITPIENSVIYGLKSFRVKM

>CYP152A1 *Bacillus subtilis* subsp. *subtilis* RO-NN-1

MNEQIPHDKSLDNSLTLLKEGYLFIKNRTERYNSDLFQARLLGKNFICMTGADAAKVFYD

TDRFQRQNALPKRVQKSLFGVNAIQGMDGSAHIHRKMLFLSLMTPPHQKRLAELMTEEWK

AAVTRWEKADEVVLFEEAKEILCRVACYWAGVPLKETEVKERADDFIDMVDAFGAVGPRH

WKGRRARPRAEEWIEVMIEDARAGLLKTTPGTALHEMAFHKQEDGSQLDSRMAAIELINV

LRPIVAISYFLVFSALALHEHPKYKEWLRSGNSREREMFVQEVRRYYPFGPFLGALVKKD

FVWNNCEFKKGTSVLLDVYGTNHDPRLWDHPDEFRPERFAEREENPFDMIPQGGGHAEKG

HRCPGEGITIEVMKASLDFLVHQIEYDVPEQSLHYSLARMPSLPESGFVMSGIRRKS

>CYP102A38 *Bacillus velezensis* NAU-B3

MKETGPIPQPKTFGPLGNLPLLDKDKPTMSLIKLANEQGPIFQLHTPAGAIIVVSGHELV

KEVCDEERFDKSIEGALEKVRAFSGDGLFTSWTHEPNWRKAHNILMPTFSQRAMKDYHSM

MTDIAVQLIQKWARLNPDEAVDVPADMTRLTLDTIGLCGFNYRFNSYYRETPHPFINSMV

RALDEAMHQMQRLDVQDKLMIRTKRQFHHDIQAMFSLVDSIIAERRSGGRDEKDLLARML

NVEDPETGEKLDDKNIRFQIITFLIAGHETTSGLLSFAIYFLLKHPRVLDKAYEEADRVL

TDPVPSYKQVLDLTYIRMILQESLRLWPTAPAFSLYAKEDTVIGGKYPITPKDRISVLIP

QLHRDKDAWGDNAEEFYPERFEHPDQVPQHAYKPFGNGQRACIGMQFALHEATLVLGMIL

QHFTFIDHTDYELDIKQTLTIKPGDFHIRVRPRNKGAVAAALPAAEKAAGDVEKEKRETK

GASIIGLDNRPLLILYGSDTGTAEGVARELADTAGMHGVRTETAPLNDRIGKLPKEGALL

IITSSYNGKPPSNAGQFVQWLEEVKPGELEGVRYAVFGCGDHNWAATYQAVPRLIDEKLA

EKGAERFSSRGEGDVSGDFEGKLDEWKKSMWTDAMKAFGLKLNENAEKERSSLGLQFVSG

LGGSPLAKTYEAVYASVAENRELQAPESGRSTRHIEITLPKEAAYHEGDHLGVLPVNSKE

QVSRVLRRFNLNGNDQVLLTASGQSAAHLPLDRPVRLHDLLSSCVELQEAASRAQIREMA

AYTVCPPHKRELEDFLEEGVYQEQILTLRVSMLDLLEKYEACELPFERFLELLRPLKPRY

YSISSSPRKHPGQASITVGVVCGPARSGLGEYRGVASNYLADRGPEDGIVMFVRTPETRF

RLPEDPEKPIIMVGPGTGVAPFRGFLQARAALKKEGKELGEAHLYFGCRNDHDFIYRDEL

EAYEKDGIVTLHTAFSRKEGVPKTYVQHLMAKDAGALISILGRGGHLYVCGDGSKMAPDV

EATLQKAYQSVHETDERQAQEWLLDLQTKGIYAKDVWAGI

>CYP102A49 *Bacillus velezensis* NAU-B3

MKQLSAIPQPKTYGPLKNLPHLEKEKLSQSLWKIAEEYGPIFRFEFPSSVGVFVSGRELA

AEVCDEKRFDKNLSKALLKVREFGGDGLFTSWTHEKNWQKAHRILLPSFSQKAMKGYHSM

MLDIAMQLVQKWSRLNPNEEIDVAEDMTRLTLDTIGLCGFHYRFNSFYRDTQHPFITSML

RALQEAMRQSQRHSLQDKLMIKTRHQFQQDIEEMNSLVDRIIAERRENPDENLSDLLALM

LEAKDPVTGERLDDENIRYQIITFLIAGHETTSGLLSFAIYCLLKNKDKLKKAVQEAERV

LTGETPEYKQIQQLTYIRMVLNETLRLYPTAPAFSLYAKEDTVLGGKYPIEKGQPVTILT

PQLHRDKSAWGEDAESFRPERFSDPAAIPADAYKPFGNGQRACIGMQFALQEATMVLGLV

LKHFELIDHTDYELTIKEALTIKPGDFKIRVKNKDVSNHQPVQNHKAEGSGHKEETKEIP

SHGTPLLILYGSNLGTAEGIAEELADIGRSKGFSTETGPLDDYAGKLPVKGAVVIVTASY

NGAPPDNAAGFVKWMETLEDQELKGVSYAVFGCGDRNWAATYQRIPRLIDKVLEEKGAKR

LTSIGEGDNADDFEYSQEAWEDSFWKDIMKTFHIEAAPKQTNKSQLSIEYVSEAAETPIA

KTYKAFEAEVITNKELHTESSKRSVRHIELRLPETETYQEGDHLGVLPQNSGELISRVIR

RFGLDPNQHFKIKGRQLPHLPMDRPVNAPELLASYVELQEPATRAQLRELAAHTVCPPHQ

KELEHLYSDDAAYKENVLKKRMTMLDLLEDYPACELPFERFLELLPSLKARYYSISSSPK

AASGELSITVGVVTAPAWSGRGEYRGVASNYLAGLQNGDPAVCFIRSPQSGFALPENPKT

PLIMVGAGTGIAPFRGFIQARAAEKMSGNSLGEAHLYFGCRHPEEDDLYKDEFDDAEKNG

LVTVHRAYSRLDQDCKVYVQDVLLREAAQIIALLDQGGHLYICGDGSKMAPAVENVLLQA

YEKVHNTDSKVSLNWLEQLQAEGRYAKDVWAGM

>CYP107H4 *Bacillus velezensis* NAU-B3

MTAGLSIAHPSLHADSDFWNDPYPFYDKLRSIDPVYKGTVLKYPGWYVTGYKEAAAILKD

TRFKNRIPLPEASTKYQNLSHIQHDMLLFKNQSDHKRMRMLIGKEFTAKTAESLRPCIKE

TVHDLLDQVQIKKNADLVSEFAFPLASLIIAEILGVPKEERYQFRQWTADVIQAIDLTRS

RKTLVRASDTAGRLTSYFRDLIHKRKAHPQQDLISRFIMEEQLSKEEVLATCILLVIAGH

ETTVNLMCNGVFTLLKHPEQLSALRENPSLIETAVEECLRYDSPAQLTARTASEDCEING

KTIKKGEQVYILLGAANRDPSIFDQPHKMDIQRKPNPHLAFGKNAHFCIGSSLARIEAQI

AILTLFERMPKLRLAAHRLEYRKLIGFRSLKELPVVIG

>CYP107K3 *Bacillus velezensis* NAU-B3

MEKTMFHPHSPEFHENPFAVLSRFREQDPIHKFELQRFGGTFPAWLITRYDDCMAFLKDG

RITRDVKRVMPKELIAKLNVSEDIDFVSEHMLAKDPPDHSRLRSLVHQGFTPRMIEQLRT

GIEQITEELLDEMETKADPDIMRDFAAPLPFIVISELLGIPKEDRAKFQVWTNAMVDTSE

SGQDATNQALKEFKQYMKTLIEEKRKHPAEDLTSKLIYAEEDGQKLSESELYSMLFLLVV

AGLETTVNLLGSGTLALLLHKDQMEKIKRQPEDIQTAVEELLRYTSPVIMMANRWAIEDF

TYKDVSIKKGDMIFIGIGSANRDPEYFDDPDTLNIARTPNRHISFGFGIHFCLGAPLARM

EASIAFTALLKRFPNIELKGAPEDVTWRKNVFLRGLETLPVRF

>CYP109B7 *Bacillus velezensis* NAU-B3

MSKLRSTRQAIQRTLMKGKNGLDVYNPFPWYEKMRRESPIHFDEETKVWSVFLYDDVKKV

ISDKETFSSLMTDVKSSIAKSMLNMDPPKHTQIRSAVNRAFTPRVLKEWEPRIKDITDHL

LKQAKNKGRIDIVKDLSYPLPVMVISELLGVPSERMDQFKKWSDILVSMPKDASPEAAEK

NQQERDQCEAELAAFFAEIIESKRKKPGQDIISILIKEEEEGEKLTAEDLIPFCNLLLVA

GNETTTNLISNAVYSILETPGLYEELRQDPSLIAQTVEETLRFRAPAPFVRRTVRHDTEL

RGRRLKSGEIVLCYVASANRDENKFEKAGVFDIHRQSNPHLSFGFGVHFCLGAPLARLEA

EAALKGIVKTFSHLEPVGIEPIRNSVMYGLESLEAEINESEGEEKR

>CYP113L1 *Bacillus velezensis* NAU-B3

MTSLTKIRQQQPYKWYQTMRETSPVHYNEKEDCWEIFTYDEVKRVISDYSHFSSDHKYLS

ADKQEKMIRHINKDSLLKMDPPEHTVFRKLVNQPFMPKSVESLAPRIAAIADDLLQAVRS

KGRMDIIEDYAFPLPIIVIAELLGFPPKDRDIFKSWVDQSQNVKDEKKMNEVQKQMIGYF

MQFILQRRKQPQNDLISHLISADLDGEPLSDKQLIGFCGLLIVAGHVTTENVIGNSFLSL

KEFPHILPRLLENKALLPDFIEEVIRLRPSIQRVTRYTAVESEIGGKTIPAGEKVYAWIG

SANRDEKKFENADQIDLGRKPNQHLSFGQGSHYCLGAPLARLEAKIALSHFFEQMPAWRF

TEDQEPNLVPSPVFHGVDRLLVEF

>CYP152A4 *Bacillus velezensis* NAU-B3

MDEQIPHEKGLDNSLALLRDGYVFVKNRAETYRSDVFRARLLGKTFICMSGAEAAKLFYD

TERFQRQQALPKRVQKTLFGTGAIQAMDGERHKHRKLLFMSLMTPPRQKRLAEAVAKQWK

ASAEMWKGSNRIVLFDEAKKVLCRAACEWAGVPLKDSEVKERAEDFTDMVDAFGAVGPRH

WKGRRARLKTEKWIEEVIEDVRSGKLQTPEGSALYEMAFHTELDGNRLDSHMAAVELINV

LRPIAAISYFITFSALALHDYPEYRGKLRSRDDQEAERFAHEVRRYYPFAPFLGAVVKKD

FVWKNCEFKKGASVMLDLYGTNHDSRLWEDPNEFRPERFQGREENKFDFIPQGGGDPANG

HRCPGEGMTVEVMKTSLAFLANEIEYDVPSQDLSFSLSRMPALPESGFVISDVRRI

>CYP102A38 *Bacillus velezensis* TrigoCor1448

MKETGPIPQPKTFGPLGNLPLLDKDKPTMSLIKLANEQGPIFQLHTPAGAIIVVSGHELV

KEVCDEERFDKSIEGALEKVRAFSGDGLFTSWTHEPNWRKAHHILMPTFSQRAMKDYHSM

MTDIAVQLIQKWARLNPDEAVDVPADMTRLTLDTIGLCGFNYRFNSYYRETPHPFINSMV

RALDEAMHQMQRLDVQDKLMIRTKRQFHHDIQAMFSLVDSIIAERRSGGRDEKDLLARML

NAEDPETGEKLDDENIRFQIITFLIAGHETTSGLLSFAIYFLLKHPRVLEKAYEEADRVL

TDPVPSYKQVLDLTYIRMILQESLRLWPTAPAFSLYAKEDTVIGGKYPITPKDRISVLIP

QLHRDKDAWGDNAEEFYPERFEHPDQVPHHAYKPFGNGQRACIGMQFALHEATLVLGMIL

QHFTFIDHTDYELDIKQTLTIKPGDFHIRVRPRNKEDVAAALPAAEKAAEDVEKEKRETK

GASIIGLDNRPLLILYGSDTGTAEGVARELADTAGMHGVRTETAPLNDRIGKLPKEGALL

IITSSYNGKPPSNAGQFVQWLEEVKPGELEGVRYAVFGCGDHNWAATYQAVPRLIDEKLA

EKGAERFSSRGEGDVSGDFEGKLDEWKKSMWTDAMKAFGLKLNENAEKERSALGLQFVSG

LGGSPLAQTYEAVYASVAENRELQAPESGRSTRHIEITLPKEAAYHEGDHLGVLPVNSKE

QVSRVLRRFNLNGNDQVLLTASGQSAAHLPLDRPVRLHDLLSSCVELQEAASRAQIREMA

AYTVCPPHKRELEDFLEEGVYQEQILTSRVSMLDLLEKYEACELPFERFLELLRPLKPRY

YSISSSPRKHPGQASITVGVVRGPARSGLGEYRGVASNYLADRGPEDGIVMFVRTPETRF

RLPEDPEKPIIMVGPGTGVAPFRGFLQARAALKKEGKELGEAHLYFGCRNDHDFIYRDEL

EAYEKDGIVTLHTAFSRKEGVPKTYVQHLMAKDAGALISILGRGGHLYVCGDGSKMAPDV

EATLQKAYQSVRETDERQAQEWLLDLQTKGIYAKDVWAGI

>CYP102A49 *Bacillus velezensis* TrigoCor1448

MKQLSAIPQPKTYGPLKNLPHLEKEKLSQSLWKIAEEYGPIFRFEFPSSVGVFVSGRELA

AEVCDEKRFDKNLSKALLKVREFGGDGLFTSWTHEKNWQKAHRILLPSFSQKAMKGYHSM

MLDIAMQLVQKWSRLNPNEEIDVAEDMTRLTLDTIGLCGFHYRFNSFYRDTQHPFITSML

RALQEAMRQSQRHSLQDKLMIKTRHQFQQDIEEMNSLVDRIIAERRENPDENLSDLLALM

LEAKDPVTGERLDDENIRYQIITFLIAGHETTSGLLSFAIYCLLKNKDKLKKAVQEAERV

LTGETPEYKQIQQLTYIRMVLNETLRLYPTAPAFSLYAKEDTVLGGKYPIAKGQPVTILT

PQLHRDKSAWGEDAELFRPERFSDPAAIPADAYKPFGNGQRACIGMQFALQEATMVLGLV

LKHFELIDHTDYELTIKEALTIKPGDFKIRVKNKDVSNHQPVQNHKAEGSGHKEETKEIP

SHGTPLLILYGSNLGTAEGIAEELADIGRSKGFSTETGPLDDYAGKLPVKGAVVIVTASY

NGAPPDNAAGFVKWMETLEDQELKGVSYAVFGCGDRNWAATYQRIPRLIDKVLEEKGAKR

LTSIGEGDNADDFEYSQEAWEDSFWKDIMKAFHIEAAPKQTNKSQLSIEYVSEAAETPIA

KTYKAFEAEVITNKELHTESSKRSVRHIELRLPETETYQEGDHLGVLPQNSGELISRVIR

RFGLDPNQHFKIKGRQLPHLPMDRPVNAPELLASYVELQEPATRAQLRELAAHTVCPPHQ

KELEHLYSDDAAYKENVLKKRMTMLDLLEDYPACELPFERFLELLPSLKARYYSISSSPK

ATSGELSITVGVVTAPAWSGRGEYRGVASNYLAGLQKGDSAVCFIRSPQSGFALPENPKT

PLIMVGAGTGIAPFRGFIQARAAEKMSGNSLGEAHLYFGCRHPEEDDLYKDEFDHAEKNG

LVTVYRAYSRLDQDCKVYVQDVLLREAAQIIALLDQGGHLYICGDGSKMAPAVENVLLQA

YEKVHNTDSKVSLEWLEQLQAEGRYAKDVWAGM

>CYP107H4 *Bacillus velezensis* TrigoCor1448

MTADLSIAHPSLHADSDFWNDPYPFYDKLRSIDPVYKGTVLKYPGWYVTGYKEAAAILKD

TRFKNRIPLPEASTKYQNLSHIQHDMLLFKNQSDHKRMRMLIGKEFTAKTAESLRPCIKE

TVHDLLDQVQIKKTADLVSEFAFPLASLIIAEILGVPKEERYQFRQWTADVIQAIDLTRS

RKTLVRASDTAGRLTSYFRDLIHKREAHPQQDLISRFIMEEQLSKEEVLATCILLVIAGH

ETTVNLMSNGVFTLLKHPEQLSALRENPSLIETAVEECLRYDSPAQLTARTASEDCEING

KTIKKGEQVYILLGAANRDPSIFDQPHKMDIQRKPNPHLAFGRNAHFCIGSSLARIEAQI

AILTLFERMPKLRLAAHRLEYRKLIGFRSLKELPVVIG

>CYP107K3 *Bacillus velezensis* TrigoCor1448

MEKTMFHPHSPEFHENPFAVLSRFREQDPIHKFELQRFGGTFPAWLITRYDDCMAFLKDG

RITRDVKRVMPKELIAKLNVSEDIDFVSEHMLAKDPPDHSRLRSLVHQGFTPRMIEQLRT

GIEQITEELLDEMETKADPDIIRDFAAPLPFIVISELLGIPKEDRAKFQVWTNAMVDTSE

SGQDATNQALKEFKQYMKTLIEEKRKHPGEDLTSKLIYAEEDGQKLSESELYSMLFLLVV

AGLETTVNLLGSGTLALLLHKDQMEKIKRQPENIQTAVEELLRYTSPVIMMANRWAIEDF

TYKDVSIKKGDMIFIGIGSANRDPEYFDDPDTLNIARTPNRHISFGFGIHFCLGAPLARM

EASIAFTALLKRFPNIELKGAAEDVTWRKNVFLRGLETLPVRF

>CYP109B7 *Bacillus velezensis* TrigoCor1448

MSKLRSTRQAIQRTLMKGKNGLDVYNPFPWYEKMRRESPIHFDEETKVWSVFLYDDVKKV

ISDKETFSSLMTDVKSSIAKSMLNMDPPKHTQIRSAVNRAFTPRVLKEWEPRIKDITDHL

LKQAKNKGRIDIVKDLSYPLPVMVISELLGVPSERMDQFKKWSDILVSMPKDASPEAAEK

NQQERDQCEAELAAFFAEIIESKRKQPGQDIISILIKEEEEGEKLTAEDLIPFCNLLLVA

GNETTTNLISNAVYSILETPGLYEELRQDPSLIAQTVEETLRFRAPAPFVRRTVRHDTEL

RGRRLKSGEIVLCYVASANRDENKFEKAGVFDIHRQSNPHLSFGFGVHFCLGAPLARLEA

EVALKGIVKAFSHLEPVRIEPIRNSVMYGLESLEAEINENEGEEK

>CYP113L1 *Bacillus velezensis* TrigoCor1448

MTSLTKIRQQQPYKWYQTMRETSPVHYNEKEDCWEIFTYDEVKRVISDYSHFSSDHKYLS

ADKQEKMIRHINKDSLLKMDPPEHTVFRKLVNQPFMPKSVESLEPRIAAIADDLLQAVRS

KGRMDIIEDYAFPLPIIVIAELLGFPPKDRDIFKSWVDQSQNVKDEKKMNEVQKQMIGYF

MQFILQRRKQPQNDLISHLISADLDGEPLSDKQLIGFCGLLIVAGHVTTENVIGNSFLSL

KEFPHILPRLLENKALLPDFIEEVIRLRPSIQRVTRYTAVESEIGGKTIPAGEKVYAWIG

SANRDEKKFENADQIDLGRKPNQHLSFGQGSHYCLGAPLARLEAKIALSHFFEQVPAWRF

TEDQEPNLVPSPVFHGVDRLLVEF

>CYP152A4 *Bacillus velezensis* TrigoCor1448

MGEQIPHEKGLDNSLALLRDGYVFVKNRAENYRSDVFRARLLGKTFICMSGAEAAKLFYD

TERFQREHALPKRVQKTLFGTGAIQSMDGERHKHRKLLFMSLLTPPRQKRLAEAVAKQWK

ASAEMWEGSNRIVLFDEAKKVLCRAACEWAGVPLKDSEVKERAEDFTDMVDAFGAVGPRH

WKGRRARLKTEKWIEEVIEGVRSGKLQTPECSALYEMAFHTELDGNRLDSHMAAVELINV

LRPIAAISYFITFSALALHDYPEYRGKLRSRDDQEAERFAHEVRRYYPFAPFLGAVVKKD

FVWKNCEFKKGASVMLDLYGTNHDSRLWENPNEFRPERFQGREENKFEFIPQGGGDPANG

HRCPGEGMTVEVMKTSLAFLANEIEYDVPSQDLSFSLSRMPALPESGFVISDVRRI

>CYP102A38 *Bacillus velezensis* UCMB5033

MLERVLFMKETGPIPQPKTFGPLGNLPLLDKDKPTMSLIKLANEQGPIFQLHTPAGAIIV

VSGHELVKEVCDEERFDKSIEGALEKVRAFSGDGLFTSWTHEPNWRKAHNILMPTFSQRA

MKDYHSMMTDIAVQLIQKWARLNPDEAVDVPADMTRLTLDTIGLCGFNYRFNSYYRETPH

PFINSMVRALDEAMHQMQRLDVQDKLMIRTKRQFHHDIQAMFSLVDSIIAERRSGGRDEK

DLLARMLNVEDPETGEKLDDENIRFQIITFLIAGHETTSGLLSFAIYFLLKHPRVLEKAY

EEADRVLTDPVPSYKQVLDLTYIRMILQESLRLWPTAPAFSLYAKEDTVIGGKYPITPKD

RISVLIPQLHRDKDAWGDNAEEFYPERFEHPDQVPHHAYKPFGNGQRACIGMQFALHEAT

LVLGMILQHFTFIDHTDYELDIKQTLTIKPGDFHIRVRPRNKEAVAAALPAAEKAAEDVE

KEKRETKGASIIGLDNRPLLILYGSDTGTAEGVARELADTAGMHGVRTETAPLNDRIGKL

PKEGALLIITSSYNGKPPSNAGQFVQWLEEVKPGELEGVRYAVFGCGDHNWAATYQAVPR

LIDEKLAEKGAERFSSRGEGDVSGDFEGKLDEWKKSMWTDAMKAFGLKLNENAEKERSAL

GLQFVSGLGGSPLAQTYEAVYASVAENRELQAPESGRSTRHIEITLPKEAAYHEGDHLGV

LPVNSKEQVSRVLRRFNLNGNDQVLLTASGQSAAHLPLDRPVRLHDLLSSCVELQEAASR

AQIREMAAYTVCPPHKRELEDFLEEGVYQEQILTSRVSMLDLLEKYEACELPFERFLELL

RPLKPRYYSISSSPRKHPGQASITVGVVRGPARSGLGEYRGVASNYLADRGPEDGIVMFV

RTPETRFRLPEDPEKPIIMVGPGTGVAPFRGFLQARAALKKEGKELGEAHLYFGCRNDHD

FIYRDELEAYEKDGIVTLHTAFSRKEGVPKTYVQHLMAKDAGALISILGRGGHLYVCGDG

SKMAPDVEATLQKAYQSVHETDERQAQEWLLDLQTKGIYAKDVWAGI

>CYP102A49 Bacillus velezensis UCMB5033

MKQLSAIPQPKTYGPLKNLPHLEKEKLSQSLWKIAEEYGPIFRFEFPSSVGVFVSGRELA

AEVCDEKRFDKNLSKALLKVREFGGDGLFTSWTHEKNWQKAHRILLPSFSQKAMKGYHSM

MLDIAMQLVQKWSRLNPNEEIDVAEDMTRLTLDTIGLCGFHYRFNSFYRDTQHPFITSML

RALQEAMRQSQRHSLQDKLMIKTRHQFQQDIEEMNSLVDRIIAERRENPDENLSDLLALM

LEAKDPVTGERLDDENIRYQIITFLIAGHETTSGLLSFAIYCLLKNKDKLKKAVQEAERV

LTGETPEYKQIQQLTYIRMVLNETLRLYPTAPAFSLYAKEDTVLGGKYPIAKGQPVTILT

PQLHRDKSAWGEDAESFRPERFSDPAAIPADAYKPFGNGQRACIGMQFALQEATMVLGLV

LKHFELIDHTDYELTIKEALTIKPGDFKIRVKNKDVSNHQPVQNHKAEGSGHKEETKEIP

SHGTPLLILYGSNLGTAEGIAEELADIGRSKGFSTETGPLDDYAGKLPVKGAVVIVTASY

NGAPPDNAAGFVKWMETLEDQELKGVSYAVFGCGDRNWAATYQRIPRLIDKVLEEKGAKR

LTSIGEGDNADDFEYSQEAWEDSFWKDIMKAFHIEAAPKQTNKSQLSIEYVSEAAETPIA

KTYKAFEAEVITNKELHTESSKRSVRHIELRLPETETYQEGDHLGVLPQNSGELISRVIR

RFGLDPNQHFKIKGRQLPHLPMDRPVNAPELLASYVELQEPATRAQLRELAAHTVCPPHQ

KELEHLYSDDAAYKENVLKKRMTMLDLLEDYPACELPFERFLELLPSLKARYYSISSSPK

ATSGELSITVGVVTAPAWSGRGEYRGVASNYLAGLQKGDSAVCFIRSPQSGFALPENPKT

PLIMVGAGTGIAPFRGFIQARAAEKMSGNSLGEAHLYFGCRHPEEDDLYKDEFDHAEKNG

LVTVHRAYSRLDQDCKVYVQDVLLREAAQIIALLDQGGHLYICGDGSKMAPAVENVLLQA

YEKVHNTDSKVSLEWLEQLQAEGRYAKDVWAGM

>CYP107H4 *Bacillus velezensis* UCMB5033

MTADLSIVHPSLHADSDFWNDPYPFYDKLRSIDPVYKGTVLKYPGWYVTGYKEAAAILKD

IRFKNRIPLPEASTKYQNLSHIQHDMLLFKNQSDHKRMRMLIGKEFTAKTAESLRPCIKE

TVHDLLDQVQIKKTADLVSEFAFPLASLIIAEILGVPKEERYQFRQWTADVIQAIDLTRS

RKTLVRASDTAGRLTSYFKDLIHKRKAHPQQDLISRFIMEEQLSKEEVLATCILLVIAGH

ETTVNLISNGVFTLLKHPEQLSALRENPSLIETAVEECLRYDSPAQLTARTASEDCEING

KTIKKGEQVYILLGAANRDPSIFDQPHKMDIQRKPNPHLAFGKNAHFCIGSSLARIEAQI

AILTLFERMPKLRLAAHRLEYRKLIGFRSLKELPVVIG

>CYP107K3 *Bacillus velezensis* UCMB5033

MEKTMFHPHSPEFHENPFAVLSRFREQDPIHKFELQRFGGTFPAWLITRYDDCMAFLKDG

RITRDVKRVMPKELIAKLNVSEDIDFVSEHMLAKDPPDHSRLRSLVHQGFTPRMIEQLRT

GIEQITEELLDEMETKADPDIMRDFAAPLPFIVISELLGIPKEDRAKFQVWTNAMVDTSE

SGQDATNQALKEFKQYMKTLIEEKRKHPGEDLTSKLIYAEEDGQKLSESELYSMLFLLVV

AGLETTVNLLGSGTLALLLHKDQMEKIKRQPENIQTAVEELLRYTSPVIMMANRWAIEDF

TYKDVSIKKGDMIFIGIGSANRDPEYFDDPDTLNIARTPNRHISFGFGIHFCLGAPLARM

EASIAFTALLKRFPNIELKGAPEDVTWRKNVFLRGLETFPVRF

>CYP109B7 *Bacillus velezensis* UCMB5033

MSKLRSTRQAIQRTLMKGKNGLDVYNPFPWYEKMRRESPIHFDEETKVWSVFLYDDVKKV

ISDKETFSSLMTDVKSSIAKSMLNMDPPKHTQIRSAVNRAFTPRVLKEWEPRIKDITDHL

LKQAKNKGRIDIVKDLSYPLPVMVISELLGVPSERMDQFKKWSDILVSMPKDASPEAAEK

NQQERDQCEAELAAFFAEIIESKRKQPGQDIISILIKEEEEGEKLTAEDLIPFCNLLLVA

GNETTTNLISNAVYSILETPGLYEELRQDPSLIAQTVEETLRFRAPAPFVRRTVRHDTEL

RGRRLKSGEIVLCYVASANRDENKFEKAGVFDIHRQSNPHLSFGFGVHFCLGAPLARLEA

EVALKGIVKAFSHLEPVQIEPIRNSVMYGLESLEAEINENEGEEK

>CYP152A4 *Bacillus velezensis* UCMB5033

MNEQIPHDKGLDNSLALLRDGYVFVKNRAENYRSDVFRARLLGKTFICMSGAEAAKLFYD

TERFQREHALPKRVQKTLFGTGAIQSMDGERHKHRKLLFMSLMTPPRQKRLAEAVAKQWK

ASAEKWEGSNRIVLFDEAKKVLCRAACEWAGVPLKDSEVKERAEDFTDMVDAFGAVGPRH

WKGRRARLKTEKWIEEVIEDVRSGKLQTPECSALYEMAFHTELDGNRLDSHMAAVELINV

LRPIAAISYFITFSALALHDYPEYRGKLGSRDDQEAERFAHEVRRYYPFAPFLGAVVKKD

FVWKNCEFKKGASVMLDLYGTNHDSRLWENPNEFRPERFQGREENKFDFIPQGGGDPANG

HRCPGEGMTVEVMKTSLAFLANEIEYDVPSQDLSFSLSRMPALPESGFVISDVRRI

>CYP113L1 *Bacillus velezensis* UCMB5033

MTSLTKIRQQQPYKWYQTMRETSPVHYNEKEDCWEIFTYDEVKRVISDYSHFSSDHKYLS

ADKQEKMIRHINKDSLLKMDPPEHTVFRKLVNQPFMPKSVESLAPRIAAIADDLLQAVRS

KDRMDIIEDYAFPLPIIVIAELLGFPPKDRDIFKSWVDQSQNVKDEKKMNEVQKQMIGYF

MQFILQRRKQPQNDLISHLISADLDGEPLSDKQLIGFCGLLIVAGHVTTENVIGNSFLSL

KEFPHILPRLLENKALLPDFIEEVIRLRPSIQRVTRYTAVESEIGGKTIPAGEKVYAWIG

SANRDEKKFRNADQIDLGRKPNQHLSFGQGSHYCLGAPLARLEAKIALSHFFEQMPAWRF

TEDQEPNLVPSPVFHGVDRLLVEF

>CYP102A38 *Bacillus velezensis* UCMB5036

MLERVLFMKETGPIPQPKTFGPLGNLPLLDKDKPTMSLIKLANEQGPIFQLHTPAGAIIV

VSGHELVKEVCDEERFDKSIEGALEKVRAFSGDGLFTSWTHEPNWRKAHHILMPTFSQRA

MKDYHSMMTDIAVQLIQKWARLNPDEAVDVPADMTRLTLDTIGLCGFNYRFNSYYRETPH

PFINSMVRALDEAMHQMQRLDVQDKLMIRTKRQFHHDIQAMFSLVDSIIAERRSGGRDEK

DLLARMLNVEDPETGEKLDDENIRFQIITFLIAGHETTSGLLSFAIYFLLKHPRVLEKAY

EEADRVLTDPVPSYKQVLDLTYIRMILQESLRLWPTAPAFSLYAKEDTVIGGKYPITPKD

RISVLIPQLHRDKDAWGDNAEEFYPERFEHPDQVPHHAYKPFGNGQRACIGMQFALHEAT

LVLGMILQHFTFIDHTDYELDIKQTLTIKPGDFHIRVRPRNKEAVAAALPAAEKAAEDVE

KEKRETKGASIIGLDNRPLLILYGSDTGTAEGVARELADTAGMHGVRTETAPLNDRIGKL

PKEGALLIITSSYNGKPPSNAGQFVQWLEEVKPGELEGVRYAVFGCGDHNWAATYQAVPR

LIDEKLAEKGAERFSSRGEGDVSGDFEGKLDEWKKSMWTDAIKAFGLKLNENAEKERSAL

GLQFVSGLGGSPLAQTYEAVYASVAENRELQAPESGRSTRHIEITLPKEAAYHEGDHLGV

LPVNSKEQVSRVLRRYNLNGNDQVLLTASGQSAAHLPLDRPVRLHDLLSSCVELQEAASR

AQIREMAAYTVCPPHKRELEDFLEEGFYQEQILTSRVSMLDLLEKYEACELPFERFLELL

RPLKPRYYSISSSPRKHPGQASITVGVVRGPARSGLGEYRGVASNYLADRGPEDGIVMFV

RTPETRFRLPEDPEKPIIMVGPGTGVAPFRGFLQARAALKKEGKELGEAHLYFGCRNDHD

FIYRDELEAYEKDGIVTLHTAFSRKEGVPKTYVQHLMAKDAGALISILGRGGHLYVCGDG

SKMAPDVEATLQKAYQSVHETDERQAQEWLLNLQTKGIYAKDVWAGI

>CYP102A49 *Bacillus velezensis* UCMB5036

MKQLSAIPQPKTYGPLKNLPHLEKEKLSQSLWKIAEEYGPIFRFEFPSSVGVFVSGRELA

AEVCDEKRFDKNLSKALLKVREFGGDGLFTSWTHEKNWQKAHRILLPSFSQKAMKGYHSM

MLDIAMQLVQKWSRLNPNEEIDVAEDMTRLTLDTIGLCGFHYRFNSFYRDTQHPFITSML

RALQEAMRQSQRHSLQDKLMIKTRHQFQQDIEEMNSLVDRIIAERRENPDENLSDLLALM

LEAKDPVTGERLDDENIRYQIITFLIAGHETTSGLLSFAIYCLLKNKDKLKKAVQEAERV

LTGETPEYKQIQQLTYIRMVLNETLRLYPTAPAFSLYAKEDTVLGGKYPIAKGQPVTILT

PQLHRDKSAWGEDAELFRPERFSDPAAIPADAYKPFGNGQRACIGMQFALQEATMVLGLV

LKHFELIDHTDYELTIKEALTIKPGDFKIRVKNKDVSNHQPVQNHKAEGSGHKEETKEIP

SHGTPLLILYGSNLGTAEGIAEELADIGRSKGFSTETGPLDDYAGKLPVKGAVVIVTASY

NGAPPDNAAGFVKWMETLEDQELKGVSYAVFGCGDRNWAATYQRIPRLIDKVLEEKGAKR

LTSIGEGDNADDFEYSQEVWEDSFWKDIMKAFHIEAAPKQTNKSQLSIEYVSEAAETPIA

KTYKAFEAEVITNKELHTESSKRSVRHIELRLPETETYQEGDHLGVLPQNSGELISRVIR

RFGLDPNQHFKIKGRQLPHLPMDRPVNAPELLASYVELQEPATRAQLRELAAHTVCPPHQ

KELEHLYSDDAAYKENVLKKRMTMLDLLEDYPACELPFERFLELLPSLKARYYSISSSPK

AASGELSITVGVVTAPAWSGRGEYRGVASNYLAGLQKGDSAVCFIRSPQSGFALPENPKT

PLIMVGAGTGIAPFRGFIQARAAEKMSGNSLGEAHLYFGCRHPEEDDLYKDEFDHAEKNG

LVTVHRAYSRLNQDCKVYVQDVLLREAAQIIALLDQGGHLYICGDGSKMAPAVENVLLQA

YEKVHNTDSKVSLEWLEQLQAEGRYAKDVWAGM

>CYP107H4 *Bacillus velezensis* UCMB5036

MTADLSIAHPSLHADSDFWNDPYPFYDKLRSSDPVYKGTVLKYPGWYVTGYKEAAAILKD

IRFKNRIPLPEASTKYQNLSHIQHDMLLFKNQSDHKRMRMLIGKEFTAKTAESLRPCIKE

TVHDLLDQVQIKKNADLVSEFAFPLASLIIAEILGVPKEERYQFRQWTADVIHAIDLTRS

RKTLVRASDTAGRLTSYFRDLIHKRKAHPQQDLISRFIMEEQLSKEEVLATCILLVIAGH

ETTVNLISNGVFTLLKHPEQLSALRENPSLIETAVEECLRYDSPTQLTARTASEDCEING

KTIKKGEQVYILLGAANRDPSIFDQSHKMDIQRKPNPHLAFGKNAHFCIGSSLARIEAQI

AILTLFERMPKLRLAAHRLEYRKLIGFRSLKELPVVIG

>CYP107K3 *Bacillus velezensis* UCMB5036

MEKTMFHPHSPEFHENPFAVLSRFRAQDPIHKFELQRFGGTFPAWLITRYDDCMAFLKDG

RITRDVKRVMPKELIAKLNVSEDIDFVSDHMLAKDPPDHSRLRSLVHQGFTPRMIEQLRT

GIEQITEELLDEMETKADPDIMRDFAAPLPFIVISELLGIPKEDRAKFQVWTNAMVDTSE

SGQDATNQALKEFKQYMKTLIEEKRKHPGEDLTSKLIYAEEDGQKLSESELYSMLFLLVV

AGLETTVNLLGSGTLALLLHKDQMGKIKRQPENIQTAVEELLRYTSPVIMMANRWAIEDF

TYKDVSIKKGDMIFIGIGSANRDPEYFDDPDTLNIARTPNRHISFGFGIHFCLGAPLARM

EASIAFTALLKRFPNIELKGAPEDVTWRKNVFLRGLETLPVRF

>CYP109B7 *Bacillus velezensis* UCMB5036

MSKLRSTRQAIQRTLMKGKNGLDVYNPFPWYEKMRRESPIHFDEETKVWSVFLYDDVKKV

ISDKETFSSLMTDVKSSIAKSMLNMDPPKHTQIRSAVNRAFTPRVLKEWEPRIKDITDHL

LKQAKNKGRIDIVKDLSYPLPVMVISELLGVPSERMDQFKKWSDILVSMPKDASPEAAEK

NQQERDQCEAELAAFFAEIIESKRKQPGQDIISILIKEEEEGEKLTAEDLIPFCNLLLVA

GNETTTNLISNAVYSILETPGLYDELRQDPSLIAQTVEETLRFRAPAPFVRRTVRHDTEL

RGRRLKSGEIVLCYVASANRDENKFEKAGVFDIHRQSNPHLSFGFGVHFCLGAPLARLEA

EVALKGIVKAFSHLEPVRIEPIRNSVMYGLESLEAEINENEGEEK

>CYP152A4 *Bacillus velezensis* UCMB5036

MNEQIPHDKGLDNSLALLRDGYVFVKNRAENYRSDVFRARLLGKTFICMSGAEAAKLFYD

TERFQREHALPKRVQKTLFGTGAIQAMDGERHKHRKLLFMSLMTPPRQKRLAEAVAKQWK

ASAEKWEGSNRIVLFDEAKKVLCRAACEWAGVPLKDSEVKERAEDFTDMVDAFGAVGPRH

WKGRRARLKTEKWIEEVIEDVRSGKLQTPECSALYEMAFHTELDGNRLDSHMAAVELINV

LRPIAAISYFITFSALALHDYPEYRCKLRSRDDQEAERFAHEVRRYYPFAPFLGAVVKKD

FVWKNCEFKKGASVMLDLYGTNHDSRLWEDPNEFRPERFQGREENKFDFIPQGGGDPANG

HRCPGEGMTVEVMKTSLAFLANEIEYGVPSQDLSFSLSRMPALPESGFVISDVRRI

>CYP113L1 *Bacillus velezensis* UCMB5036

MTSLTKIRQQQPYKWYQTMRETSPVHYNEKEDCWEIFTYDEVKRVISDYSHFSSDHKYLS

ADKQEKMIRHINKDSLLKMDPPEHTVFRKLVNQPFMPKSVESLAPRIAAIADDLLQAVRS

KGRMDIIEDYAFPLPIIVIAELLGFPPKDRDIFKSWVDQSQNVKDEKKMNEVQKQMIGYF

MQFILQRRKQPQNDLISHLISADLDGEPLSDKQLIGFCGLLIVAGHVTTENVIGNSFLSL

KEFPHILPRLLENKALLPDFIEEVIRLRPSIQRVTRYTAVESEIGGKTIPAGEKVYAWIG

SANRDEKKFENADQIDLGRKPNQHLSFGQGSHYCLGAPLARLEAKIALSHFFEQMSAWRF

TEDQEPNLVPSPVFHGVDRLLVEF

>CYP102A38 *Bacillus velezensis* UCMB5113

MLERVLFMKETGPIPQPKTFGPLGNLPLLDKDKPTMSLIKLANEQGPIFQLHTPAGAIIV

VSGHELVKEVCDEERFDKSIEGALEKVRAFSGDGLFTSWTHEPNWRKAHNILMPTFSQRA

MKDYHSMMTDIAVQLIQKWARLNPDEAVDVPADMTRLTLDTIGLCGFNYRFNSYYRETPH

PFINSMVRALDEAMHQMQRLDVQDKLMIRTKRQFHHDIQAMFSLVDSIIAERRSGGRDEK

DLLARMLNVEDPETGEKLDDENIRFQIITFLIAGHETTSGLLSFAIYFLLKHPRVLEKAY

EEADRVLTDPVPSYKQVLDLTYIRMILQESLRLWPTAPAFSLYAKEDTVIGGKYPITPKD

RISVLIPQLHRDKDAWGDNAEEFYPERFEHPDQVPHHAYKPFGNGQRACIGMQFALHEAT

LVLGMILQHFTFIDHTDYELDIKQTLTIKPGDFHIRVRPRNKEAVAAALPAAEKAAEDVE

KEKRETKGASIIGLDNRPLLILYGSDTGTAEGVARELADTAGMHGVRTETAPLNDRIGKL

PKEGALLIITSSYNGKPPSNAGQFVQWLEEVKPGELEGVRYAVFGCGDHNWAATYQAVPR

LIDEKLAEKGAERFSSRGEGDVSGDFEGKLDEWKKSMWTDAMKAFGLKLNENAEKERSAL

GLQFVSGLGGSPLAQTYEAVYASVAENRELQAPESGRSTRHIEITLPKEAAYHEGDHLGV

LPVNSKEQVSRVLRRFNLNGNDQVLLTASGQSAAHLPLDRPVRLHDLLSSCVELQEAASR

AQIREMAAYTVCPPHKRELEDFLEEGVYQEQILTSRVSMLDLLEKYEACELPFERFLELL

RPLKPRYYSISSSPRKHPGQASITVGVVRGPARSGLGEYRGVASNYLADRGPEDGIVMFV

RTPETRFRLPEDPEKPIIMVGPGTGVAPFRGFLQARAALKKEGKELGEAHLYFGCRNDHD

FIYRDELEAYEKDGIVTLHTAFSRKEGVPKTYVQHLMAKDAGALISILGRGGHLYVCGDG

SKMAPDVEATLQKAYQSVHETDERQAQEWLLDLQTKGIYAKDVWAGI

>CYP102A49 *Bacillus velezensis* UCMB5113

MKQLSAIPQPKTYGPLKNLPHLEKEKLSQSLWKIAEEYGPIFRFEFPSSVGVFVSGRELA

AEVCDEKRFDKNLSKALLKVREFGGDGLFTSWTHEKNWQKAHRILLPSFSQKAMKGYHSM

MLDIAMQLVQKWSRLNPNEEIDVAEDMTRLTLDTIGLCGFHYRFNSFYRDTQHPFITSML

RALQEAMRQSQRHSLQDKLMIKTRHQFQQDIEEMNSLVDRIIAERRENPDENLSDLLALM

LEAKDPVTGERLDDENIRYQIITFLIAGHETTSGLLSFAIYCLLKNKDKLKKAVQEAERV

LTGETPEYKQIQQLTYIRMVLNETLRLYPTAPAFSLYAKEDTVLGGKYPIAKGQPVTILT

PQLHRDKSAWGEDAELFRPERFSDPAAIPADAYKPFGNGQRACIGMQFALQEATMVLGLV

LKHFELIDHTDYELTIKEALTIKPGDFKIRVKNKDVSNHQPVQNHKAEGSGHKEETKEIP

SHGTPLLILYGSNLGTAEGIAEELADIGRSKGFSTETGPLDDYAGKLPVKGAVVIVTASY

NGAPPDNAAGFVKWMETLEDQELKGVSYAVFGCGDRNWAATYQRIPRLIDKVLEEKGAKR

LTSIGEGDNADDFEYSQEAWEDSFWKDIMKAFHIDIEAAPKQANKSQLSIEYVSEAAETP

IAKTYKAFEAEVITNKELHTESSKRSVRHIELRLPETETYQEGDHLGVLPQNSGELISRV

IRRFGLDPNQHFKIKGRQLPHLPMDRPVNAPELLASYVELQEPATRAQLRELAAHTVCPP

HQKELEHLYSDDAAYKENVLKKRMTMLDLLEDYPACELPFERFLELLPSLKARYYSISSS

PKATSGELSITVGVVTAPAWSGRGEYRGVASNYLAGLQKGDSAVCFIRSPQSGFALPENP

KTPLIMVGAGTGIAPFRGFIQARAAEKMSGNSLGEAHLYFGCRHPEEDDLYKDEFDHAEK

NGLVTVHRAYSRLDQDCKVYVQDVLLREAAQIIALLDQGGHLYICGDGSKMAPAVENVLL

QAYEKVHNTDSKVSLEWLEQLQAEGRYAKDVWAGM

>CYP107H4 *Bacillus velezensis* UCMB5113

MTADLSIAHPSLHADSDFWNDPYPFYDKLRSIDPVYKGTVLKYPGWYVTGYKEAAAILKD

IRFKNRIPLPEASTKYQNLSHIQHDMLLFKNQSDHKRMRMLIGKEFTAKTAESLRPCIKE

TVHDLLDQVQIKKTADLVSEFAFPLASLIIAEILGVPKEERYQFRQWTADVIQAIDLTRS

RKTLVRASDTAGRLTSYFKDLIHKRKAHPQQDLISRFIMEEQLSKEEVLATCILLVIAGH

ETTVNLISNGVFTLLKHPEQLSALRENPSLIETAVEECLRYDSPAQLTARTASEDCEING

KTIKKGEQVYILLGAANRDPSIFDQPHKMDIQRKPNPHLAFGKNAHFCIGSSLARIEAQI

AILTLFERMPKLRLAAHRLEYRKLIGFRSLKELPVVIG

>CYP107K3 *Bacillus velezensis* UCMB5113

MEKTMFHPHSPEFHENPFAVLSRFREQDPIHKFELQRFGGTFPAWLITRYDDCMAFLKDG

RITRDVKRVMPKELIAKLNVSEDIDFVSEHMLAKDPPDHSRLRSLVHQGFTPRMIEQLRT

GIEQITEELLDEMETKADPDIMRDFAAPLPFIVISELLGIPKEDRAKFQVWTNAMVDTSE

SGQDATNQALKEFKQYMKTLIEEKRKHPGEDLTSKLIYAEEDGQKLSESELYSMLFLLVV

AGLETTVNLLGSGTLALLLHKDQMEKIKRQPENIQTAVEELLRYTSPVIMMANRWAIEDF

TYKDVSIKKGDMIFIGIGSANRDPEYFDDPDTLNIARTPNRHISFGFGIHFCLGAPLARM

EASIAFTALLKRFPNIELKGAPEDVTWRKNVFLRGLETLPVRF

>CYP109B7 *Bacillus velezensis* UCMB5113

MSKLRSTRQAIQRTLMKGKNGLDVYNPFPWYEKMRRESPIHFDEETKVWSVFLYDDVKKV

ISDKETFSSLMTDVKSSIAKSMLNMDPPKHTQIRSAVNRAFTPRVLKEWEPRIKDITDHL

LKQAKNKGRIDIVKDLSYPLPVMVISELLGVPSERMDQFKKWSDILVSMPKDASPEAAEK

NQQERDQCEAELAAFFAEIIESKRKQPGQDIISILIKEEEEGEKLTAEDLIPFCNLLLVA

GNETTTNLISNAVYSILETPGLYEELRQDPSLIAQTVEETLRFRAPAPFVRRTVRHDTEL

RGRRLKSGEIVLCYVASANRDENKFEKAGVFDIHRQSNPHLSFGFGVHFCLGAPLARLEA

EVALKGIVKAFSHLEPVRLSRSETVSCTGWSL

>CYP152A4 *Bacillus velezensis* UCMB5113

MNEQIPHDKGLDNSLALLRDGYVFVKNRAENYRSDVFRARLLGKTFICMSGAEAAKLFYD

TERFQREHALPKRVQKTLFGTGAIQSMDGERHKHRKLLFMSLMTPPRQKRLAEAVAKQWK

ASAEKWEGSNRIVLFDEAKKVLCRAACEWAGVPLKDSEVKERAEDFTDMVDAFGAVGPRH

WKGRRARLKTEKWIEEVIEDVRSGKLQTPECSALYEMAFHTELDGNRLDSHMAAVELINV

LRPIAAISYFITFSALALHDYPEYRGKLRSRDDQEAERFVHEVRRYYPFAPFLGAVVKKD

FVWKNCEFKKGASVMLDLYGTNHDSRLWENPNEFRPERFQGREENKFDFIPQGGGDSANG

HRCPGEGMTVEVMKTSLAFLANEIEYDVPSQDLSFSLSRMPALPESGFVISDVRRI

>CYP113L1 *Bacillus velezensis* UCMB5113

MTSLTKIRQQQPYKWYQTMRETSPVHYNEKEDCWEIFTYDEVKRVISDYSHFSSDHKYLS

ADKQEKMIRHINKDSLLKMDPPEHTVFRKLVNQPFMPKSVETLAPRIAAIADDLLQAVRS

KGRMDIIEDYAFPLPIIVIAELLGFPPKDRDIFKSWVDQSQNVKDEKKMNEVQKQMIGYF

MQFILQRRKQPQNDLISHLISADLDGEPLSDKQLIGFCGLLIVAGHVTTENVIGNSFLSL

KEFPHILPRLLENKALLPDFIEEVIRLRPSIQRVTRYTAVESEIGGKTIPAGEKVYAWIG

SANRDEKKFENADQIDLGRKPNQHLSFGQGSHYCLGAPLARLEAKIALSHFFEQMPAWRF

TEDQEPNLVPSPVFHGVDRLLVEF

>CYP102A38 *Bacillus velezensis* YAU B9601-Y2

MKETGPIPQPKTFGPLGNLPLLDKDKPTMSLIKLANEQGPIFQLHTPAGAIIVVSGHELV

KEVCDEERFDKSIEGALEKVRAFSGDGLFTSWTHEPNWRKAHNILMPTFSQRAMKDYHSM

MTDIAVQLIQKWARLNPDEAVDVPADMTRLTLDTIGLCGFNYRFNSYYRETPHPFINSMV

RALDEAMHQMQRLDVQDKLMIRTKRQFHHDIQAMFSLVDSIIAERRSGGRDEKDLLARML

NVEDPETGEKLDDENIRFQIITFLIAGHETTSGLLSFAIYFLLKHPRVLEKAYEEADRVL

TDPVPSYKQVLDLTYIRMILQESLRLWPTAPAFSLYAKEDTVIGGKYPITPKDRISVLIP

QLHRDKDAWGDNAEEFYPERFEHPDQVPHHAYKPFGNGQRACIGMQFALHEATLVLGMIL

QHFTFIDHTDYELDIKQTLTIKPGDFHIRVRPRNKGAVAAALPAAEKAAEDVEKEKRETK

GASIIGLDNRPLLILYGSDTGTAEGVARELADTAGMHGVRTETAPLNDRIGKLPKEGALL

IITSSYNGKPPSNAGQFVQWLEEVKPGELEGVRYAVFGCGDHNWAATYQAVPRLIDEKLA

EKGAERFSSRGEGDVSGDFEGKLDEWKKSMWTDAMKAFGLKLNENAEKERSSLGLQFVSG

LGGSPLAQTYEAVYASVAENRELQAPESGRSTRHIEITLPKEAAYHEGDHLGVLPVNSKE

QVSRVLRRFNLNGNDQVLLTASGQSAAHLPLDRPVRLHDLLSSCVELQEAASRAQIREMA

AYTVCPPHKRELEDFLEEGVYQEKILTLRVSMLDLLEKYEACELPFERFLELLRPLKPRY

YSISSSPRKHPGQASITVGVVRGPARSGLGEYRGVASNYLADRGPEDGIVMFVRTPETRF

RLPEDPEKPIIMVGPGTGVAPFRGFLQARAALKKEGKELGEAHLYFGCRNDHDFIYRDEL

EAYEKDGIVTLHTAFSRKEGVPKTYVQHLMAKDAGALISILGRGGHLYVCGDGSKMAPDV

EATLQKAYQSVHETDERQAQEWLLDLQTKGIYAKDVWAGI

>CYP102A49 *Bacillus velezensis* YAU B9601-Y2

MKQLSAIPQPKTYGPLKNLPHLEKEKLSQSLWKIAEEYGPIFRFEFPSSVGVFVSGRELA

AEVCDEKRFDKNLSKALLKVREFGGDGLFTSWTHEKNWQKAHRILLPSFSQKAMKGYHSM

MLDIAMQLVQKWSRLNPNEEIDVAEDMTRLTLDTIGLCGFHYRFNSFYRDTQHSFITSML

RALQEAMRQAQRHSLQDKLMIKTRHQFQQDIEEMNSLVDRIIAERRENPDENLSDLLALM

LEAKDPVTGERLDDENIRYQIITFLIAGHETTSGLLSFAIYCLLKNKDKLKKAVQEAERV

LTGETPEYKQIQQLTYIRMVLNETLRLYPTAPAFSLYAKEDTVLGGKYPIAKGQPVTILT

PQLHRDKSAWGEDAESFRPERFSDPAAIPADAYKPFGNGQRACIGMQFALQEATMVLGLV

LKHFELIDHTDYELTIKEALTIKPGDFKIRVKNKDVSNQQSAQNHKAEGSGHKEEMKEIP

SHGTPLLILYGSNLGTAEGIAEELADIGRSKGFSTETGPLDDYAGKLPVKGAVVIVTASY

NGAPPDNAAGFVKWMETLEDQELKGVSYAVFGCGDRNWAATYQRIPRLIDKVLEEKGAKR

LTSIGEGDNADDFEYSQEAWEDSFWKDIMKAFHIEAAPKQTNKSQLSIEYVSEAAETPIA

KTYKAFEAEVITNKELHTESSKRSVRHIELRLPETETYQEGDHLGVLPQNSGELISRVIR

RFGLDPNQHFKIKGRQLPHLPMDRPVNAPELLASYVELQEPATRAQLRELAAHTVCPPHQ

KELEHLYSDDAAYKENVLKKRMTMLDLLEDYPACELPFERFLELLPSLKARYYSISSSPK

AASGELSITVGVVTAPAWSGRGEYRGVASNYLAGLQNGDPAVCFIRSPQSGFALPENPKT

PLIMVGAGTGIAPFRGFIQARAAEKMSGNSLGEAHLYFGCRHPEEDDLYKDEFDDAEKNG

LVTVHRAYSRLDQDCKVYVQDVLLREAAQIIALLDQGGHLYICGDGSKMAPAVENVLLQA

YEKVHNTDSKVSLNWLEQLQAEGRYAKDVWAGM

>CYP107H4 *Bacillus velezensis* YAU B9601-Y2

MTAGLSIAHPSLHADSDFWNDPYSFYDKLRSIDPVYKGTVLKYPGWYVTGYKEAAAILKD

TRFKNRIPLPEASTKYQNLSHIQHDMLLFKNQSDHKRMRMLIGKEFTAKTAESLRPCIKE

TVHDLLDQVQIKKNADLVSEFAFPLASLIIAEILGVPKEERYQFRQWTADVIQAIDLTRS

RKTLVRASDTAGRLTSYFRDLIHKRKAHPQQDLISRFIMEEQLSKEEVLATCILLVIAGH

ETTVNLMCNGVFTLLKHPEQLSALRENPSLIETAVEECLRYDSPAQLTARTASEDCEING

KTIKKGEQVYILLGAANRDPSIFDQPHKMDIQRKPNPHLAFGKNAHFCIGSSLARIEAQI

AILTLFERMPKLRLAAHRLEYRKLIGFRSLKELPVVIG

>CYP107K3 *Bacillus velezensis* YAU B9601-Y2

MEKTMFHPHSPEFHENPFAVLSRFREQDPIHKFELQRFGGTFPAWLITRYDDCMAFLKDG

RITRDVKRVMPKELIAKLNVSEDIDFVSEHMLAKDPPDHSRLRSLVHQGFTPRMIEQLRT

GIEQITEELLDEMETKADPDIMRDFAAPLPFIVISELLGIPKEDRAKFQVWTNAMVDTSE

SGQDATNQALKEFKQYMKTLIEEKRKHPAEDLTSKLIYAEEDGQKLSESELYSMLFLLVV

AGLETTVNLLGSGTLALLLHKDQMEKIKRQPENIQTAVEELLRYTSPVIMMANRWAIEDF

TYKDVSIKKGDMIFIGIGSANRDPEYFDDSDTLNIARTPNRHISFGFGIHFCLGAPLARM

EASIAFTALLKRFPNIELKGAPEDVTWRKNVFLRGLETLPVRF

>CYP109B7 *Bacillus velezensis* YAU B9601-Y2

MSKLRSTRQAIQRTLMKGKNGLDVYNPFPWYEKMRRESPIHFDEETKVWSVFLYDDVKKV

ISDKETFSSLMTDVKSSIAKSMLNMDPPKHTQIRSAVNRAFTPRVLKEWEPRIKDITDHL

LKQAKNKGRIDIVKDLSYPLPVMVISELLGVPSEHMDQFKKWSDILVSMPKDASPEAAEK

NQQERDQCEAELAAFFAEIIESKRKKPGQDIISILIKEEEEGEKLTAEDLIPFCNLLLVA

GNETTTNLISNAVYSILETPGLYEELRQDPSLIAQTVEETLRFRAPAPFVRRTVRHDTEL

RGRRLKSGEIVLCYVASANRDENKFEKAGVFDIHRQSNPHLSFGFGVHFCLGAPLARLEA

EAALKGIVKTFSHLEPVGIEPIRNSVMYGLESLEAEINESEGEEKR

>CYP113L1 *Bacillus velezensis* YAU B9601-Y2

MTSLTKIRQQQPYKWYQTMRETSPVHYNEKEDCWEIFTYDEVKRVISDYSHFSSDHKYLS

ADKQEKMIRHINKDSLLKMDPPEHTVFRKLVNQPFMPKSVESLAPRIAAIADDLLQAVRS

KGRMDIIEDYAFPLPIIVIAELLGFPPKDRDIFKSWVDQSQNVKDEKKMNEVQKQMIGYF

MQFILQRRKQPQNDLISHLISADLDGEPLSDKQLIGFCGLLIVAGHVTTENVIGNSFLSL

KEFPHILPRLLENKALLPDFIEEVIRLRPSIQRVTRYTAVESEIGGKTIPAGEKVYAWIG

SANRDEKKFENADQIDLGRKPNQHLSFGQGSHYCLGAPLARLEAKIALSHFFEQMPAWRF

TEDQEPNLVPSPVFHGVDRLLVEF

>CYP152A4 *Bacillus velezensis* YAU B9601-Y2

MDEQIPHEKGLDNSLALLRDGYVFVKNRAETYRSDVFRARLLGKTFICMSGAEAAKLFYD

TERFQRQQALPKRVQKTLFGTGAIQAMDGERHKHRKLLFMSLMTPPRQKRLAEAVAKQWK

ASAEMWKGSNRIVLFDEAKKVLCRAACEWAGVPLKDSEVKERAEDFTDMVDAFGAVGPRH

WKGRRARLKTEKWIEEVIEDVRSGKLQTPEGSALYEMAFHTELDGNRLDSHMAAVELINV

LRPIAAISYFITFSALALHDYPEYRGKLRSRDDQEAERFAHEVRRYYPFAPFLGAVVKKD

FVWKNCEFKKGASVMLDLYGTNHDSRLWEDPNEFRPERFQGREENKFDFIPQGGGDPANG

HRCPGEGMTVEVMKTSLAFLANEIEYDVPSQDLSFSLSRMPALPESGFVISDVRRI

>CYP102A38 *Bacillus amyloliquefaciens* DSM 7

MKETSPIPKPKTFGPLGNLPLLDKDKPTMSLIKLAKEQGPIFQLHTPAGTIIVVSGHELV

KEVCDEERFDKSIEGALEKVRAFSGDGLFTSWTHEPNWRKAHNILMPTFSKRAMKDYHSM

MTDIAVQLIQKWARLNPDEAVDVPADMTRLTLDTIGLCGFNYRFNSYYRETPHPFINSMV

RALDEAMHQMQRLDVQDKLMIRTKRQFHHDIQAMFSLVDSIIAERRSDGGRDEKDLLARM

LNVEDPETGEKLDDENIRFQIITFLIAGHETTSGLLSFAIYFLLKHPRVLEKAYEEADRV

LTDPVPSYKQVLELTYIRMILQESLRLWPTAPAFSLYAKEDTVIGGKYPITTKDRISVLI

PQLHRDKDAWGDNAEEFYPERFEHPDQVPHHAYKPFGNGQRACIGMQFALHEATLVLGMI

LQHFTFTDHTDYELDIKQTLTLKPNDFHIRVRPRSKEAVPAAFTAAEKAAEDVTKEKQET

KGASIIGLDNRPLLILYGSDTGTAEGVAWELADTAGMHGVRTETAPLNDRIGKLPKEGAL

LIITSSYNGKPPSNAGQFVQWLEEVKQGELEGVRYAVFGCGDHNWAATYQAVPRLIDEKL

AEKGAERFSSRGEGDVSGDFEGKLDEWKKSMWKDAMKAFGLKLNENAEKERSTLGLQFVS

GLGGSPLAQTYEAVYASVAENRELQAPESDRSTRHIEIILPKEAAYNEGDHLGVLPVNSK

EQVSRVLRRYNLNGNDQVLLTASGQSAAHLPLDRPVRLHDLLSSCVELQEAASRAQIREM

AAYTVCPPHKHELEGLLEEGVYQEQILTLRVSMLDLLEKYEACELPFERFLELLRPLKPR

YYSISSSPLKHPGQASITVGVVRGPARSGLGEYRGVASNYLADRSPDDGIVMFVRTPETK

FRLPEDPEKPIIMVGPGTGVAPFRGFLQARAALKGEGKELGEAHLYFGCRNDYDFIYRDE

LEAYEKDGIVTLHTAFSRKEGVPKTYVQHLMAEDAETLISILDRGGHLYVCGDGSKMAPD

VEETLQKAYQSAHGTDERQAQEWLLDLQTKGIYAKDVWAGI

>CYP102A49 *Bacillus amyloliquefaciens* DSM 7

MKQLSAIPQPKTYGPLKNLPHLEKEKLSQSLWKIAEEYGPIFRFEFPSSVGVFVSGRELA

AEVCDEKRFDKNLSKALLKVREFGGDGLFTSWTHEKNWQKAHRILLPSFSQKAMKGYHSM

MLDIAMQLVQKWSRLNPNEEIDVAEDMTRLTLDTIGLCGFHYRFNSFYRDTQHPFITSML

RALQEAMRQSQRHSLQDKLMIKTRHQFQQDIEVMNELVDRIIAERRENPDENLSDLLALM

LEAKDPVTGERLDDENIRYQIITFLIAGHETTSGLLSFAIYCLLKNKDKLKKAIQEAERV

LTGETPEYKQIQQLTYIRMVLNETLRLYPTAPAFSLYAKEDTVLGGKYPIAKGQPVTILT

PQLHRDKSAWGEDAELFRPERFSDPAAIPADAYKPFGNGQRACIGMQFALQEATMVLGLV

LKHFELIDHTDYELTIKEALTIKPGDFKIRVKNKDVSNQQSAQVHKAEGGGHKEETKEIP

SHGTPLLILYGSNLGTAEGIAEELADIGRSKGFSSETGPLDDYAGKLPVKGAVVIVTASY

NGAPPDNAAGFVKWMETLEDQELKGVSYAVFGCGDRNWAATYQRIPRLIDKVLEEKGAKR

LTSIGEGDNADDFEYSQEAWEDSFWKDIMKAFHIEAAPKQTNKSQLSIEYVSEAAETPIA

KTYKAFEAEVTTNKELHTESSKRSVRHIELRLPETETYQEGDHLGVLPQNSGELISRVIR

RFGLDPNQHFKIKGRQLPHLPMDRPVNAPELLASYVELQEPATRAQLRELAAHTVCPPHQ

KELEHLYSDYAAYKENVLKKRMTMLDLLEDYPACELPFERFLELLPSLKARYYSISSSPK

ANKRELSITVGVVTAPAWSGRGEYRGVASNYLAGLQNGDSAVCFIRSPQSGFALPENTKT

PLIMIGAGTGIAPFRGFIQARAAEKMSGNSLGEAHLYFGCRHPEEDDLYKDEFDHAEKNG

LVTVHRAYSRLDQDCKVYVQDVLLREAAQIIALLDQGGHLYICGDGSKMAPAVENVLLQA

YEKVHNTDSKVSLNWLEQLQAEGRYAKDVWAGV

>CYP107H2 *Bacillus amyloliquefaciens* DSM 7

MTAGLSIAHPSLHADSDFWNNPYPFYDKLRAVDPVYQGTVLKYPGRYITGYQEAEAVLKD

TRFKNRIPMPEASAKYKHLKNLQKDMLLFTNHSDHKRLRMLIGKAFTLKKAERLKPFITA

TVHDVLDQIDHSKTADLVSDFAFPVASLVIADILGVPKEDRASFREWTADVIQAIDLTRS

KKSLLKAGGTAGKLTAYFKDLIQKRKTEPQKDVITTLISEEQLTEEEVLASCILLIIAGH

ETTVNLICNGVFSLLKHPAELSKLLENPQLIASATEEFLRFESPAQLTARTASEDCVINQ

HLIKKGEQVYILLGAANRDPEVFHRPHQLDITRNPNPHLAFGKGAHVCIGSSLARIEAQT

AILTLLERAPDIRLVKTDVTYRKLFGFRSLSALPVVLS

>CYP107K3 *Bacillus amyloliquefaciens* DSM 7

MEKIMFHPHSSEFHENPFAVLSRFREQDPIHKFELQRFGGTFPAWLITRYDDCMAFLKDG

RITRDVKRVMPKELIAKLNVSEDIDFVSEHMLAKDPPDHSRLRSLVHQGFTPRMIEQLRT

GIERITEELLDEMETKADPDIMRDFAAPLPFIVISELLGIPKEDRAKFQVWTSAMVDTSE

SGQDATNQALREFKQYMQTLIEEKRNHPGEDLTSKLIYAEEDGQKLSESELYSMLFLLVV

AGLETTVNLLGSGTLALLLHKDQLENIKRQPDTIQTAVEELLRYTSPVIMMANRWAIEDF

TYKDVSIKKGDMIFIGIGSANRDPEYFDEPDTLNVARTPNRHISFGFGIHFCLGAPLARM

EASIAFNALLKRFPNIELNGSSEDLTWRKNVFLRGLETLPVKF

>CYP109B3 *Bacillus amyloliquefaciens* DSM 7

MSNLRSPRQAIQRTLMKGKDGLDVYNPFPWYEKMRRESPIQFDEETKVWSVFLYDDAKKV

ISDKETFSSLMSDMKSSIAKSMLNMDPPKHTQIRSAVNRAFTPRVLKEWEPRIEEITDNL

LKQAKNKGRIDIVKDLSYPLPVIVISELLGVPSEHMDQFKKWSDILVSMPKDASPEEAEK

NQKERDQCETELAAFFAEIIDSKRKQPGQDIISILIKEEEEGEKLSAEDLIPFCNLLLVA

GNETTTNLISNAVYSILETPGLYEELRHDPSLIAQTVEETLRFRAPAPFVRRTVRHDTEL

CGRRLKSGDIVLCYIASANRDENKFEQADVFDIHRQSNPHLSFGFGVHFCLGAPLARLEA

EVALRGIVKAFSHLEPVRVEPIQNSVMYGLDSLEAEINENRGEEKR

>CYP152A4 *Bacillus amyloliquefaciens* DSM 7

MTEQIPHEKGLDNSLALLRDGYVFVKNRAENYRSDVFRARLLGKTFICMSGAEAAKLFYD

TERFQRQQALPKRVQKTLFGTGAIQSMDGERHKHRKLLFMSLMTPPRQKRLAEAVAKQWK

ASAEKWEGADRIVLFDEAKKVLCRAACEWAGVPLKDSEVKERAEDFTDMVDAFGAVGPRH

WKGRRARPKTEKWVEEVIEDVRSGKLQTPEGSALYEMAVHTELDGSRLDSHMAAVELINV

LRPIAAISYFIAFSALALHDHPEYRDKLRSGDDQEAERFVHEVRRYYPFAPFLGAVVKKD

FVWKNCEFKKGASVMLDLYGTNHDSRLWENPNEFRPERFQGREENKFDFIPQGGGDPADG

HRCPGEGMTVEVMKASLAFLTNEIEYDVPPQDLSFSLSRMPALPESGFVIIGDRVFSIPV

SCLPGGFFRQAGSFSD

>CYP102A38*Bacillus amyloliquefaciens* IT-45

MKETGPIPQPKTFGPLGNLPLLDKDKPTMSLIKLANEQGPIFQLHTPAGAIIVVSGHELV

KEVCDEERFDKSIEGALEKVRAFAGDGLFTSWTHEPNWRKAHNILMPTFSQRAMKDYHSM

MTDIAVQLIQKWARLNPDEAVDVPADMTRLTLDTIGLCGFNYRFNSYYRETPHPFINSMV

RALDEAMHQMQRLDVQDKLMIRTKRQFHHDIQAMFSLVDSIIAERRSGGRDEKDLLARML

NVEDPETGEKLDDENIRFQIITFLIAGHETTSGLLSFAIYFLLKHPRVLEKAYEEADRVL

TDPVPSYKQVLELSYIRMILQESLRLWPTAPAFSLYAKEDTVIGGKYPITPKDRISVLIP

QLHREKDAWGDNAEEFYPERFEHPDQVPHHAYKPFGNGQRACIGMQFALHEATLVLGMIL

QHFTFIDHTDYELDIKQTLTIKPGDFHIRVRPRNKEAVAAALPAAEKAAEDVEKEKRETK

GASIIGLDNRPLLILYGSDTGTAEGVARELADTAGMHGVRTETAPLNDRIGKLPKEGALL

IITSSYNGKPPSNAGQFVQWLEEVKPGELEGVRYAVFGCGDHNWAATYQAVPRLIDEKLA

EKGAERFSSRGEGDVSGDFEGKLDEWKKSMWTDAMKAFGLKLNENAEKERSALGLQFVSG

LGGSPLAQTYEAVYASVAENRELQAPESGRSTRHIEITLPKEAAYHEGDHLGVLPVNSKE

QVSRVLRRFHLNGNDQVLLTASGQSAAHLPLDRPVRLHDLLSSCVELQEAASRAQIREMA

AYTVCPPHKRELEDFLEEGVYQEQILTSRVSMLDLLEKYEACELPFERFLELLRPLKPRY

YSISSSPRKHPGQASITVGVVRGPARSGLGEYRGVSSNYLADRRPEDGIVMFVRTPETRF

RLPEDPEKPIIMVGPGTGVAPFRGFLQARAALKKEGKELGEAHLYFGCRNDHDFIYRDEL

EAYEKDGIVTLHTAFSRKEGVPKTYVQHLMAKDAGALISILGRGGRLYVCGDGSKMAPDV

EATLQKAYQSVRETDERQAQEWLLDLQTKGIYAKDVWAGI

>CYP102A49 *Bacillus amyloliquefaciens* IT-45

MKQLSAIPQPKTYGPLKNLPHLEKEKLSQSLWKIAEEYGPIFRFEFPSSVGVFVSGRELA

AEVCDEKRFDKNLSKALLKVREFGGDGLFTSWTHEKNWQKAHRILLPSFSQKAMKGYHSM

MLDIAMQLVQKWSRLNPNEEIDVAEDMTRLTLDTIGLCGFHYRFNSFYRDTQHPFITSML

RALQEAMRQSQRHSLQDKLMIKTRHQFQQDIEVMNELVDRIIAERRENPDENLSDLLALM

LEAKDPVTGERLDDENIRYQIITFLIAGHETTSGLLSFAIYCLLKNKDKLKKAVQEAERV

LTGETPEYKQIQQLIYIRMVLNETLRLYPTAPAFSLYAKEDTVLGGKYPIAKGQPVTILT

PQLHRDKSAWGEDAELFRPERFSDPAAIPADAYKPFGNGQRACIGMQFALQEATMVLGLV

LKHFELIDHTDYELTIKEALTIKPGDFKIRVKNKDVSNHQPVQNHKAEGGGHKEETKEIP

SHGTPLLILYGSNLGTAEGIAEELADIGRSKGFSTETGPLDDYAGKLPVKGAVVIVTASY

NGAPPDNAAGFVKWMETLEDQELKGVSYAVFGCGDRNWAATYQRIPRLIDKVLEEKGAKR

LTSIGEGDNADDFEYSQEAWEDSFWKDIMKAFHIEAAPKQANKSQLSIEYVSEAAETPIA

KTYKAFEAEVITNKELHTESSKRSVRHIELRLPKTETYQEGDHLGVLPQNSGELISRVIR

RFGLDPNQHFKIKGRQLPHLPMDRPVNAPELLASYVELQEPATRAQLRELAAHTVCPPHQ

KELEHLYSDDAAYKENVLKKRMTMLDLLEDYPACELPFERFLELLPSLKARYYSISSSPK

ANKRELSITVGVVTAPAWSGRGEYRGVASNYLAGLQNGDSAVCFIRSPQSGFALPENTKT

PLIMIGAGTGIAPFRGFIQARAAEKMSGNSLGEAHLYFGCRHPEEDDLYKDEFDHAEKNG

LVTVHRAYSRLDQDCKVYVQDVLLREAAQIIALLDQGGHLYICGDGSKMAPAVENVLLQA

YEKVHNTDSKVSSNWLEQLQAEGRYAKDVWAGV

>CYP107H4 *Bacillus amyloliquefaciens* IT-45

MTAGLSTAHPSLHADSDFWNDPYPFYEKLRAIDPVYKGTVLKHPGWYVTGYKEAAAILKD

TRFKNRVPFPEASTKYQNLSHIQHDMLLFKNQSDHKRMRMLIGKEFTAKTAESLRPCIKE

TVHDLLDQIQNKKNADLVSEFAFPLASLIIAEILGVPKDERYQFRQWTADVIQAIDLTRS

RKMLVRASDTAGRLTAYFRDLIHERKAHPQEDLISRFIMGEQLSKDEVLATCILLVIAGH

ETTVNLMSNGVFTLLKHPEQLSALRENPSLIETAVEECLRYDSPAQLTARTASEDCEING

KTIKKGEQVYILLGAANRDPSIFDQPHKMDIQRKPNPHLAFGKNAHFCIGSSLARIEAQI

AILTLFERMPKLRLAAHRLEYRKLIGFRSLKELPVVLG

>CYP107K3 *Bacillus amyloliquefaciens* IT-45

MEKTMFHPHSPEFHEDPFAVLSRFREQDPIHKFELQRFGGTFPAWLITRYDDCMAFLKDG

RITRDVKRVMPKELIAKLNVSEDIDFVSEHMLAKDPPDHSRLRSLVHQGFTPRMIEQLRT

GIEQITEELLNEMETKADPDIMRDFAAPLPFIVISELLGIPKEDRAKFQVWTNAMVDTSE

SGQDATNQALKEFKQYMKTLIEEKRKHPAEDLTSKLIYAEEDGQKLSESELYSMLFLLVV

AGLETTVNLLGSGTLALLLHKDQMEKIKRQPENIQTAVEELLRYTSPVIMMANRWAIEDF

TYKDVSIKKGDMIFIGIGSANRDPEYFDDPDTLNIARTPNRHISFGFGIHFCLGAPLARM

EASIAFTSLLKRFPNIELKGAHEDVTWRKNVFLRGLETLPVRF

>CYP109B7 *Bacillus amyloliquefaciens* IT-45

MSKLRSTRQAIQRTLMKGKNGLDVYNPFPWYEKMRRESPIYFDEETKVWSVFLYDDVKKV

ISDKETFSSRMTDVKSSIAKSMLNMDPPKHTQIRSAVNRAFTPRVLKEWEPRIKDITDHL

LKQAKNKGRIDIVKDLSYPLPVMVISELLGVPSERMDQFKKWSDILVSMPKDASPEAAEK

NQQERDQCEAELAAFFAGIIESKRKQPGQDIISILIKEEEEGEKLTPEDLIPFCNLLLVA

GNETTTNLISNAVYSILETPGLYEELRQDPSLIAQTVEETLRFRAPAPFVRRTVRHDTEL

RGRRLKSGEIVLCYVASANRDENKFEKAGVFDIHRQSNSHLSFGFGVHFCLGAPLARLEA

EAALKGIVKAFSHLEPVRIEPIRNSVMYGLESLEAEINENEGEEKR

>CYP113L1 *Bacillus amyloliquefaciens* IT-45

MTSLTKIRQQQPYKWYQTMRETSPVHYNEKEDCWEIFTYDEVKRVISDYSHFSSDHKYLS

ADKQEKMIRHINKDSLLKMDPPEHTVFRKLVNQPFMPKSVESLAPRIAAIADDLLQAVRS

KGRMDIIEDYAFPLPIIVIAELLGFPPKDRDIFKSWVDQSQNVKDEKKMNEVQKQMIGYF

MQFILQRRKQPQNDLISHLISADLDGEPLSDKQLIGFCGLLIVAGHVTTENVIGNSFLSL

KEFPHILPRLLENKALLPDFIEEVIRLRPSIQRVTRYTAVESEIGGKTIPAGEKVYAWIG

SANRDEKKFENADQIDLGRKPNQHLSFGQGSHYCLGAPLARLEAKIALSHFFEQMPAWRF

TEDQEPNLVPSPVFHGVDRLLVEF

>CYP102A38 *Bacillus amyloliquefaciens* LFB112

MKETGPIPQPKTFGPLGNLPLLDKDKPTMSLIKLANEQGPIFQLHTPAGAIIVVSGHELV

KEVCDEERFDKSIEGALEKVRAFAGDGLFTSWTHEPNWRKAHNILMPTFSQRAMKDYHSM

MTDIAVQLIQKWARLNPDEAVDVPADMTRLTLDTIGLCGFNYRFNSYYRETPHPFINSMV

RALDEAMHQMQRLDVQDKLMIRTKRQFHHDIQAMFSLVDSIIAERRSGGRDEKDLLARML

NVEDPETGEKLDDENIRFQIITFLIAGHETTSGLLSFAIYFLLKHPRVLEKAYEEADRVL

TDPVPSYKQVLELSYIRMILQESLRLWPTAPAFSLYAKEDTVIGGKYPITPKDRISVLIP

QLHRDKDAWGDNAEEFYPERFEHPDQVPHHAYKPFGNGQRACIGMQFALHEATLVLGMIL

QHFTFIDHTDYELDIKQTLTIKPGDFHIRVRPRNKGAVAAALPAAEKAAEDVEKEKRETK

GASIIGLDNRPLLILYGSDTGTAEGVARELADTAGMHGVRTETAPLNDRIGKLPKEGALL

IITSSYNGKPPSNAGQFVQWLEEVKPGELEGVRYAVFGCGDHNWAATYQAVPRLIDEKLA

EKGAERFSARGEGDVSGDFEGKLDEWKKSMWTDAMKAFGLKLNENAEKERSALGLQFVSG

LGGSPLAQTYEAVYASVAENRELQAPESGRSTRHIEITLPKEAAYHEGDHLGVLPVNSKE

QVSRVLRRFNLNGNDQVLLTASGQSAAHLPLDRPVRLHDLLSSCVELQEAASRAQIREMA

AYTVCPPHKRELEDFLEEGVYQEQILTSRVSMLDLLEKYEACELPFERFLELLRPLKPRY

YSISSSPRKHPGQASITVGVVRGPARSGLGEYRGVASNYLADRRPEDGIVMFVRTPETRF

RLPEDPEKPIIMVGPGTGVAPFRGFLQARAALKKEGKELGEAHLYFGCRNDHDFIYRDEL

EAYEKDGIVTLHTAFSRKEGVPKTYVQHLMAKDAGALISILGRGGHLYVCGDGSKMAPDV

EATLQKAYQSVRETDERQAQEWLLDLQTKGIYAKDVWAGI

>CYP102A49 *Bacillus amyloliquefaciens* LFB112

MKQLSAIPQPKTYGPLKNLPHLEKEKLSQSLWKIAEEYGPIFRFEFPSSVGVFVSGRELA

AEVCDEKRFDKNLSKALLKVREFGGDGLFTSWTHEKNWQKAHRILLPSFSQKAMKGYHSM

MLDIAMQLVQKWSRLNPNEEIDVAEDMTRLTLDTIGLCGFHYRFNSFYRDTQHPFITSML

RALQEAMRQSQRHSLQDKLMIKTRHQFQQDIEVMNELVDRIIAERRENPDENLSDLLALM

LEAKDPVTGERLDDENIRYQIITFLIAGHETTSGLLSFAIYCLLKNKDKLKKAVQEAERV

LTGETPEYKQIQQLTYIRMVLNETLRLYPTAPAFSLYAKEDTVLGGKYPIAKGQPVTILT

PQLHRDKSAWGEDAELFRPERFSDPAAIPADAYKPFGNGQRACIGMQFALQEATMVLGLV

LKHFELIDHTDYELTIKEALTIKPGDFKIRVKNKDVSNHQPVQNHKAEGGGHKEETKEIP

SHGTPLLILYGSNLGTAEGIAEELADIGRSKGFSTETGPLDDYAGKLPVKGAVVIVTASY

NGAPPDNAVGFVKWTETLEDQELKGVSYAVFGCGDRNWAATYQRIPRLIDKVLEEKGAKR

LTSIGEGDNADDFEYSQEAWEDSFWKDIMKAFHIEAAPKQANKSQLSIEYVSEAAETPIA

KTYKAFEAEVITNKELHTESSKRSVRHIELRLPKTETYREGDHLGVLPQNSGELISRVIR

RFGLDPNQHFKIKGRQLPHLPMDRPVNAPELLASYVELQEPATRAQLRELAAHTVCPPHQ

KELEHLYSDDAAYKENVLKKRMTMLDLLEDYPACELPFERFLELLPSLKARYYSISSSPK

ANKRELSITVGVVTAPAWSGRGEYKGVASNYLAGLQNGDSAVCFIRSPQSGFALPENTKT

PLIMIGAGTGIAPFRGFIQARAAEKMSGNSLGEAHLYFGCRHPEEDDLYKDEFDHAEKNG

LVTVHRAYSRLDQDCKVYVQDVLLREAAQIIALLDQGGHLYICGDGSKMAPAVENVLLQA

YEKVHNTDSKVSSNWLEQLQAEGRYAKDVWAGV

>CYP107H4 *Bacillus amyloliquefaciens* LFB112

MTAGLSTAHPSLHADSDFWNDPYPFYEKLRAIDPVYKGTVLKHPGWYVTGYKEAAAILKD

TRFKNRVPFPEASTKYQNLSHIQHDMLLFKNQSDHKRMRMLIGKEFTAKTAESLRPCIKE

TVHDLLDQIQNKKNADLVSEFAFPLASLIIAEILGVPKEERYQFRQWTADVIQAIDLTRS

RKMLVRASDTAGRLTAYFRDLIHERKAHPQEDLISRFIMGEQLSKDEVLATCILLVIAGH

ETTVNLMSNGVFTLLKHPEQLSALRENPSLIETAVEECLRYDSPAQLTARTASEDCEING

KTIKKGEQVYILLGAANRDPSIFDQPHKMDIQRKPNPHLAFGKNAHFCIGSSLARIEAQI

AILTLFERMPKLRLAAHRLEYRKLIGFRSLKELPVVLG

>CYP107K3 *Bacillus amyloliquefaciens* LFB112

MEKTMFHPHSPEFHEDPFAVLSRFREQDPIHKFELQRFGGTFPAWLITRYDDCMAFLKDG

RITRDVKRVMPKELIAKLNVSEDIDFVSEHMLAKDPPDHSRLRSLVHQGFTPRMIEQLRT

GIEQITEELLNEMETKADPDIMRDFAAPLPFIVISELLGIPKEDRAKFQVWTNAMVDTSE

SGQDATNQALKEFKQYMKTLIEEKRKHPAEDLTSKLIYAEEDGQKLSESELYSMLFLLVV

AGLETTVNLLGSGTLALLLHKDQMEKIKRQPENIQTAVEELLRYTSPVIMMANRWAIEDF

TYKDVSIKKGDMIFIGIGSANRDPEYFDDPDTLNIARTPNRHISFGFGIHFCLGAPLARM

EASIAFTSLLKRFPNIELKGAPEDVTWRKNVFLRGLETLPVRF

>CYP109B7 *Bacillus amyloliquefaciens* LFB112

MSKLRSTRQAIQRTLMKGKNGLDVYNPFPWYEKMRRESPIYFDEETKVWSVFLYDDVKKV

ISDKETFSSRMTDVKSSIAKSMLNMDPPKHTQIRSAVNRAFTPRVLKEWEPRIKDITDHL

LKQAKNKGRIDIVKDLSYPLPVMVISELLGVPSERMDQFKKWSDILVSMPKDASPEAAEK

NQQERDQCEAELAAFFAGIIESKRKQPGQDIISILIKEEEEGEKLTPEDLIPFCNLLLVA

GNETTTNLISNAVYSILETPGLYEELRQDPSLIAQTVEETLRFRAPAPFVRRTVRHDTEL

RGRRLKSGEIVLCYVASANRDENKFEKAGVFDIHRQSNPHLSFGFGVHFCLGAPLARLEA

EAALKGIVKAFSHLEPVRIEPIRNSVMYGLESLEAEINENEGEEKR

>CYP113L1 *Bacillus amyloliquefaciens* LFB112

MTSLTKIRQQQPYKWYQTMRETSPVHYNEKEDCWEIFTYDEVKRVISDYSHFSSDHKYLS

ADKQEKMIRHINKDSLLKMDPPEHTVFRKLVNQPFMPKSVESLAPRIAAIADDLLQAVRS

KGRMDIIEDYAFPLPIIVIAELLGFPPKDRDIFKSWVDQSQNVKDEKKMNEVQKQMIGYF

MQFILQRRKQPQNDLISHLISADLDGEPLSDKQLIGFCGLLIVAGHVTTENVIGNSFLSL

KEFPHILPRLLENKALLPDFIEEVIRLRPSIQRVTRYTAVESEIGGKTIPAGEKVYAWIG

SANRDEKKFENPDEIDLGRKPNQHLSFGQGSHYCLGAPLARLEAKIALSHFFEQMPAWRF

TEDQEPNLVPSPVFHGVDRLLVEF

>CYP102A35 *Bacillus atrophaeus* 1942

MKKTSPIPQPKTFGPLGNLPLIDKDRPTLSLSKLADEYGPIFQLNTPAGTTIIVSGHELV

EEICDESRFDKSIEGALEKVRAFSGDGLFTSWTHEPNWRKAHNILMPTFSQRAMKEYHSM

MVDIAVQLIQKWARLNPNEAVDVPGDMTRLTLDTIGLCGFNYRFNSYYRETPHPFINSMV

RALDEAMHQMQRLDFQDKLMVRTKRQFHHDIQTMFSLVDSIIAERKADGDQDEKDLLARM

LNVEDPETGEKLDDENIRFQIITFLIAGHETTSGLLSFAIYFLLKNPDKMKKAYEEVDQV

LTGPTPTYKQVLQLSYIRMILNESLRLWPTAPAFSLYAKEDTVIGGKYPITPKDRISVLI

PQLHRDKEAWGENAEEFHPERFENPDQVPHHAYKPFGNGQRACIGMQFALHEATLVLGMI

LQYFKLIDHTNYELDIKQTLTLKPGDFKIRVQSRNQEAMNSAVLTSDEKAPDDQKEKPDT

KSASIVGVNNRPLLVLYGSDTGTAEGVARELADTASMHGVRTEVAALNDQIGKLPKEGAV

LIVTSSYNGKPPSNAGQFVQWLEEIKPGELTGVQYAVFGCGDHNWASTYQDVPRYIDEQL

AQKGAARFSARGEGDVSGDFEGQLDQWKQTMWSDAMNAFGLKLNENAEKERSTLSLEFVR

GLGGSPLARSYEAVHASVTENRELQSADSDRSTRHIEIALPPGVTYQEGDHLGVLPSNSQ

EKVNRILRRFGLKGNDQVTLTASGRSAAHLPLDRPVSLHDLLSYSVDVQEAATRAQIREL

AAFTVCPPHKRELEGLAEEGVYQEKILQKRISMLDLLEEYEACEMPFERFLELLRPLKPR

YYSISSSPRVNPEQAAITVGVVRSPAWSGHGEYRGVASNYLADRTPGDDIDMFVRTPESR

FQLPEDPEKPIIMVGPGTGVAPFRGFLQARAALKQEGKTLGEAHLYFGCRNDNDFIYRGE

LEAYEKEGIVTLHTAFSRKEGIPKTYVQHLMAENAEELISILDQGGHLYVCGDGSKMAPD

VEATLQKAYQSVHGVGEQEAQKWLGNLQTNGMYAKDVWAGI

>CYP102A47 *Bacillus atrophaeus* 1942

MKQASLIPQPKTYGPLKNLPQLDKEKVSQSLWRIADEYGPIFRFEFPGAVGVFVSGHELV

AEVCDESRFDKNLSNSLQKVREFSGDGLFTSWTHEHNWQKAHRILLPSFSQKAMKGYHSM

MLDIAEQLIQKWSRLNPNEEIDVAEDMTRLTLDTIGLCGFNYRFNSFYRDTQHPFITSML

RALKEAMRQTQRLSLQDKLMVKAKQQFQHDIEVMNALVDRIIAERKENPDENVKDLLSLM

LHAEDPVTGERLDDENIRHQIITFLIAGHETTSGLLSFAIYCLLKNRDKLEKACQEAEQV

LTGDTPTYKQIQHLKYIRMVLNEALRLYPTAPAFSVYAKEDTVLGGQYQISKGQPVSVLV

PKLHRDQSVWGEDAEDFRPERFENPSDIPNHAYKPFGNGQRACIGMQFALQEATMVLGLV

LKHFELIDHTDYELKIKEALTIKPDHFKIRVKPKKSASKSFSKQTAEQPEQTNKRAETAG

KPSHGTPLLVLYGSNLGTAEGLAEELADIGRYQGFQTETAPLDDYIGNLPAKGAVVIVTA

SYNGAPPDNAAGFVKWIETLEAGELKGVNYAVFGCGNRNWAGTYQRIPRLIDETLAAKGA

NRLIPIGEGDAADDFESSQEEWEQRFWEDTLKAFHLQASSAKEERPALSIEFMSETIGTP

LAKTYDAFEAIVEENRKLQTNTSPRDTRHIELQVPVAEDYKEGDHIGILPKNSKELVGRV

IKRFGLAPHSLVKISGGRNVSHLPLEQPINVADLLSSNVELQEPATRAQLRELAAYTVCP

PHKKELEMLLSDQTYKDQVLKKRITMIDLLEDYPACEMPFERFLELLPSLKARYYSISSS

PRVYQHKVSITVGVVASPAWSGSGEYRGVASNYLAGLKAGDRVVCFIRTPQSGFRLPESF

ETPLIMVGPGTGIAPYRGFIQARGVWKEKGNKLGEAHLYFGCRHPEQDDLYREELDQAED

AGLVNVHRGYSRRETEPKVYVQHLLKQDAEQVIALLDQGAYFYVCGDGSRMAPEVEETLR

EAFEAVKGESRKASAEWISKLQEEGRYVKDVWTGV

>CYP107H6 *Bacillus atrophaeus* 1942

MTIKNLTIPVSLSSSEFFENPYPFYDKLRSIHPIYKGNFLKYPGWYVTGYAEAAAILKDT

RFKNRIPLPENSQKYSELKNMQHHMMLFKNPPDHKRIRMMVSRAFTPRFAESYRPYINET

VNVLLNEVQYKRTMDVVSDFAFPLASLIIAKMLGVPAGESYQFREWAASLIQTIDFTRSR

KELTKGNDKAIKLIAYFKDLIQQKKHNPQNDLISMLMKEQQDERLSDEELLATCILLVIA

GHETTVNLISNSILSLLNHPEKLMELKEKPSLIESAVEEFLRYESPTQMTARVASEDIEM

NGMMIKKEEHVYILLGAANRDPKKFTHPHVLDITRNPNPHLAFGQGIHFCLGSSLARLEA

QIAICTLLQRIPNLQLSTPNVQYRKLIGFRSLTELPVTF

>CYP107K2 *Bacillus atrophaeus* 1942

MEKLVFNAHSSEFHENPFTVLGHFREHDPIHHFELHRFGVTYRAWLITRYDDCMAFLKDN

RITRDVKNVMSKEQIKKLNVSDDIDFVSDHMLAKDTPDHTRLRSLVHQAFTPRIIENLRT

SIEQITERLLDDMEKENEPDIMKSFASPLPFIVISEMMGIPEEDRSQFQVWTNAMVDTSE

GNQEKTNQSLREFKDYIGKLIQERRIHPKDDMISKLVHAEEDGHKLSEKELYSMLFLLVV

AGLETTVNLLGSGTLALLQHREELEKLKQHPDMIDTAVEELLRYTAPVVMMANRWAIEDF

TYQGHSIKRGDMIFIGIGSANRDPERFENPEKFDISRSPNRHISFGFGIHFCLGAPLARL

EGQIAFNALLKRFPYIELAVASDEMKWRKNAFLRGLESLPVSLAKKNSLSRYS

>CYP109B2 *Bacillus atrophaeus* 1942

MNVVNQRQALQRAILKGINKQDAYHPFPWYESMRRESPVHYDEENQVWSVFLYEDVKKII

GDKNAFSNYVKQQANSLGNSIINMDPPRHTQIRSVVNKAFTPRVLKQWESRIQGITDDLI

DRLLGSQEFDLVQDFSYPLPVVVISELLGVPSEYMDQFKKWSDILVSTPKDGSEEAEKAF

QEERNKCEQELAAFFAAIIEEKRKQPAEDLISILIKAEEEGEKLSEDELIPFCNLLLVAG

NETTTNLISNAMYSILDMPGAYDELRDDPALIPQAVEEALRFRAPAPILRRIAKQDVEIR

GHLIREGDMVLAFVASANRDEAKFEQAHIFDIHRHPNPHIAFGHGNHFCLGAPLARLEAQ

IALKALTNAFPGMERISISPIANSVIYGLKSFRVKV

>CYP152A9 *Bacillus atrophaeus* 1942

MSMDKQVPHDKALDNSLAMMREGYLFIKNRVDRYQSDLFETRLLGKKAICMSGEEAAKIF

YDPERMKRNGALPKRLLKTLFGVDAIQTMDGDAHTHRKLLFMLLMTPPHQKRLAELAMDQ

WLAALSKWEGAEKVELFEEAKNVLCKIACQWAGVPLEESEVKERADDFSAMVDAFGAVGP

RHWKGRRARPRAEEWIRDIIENVRAGKTEAEKGTALYEMAFHTELDGSRLDTQMAAVELI

NVLRPIVAISTFITFSALALHNHPEYKEKLKSGNGDDLEMFVQEVRRFYPFGPFLGAQAR

KDFVWNQCEFKEGMLVLLDLYGTNHDARLWEAPNEFRPERFKDRKDQLFDLIPQGGGDPA

KGHRCPGEGITIEVMKVTLDFLINHIEYEVPEQDLSYSLVRMPSLPESGFVMSRIRRK

>CYP102A35 *Bacillus atrophaeus* NRS 1221A

MKKTSPIPQPKTFGPLGNLPLIDKDRPTLSLSKLADEYGPIFQLNTPAGTTIIVSGHELV

EEICDESRFDKSIEGALEKVRAFSGDGLFTSWTHEPNWRKAHNILMPTFSQRAMKEYHSM

MVDIAVQLIQKWARLNPNEAVDVPGDMTRLTLDTIGLCGFNYRFNSYYRETPHPFINSMV

RALDEAMHQMQRLDFQDKLMVRTKRQFHHDIQTMFSLVDSIIAERKADGDQDEKDLLARM

LNVEDPETGEKLDDENIRFQIITFLIAGHETTSGLLSFAIYFLLKNPDKMKKAYEEVDQV

LTGPTPTYKQVLQLSYIRMILNESLRLWPTAPAFSLYAKEDTVIGGKYPITPKDRISVLI

PQLHRDKEAWGENAEEFHPERFENPDQVPHHAYKPFGNGQRACIGMQFALHEATLVLGMI

LQYFKLIDHTNYELDIKQTLTLKPGDFKIRVQSRNQEAMNSAVLTSDEKAPDDQKEKPDT

KSASIVGVNNRPLLVLYGSDTGTAEGVARELADTASMHGVRTEVAALNDQIGKLPKEGAV

LIVTSSYNGKPPSNAGQFVQWLEEIKPGELTGVQYAVFGCGDHNWASTYQDVPRYIDEQL

AQKGAARFSARGEGDVSGDFEGQLDQWKQTMWSDAMNAFGLKLNENAEKERSTLSLEFVR

GLGGSPLARSYEAVHASVTENRELQSADSDRSTRHIEIALPPGVTYQEGDHLGVLPSNSQ

EKVNRILRRFGLKGNDQVTLTASGRSAAHLPLDRPVSLHDLLSYSVDVQEAATRAQIREL

AAFTVCPPHKRELEGLAEEGVYQEKILQKRISMLDLLEEYEACEMPFERFLELLRPLKPR

YYSISSSPRVNPEQAAITVGVVRSPAWSGHGEYRGVASNYLADRTPGDDIDMFVRTPESR

FQLPEDPEKPIIMVGPGTGVAPFRGFLQARAALKQEGKTLGEAHLYFGCRNDNDFIYRGE

LEAYEKEGIVTLHTAFSRKEGIPKTYVQHLMAENAEELISILDQGGHLYVCGDGSKMAPD

VEATLQKAYQSVHGVGEQEAQKWLGNLQTNGMYAKDVWAGI

>CYP102A47 *Bacillus atrophaeus* NRS 1221A

MKQASLIPQPKTYGPLKNLPQLDKEKVSQSLWRIADEYGPIFRFEFPGAVGVFVSGHELV

AEVCDESRFDKNLSNSLQKVREFSGDGLFTSWTHEHNWQKAHRILLPSFSQKAMKGYHSM

MLDIAEQLIQKWSRLNPNEEIDVAEDMTRLTLDTIGLCGFNYRFNSFYRDTQHPFITSML

RALKEAMRQTQRLSLQDKLMVKAKQQFQHDIEVMNALVDRIIAERKENPDENVKDLLSLM

LHAEDPVTGERLDDENIRHQIITFLIAGHETTSGLLSFAIYCLLKNRDKLEKACQEAEQV

LTGDTPTYKQIQHLKYIRMVLNEALRLYPTAPAFSVYAKEDTVLGGQYQISKGQPVSVLV

PKLHRDQSVWGEDAEDFRPERFENPSDIPNHAYKPFGNGQRACIGMQFALQEATMVLGLV

LKHFELIDHTDYELKIKEALTIKPDHFKIRVKPKKSASKSFSKQTAEQPEQTNKRAETAG

KPSHGTPLLVLYGSNLGTAEGLAEELADIGRYQGFQTETAPLDDYIGNLPAKGAVVIVTA

SYNGAPPDNAAGFVKWIETLEAGELKGVNYAVFGCGNRNWAGTYQRIPRLIDETLAAKGA

NRLIPIGEGDAADDFESSQEEWEQRFWEDTLKAFHLQASSAKEERPALSIEFMSETIGTP

LAKTYDAFEAIVEENRKLQTNTSPRDTRHIELQVPVAEDYKEGDHIGILPKNSKELVGRV

IKRFGLAPHSLVKISGGRNVSHLPLEQPINVADLLSSNVELQEPATRAQLRELAAYTVCP

PHKKELEMLLSDQTYKDQVLKKRITMIDLLEDYPACEMPFERFLELLPSLKARYYSISSS

PRVYQHKVSITVGVVASPAWSGSGEYRGVASNYLAGLKAGDRVVCFIRTPQSGFRLPESF

ETPLIMVGPGTGIAPYRGFIQARGVWKEKGNKLGEAHLYFGCRHPEQDDLYREELDQAED

AGLVNVHRGYSRRETEPKVYVQHLLKQDAEQVIALLDQGAYFYVCGDGSRMAPEVEETLR

EAFEAVKGESRKASAEWISKLQEEGRYVKDVWTGV

>CYP107H6 *Bacillus atrophaeus* NRS 1221A

MTIKNLTIPVSLSSSEFFENPYPFYDKLRSIHPIYKGNFLKYPGWYVTGYAEAAAILKDT

RFKNRIPLPENSQKYSELKNMQHHMMLFKNPPDHKRIRMMVSRAFTPRFAESYRPYINET

VNVLLNEVQYKRTMDVVSDFAFPLASLIIAKMLGVPAGESYQFREWAASLIQTIDFTRSR

KELTKGNDKAIKLIAYFKDLIQQKKHNPQNDLISMLMKEQQDERLSDEELLATCILLVIA

GHETTVNLISNSILSLLNHPEKLMELKEKPSLIESAVEEFLRYESPTQMTARVASEDIEM

NGMMIKKEEHVYILLGAANRDPKKFTHPHVLDITRNPNPHLAFGQGIHFCLGSSLARLEA

QIAICTLLQRIPNLQLSTPNVQYRKLIGFRSLTELPVTF

>CYP107K2 *Bacillus atrophaeus* NRS 1221A

MEKLVFNAHSSEFHENPFTVLGHFREHDPIHHFELHRFGVTYRAWLITRYDDCMAFLKDN

RITRDVKNVMSKEQIKKLNVSDDIDFVSDHMLAKDTPDHTRLRSLVHQAFTPRIIENLRT

SIEQITERLLDDMEKENEPDIMKSFASPLPFIVISEMMGIPEEDRSQFQVWTNAMVDTSE

GNQEKTNQSLREFKDYIGKLIQERRIHPKDDMISKLVHAEEDGHKLSEKELYSMLFLLVV

AGLETTVNLLGSGTLALLQHREELEKLKQHPDMIDTAVEELLRYTAPVVMMANRWAIEDF

TYQGHSIKRGDMIFIGIGSANRDPERFENPEKFDISRSPNRHISFGFGIHFCLGAPLARL

EGQIAFNALLKRFPYIELAVASDEMKWRKNAFLRGLESLPVSLAKKNSLSRYS

>CYP109B2 *Bacillus atrophaeus* NRS 1221A

MNVVNQRQALQRAILKGINKQDAYHPFPWYESMRRESPVHYDEENQVWSVFLYEDVKKII

GDKNAFSNYVKQQANSLGNSIINMDPPRHTQIRSVVNKAFTPRVLKQWESRIQGITDDLI

DRLLGSQEFDLVQDFSYPLPVVVISELLGVPSEYMDQFKKWSDILVSTPKDGSEEAEKAF

QEERNKCEQELAAFFAAIIEEKRKQPAEDLISILIKAEEEGEKLSEDELIPFCNLLLVAG

NETTTNLISNAMYSILDMPGAYDELRDDPALIPQAVEEALRFRAPAPILRRIAKQDVEIR

GHLIREGDMVLAFVASANRDEAKFEQAHIFDIHRHPNPHIAFGHGNHFCLGAPLARLEAQ

IALKALTNAFPGMERISISPIANSVIYGLKSFRVKV

>CYP152A9 *Bacillus atrophaeus* NRS 1221A

MDKQVPHDKALDNSLAMMREGYLFIKNRVDRYQSDLFETRLLGKKAICMSGEEAAKIFYD

PERMKRNGALPKRLLKTLFGVDAIQTMDGDAHTHRKLLFMLLMTPPHQKRLAELAMDQWL

AALSKWEGAEKVELFEEAKNVLCKIACQWAGVPLEESEVKERADDFSAMVDAFGAVGPRH

WKGRRARPRAEEWIRDIIENVRAGKTEAEKGTALYEMAFHTELDGSRLDTQMAAVELINV

LRPIVAISTFITFSALALHNHPEYKEKLKSGNGDDLEMFVQEVRRFYPFGPFLGAQARKD

FVWNQCEFKEGMLVLLDLYGTNHDARLWEAPNEFRPERFKDRKDQLFDLIPQGGGDPAKG

HRCPGEGITIEVMKVTLDFLINHIEYEVPEQDLSYSLVRMPSLPESGFVMSRIRRK

>CYP102A4 *Bacillus cereus* AH187

MDKKVSAIPQPKTYGPLGNLPLIDKDKPTLSFIKLAEEYGPIFQIQTLSDTIIVVSGHEL

VAEVCDETRFDKSIEGALAKVRAFAGDGLFTSETQEPNWQKAHNILMPTFSQRAMKDYHA

MMVDIAVQLVQKWARLNPNENVDVPEDMTRLTLDTIGLCGFNYRFNSFYRETPHPFITSM

TRALDEAMHQLQRLDIEDKLMWRTKRQFQHDIQSMFSLVDNIIAERKSSEKQEENDLLSR

MLNVQDPETGEKLDDENIRFQIITFLIAGHETTSGLLSFAIYFLLKNPDKLKKAYEEVDR

VLTDPTPTYQQVMKLKYIRMILNESLRLWPTAPAFSLYAKEDTVIGGKYPIKKGKDRISV

LIPQLHRDKDAWGDNVEEFQPERFEDLDKVPHHAYKPFGNGQRACIGMQFALHEATLVMG

MLLQHFEFIDYEDYQLDVKQTLTLKPGDFKIRIVPRNQTISHTTVLAPTEEKLKKHEIKQ

QVQKTPSIIGADNLSLLVLYGSDTGVAEGIARELADTASLEGVQTEVVALNDRIGSLPKE

GAVLIVTSSYNGKPPSNAGQFVQWLEELKPDELKGVQYAVFGCGDHNWASTYQRIPRYID

EQMAQKGATRFSTRGEADASGDFEEQLEQWKQRMWSDAMKAFGLELNKNMEKERSTLSLQ

FVSRLGGSPLARTYEAVYASILENRELQSSSSERSTRHIEISLPEGATYKEGDHLGVLPI

NSEKNVNRILKRFGLNGKDQVILSASGRSVNHIPLDSPVRLYDLLSYSVEVQEAATRAQI

REMVTFTACPPHKKELESLLEDGVYQEQILKKRISMLDLLEKYEACEIRFERFLELLPAL

KPRYYSISSSPLVAQDRLSITVGVVNAPAWSGEGTYEGVASNYLAQRHNKDEIICFIRTP

QSNFQLPENPETPIIMVGPGTGIAPFRGFLQARRVQKQKGMNVGEAHLYFGCRHPEKDYL

YRTELENDERDGLISLHTAFSRLEGQAKTYVQHVIKEDRIHLISLLDNGAHLYICGDGSK

MAPDVEDTLCQAYQEIHEVSEQEARNWLDRLQEEGRYGKDVWAGI

>CYP102A8 *Bacillus cereus* AH187

MDKKVSAIPQPKTYGPLGNLPLIDKDKPTLSFIKLAEEYGPIFQIQTLSDTIIVVSGHEL

VAEVCDETRFDKSIEGALAKVRAFAGDGLFTSETDEPNWKKAHNILMPTFSQRAMKDYHA

MMVDIAVQLVQKWARLNPNENVDVPGDMTRLTLDTIGLCGFNYRFNSFYRETSHPFITSM

TRALDEAMHQLQRLDIEDKLMWRTKRQFQHDIQSMFSLVDNIIAERKSSGNQEENDLLSR

MLHVQDPETGEKLDDENIRFQIITFLIAGHETTSGLLSFAIYFLLKNPDKLEKAYEEVDR

VLTDPTPTYQQVMKLKYIRMILNESLRLWPTAPAFSLYAKEDTVIGGKYPIKKGEDRISV

LIPQLHRDKDAWGDNVEEFQPERFEELDKVPHHAYKPFGNGQRACIGMQFALHEATLVMG

MLLQHFEFIDYEDYQLDVKQTLTLKPGDFKIRIVPRNETISHNTVLAPTEEKLKNDETKQ

QVQKTPSIIGADNLSLLVLYGSDTGVAEGIARELADTASLEGVQTEVAALNDRIGSLPKE

GAVLIVTSSYNGKPPSNAGQFVQWLEELKPDELKGVQYAVFGCGDHNWASTYQRIPRYID

EQMAQKGATRFSKRGEADASGDFEEQLEQWKQSMWSDAMKSFGLELNKNMEKERSTLSLQ

FVSRLGGSPLARTYEAVYASILENRELQSSSSERSTRHIEISLPEGATYKEGDHLGVLPI

NSEKNVNRILKRFGLNGKDQVILSASGRSVNHIPLDSPVRLYDLLSYSVEVQEAATRAQI

REMATFTVCPPHKKELESLLEEGVYQEQILKKRISMLDLLEKYEACEIRFERFLELLPAL

KPRYYSISSSPLVAQDRLSITVGVVNAPAWSGEGTYEGVASNYLAQRHNKDEIICFIRTP

QSNFQLPENLETPIIMVGPGTGIAPFRGFLQARRVQKQKGMKVGEAHLYFGCRHPEKDYL

YRTELENDEREGLISLHTAFSRLEGHPKTYVQHVIKEDRIHLISLLDNGAHLYICGDGSK

MAPDVEDTLCQAYQEIHEVSEQEARNWLDRLQDEGRYGKDVWAGI

>CYP106B1 *Bacillus cereus* AH187

MASPENVILVHEISKLKTKEELWNPYEWYQFMRDNHPVHYDDEQDVWNVFLYDDVNRVLS

DYSLFSSRRERRQFAIPPLETRININSTDPPEHRNVRSIVSKAFTPRSLEQWKPRIQSIA

NELVKDIENCSEVDIVEQFAAPLPVTVISDLLGVPTTDRKKIKAWSDILFMPYSKEKFND

LDAEKGIALNEFKAYLLPIVQEKRYHLTDDIISDLIRAEYEGERLTDEEIVTFSLGLLAA

GNETTTNLIINSFYCFLVDSPATYKEVREKPKLISKAVEEVLRYRFPVTLARRITEDTNI

FGPLMKKDQMVVAWVSAANLDEKKFSQASKFNIHRIGNEKHLTFGKGPHFCLGAPLARLE

AEIALTTFINAFEKIALSPSFNIEQCILENEQTLKFLPIRLKPQ

>CYP107J2 *Bacillus cereus* AH187

MAMKNKVGLRIEDGINLASAQFKEDAYEIYKESRKVQPVLFVNKTELGAEWLITRYEDAL

PLLKDNRLKKDPANVFSQDTLNVFLTVDNSDYLTTHMLNSDPPNHNRLRSLVQKVFTPKM

IAQLEGRIQDIADDLLNEVERKGSLNLVDDYSFPLPIIVISEMLGIPKEDQAKFRIWSHA

VIAYPETPEEIKETEKQLSEFITYLQYLVDMKRKEPKEDLVSALILAESEGHKLSARELY

SMIMLLIVAGHETTVNLITNTVLALLENPNQLQLLKENPKLIDAAIEEGLRYYSPVEVTT

SRWADEPFQIHDQTIEKGDMVVIALASANRDETVFENPEVFDITRENNRHIAFGHGSHFC

LGAPLARLEAKIAITTLFERMPELQIKGNREDIKWQGNYLMRSLEELPLTF

>CYP109T2 *Bacillus cereus* AH187

MKTPYQSVVMIPTNKLMGKKALEDPYKPFAWYKEMREKEPICFNHQADMWNVFLYEDVKT

VLEDKEYFSSIMPEKKKSPFPQSILGMDPPKHTQIRSIVNRSFTPKALREWEPRIQQITN

DILNQLSNRETFDIVRELFYPLPVIVIAEMLGVSAKDMERFKRWSDIIVSSPSHDDSDYL

EEFFNTRLQAENELGEFFEEIIQSNRGKSKKDSNNIISLLVQSEADKNISGKEIVPFCKL

LLVAGNETTTNLLGNALYCFIEHPNVYDQLQQDVSLVPKAIEEVLRYRSPVQRIVRRVKK

EIQLKGQTLQVDQIISAWVGSANRDSHQFKDVDSFNIYRRRNPHLTFGHGIHFCLGAPLA

RLEAKIVLTELIKRYKSFSFIDQDLPVPISNSSSIYGLHSFPVKSELN

>CYP102A8 *Bacillus cereus* ATCC 10987

MDKKVSAIPQPKTYGPLGNLPLIDKDKPTLSFIKIAEEYGPIFQIQTLSDTIIVISGHEL

VAEVCDETRFDKSIEGALAKVRAFAGDGLFTSETQEPNWKKAHNILMPTFSQRAMKDYHA

MMVDIAVQLVQKWARLNPNENVDVPEDMTRLTLDTIGLCGFNYRFNSFYRETPHPFITSM

TRALDEAMHQLQRLDIEDKLMWRTKRQFQHDIQSMFSLVDNIIAERKSSGNQEENDLLSR

MLHVQDPETGEKLDDENIRFQIITFLIAGHETTSGLLSFAIYFLLKNPDKLKKAYEEVDR

VLTDPTPTYQQVMKLKYIRMILNESLRLWPTAPAFSLYAKEDTVIGGKYPIKKGEDRISV

LIPQLHRDKDAWGDNVEEFQPERFEDLDKVPHHAYKPFGNGQRACIGMQFALHEATLVMG

MLLQHFEFIDYEDYQLDVKQTLTLKPGDFKIRIVPRNQNISHTTVLAPTEEKLKNHEIKQ

QVQKTPSIIGADNLSLLVLYGSDTGVAEGIARELADTASLEGVQTEVAALNDRIGSLPKE

GAVLIVTSSYNGKPPSNAGQFVQWLEELKPDELKGVQYAVFGCGDHNWASTYQRIPRYID

EQMAQKGATRFSTRGEADASGDFEEQLEQWKESMWSDAMKAFGLELNKNMEKERSTLSLQ

FVSRLGGSPLARTYEAVYASILENRELQSSSSERSTRHIEISLPEGATYKEGDHLGVLPI

NSEKNVNRILKRFGLNGKDQVILSASGRSVNHIPLDSPVRLYDLLSYSVEVQEAATRAQI

REMVTFTACPPHKKELESLLEDGVYHEQILKKRISMLDLLEKYEACEIRFERFLELLPAL

KPRYYSISSSPLIAQDRLSITVGVVNAPAWSGEGTYEGVASNYLAQRHNKDEIICFIRTP

QSNFQLPENPETPIIMVGPGTGIAPFRGFLQARRVQKQKGMNLGEAHLYFGCRHPEKDYL

YRTELENDERDGLISLHTAFSRLEGHPKTYVQHVIKEDRMNLISLLDNGAHLYICGDGSK

MAPDVEDTLCQAYQEIHEVSEQEARNWLDRLQDEGRYGKDVWAGI

>CYP106B1 *Bacillus cereus* ATCC 10987

MASPENVILVHEISKLKTKEELWNPYEWYQFMRDNHPVHYDEEQDVWNVFLYEDVNRVLS

DYRLFSSRRERRQFSIPPLETRININSTDPPEHRNVRSIVSKAFTPRSLEQWKPRIQAIA

NELVQHIGKYSEVNIVEEFAAPLPVTVISDLLGVPTTDRKKIKAWSDILFMPYSKEKFND

LDVEKGIALNEFKAYLLPIVQEKRYHLTDDIISDLIRAEYEGERLTDEEIVTFSLGLLAA

GNETTTNLIINSFYCFLVDSPGTYKELREEPTLISKAIEEVLRYRFPITLARRITEDTNI

FGPLMKKDQMVVAWVSAANLDEKKFSQASKFNIHRIGNEKHLTFGKGPHFCLGAPLARLE

AEIALTTFINAFEKIALSPSFNLEQCILENEQTLKFLPICLKTQ

>CYP107J3 *Bacillus cereus* ATCC 10987

MSMKNKVGLSIEDGINLASAQFKEDAYEIYKESRKKQPILFVNQVEIGKEWLITRYEDAL

PLLKDNRLKKDWTNVFSQDTKNMYLSVDNSDHLTTHMLNSDPPNHSRLRSLVQKAFTPKM

IAQLDGRIQRIADDLISDIERKGTLNLVDDYSFPLPIIVISEMLGIPKEDQAKFRIWSHA

VIASPETPEEIKETEKQLSEFITYLQYLVDVKRKEPKEDLVSALILAESEGHKLSARELY

SMIMLLIVAGHETTVNLITNTVLALLENPNQLQLLKDNPKLIDSAIEEGLRYYSPVEVTT

ARWAAEPFQIHHQTIQKGDMVIIALASANRDETVFENPEIFDITRENNRHIAFGHGSHFC

LGAPLARLEAKIAITTLFKRMPELQIKGDRKDIKWQGNYLMRSLEELPLTF

>CYP107DY3 *Bacillus cereus* ATCC 10987

MKKLTFNDLNSPETMRNPIMFYKNLMEQKERFFHIDDFYGMGGAWVVFHYDDVVAILKDS

RFIKDLRKFTPPHYKQNPIEENTAVSKLFEWLMNMPNMLTVDPPDHTRLRRLVSKSFTPR

MIEDLRPRIQQIADELLDVVQEQRKMEIIADFAYPLPIIVISEMLGIPATDRNQFRAWTQ

ELMKASVDPGQGTTVTATLEKFINYIEILFNEKHLNPSDDLISALVQAKEQEDKLSKNEL

LSTIWLLIIAGHETTVNLISNGVLALLQHPEQMNLLRQDPSLLASAVDELLRYAGPIMFS

SRFASEDVTIHGNRIRKGELVLLSLTAANIDPNIFPYPEELNISREENNHLAFGAGIHQC

LGAPLARLEGQIALDTLLKRLPNLRLAIEADQLIYNHSKIRSLASLPVIF

>CYP109T4 *Bacillus cereus* ATCC 10987

MKTPYQSIDMIPTSKLIGKKALEDPYKPFAWYKEMREKEPICFNHQADMWNVFLYEDVKT

VLEDKEHFSNIMPEKKKSPFPQSILGMDPPKHTQIRSIVNRSFTPKSLREWEPRIQQITN

DILNQLSNRKTFDIVRELFYPLPVIVIAEMLGVSAKDMERFKKWSDIIVSSPSHDDPDYL

AEFFHTRLQAENELGDFFEEIIQLNREKSQKDANDIISLLVQSEAEKSISGKELVSFCKL

LLVAGNETTTNLLGNALYCFIEYPNVYEQLQQDISLIPKAIEEVLRYRSPVQRITRRVKK

EIQLKYQTLQVDQIISAWVGSANRDSHQFTDGDSFNIYRGRNPHLTFGHGIHFCLGAPLA

RLEAKIVLTELIKRYKSFSFIDENLPTPISNSSTIYGLSSFPVKSELLHIK

>CYP109T *Bacillus cereus* ATCC 10987

MLGVSINDMDRFKEWSDIIISSPSHNNPDYLTEFFQRRLQAENELEGLFEEMLKIKREKS

KDNSDDIISLLVQSEADNKISGEEIVSFYKLLLVAGNETTTNLLGNLLYCLIEYPDVYKQ

IQQDISLIPKVIEEVLRYRSPAQRIVRRVKKGMQLNGQTLKVGQTVAAWIGSANRDSHYF

NDADSFNIHRPRNPHLGFGYGINFCLGARLARLEATIVLTEIIKKYKSFSFIDQNLPIPI

SNSSSVYGLKSFPVKSEIIHIK

>CYP102A36 *Bacillus gibsonii*

MKETSPIPQPKTFGPLGNLPLIDKDKPTLSLIKLAEEQGPIFQIHTPAGTTIVVSGHELV

KEVCDEERFDKSIEGALEKVRAFSGDGLFTSWTHEPNWRKAHNILMPTFSQRAMKDYHEK

MVDIAVQLIQKWARLNPNEAVDVPGDMTRLTLDTIGLCGFNYRFNSYYRETPHPFINSMV

RALDEAMHQMQRLDVQDKLMVRTKRQFHHDIQTMFSLVDSIIAERRSNGDQDEKDLLARM

LNVEDPETGEKLDDENIRFQIITFLIAGHETTSGLLSFATYFLLKHPDKLKKAYEEVDRV

LTDAAPTYKQVLELTYIRMILNESLRLWPTAPAFSLYPKEDTVIGGKFPITTNDRISVLI

PQLHRDRDAWGKDAEEFRPERFEHQDQVPHHAYKPFGNGQRACIGMQFALHEATLVLGMI

LKYFTLIDHENYELDIKQTLTLKPGDFHIRVQSRNQDAIHADVQAAEKAASDEQKEKTEA

KGASVIGLNNRPLLVLYGSDTGTAEGVARELADTASLHGVRTETAPLNDRIGKLPKEGAV

VIVTSSYNGKPPSNAGQFVQWLQEIKPGELEGVHYAVFGCGDHNWASTYQYVPRFIDEQL

AEKGATRFSARGEGDVSGDFEGQLDEWKKSMWADAIKAFGLELNENADKERSTLSLQFVR

GLGESPLARSYEASHASIAENRELQSADSDRSTRHIEIALPPDVEYQEGDHLGVLPKNSQ

TNVSRILHRFGLKETDQVTLSASGRSAGHLPLGRPVSLHDLLSYSVEVQEAATRAQIREL

AAFTVCPPHRRELEELSAEGVYQEQILKKRISMLDLLEKYEACDMPFERFLELLRPLKPR

YYSISSSPRVNPRLASITVGVVRGPAWSGRGEYRGVASNDLAERQAGDDLVMFVRTPESR

FQLPEDPETPIIMVGPGTGVAPFRGFLQAREVLKREGKTLGEAHLYFGCRNDRDFIYRDE

LEQFEKDGIVTVHTAFSRKEGMPKTYVQHVMADHAETLISILDRGGRLYVCGDGSKMAPD

VEAALQKAYQSVHGTGEQEAQNWLRHLQDTGMYAKDVWAGI

>CYP102A48 *Bacillus gibsonii*

MKQASAIPQPKTYGPLKNLPHLEKEQLSQSLWRIADELGPIFRFDFPGVSSVFVSGHNLV

AEVCDEKRFDKNLGKGLQKVREFGGDGLFTSWTHEPNWQKAHRILLPSFSQKAMKGYHSM

MLDIATQLIQKWSRLNPNEEIDVADDMTRLTLDTIGLCGFNYRFNSFYRDSQHPFITSML

RALKEAMNQSKRLGLQDKMMVKTKLQFQKDIEVMNSLVDRMIAERKANPDENIKDLLSLM

LYAKDPVTGETLDDENIRYQIITFLIAGHETTSGLLSFAIYCLLTHPEKLKKAQEEADRV

LTDDTPEYKQIQQLKYIRMVLNETLRLYPTAPAFSLYAKEDTVLGGEYPISKGQPVTVLI

PKLHRDQNAWGPDAEDFRPERFEDPSSIPHHAYKPFGNGQRACIGMQFALQEATMVLGLV

LKHFELINHTGYELKIKEALTIKPDDFKITVKPRKTAAINVQRKEQADIKAETKPKETKP

KHGTPLLVLFGSNLGTAEGIAGELAAQGRQMGFTAETAPLDDYIGKLPEEGAVVIVTASY

NGAPPDNAAGFVEWLKELEEGQLKGVSYAVFGCGNRSWASTYQRIPRLIDDMMKAKGASR

LTAIGEGDAADDFESHRESWENRFWKETMDAFDINEIAQKEDRPSLSITFLSEATETPVA

KAYGAFEGIVLENRELQTAASTRSTRHIELEIPAGKTYKEGDHIGILPKNSRELVQRVLS

RFGLQSNHVIKVSGSAHMAHLPMDRPIKVADLLSSYVELQEPASRLQLRELASYTVCPPH

QKELEQLVSDDGIYKEQVLAKRLTMLDFLEDYPACEMPFERFLALLPSLKPRYYSISSSP

KVHANIVSMTVGVVKASAWSGRGEYRGVASNYLAELNTGDAAACFIRTPQSGFQMPDEPE

TPMIMVGPGTGIAPFRGFIQARSVLKKEGSTLGEALLYFGCRRPDHDDLYREELDQAEQD

GLVTIRRCYSRVENEPKEYVQHLLKQDTQKLMTLIEKGAYIYVCGDGSQMAPDVEKTLRL

AYEAEKGASQEESAEWLQKLQDQKRYVKDVWTGM

>CYP107J1 *Bacillus gibsonii*

MSSKEKKSVTILTESQLSSRAFKDEAYEFYKELRKSQPLYPLSLGSLGKGWLISRYDDAI

HLLKNEKLKKNYENVFTAKEKRPALLKNEESLTKHMLNSDPPDHNRLRTLVQKAFTHRMI

LQLEDKIQHIADSLLDKVQPNKFMNLVDDYAFPLPIIVISEMLGIPLEDRQKFRVWSQAI

IDFSDAPERLQENDHLLGEFVEYLESLVRKKRREPAGDLISALIQAESEGTQLSTEELYS

MIMLLIVAGHETTVNLITNMTYALMCHGDQFEKLRQQPDLMNSAIEEALRFHSPVELTTI

RWTAEPFILHGQEIKRKDVIIISLASANRDEKIFPNADIFDIERKNNRHIAFGHGNHFCL

GAQLARLEAKIAMSTLLRRCPNIQLKGEKEQMKWKGNFLMRALEELPLSF

>CYP107K1 *Bacillus gibsonii*

MEKLMFHPHGKEFHHNPFSVLGRFREEEPIHRFELKRFGATYPAWLITRYDDCMAFLKDN

RITRDVKNVMNQEQIKMLNVSEDIDFVSDHMLAKDTPDHTRLRSLVHQAFTPRTIENLRG

SIEQIAEQLLDEMEKENKADIMKSFASPLPFIVISELMGIPKEDRSQFQIWTNAMVDTSE

GNRELTNQALREFKDYIAKLIHDRRIKPKDDLISKLVHAEENGSKLSEKELYSMLFLLVV

AGLETTVNLLGSGTLALLQHKKECEKLKQYPEMIATAVEELLRYTSPVVMMANRWAIEDF

TYKGHSIKRGDMIFIGIVSANRDPNFFENPEILNINRSPNRHISFGFGIHFCLGAPLARL

EGHIAFNALLKRFPDIQLAVAPDDIQWRKNVFLRGLESLPVSLSK

>CYP134A1 *Bacillus gibsonii*

MSQSIKLFSVLSDQFQNNPYAYFSQLREEDPVHYEESIDSYFISRYHDVRYILQHPDIFT

TKSLVERAEPVMRGPVLAQMHGKEHSAKRRIVVRSFIGDALDHLSPLIKQNAENLLAPYL

ERGKIDLVNDFGKTFAVCVTMDMLGLDKRDHEKISEWHSGVADFITSISQSPEARAHSLW

CSEQLSQYLMPVIKERRVNPGSDLISILCTSEYEGMALSDKDILALILNVLLAATEPADK

TLALMIYHLLNNPEQMNDVLADRSLVPRAIAETLRYKPPVQLIPRQLSQDTVVGGMEIKK

DTIVFCMIGAANRDPEAFEQPDVFNIHREDLGIKSAFSGAARHLAFGSGIHNCVGAAFAK

NEIEIVANIVLDKMRNIRLEEDFCYAESGLYTRGPVSLLVAFDGA

>CYP152A1 *Bacillus gibsonii*

MNEQIPHDKSLDNSLTLLKEGYLFIKNRTERYNSDLFQARLLGKNFICMTGAEAAKVFYD

TDRFQRQNALPKRVQKSLFGVNAIQGMDGSAHIHRKMLFLSLMTPPHQKRLAELMTEEWK

AAVTRWEKADEVVLFEEAKEILCRVACYWAGVPLKETEVKERADDFIDMVDAFGAVGPRH

WKGRRARPRAEEWIEVMIEDARAGLLKTTPGTALHEMAFHTQEDGSQLDSRMAAIELINV

LRPIVAISYFLVFSALALHEHPKYKEWLRSGNSREREMFVQEVRRYYPFGPFLGALVKKD

FVWNNCEFKKGTSVLLDVYGTNHDPRLWDHPDEFRPERFAEREENPYDMIPQGGGHAEKG

HRCPGEGITIEVMKASLDFLVHQIEYDVPEQSLHYSLARMPSLPESGFVMSGIRRKS

>CYP102A7 *Bacillus licheniformis* ATCC 14580

MNKLDGIPIPKTYGPLGNLPLLDKNRVSQSLWKIADEMGPIFQFKFADAIGVFVSSHELV

KEVSEESRFDKNMGKGLLKVREFSGDGLFTSWTEEPNWRKAHNILLPSFSQKAMKGYHPM

MQDIAVQLIQKWSRLNQDESIDVPDDMTRLTLDTIGLCGFNYRFNSFYREGQHPFIESMV

RGLSEAMRQTKRFPLQDKLMIQTKRRFNSDVESMFSLVDRIIADRKQAESESGNDLLSLM

LHAKDPETGEKLDDENIRYQIITFLIAGHETTSGLLSFAIYLLLKHPDKLKKAYEEADRV

LTDPVPSYKQVQQLKYIRMILNESIRLWPTAPAFSLYAKEETVIGGKYLIPKGQSVTVLI

PKLHRDQSVWGEDAEAFRPERFEQMDSIPAHAYKPFGNGQRACIGMQFALHEATLVLGMI

LQYFDLEDHANYQLKIKESLTLKPDGFTIRVRPRKKEAMTAMPGAQPEENGRQEERPSAP

AAENTHGTPLLVLYGSNLGTAEEIAKELAEEAREQGFHSRTAELDQYAGAIPAEGAVIIV

TASYNGNPPDCAKEFVNWLEHDQTDDLRGVKYAVFGCGNRSWASTYQRIPRLIDSVLEKK

GAQRLHKLGEGDAGDDFEGQFESWKYDLWPLLRTEFSLAEPEPNQTETDRQALSVEFVNA

PAASPLAKAYQVFTAKISANRELQCEKSGRSTRHIEISLPEGAAYQEGDHLGVLPQNSEV

LIGRVFQRFGLNGNEQILISGRNQASHLPLERPVHVKDLFQHCVELQEPATRAQIRELAA

HTVCPPHQRELEDLLKDDVYKDQVLNKRLTMLDLLEQYPACELPFARFLALLPPLKPRYY

SISSSPQLNPRQTSITVSVVSGPALSGRGHYKGVASNYLAGLEPGDAISCFIREPQSGFR

LPEDPETPVIMVGPGTGIAPYRGFLQARRIQRDAGVKLGEAHLYFGCRRPNEDFLYRDEL

EQAEKDGIVHLHTAFSRLEGRPKTYVQDLLREDAALLIHLLNEGGRLYVCGDGSRMAPAV

EQALCEAYRIVQGASREESQSWLSALLEEGRYAKDVWDGGVSQHNVKADCIART

>CYP107H8 *Bacillus licheniformis* ATCC 14580

MTTELHEAGPSLKSDSDFWRDPYLFYDQLRSIHPVYKGTVLKHPGWYVTGYEEAAAILKE

PAFINRVPLPEASTKYEQLQRLQRNMMLFQNESGHKRMRTIAGKEFMPQKTATFRPAIEE

TVHELLDQLENKKTADMVSEFAFPLASLIIAGMLGVPEEEWYQFRQWTADLIQTIDLTRT

RKVMAKGGDTVAKLTAYFKNLIEKRKAHPSEDLISTFAGHEQLADEEVLATCILLVIAGH

ETTVNLLTNGLFLLMTHPDQLSELKENPLLIESAVEECLRYESPTQLTARTASEDCEING

KIIKKGEHLYILLGAANRDPKIFQNPHLFDITRKPNPHLAFGAGAHVCLGSALARLEAQI

AIPALLARLPNLKLASTDVPFRRLIGFRSLAELPVILN

>CYP107J9 *Bacillus licheniformis* ATCC 14580

MSSKQEESFAILTERELSSAAFKDEAYEFYKRLRASRPVCPVSMGELGEGWLITRYDDAV

HILKDARVKKNYENAFTEEELENFSALENEEPLSKHMLNADPPDHGRLRSLVQKAFTPRM

VLQLENRIQKIADSLLDQVEPNHSMNLVDDFAFPLPIIVISEMLGIPLEDRQKFRVWSQA

VIDFSDTPESLEEYKYKIGEFAEYLEYLVRKKRDEPAEDLVSALIQAESEGTKLSIEELY

ATIMLLIVAGHETTVNLITNMTLALLNHPEQLEKLRQNADLIDSAIEEALRFYSPVELTT

LRWAAEPFTLHGQEIKRKDVVIISLASANRDYMVFSNADRFDIERKDNRHLAFGHGSHFC

LGAPLARLEAKIAIQTLLRRFEHIEIKGEREQIKWKGNFLMRALEELPLSF

>CYP109A6 *Bacillus licheniformis* ATCC 14580

MANSNSLQSSKHYANWIPMKEISSSNDRLFPFPIYNRIRKTSPVRYDDERKCFDIFSYED

VQFVLKNPKLFSSKRGGNMEGKSILTMDPPRHTKMRAIVNKAFTPKAVKELEPHIEEVTA

FLFNEAKQKELFDVVDDLAAPLPVIIIAELLGVPAEDRLMFKHYSDILVAGAEDRSAEAA

ERMYKRREEGNRFLADYFKNIIKQRKKEPKDDLISLLLRAEVDGKSLTEEELLHFCIILL

VAGNETTTNLIANSVRYLTEDKITQEAVRQDPSLVPVFVEEMLRYYPPVQAIGRTAAEDV

DIGGVRIAKGSTVISWVASANRDELKFDDPDSFKLDRKSNPHMSFGFGIHFCLGAPLARL

EAKVALDYLLRRAYMERDSSKELEAIQSPFVFGVRHLPVQLSQK

>CYP109B4 *Bacillus licheniformis* ATCC 14580

MSQTKQQNPIQKALINGKNRQDPYDPFPWYKKMRTESPIHYDEDSKVWSVFRYDDVKRVI

SDKDFFSNQFPQLGTGNTFAKTMISMDPPKHTRIRSIVNKAFTPRVMKEWEPRIRELTNQ

LLADVRGREEIDLVQDFSYPLPVIVISELLGVPLVYKHHFKEWSDLLVSLPKSDRPEDVN

EWKNIRDQGEEELTAFFEKMIEEKRQNLGNDLISLLIKAEQEGDKLSPDELVPFCNLLLM

AGNETTTNLVSNAVYSILETPGVYDELARHPELIPQAVEEAVRFRAPAPMIVRFVKQDTE

IRGVSLKKGEGVIAFLASANRDETKFERAHEFDIHRHPNPHIGFGHGIHFCLGAPLARLE

AAIAIEALLKQYASMEKLAVVPMADSSMYGLKHFRLRVKSAGETAPN

>CYP134A5 *Bacillus licheniformis* ATCC 14580

MNQSLKTFSVLSEQYHENPYQYFSYLRESDPVHYEESLDSYFISRYQDVRRVLQNQDVFT

TKSLAKRAEPVMRGPVLAQMKGKEHTAKRRIVLRRFIGESLDHLTPLIKENAQRLLAPHV

EKGRIDLVNDFGKTFAVCVTMDILGLDKNDHQTVRNWHSGVADFITSLNQAPEDREHSLK

CSEQLAEYLNPIIEERRKNPGHDLISILCTSEYEGVAMSDRDIRALILNILLAATEPADK

TLALMIYHLLHHPDQMNDVLEDRTLLPQAIAETLRYKPPVQLIPRQLSQDAEIGGVELKE

GTTVFCMIGAANRDPEAFEDPDKFNIHRSDLEVKSAFSGAARHLAFGSGVHNCVGAGFAK

TEIELVANIVLDQLKNIRLEEDFIYRETGLYTRGPVSLNIRFDAKH

>CYP102A7 *Bacillus licheniformis* DSM 13 = ATCC 14580

MNKLDGIPIPKTYGPLGNLPLLDKNRVSQSLWKIADEMGPIFQFKFADAIGVFVSSHELV

KEVSEESRFDKNMGKGLLKVREFSGDGLFTSWTEEPNWRKAHNILLPSFSQKAMKGYHPM

MQDIAVQLIQKWSRLNQDESIDVPDDMTRLTLDTIGLCGFNYRFNSFYREGQHPFIESMV

RGLSEAMRQTKRFPLQDKLMIQTKRRFNSDVESMFSLVDRIIADRKQAESESGNDLLSLM

LHAKDPETGEKLDDENIRYQIITFLIAGHETTSGLLSFAIYLLLKHPDKLKKAYEEADRV

LTDPVPSYKQVQQLKYIRMILNESIRLWPTAPAFSLYAKEETVIGGKYLIPKGQSVTVLI

PKLHRDQSVWGEDAEAFRPERFEQMDSIPAHAYKPFGNGQRACIGMQFALHEATLVLGMI

LQYFDLEDHANYQLKIKESLTLKPDGFTIRVRPRKKEAMTAMPGAQPEENGRQEERPSAP

AAENTHGTPLLVLYGSNLGTAEEIAKELAEEAREQGFHSRTAELDQYAGAIPAEGAVIIV

TASYNGNPPDCAKEFVNWLEHDQTDDLRGVKYAVFGCGNRSWASTYQRIPRLIDSVLEKK

GAQRLHKLGEGDAGDDFEGQFESWKYDLWPLLRTEFSLAEPEPNQTETDRQALSVEFVNA

PAASPLAKAYQVFTAKISANRELQCEKSGRSTRHIEISLPEGAAYQEGDHLGVLPQNSEV

LIGRVFQRFGLNGNEQILISGRNQASHLPLERPVHVKDLFQHCVELQEPATRAQIRELAA

HTVCPPHQRELEDLLKDDVYKDQVLNKRLTMLDLLEQYPACELPFARFLALLPPLKPRYY

SISSSPQLNPRQTSITVSVVSGPALSGRGHYKGVASNYLAGLEPGDAISCFIREPQSGFR

LPEDPETPVIMVGPGTGIAPYRGFLQARRIQRDAGVKLGEAHLYFGCRRPNEDFLYRDEL

EQAEKDGIVHLHTAFSRLEGRPKTYVQDLLREDAALLIHLLNEGGRLYVCGDGSRMAPAV

EQALCEAYRIVQGASREESQSWLSALLEEGRYAKDVWDGGVSQHNVKADCIART

>CYP107H8 *Bacillus licheniformis* DSM 13 = ATCC 14580

MTTELHEAGPSLKSDSDFWRDPYLFYDQLRSIHPVYKGTVLKHPGWYVTGYEEAAAILKE

PAFINRVPLPEASTKYEQLQRLQRNMMLFQNESGHKRMRTIAGKEFMPQKTATFRPAIEE

TVHELLDQLENKKTADMVSEFAFPLASLIIAGMLGVPEEEWYQFRQWTADLIQTIDLTRT

RKVMAKGGDTVAKLTAYFKNLIEKRKAHPSEDLISTFAGHEQLADEEVLATCILLVIAGH

ETTVNLLTNGLFLLMTHPDQLSELKENPLLIESAVEECLRYESPTQLTARTASEDCEING

KIIKKGEHLYILLGAANRDPKIFQNPHLFDITRKPNPHLAFGAGAHVCLGSALARLEAQI

AIPALLARLPNLKLASTDVPFRRLIGFRSLAELPVILN

>CYP107J9 *Bacillus licheniformis* DSM 13 = ATCC 14580

MSSKQEESFAILTERELSSAAFKDEAYEFYKRLRASRPVCPVSMGELGEGWLITRYDDAV

HILKDARVKKNYENAFTEEELENFSALENEEPLSKHMLNADPPDHGRLRSLVQKAFTPRM

VLQLENRIQKIADSLLDQVEPNHSMNLVDDFAFPLPIIVISEMLGIPLEDRQKFRVWSQA

VIDFSDTPESLEEYKYKIGEFAEYLEYLVRKKRDEPAEDLVSALIQAESEGTKLSIEELY

ATIMLLIVAGHETTVNLITNMTLALLNHPEQLEKLRQNADLIDSAIEEALRFYSPVELTT

LRWAAEPFTLHGQEIKRKDVVIISLASANRDYMVFSNADRFDIERKDNRHLAFGHGSHFC

LGAPLARLEAKIAIQTLLRRFEHIEIKGEREQIKWKGNFLMRALEELPLSF

>CYP109A6 *Bacillus licheniformis* DSM 13 = ATCC 14580

MANSNSLQSSKHYANWIPMKEISSSNDRLFPFPIYNRIRKTSPVRYDDERKCFDIFSYED

VQFVLKNPKLFSSKRGGNMEGKSILTMDPPRHTKMRAIVNKAFTPKAVKELEPHIEEVTA

FLFNEAKQKELFDVVDDLAAPLPVIIIAELLGVPAEDRLMFKHYSDILVAGAEDRSAEAA

ERMYKRREEGNRFLADYFKNIIKQRKKEPKDDLISLLLRAEVDGKSLTEEELLHFCIILL

VAGNETTTNLIANSVRYLTEDKITQEAVRQDPSLVPVFVEEMLRYYPPVQAIGRTAAEDV

DIGGVRIAKGSTVISWVASANRDELKFDDPDSFKLDRKSNPHMSFGFGIHFCLGAPLARL

EAKVALDYLLRRAYMERDSSKELEAIQSPFVFGVRHLPVQLSQK

>CYP109B4 *Bacillus licheniformis* DSM 13 = ATCC 14580

MSQTKQQNPIQKALINGKNRQDPYDPFPWYKKMRTESPIHYDEDSKVWSVFRYDDVKRVI

SDKDFFSNQFPQLGTGNTFAKTMISMDPPKHTRIRSIVNKAFTPRVMKEWEPRIRELTNQ

LLADVRGREEIDLVQDFSYPLPVIVISELLGVPLVYKHHFKEWSDLLVSLPKSDRPEDVN

EWKNIRDQGEEELTAFFEKMIEEKRQNLGNDLISLLIKAEQEGDKLSPDELVPFCNLLLM

AGNETTTNLVSNAVYSILETPGVYDELARHPELIPQAVEEAVRFRAPAPMIVRFVKQDTE

IRGVSLKKGEGVIAFLASANRDETKFERAHEFDIHRHPNPHIGFGHGIHFCLGAPLARLE

AAIAIEALLKQYASMEKLAVVPMADSSMYGLKHFRLRVKSAGETAPN

>CYP134A5 *Bacillus licheniformis* DSM 13 = ATCC 14580

MNQSLKTFSVLSEQYHENPYQYFSYLRESDPVHYEESLDSYFISRYQDVRRVLQNQDVFT

TKSLAKRAEPVMRGPVLAQMKGKEHTAKRRIVLRRFIGESLDHLTPLIKENAQRLLAPHV

EKGRIDLVNDFGKTFAVCVTMDILGLDKNDHQTVRNWHSGVADFITSLNQAPEDREHSLK

CSEQLAEYLNPIIEERRKNPGHDLISILCTSEYEGVAMSDRDIRALILNILLAATEPADK

TLALMIYHLLHHPDQMNDVLEDRTLLPQAIAETLRYKPPVQLIPRQLSQDAEIGGVELKE

GTTVFCMIGAANRDPEAFEDPDKFNIHRSDLEVKSAFSGAARHLAFGSGVHNCVGAGFAK

TEIELVANIVLDQLKNIRLEEDFIYRETGLYTRGPVSLNIRFDAKH

>CYP102A51 *Bacillus megaterium* NBRC 15308 = ATCC 14581

MTIKEMPQPKTFGELKNLPLLNTDKPVQALMKIADELGEIFKFEAPGRVTRYLSSQRLIK

EACDESRFDKNLSQALKFVRDFAGDGLFTSWTHEKNWKKAHNILLPSFSQQAMKGYHAMM

VDIAVQLVQKWERLNADEHIEVPEDMTRLTLDTIGLCGFNYRFNSFYRDQPHPFITSMVR

ALDEAMNKLQRANPDDPAYDENKRQFQEDIKVMNDLVDKIIADRKASGEQSDDLLTHMLN

GKDPETGEPLDDENIRYQIITFLIAGHETTSGLLSFALYFLVKNPHVLQKAAEEAARVLV

DPVPSYKQVKQLKYVGMVLNEALRLWPTAPAFSLYAKEDTVLGGEYPLEKGDELMVLIPQ

LHRDKTIWGDDVEEFRPERFENPSAIPQHAFKPFGNGQRACIGQQFALHEATLVLGMMLK

HFDFEDHTNYELDIKETLTLKPEGFVVKAKSKKIPLGGIPSPSTEQSAKKVRKKAENAHN

TPLLVLYGSNMGTAEGTARDLADIAMSKGFAPQVATLDSHAGNLPREGAVLIVTASYNGH

PPDNAKQFVDWLDQASADEVKGVRYSVFGCGDKNWATTYQKVPAFIDETLAAKGAENIAD

RGEADASDDFEGTYEEWREHMWSDVAAYFNLDIENSEDNKSTLSLQFVDSAADMPLAKMH

GAFSTNVVASKELQQPGSARSTRHLEIELPKEASYQEGDHLGVIPRNYEGIVNRVTARFG

LDASQQIRLEAEEEKLAHLPLAKTVSVEELLQYVELQDPVTRTQLRAMAAKTVCPPHKVE

LEALLEKQAYKEQVLAKRLTMLELLEKYPACEMKFSEFIALLPSIRPRYYSISSSPRVDE

KQASITVSVVSGEAWSGYGEYKGIASNYLAELQEGDTITCFISTPQSEFTLPKDPETPLI

MVGPGTGVAPFRGFVQARKQLKEQGQSLGEAHLYFGCRSPHEDYLYQEELENAQSEGIIT

LHTAFSRMPNQPKTYVQHVMEQDGKKLIELLDQGAHFYICGDGSQMAPAVEATLMKSYAD

VHQVSEADARLWLQQLEEKGRYAKDVWAG

>CYP106A1 *Bacillus megaterium* NBRC 15308 = ATCC 14581

MNKEVIPVTEIPKFQSRAEEFFPIQWYKEMLNNSPVYFHEETNTWNVFQYEHVKQVLSNY

DFFSSDGQRTTIFVGDNSKKKSTSPITNLTNLDPPDHRKARSLLAAAFTPRSLKNWEPRI

KQIAADLVEAIQKNSTINIVDDLSSPFPSLVIADLFGVPVKDRYQFKKWVDILFQPYDQE

RLEEIEQEKQRAGAEYFQYLYPIVIEKRSNLSDDIISDLIQAEVDGETFTDEEIVHATML

LLGAGVETTSHAIANMFYSFLYDDKSLYSELRNNRELAPKAVEEMLRYRFHISRRDRTVK

QDNELLGVKLKKGDVVIAWMSACNMDETMFENPFSVDIHRPTNKKHLTFGNGPHFCLGAP

LARLEMKIILEAFLEAFSHIEPFEDFELEPHLTASATGQSLTYLPMTVYR

>CYP109A2 *Bacillus megaterium* NBRC 15308 = ATCC 14581

MNPKAVKRENRYANLIPMQEIKSVEQQLYPFDIYNSLRQEAPIRYDESRNCWDVFDYETV

KYILKNPSLFSSKRAMEERQESILMMDPPKHTKLRNLVNKAFTPRAIQHLEGHIEEIADY

LLDEVSSKEKFDIVEDFAGPLPIIVIAELLGVPIQDRALFKKYSDDLVSGAENNSDEAFA

KMMQKRNEGVIFLQGYFKEIIAERQQNKQEDLISLLLEAEIDGEHLTEEEVLGFCILLLV

AGNETTTNLITNGVRYMTEDVDVQNEVRRDISLVPNLVEETLRYYPPIQAIGRIAAEDVE

LGECKIKRGQQVISWAASANRDSAKFEWPDTFVVHRKTNPHVSFGFGIHFCLGAPLARME

GKIAFTKLLEKGGFSKVQNQSLKPIDSPFVFGVKKYEIAFNNA

>CYP109E1 *Bacillus megaterium* NBRC 15308 = ATCC 14581

MKTERENGIVRQVNTIQTKEERFNPFSWYEEMRNTEPVQWDEKRQVWDVFHYDGVKEVLE

QKNIFSSDRRPPQNQRQTALGTSLINIDPPKHAEMRALVNKAFTPKAMKAWEPKIARITN

ELLQEVEHLEDIDIVEHLSYPLPVMVIADILGVPIEDQRQFKDWSDIIVAGPSNNERETL

EKLQQEKMKANDELETYFYRIIEEKRTRPGDDIISLLLQAKEEGKQLTDEEIVGFSILLL

IAGNETTTNLISNTIYCLMEDKASFERLKREKELLPSAIEEVLRYRSPVQALHRIVKEDV

TLAGKKLKAGEHVVPWMGSAHRDAEYFEDPDVFKIDRKPNVHMAFGRGIHFCLGAPLARI

EAKIMLAELIDRYPQMDWSPSFELKPIESTFVYGLKELLIRKNV

>CYP152K5 *Bacillus megaterium* NBRC 15308 = ATCC 14581

MCRTACQWAGVPIQEGKVRELTKDLGAMFESPAAIGPNHWLGRNTRNRVGKWIGELIDEV

RNGNMNVPTNIILYRFTQYRDLEGNLLDTNTAAVEVINILRPIVAIAIFINFIMLSLHHF

PEEKGKLKSRDEEYAQIFIQEVRRFYPFFPFVTALVKKDFTWNNYKFEEGTLTLLDLYGT

NHDPKIWNNPDIFNPDRFTKWEGSPFSFIPQGGGDYFMGHRCAGEWVTLEVMKVSLDYLV

NRIEYEIPDQDLNFSMVDIPSIPHSKIVIKNVVTAKVTWLFLTFGEGAL

>CYP1756A1 *Bacillus megaterium* NBRC 15308 = ATCC 14581

MENLTQYGEIATFRVAHKRFYVTRDPQLIKDVVITNSKAFQKIKLTHMFKTLLGEEMLWT

DEALYMSPIQPSQLKQHLTFNKEAIAKIIEKHTETWEEGQLRTIVKDIRQIVIAVLLQLV

FGISIEEKDKIHYVQALMRKKEKLGKIYIRLPLHQPDSDEQLEQLLFERVQMRVQNKTAG

NDLLQYILNSYGEDSDEREIYEQLNSIFLSMYEMITHVCSWSIHLLSQNTREHLQLHKEI

QAYASGESLSTKNLTYMRKIIAESMRLYPPLWLFGRQAREDIQIDGYSIKKGEIMLISPY

MMHRHEDYFLEPSEFLPDRFEKGGSIDVPSYMYMPLGIEHQAERGMDYITEIVTIFLSEM

TKRFLFQLTKPESIAPMAGVMLNMKEELKVNVHKVHAQS

>CYP102A51 *Bacillus megaterium* QM B1551

MPIKEMPQPKTFGELKNLPLLNTDKPIQTLMKIADELGEIFKFEAPGRVTRYLSSQRLIK

EACDESRFDKNLSQALKFVRDFAGDGLFTSWTHEKNWKKAHNILLPSFSQQAMKGYHAMM

VDIAVQLIQKWERLNTDEHIEVPEDMTRLTLDTIGLCGFNYRFNSFYRDQPHPFITSMVR

ALDEAMNKLQRANPDDPAYDENKRQFQEDIKVMNDLVDKIITDRKASGEQSDDLLTHMLN

GKDPETGEPLDDENIRYQIITFLIAGHETTSGLLSFALYFLVKNPHVLQKAAEEAARVLV

DPVPSYKQVKQLKYVGMVLNEALRLWPTAPAFSLYAKEDTVLGGEYPLEKGDELMVLIPQ

LHRDKTIWGDDVEEFRPERFENPSAIPQHAFKPFGNGQRACIGQQFALHEATLVLGMMLK

HFDFEDHTNYELDIKETLTLKPEGFVVKAKSKQIPLGGIPSPSREQSAKKERKTVENAHN

TPLLVLYGSNMGTAEGTARDLADIAMSKGFAPQVATLDSHAGNLPREGAVLIVTASYNGH

PPDNAKEFVDWLDQASADEVKGVRYSVFGCGDKNWATTYQKVPAFIDETLAAKGAENIAE

RGEADASDDFEGTYEEWREHMWSDLAAYFNLDIENSEENASTLSLQFVDSASDMPLAKMH

RAFSANVVASKELQKPGSARSTRHLEIELPKEASYQEGDHLGVIPRNYEGIVNRVATRFG

LDASQQIRLEAEEEKLAHLPLGKTVSVEELLQYVELQDPVTRTQLRAMAAKTVCPPHKVE

LEVLLEKQAYKEQVLAKRLTMLELLEKYPACEMEFSEFIALLPSMRPRYYSISSSPRVDE

KQASITVSVVSGEAWSGYGEYKGIASNYLANLQEGDTITCFVSTPQSGFTLPKGPETPLI

MVGPGTGVAPFRGFVQARKQLKEQGQSLGEAHLYFGCRSPHEDYLYQKELENAQNEGIIT

LHTAFSRVPNQPKTYVQHVMEQDGKKLIELLDQGAHFYICGDGSQMAPDVEATLMKSYAE

VHQVSEADARLWLQQLEEKGRYAKDVWAG

>CYP106A1 *Bacillus megaterium* QM B1551

MNKEVIPVTEIPKFQSRAEEFFPIQWYKEMLNNHPVYFHEETNTWNVFQYDHVKQVLSNY

EFFSSDGQRTTIFVGDNSKKKSTSPITNLTNLDPPDHRKVRSLLAAAFTPRSLKNWEPRI

KQIAAELVEAIQKNLTIDIVDDLSSPFPSLVIADLFGVPVKDRYQFKKWVDILFQPYDQE

RLEKIEQEKQRAGAEYFQYLYPIVIEKRSNLSDDIISDLIQAEVDGETFTDEEIVHATML

LLGAGVETTSHAIANMFYSFLYDDESLYSELRNNRELAPKAVEEMLRYRFHISRRDRTVK

QDNDLLGVKLKKGDVVIAWMSACNMDENMFKTPFSVDIHRPTNKKHLTFGNGPHFCLGAP

LARLEMTIILEAFLEAFSHIEPFEDFELESHLTASATGQSLTYLPMTVYR

>CYP107DY1 *Bacillus megaterium* QM B1551

MKKVTVDDFSSPENMHDVIGFYKKLTEHQEPLIRLDDYYGLGPAWVALRHDDVVTILKNP

RFLKDVRKFTPLQDKKDSIDDSTSASKLFEWMMNMPNMLTVDPPDHTRLRRLASKAFTPR

MIENLRPRIQQITNELLDSVEGKRNMDLVADFSFPLPIIVISEMLGIPPLDQKRFRDWTD

KLIKAAMDPSQGAVVMETLKEFIDYIKKMLVEKRNHPDDDVMSALLQAHEQEDKLSENEL

LSTIWLLITAGHETTAHLISNGVLALLKHPEQMRLLRDNPSLLPSAVEELLRYAGPVMIG

GRFAGEDIIMHGKMIPKGEMVLFSLVAANIDSQKFSYPEGLDITREENEHLTFGKGIHHC

LGAPLARMEAHIAFGTLLQRFPDLRLAIESEQLVYNNSTLRSLKSLPVIF

>CYP109A2 *Bacillus megaterium* QM B1551

MNSKAVKRENRYANWIPMQEIKSVEQQLYPFDIYNSLRQEAPIRYDESRNCWDVFDYETV

KYILKNPSLFSSKRAMEERQESILMMDPPKHTKLRNLVNKAFTPRAIQHLEGHIEEIADY

LLDEVSSKKKFDIVEDFAGPLPIIVIAELLGVPIQDRALFKKYSDDLVSGAENNSDEAFA

KMMQKRNEGVIFLQGYFKEIIAERQQNKQEDLISLLLEAEIDGEHLTEEEVLGFCILLLV

AGNETTTNLITNGVRYMTEDVDVQNEVRRDISLVPNLVEETLRYYPPIQAIGRIAAEDVE

LGECKIKKGQQVISWAASANRDSAKFERPDTFVVHRKTNPHVSFGFGIHFCLGAPLARME

GKIAFTKLLEKGEFSKVQNQSLKPIGSPFVFGVKKYEITFNKA

>CYP109E1 *Bacillus megaterium* QM B1551

MKTERENGIVRQVNTIQTKEERFNPFSWYEEMRNSAPVQWDEERQVWDVFHYDGVKEVLE

QKNIFSSDRRPPQNQRQTALGTSLINIDPPKHAEMRSLVNKAFTPKAMKAWEPKIARITN

ELLQEVEHLEDIDIVEHLSYPLPVMVIADILGVPIEDQRQFKDWSDIIVAGPSNNERETL

EKLQQEKMKANDELETYFYRIIEEKRTRPGDDIISVLLQAKEEGKQLTDEEIVGFSILLL

IAGNETTTNLISNTIYCLMEDKASFERLKREKELLPSAIEEVLRYRSPVQALHRIVKEDV

ILAGQKLKAGEHVVPWMGSAHRDAEYFEDPDVFKIDRKPNVHMAFGRGIHFCLGAPLARI

EAKIMLAELIDRYPQMDWSPAFELKPIESTFVYGLKELLIRKNV

>CYP1756A1 *Bacillus megaterium* QM B1551

MENLTQYGEIATFRVAHKRFYVTRDPQLIKDVVITNSKAFQKIKLTHMFKTLLGEEMLWT

DEALYMSPIQPSQLKQHLTYNKEVIAKIIEKHTETWEEGQLRTIVKDIRQIVVAVLLQLV

FGISIQDKDKIHYVQALMRKKEKLGKIYIRLPLHQPDSDEQLEQLLFERVQMRVQNKTEG

NDLLEYILNSCGEEIDEREIYEQLKSIFLSMYEMITHVCSWSIHLLSQNTREHLQLHKEI

QAYASGESLSTKNLTYMRKIIAESMRLYPPLWLFGRQAREDIQIDGYSIKKGEIMLISPY

MMHRHEDYFLEPSEFLPDRFEKGGSIDVPSYMYMPLGIEHQAERGMDYITEIVTIFMSEM

TKRFLFQLTKPESIAPMAGVMLNMKEELNVNVHKVHTQS

>CYP102A36 *Bacillus sp*. LM 4-2

MKETSPIPQPKTFGPLGNLPLIDKDKPTLSLIKLAEEQGPIFQIHTPAGTTIVVSGHELV

KEVCDEERFDKSIEGALEKVRAFSGDGLFTSWTHEPNWRKAHNILMPTFSQRAMKDYHEK

MVDIAVQLIQKWARLNPNEAVDVPGDMTRLTLDTIGLCGFNYRFNSYYRETPHPFINSMV

RALDEAMHQMQRLDVQDKLMVRTKRQFRHDIQTMFSLVDSIIAERRANGDQDEKDLLARM

LNVEDPETGEKLDDENIRFQIITFLIAGHETTSGLLSFATYFLLKHPDKLKKAYEEVDRV

LTDAAPTYKQVLELTYIRMILNESLRLWPTAPAFSLYPKADTVIGGKFPITTNDRISVLI

PQLHRDRDAWGKDAEEFRPERFEHQDQVPHHAYKPFGNGQRACIGMQFALHEATLVLGMI

LKYFTLIDHENYELDIKQTLTLKPGDFHIRVQSRHLEAIHADVRAAEKAASDEQKEKTEA

KGTSVIGLNNRPLLVLYGSDTGTAEGVARELADTASLHGVRTETAPLNDRIGKLPKEGAV

VIVTSSYNGKPPSNAGQFVQWLQEIKPGELEGVHYAVFGCGDHNWASTYQYVPRFIDEQL

AEKGATRFSARGEGDVSGDFEGQLDEWKKSMWADAIKAFGLELNENADKERSTLSLQFVR

GLGESPLARSYEASHASIAENRELQSADSDRSTRHIEIALPPDVEYQEGDHLGVLPKNSQ

TNVNRILHRFGLKGTDQVTLSASGRSAGHLPLGRPVSLHDLLSYSVEVQEAATRAQIREL

AAFTVCPPHKRELEELTAEGVYQEQILKKRISMLDLLEKYEACDMPFERFLELLRPLKPR

YYSISSSPRVNPGQASITVGVVRGPAWSGRGEYRGVASNDLAERQAGDDVVMFIRTPESR

FQLPKDPETPIIMVGPGTGVAPFRGFLQAREVLKREGKTLGEAHLYFGCRNDRDFIYRDE

LEQFEKDGIVTVHTAFSRKEGMPKTYVQHVMAAHAETLISIFDRGGRLYVCGDGSKMAPD

VEAALQKAYQSVHGTGEQEAQNWLRHLQDTGMYAKDVWAGI

>CYP102A48 *Bacillus sp.* LM 4-2

MKQASAIPQPKTYGPLKNLPHLEKEQLSQSLWRIADELGPIFRFDFPGVSSVFVSGHNLV

AEVCDEKRFDKNLGKGLQKVREFGGDGLFTSWTHEPNWQKAHRILLPSFSQKAMKGYHSM

MLDIATQLIQKWSRLNPNEEIDVADDMTRLTLDTIGLCGFNYRFNSFYRDSQHPFITSML

RALKEAMNQSKRLGLQDKMMVKTKLQFQKDIEVMNSLVDRMIAERKANPDENIKDLLSLM

LYAKDPVTGETLDDENIRYQIITFLIAGHETTSGLLSFAIYCLLTHPEKLKKAQEEADRV

LTDDTPEYKQIQQLKYIRMVLNETLRLYPTAPAFSLYAKEDTVLGGEYPISKGQPVTVLI

PKLHRDQNAWGPDAEDFRPERFEDPSSIPHHAYKPFGNGQRACIGMQFALQEATMVLGLV

LKHFELINHTGYELKIKEALTIKPDDFKITVKPRKTAAINVQRKEQADIKAETKPKETKP

KHGTPLLVLFGSNLGTAEGIAGELAAQGRQMGFTAETAPLDDYIGKLPEEGAVVIVTASY

NGAPPDNAAGFVEWLKELEEGQLKGVSYAVFGCGNRSWASTYQRIPRLIDDMMKAKGASR

LTAIGEGDAADDFESHRESWENRFWKETMDAFDINEIAQKEDRPSLSITFLSEATETPVA

KAYGAFEGIVLENRELQTAASTRSTRHIELEIPAGKTYKEGDHIGILPKNSRELVQRVLS

RFGLQSNHVIKVSGSAHMAHLPMDRPIKVVDLLSSYVELQEPASRLQLRELASYTVCPPH

QKELEQLVSDDGIYKEQVLAKRLTMLDFLEDYPACEMPFERFLALLPSLKPRYYSISSSP

KVHANIVSMTVGVVKASAWSGRGEYRGVASNYLAELNTGDAAACFIRTPQSGFQMPNDPE

TPMIMVGPGTGIAPFRGFIQARSVLKKEGSTLGEALLYFGCRRPDHDDLYREELDQAEQD

GLVTIRRCYSRVENEPKGYVQHLLKQDTQKLMTLIEKGAHIYVCGDGSQMAPDVERTLRL

AYEAEKAASQEESAVWLQKLQDQRRYVKDVWTGM

>CYP107H1 *Bacillus sp*. LM 4-2

MMSIAFIFEQKGENHVTIASSTASSEFLKNPYSFYDTLRAVHPIYKGSFLKYPGWYVTGY

EETAAILKDARFKVRTPLPESSTKYQDLSHVQNQMMLFQNQPDHRRLRTLASGAFTPRTT

ESYQPYIIETVHQLLDQVQGEKKMEVISDFAFPLASFVIANIIGVPEEDREQLKEWAASL

IQTIDFTRSRKALTEGNHMAVQAMAYFKELIQKRKRHPQQDMISMLLKGREKDKLTEEEA

ASTCILLAIAGHETTVNLISNSVLCLLQHPEQILKLRENPDLIGTAVEECLRYESPTQMT

ARVASEDIDICGVTIRQGEQVYLLLGAANRDPSIFTNPDVFDITRSPNPHLSFGHGYHVC

LGSSLARLEAQIAINTLLQRMPSLKLADFEWRYRPLFGFRALEELPVTFE

>CYP107K1 *Bacillus sp*. LM 4-2

MEKLMFHPHGKEFHHNPFSVLGRFREEEPIHRFELKRFGATYPAWLITRYDDCMAFLKDN

RITRDVKNVMNQEQIKMLNVSEDIDFVSDHMLAKDTPDHTRLRSLVHQAFTPRTIENLRG

SIEQIAEQLLDEMEKENKADIMKSFASPLPFIVISKLMGIPKEDRSQFQIWTNAMVDTSE

GNRELTNQALREFKDYIAKLIHDRRIKPKDDLISKLVHAEENGSKLSEKELYSMLFLLVV

AGLETTVNLLGSGTLALLQHKKECEKLKQHPEMIATAVEELLRYTSPVVMMANRWAIEDF

TYKGHSIKRGDMIFIGIGSANRDPNFFENPEILNINRSPNRHISFGFGIHFCLGAPLARL

EGHIAFNALLKRFPDIELAVAPDDIQWRKNVFLRGLESLPVSLSK

>CYP152A1 *Bacillus sp*. LM 4-2

MNEQIPHDKSLDNSLILLKEGYLFIKNRTERYNSDLFQARLLGKNFICMTGAEAAKVFYD

TDRFQRQNALPKRVQKSLFGVNAIQGMDGSAHIHRKMLFLSLMTPPHQKRLAELMTEEWK

AAVTRWEKADEVVLFEEAKEILCRVACYWAGVPLKETEVKERADDFIDMVDAFGAVGPRH

WKGRRARPRAEEWIEVMIEDARAGLLKTTSGTALHEMAFHTQEDGSQLDSRMAAIELINV

LRPIVAISYFLVFSALALHEHPKYKEWLRSGNSREREMFVQEVRRYYPFGPFLGALVKKD

FIWNNCEFKKGTSVLLDLYGTNHDPRLWDHPDEFRPERFAEREENLFDMIPQGGGHAEKG

HRCPGEGITIEVMKASLDFLVHQIEYDVPEQSLHYSLARMPSLPESGFVMSGIRRKS

>CYP134A1 *Bacillus sp*. LM 4-2

MSQSIKLFSVLSDQFQNNPYAYFSQLREEDPVHYEESIDSYFISRYHDVRYILQHPDIFT

TKSLVERAEPVMRGPVLAQMHGKEHSAKRRIVVRSFIGDALDHLSPLIKQNAENLLAPYL

ERGKSDLVNDFGKTFAVCVTMDMLGLDKRDHEKISEWHSGVADFITSISQSPEARAHSLW

CSEQLSQYLMPVIKERRVNPGSDLISILCTSEYEGMALSDKDILALILNVLLAATEPADK

TLALMIYHLLNNPEQMNDVLADRSLVPRAIAETLRYKPPVQLIPRQLSQDTVVGGMEIKK

DTIVFCMIGAANRDPEAFEQPDVFNIHREDLGIKSAFSGAARHLAFGSGIHNCVGAAFAK

NEIEIVANIVLDKMRNIRLEEDFCYAESGLYTRGPVSLLVAFDGA

>CYP102A38 *Bacillus sp.* Pc3

MKETGPIPQPKTFGPLGNLPLLDKDKPTMSLIKLANEQGPIFQLHTPAGAIIVVSGHELV

KEVCDEERFDKSIEGALEKVRAFSGDGLFTSWTHEPNWRKAHHILMPTFSQRAMKDYHSM

MTDIAVQLIQKWARLNPDEAVDVPADMTRLTLDTIGLCGFNYRFNSYYRETPHPFINSMV

RALDEAMHQMQRLDVQDKLMIRTKRQFHHDIQAMFSLVDSIIAERRSGGRDEKDLLARML

NVEDPETGEKLDDENIRFQIITFLIAGHETTSGLLSFAIYFLLKHPRVLEKAYEEADRVL

TDPVPSYKQVLELSYIRMILQESLRLWPTAPAFSLYAKEDTVIGGKYPITPKDRISVLIP

QLHRDKDAWGDNAEEFYPERFEHPDQVPHHAYKPFGNGQRACIGMQFALHEATLVLGMIL

QHFTFIDHTDYELDIKQTLTIKPGDFHIRVRPRNKEAVAAALPAAEKAAEDVEKEKRETK

GASIIGLDNRPLLILYGSDTGTAEGVARELADTAGMHGVRTETAPLNDRIGKLPKEGALL

IITSSYNGKPPSNAGQFVQWLEEVKPGELEGVRYAVFGCGDHNWAATYQAVPRLIDEKLA

EKGAERFSSRGEGDVSGDFEGKLDEWKKSMWTDAMKAFGLKLNENAEKERSALGLQFVSG

LGGSPLAQTYEAVYASVAENRELQAPESGRSTRHIEITLPKEAAYHEGDHLGVLPVNSKE

QVSRVLRRYNLNGNDQVLLTASGQSAAHLPLDRPVRLHDLLSSCVELQEAASRAQIREMA

AYTVCPPHKRELEDFLEEGVYHEQILTSRVSMLDLLEKYEACELPFERFLELLRPLKPRY

YSISSSPRKHPGQASITVGVVRGPARSGLGEYRGVASNYLADRGPEDGIVMFVRTPETRF

RLPEDSEKPIIMVGPGTGVAPFRGFLQARAALKKEGKELGEAHLYFGCRNDHDFIYRDEL

EAYEKDGIVTLHTAFSRKEGVPKTYVQHLMAKDAGALISILGRGGHLYVCGDGSKMAPDV

EATLQKAYKSVHETDERQAQEWLLDLQTKGIYAKDVWAGI

>CYP102A49 *Bacillus sp*. Pc3

MKQLSAIPQPKTYGPLKNLPHLEKEKLSQSLWKIAEEYGPIFRFEFPSSVGVFVSGRELA

AEVCDEKRFDKNLSKALLKVREFGGDGLFTSWTHEKNWQKAHRILLPSFSQKAMKGYHSM

MLDIAMQLVQKWSRLNPNEEIDVAEDMTRLTLDTIGLCGFHYRFNSFYRDTQHPFITSML

RALQEAMRQSQRHSLQDKLMIKTRHQFQQDIEEMNSLVDRIIAERRENPDENLSDLLALM

LEAKDPVTGERLDDENIRYQIITFLIAGHETTSGLLSFAIYCLLKNKDKLKKAVQEAERV

LTGETPEYKQIQQLTYIRMVLNETLRLYPTAPAFSLYAKEDTVLGGKYPIAKGQPVTILT

PQLHRDKSAWGEDAELFRPERFSDPAAIPADAYKPFGNGQRACIGMQFALQEATMVLGLV

LKHFELIDHTDYELTIKEALTIKPGDFKIRVKNKDVSNHQPVQNHKAEGSGHKEETKEIP

SHGTPLLILYGSNLGTAEGIAEELADIGRSKGFSTETGPLDDYAGKLPVKGAVVIVTASY

NGAPPDNAAGFVKWMETLEDQELKGVSYAVFGCGDRNWAATYQRIPRLIDKVLEEKGAKR

LTSIGEGDNADDFEYSQEVWEDSFWKDIMKAFHIEAAPKQTNKSQLSIEYVSEAAETPIA

KTYKAFEAEVITNKELHTESSKRSVRHIELRLPETETYQEGDHLGVLPQNSGELISRVIR

RFGLDPNQHFKIKGRQLPHLPMDRPVNAPELLASYVELQEPATRAQLRELAAHTVCPPHQ

KELEHLYSDDAAYKENVLKKRMTMLDLLEDYPACELPFERFLELLPSLKARYYSISSSPK

AASGELSITVGVVTAPAWSGRGEYRGVASNYLAGLQKGDSAVCFIRSPQSGFALPENPKT

PLIMVGAGTGIAPFRGFIQARAAEKMSGNSLGEAHLYFGCRHPEEDDLYKDEFDHAEKNG

LVTVHRAYSRLNQDCKVYVQDVLLREAAQIIALLDQGGHLYICGDGSKMAPAVENVLLQA

YEKVHNTDSKVSLEWLEQLQAEGRYAKDVWAGM

>CYP107H4 *Bacillus sp*. Pc3

MTADLSIAHPSLHADSDFWNDPYPFYDKLRSSDPVYKGTVLKYPGWYVTGYKEAAAILKD

IRFKNRIPLPEASTKYQNLSHIQHDMLLFKNQSDHKRMRMLIGKEFTAKTAESLRPCIKE

TVHDLLDQVQIKKTADLVSEFAFPLASLIIAEILGVPKEERYQFRQWTADVIHAIDLTRS

RKTLVRASDTAGRLTSYFRDLIHKRKAHPQQDLISRFIMEEQLSKEEVLATCILLVIAGH

ETTVNLISNGVFTLLKHPEQLSALRENPSLIETAVEECLRYDSPTQLTARTASEDCEING

KTIKKGEQVYILLGAANRDPSIFDQSHKMDIQRKPNPHLAFGKNAHFCIGSSLARIEAQI

AILTLFERMPKLRLAAHRLEYRKLIGFRSLKELPVVIG

>CYP107K3 *Bacillus sp.* Pc3

MEKTMFHPHSPEFHENPFAVLSRFRAQDPIHKFELQRFGGTFPAWLITRYDDCMAFLKDG

RITRDVKRVMPKELIAKLNVSEDIDFVSDHMLAKDPPDHSRLRSLVHQGFTPRMIEQLRT

GIEQITEELLDEMETKADPDIMRDFAAPLPFIVISELLGIPKEDRAKFQVWTNAMVDTSE

SGQDATNQALKEFKQYMKTLIEGKRKHPGEDLTSKLIYAEEDGQKLSESELYSMLFLLVV

AGLETTVNLLGSGTLALLLHKDQMEKIKRQPENIQTAVEELLRYTSPVIMMANRWAIEDF

TYKDVSIKKGDMIFIGIGSANRDPEYFDDPDTLNIARTPNRHISFGFGIHFCLGAPLARM

EASIAFTALLKRFPNIELKGAPEDVTWRKNVFLRGLETLPVRF

>CYP109B7 *Bacillus sp*. Pc3

MSKLRSTRQAIQRTLMKGKNGLDVYNPFPWYEKMRRESPIHFDEETKVWSVFLYDDVKKV

ISDKETFSSLMTDVKSSIAKSMLNMDPPKHTQIRSAVNRAFTPRVLKEWEPRIKDITDHL

LKQAKNKGRIDIVKDLSYPLPVMVISELLGVPSERMDQFKKWSDILVSMPKDASPEAAEK

NQQERDQCEAELAAFFAEIIESKRKQPGQDIISILIKEEEEGEKLTAEDLIPFCNLLLVA

GNETTTNLISNAVYSILETPGLYEELRQDPSLIAQTVEETLRFRAPAPFVRRTVRHDTEL

RGRRLKSGEIVLCYVASANRDENKFEKAGVFDIHRQSNPHLSFGFGVHFCLGAPLARLEA

EVALKGIVKAFSHLEPIRIEPIRNSVMYGLESLEAEINENEGEEK

>CYP113L1 *Bacillus sp*. Pc3

MTSLTKIRQQQPYKWYQTMRETSPVHYNEKEDCWEIFTYDEVKRVISDYSHFSSDHKYLS

ADKQEKMIRHINKDSLLKMDPPEHTVFRKLVNQPFMPKSVESLAPRIAAIADDLLQAVRS

KDRMDIIEDYAFPLPIIVIAELLGFPPKDRDIFKSWVDQSQNVKDEKKMNEVQKQMIGYF

MQFILQRRKQPQNDLISHLISADLDGEPLSDKQLIGFCGLLIVAGHVTTENVIGNSFLSL

KEFPHILPRLLENKALLPDFIEEVIRLRPSIQRVTRYTAVESEIGGKTIPAGEKVYAWIG

SANRDEKKFENADQIDLGRKPNQHLSFGQGSHYCLGAPLARLEAKIALSHFFEQMPAWRF

TEDQEPNLVPSPVFHGVDRLLVEF

>CYP107H4 *Bacillus sp*. SDLI1

MTAGVSISHPSLQADSDFWNDPYPFYDNLRAIDPVYKGTVLKYPGWYVTGYKEAAAILKD

TRFKNRVPLPETSTKYQNLSHIQHDMLLFKNQSDHKRMRMVIGKEFTAKTAESLRPCIKE

TVHDLLDQVQNKKTADLVSEFAFPLASLIIAKILGVPKEERYQFRQWTADVIQAIDFTRS

RKTLVRASDTQGRMTSYFRDLIHKRETHPKEDLISRFIMEEQLSKDEVLATCVLLIIAGH

ETTVNLISNGVYTLLKHPEQLSALRENPTLIETAVEECLRYDSPTQLTARTASEDCEIYG

KIIKKGEQVYILLGAANRDPSIFDQPHKMDIERKPNPHLAFGKNAHFCVGSRLARIEAQI

AILALLERLPKLRLAAPKLEYRKLIGFRSLTELPVVVG

>CYP107K3 *Bacillus sp*. SDLI1

MEKIMFHPHSFEFHENPFAVLSTFREQDPIHKFELQRFGGTFPAWLITRYDDCMAFLKDS

RITRDVKRVMPKELIAKLNVSEDIDFVSEHMLAKDPPDHSRLRSLVHQGFTPRMIEQLRT

GIEWITEELLDEMETKTDPDIMRDFAAPLPFIVISELLGIPKEDRASFQVWTNAMVDTSE

SGREATNQALKEFKQYMKTLIEEKRIHPGEDLTSKLIYAEEDGQKLSESELYSMLFLLVV

AGLETTVNLLGSGTLALLLHKDQLEKIKRQPETIQTAVEELLRYTSPVIMMANRWAIEDF

TYKDVSIKKGDMIFIGIGSANRDPEYFDDPDTLNTARTPNRHISFGFGIHFCLGAPLARM

EASIAFTALVKRFPNIELKGAPEDLSWRKNVFLRGLEALPVRL

>CYP113L1 *Bacillus sp*. SDLI1

MTSLTKIRQQQPYKWYQTMRETSPVHYNEKEDCWEIFTYDEVKRVISDYSHFSSDHKYLS

ADKQEKMIRHINKDSLLKMDPPEHTVFRKLVNQPFMPKSVETLTPRIAAIADDLLQAVRS

KGRMDIIEDYAFPLPIIVIAELLGFPPKDRDIFKSWVDQSQNVKDEKKMNEVQKQMIGYF

MQFILQRRKQPENDLISHLISADLDGEPLSDKQLIGFCGLLIVAGHVTTENVIGNSFLSL

KEFPHILPRLLENKAFLPDFIEEVIRLRPSIQRVTRYTAIESEIGGKTIPAGEKVYAWIG

SANRDEKKFENADQIDLGRKPNQHLSFGQGSHYCLGAPLARLEAKIALSHFFEQMPAWRF

TEDQEPNLVPSPVFHGVDRLLVEF

>CYP109B3(AUL54_04475) *Bacillus sp*. SDLI1

MSKLRSPRQAIQRTLMKGKNGLDVYNPFPWYEKMRRESPIQFDEETKVWSVFLYDDVKRV

ISDKETFSSLMPDVKSSIAKSMLNMDPPKHTQIRSAVNRAFTPRVLKEWEPRIEDITENL

LKQAKNKGRIDIVKDLSYPLPVTVISELLGVPSENMEQFKKWSDILVSMPKDASPEAAEK

NQQERDQCEAELAAFFAEIIESKRKQPSRDIISILIKEEEEGEKLSAEDLIPFCNLLLVA

GNETTTNLISNAVYSILETPGLYEELRQDPSLIAQTVEETLRFRAPAPFVRRTVRHDTEL

CGRRLKAGDIVLCYIASANRDENKFEQADVFDIHRRSNPHLSFGFGVHFCLGAPLARLEA

EAALKGIVKSFPHLEPVRIEPIRKSVMYGLESLEAEINENKGGEQR

>CYP102A38(AUL54_07110) *Bacillus sp*. SDLI1

MKETSPIPQPKTFGPLGNLPLLDKDKPTMSLLKLANEQGPIFQLHTPAGTIIVVSGHELV

KEVCDEERFDKSIEGALEKVRAFAGDGLFTSWTHEPNWRKAHHILMPTFSQRAMKDYHAM

MTDIAVQLIQKWARLNPDEAVDVPADMTRLTLDTIGLCGFNYRFNSYYRETPHPFINSMV

RALDEAMHQMQRLDVQDKLMIRTKRQFHHDIQAMFSLVDSIIAERKSDGGRDEKDLLARM

LNVEDPETGDKLDDENIRFQIITFLIAGHETTSGLLSFAIYFLLKHPRVLEKAYEEADRV

LTDPVPSYKQVLDLTYIRMILQESLRLWPTAPAFSLYAKEDTVIGGKYPITAKDRISVLI

PQLHRDKEAWGDNAEEFYPERFEDPDQVPHHAYKPFGNGQRACIGMQFALHEATLVLGMI

LQHFTFTDHTDYELDIKQTLTLKPGDFHIRVRPRNKEAVSAALPAAEKAGEGVKKEKQET

KGASIIGLDNRPLLILYGSDTGTAEGVARELADTAGMHGVRTETAPLNDRIGKLPKEGAL

LIITSSYNGKPPSNAGQFVQWLEEVKPGELKGVRYAVFGCGDHNWAATYQAVPRLIDEKL

AEKGAERFSSRGEGDVSGDFEGKLDEWKKRMWADAMKAFDLKLNENAEKERSTLGLQFVS

GLGGSPLAQTYEAVYASVAVNRELQAPESGRSTRHIEIILPKEAAYNEGDHLGVLPVNSK

EQVGRVLRRYNLNGNDQVLLTASGQSAAHLPLDRPVRLHDLLSSCVELQEAASRAQIREM

AAYTVCPPHKRELEDLLGEGVYQEQILTLRVSMLDLLEKYEACELPFERFLELLRPLKPR

YYSISSSPRKHPGQASITVGVVRGPARSGLGEYRGVASNYLAERRPDDGIVMFVRTPETR

FRLPEDPEKPIIMVGPGTGVAPFRGFLQARAALKEEGKELGEAHLYFGCRNDHDFIYRDE

LEGYEKDGVVTLHTAFSRKEGVPKTYVQHVMTEDAEALISILDRGGHLYVCGDGSKMAPD

VEAALQKAYQSVHETDARQAQEWLLDLQTKGIYAKDVWAGI

>CYP102A49(AUL54_17680) *Bacillus sp*. SDLI1

MKQLNAIPQPKTYGPLKNLPHLEKEKLSQSLWKIAEEYGPIFRFEFPSSVGVFVSGRELA

AEVCDEKRFDKNLSKALLKVREFGGDGLFTSWTNEKNWQKAHRILLPSFSQKAMKGYHSM

MLDIAMQLVQKWSRLNPNEEIDVAEDMTRLTLDTIGLCGFHYRFNSFYRDTQHPFITSML

RALQEAMRQSQRHSLQDKLMIKTRHQFQQDIEVMNELVDRIIAERRENPDENLSDLLSLM

LEAKDPVTGERLDDENIRYQIITFLIAGHETTSGLLSFAIYCLLKNKDKLEKAVEEAERV

LTGETPEYKQIQQLTYIRMVLNETLRLYPTAPAFSLYAKEDTVLGGKYPIEKGQPVTILT

PQLHRDKSAWGEDAELFRPERFSDPAAIPADAYKPFGNGQRACIGMQFALQEATMVLGLV

LKHFELIDHTDYELTIKEALTIKPGDFKIRVKNKDVSNQQPAQNHKAENSGHKEETEEIP

SHGTPLLILYGSNLGTAEGIAEELADIGRSKGFSTETGPLDDYAGKLPVKGAVVIVTASY

NGAPPDNAAGFVKWMETLEDQELKGVSYAVFGCGDRNWAATYQRIPRLIDKVLEEKGAAR

LTSLGEGDNADDFEYSQEAWEDSFWQDIIKAFHIEAAPKQQNKSQLSIEYVSEAAETPIA

KTYKAFEAEVITNKELHTESSKRSVRHIELRLPESETYQEGDHLGVLPQNSGKLISRVIR

RFGLDPNQPFKIKGRQLPHLPMDRAVNAPEILASYVELQEPATRAQLRELAAHAVCPPHQ

KELEHLYSDDAAYKENVLKKRMTMLDLLEEYPACELPFARFLELLPSLKARYYSISSSPK

AKSGELSITVGVVTAPAWSGRGEYRGVASNYLAGLQNGDHAACFIRSPQSGFSLPENTKT

PLIMVGAGTGIAPFRGFIQARAAEKMSGHSLGEAHLYFGCRHPEEDDLYKDEFDQAEKNG

LVTVHRAYSRLDQDCKVYVQDVLLREAAQIIALLGQGGHLYICGDGSKMAPAVENVLLQA

YEKVHNTDSKVSLNWLEQLQAEGRYAKDVWAGV

>CYP102A3 *Bacillus subtilis* subsp. natto BEST195

MLMKQASAIPQPKTYGPLKNLPHLEKEQLSQSLWRIADELGPIFRFDFPGVSSVFVSGHN

FVAEVCDESRFDKNLGKGLQKVREFGGDGLFTSWTHEPNWQKAHRILLPSFSQKAMKGYH

SMMLDIATQLIQKWSRLNPNEEIDVADDMTRLTLDTIGLCGFNYRFNSFYRDSQHPFITS

MLRALKEAMNQSKRLGLQDKMMVKTKLQFQKDIEVMNSLVDRMIAERKANPDDNIKDLLS

LMLYAKDPVTGETLDDENIRYQIITFLIAGHETTSGLLSFAIYCLLTHPEKLKKA

>CYP102A36 *Bacillus subtilis* subsp. *natto* BEST195

MKETSPIPQPKTFGPLGNLPLIDKDKPTLSLIKLAEEQGPIFQIHTPAGTTIVVSGHELV

KEVCDEERFDKSIEGALEKVRAFSGDGLFTSWTHEPNWRKAHNILMPTFSQQAMKDYHEK

MVDIAVQLIQKWARLNPNEVVDVPGDMTRLTLDTIGLCGFNYRFNSYYRETPHPFINSMV

RALDEAMHQMQRLDVQDKLMVRTKRQFRHDIQTMFSLVDSIIAERRANGDQDEKDLLARM

LNVEDPETGEKLDDENIRFQIITFLIAGHETTSGLLSFATYFLLKHPDKLKKAYEEVDRV

LTDAAPTYKQVLELTYIRMILNESLRLWPTAPAFSLYPKEDTVIGGKFPITTKDRISVLI

PQLHRDRDAWGKDAEEFRPERFEHQDQVPHHAYKPFGNGQRACIGMQFALHEATLVLGMI

LKYFTLIDHENYELDIKQTLTLKPGDFHIRVQSRHQEAIHADVQTAEKAAPDEQKEKTEA

KGASVIGLNNRPLLVLYGSDTGTAEGVARELADTASLHDVRTETAPLNDRIGKLPKEGAV

VIVTSSYNGKPPSNAGQFVQWLQEIKPGELEGVHYAVFGCGDHNWASTYQYVPRFIDEQL

AEKGATRFSGRGEGDVSGDFEGQLDEWKKSMWADAIKAFGLELNENADKERSTLSLQFVR

GLGESPLARSYEASHASIAENRELQSADSDRSTRHIEIALPPDVEYQEGDHLGVLPKNSQ

TNVSRILHRFGLKGTDQVTLSASGRSAGHLPLGRPVSLYDLLSYSVEVQEAATRAQIREL

AAFTVCPPHRRELEELSAEGVYQEQILKKRISMLDLLEKYEACDMPFERFLELLRPLKPR

YYSISSSPRVNPRLASITVGVVRGPAWSGRGEYRGVASNDLAERQAGDDVVMFIRTPESR

FQLPEDPETPIIMVGPGTGVAPFRGFLQAREVLKREGKTLGEAHLYFGCRNDRDFIYRDE

LEQFEKDGIVTVHTAFSRKEGMPKTYVQHVMADHAETLISILDRGGRLYVCGDGSKMAPD

VEAALQKAYQSVHGTGEQEAQNWLRHLQDTGMYAKDVWAGI

>CYP102A48 *Bacillus subtilis* subsp. *natto* BEST195

MLTDDTPEYKQIQQLKYTRMVLNETLRLYPTAPAFSLYAKEDTVLGGEYPISKGQPVTVL

IPKLHRDQNAWGPDAEDFRPERFEDPSSIPHHAYKPFGNGQRACIGMQFALQEATMVLGL

VLKHFELINHTGYELKIKEALTIKPDDFKITVKPRKTAAINVQRKEQAEIKAETKPKETK

PKHGTPLLVLYGSNLGTAEGIADELASQGRQMGFTAETAPLDDYIGKLPEEGAVVIVTAS

YNGSPPDNAAGFVEWLKELEEGQLKGVSYAVFGCGNRSWASTYQRIPRLIDDMMKAKGAS

RLTEIGEGDAADDFESHRESWENRFWKETMDAFDINEIAQKEDRPSLSIAFLSEATETPV

AKAYGAFEGVVLENRELQTADSTRSTCHIELEIPAGKTYKEGDHIGILPKNSRELVQRVL

SRFGLQSNHVIKVSGSAHMAHLPMDRPIKVADLLSSYVELQEPASRLQLRELASYTVCPP

HQKELEQLVSDDGIYKEQVLAKRLTMLDFLEDYPACEMPFERFLALLPSLKPRYYSISSS

PKVHANIVSMTVGVVKASAWSGRGEYRGVASNYLAELNTGDAAACFIRTPQSGFQMPDEP

ETPMIMVGPGTGIAPFRGFIQARSVLKKEGSTLGEALLYFGCRRPDHDDLYREELDQAEQ

EGLVTIRRCYSRVKNEPKEYVQHLLKQDTQKLMTLIEKGAHIYVCGDGSQMAPDVEKTLR

LAYEAEKGASQEESAEWLQKLQDQRRYVKDVWTGM

>CYP107H1 *Bacillus subtilis* subsp. *natto* BEST195

MMSIAFIFEQKGENHVTIASSTASSEFLKNPYSFYDTLRAVHPIYKGSFLKYPGWYVTGY

EETAAILKDARFKVRTPLPESSTKYQDLSHVQNQMMLFQNQPDHRRLRTLASGAFTPRAT

ESYQPYIIETVHQLLDQVQGEKKMEVISDFAFPLASFVIANIIGVPEEDREQLKEWAASL

IQTIDFTRSRKALTEGNHMAVQAMAYFKELIQKRKRHPQQDMISMLLKGKENDKLTEEEA

ASTCILLAIAGHETTVNLISNSVLCLLQHPEQLLKLRENPDLIGTAVEECLRYESPTQMT

ARVASEDIDISGVTIRQGEQVYLLLGAANRDPSIFTNPDVFDITRSPNPHLSFGHGHHVC

LGSSLARLEAQIAINTFLQRMPSLKLADFEWRYRPLFGFRALEELPVTFE

>CYP107J1 *Bacillus subtilis* subsp. *natto* BEST195

MSSKEKNSVTILTESQLSSRAFKDEAYEFYKELRKSQPLYPLSLGALGKGWLISRYEDAI

HLLKNEKLKKNYENVFTAKEKRPALLKNEESLTKHMLNSDPPDHNRLRTLVQKAFTHRMI

FQLEDKIQHIADSLLDKVQPNKFMNLVDDYAFPLPIIVISEMLGIPLEDRQKFRVWSQAI

IDFSDAPERLQENDHLLGEFVEYLESLVRKKRSEPSGDLISALIQAESEGTQLSTEELYS

MIMLLIVIGHETTVNLITNMTYALMCHGDQLEKLRQQPDLMNSAIEEALWFHSPVELTTI

RWTAEPFMLHGQEIKRKDVIIISLASANRDEKIFPNADIFDIERKNNRHIAFGHGNHFCL

GAQLARLEAKIAISTLLRRCPNIQLKGEKEQIKWKGNFLMRALEELPLSF

>CYP152A1 *Bacillus subtilis* subsp. *natto* BEST195

MNEQIPHDKSLDNSLTLLKEGYLFIKNRTERYNSDLFQARLLGKNFICMTGAEAAKVFYD

TDRFQRQNALPKRVQKSLFGVNAIQGMDGSAHIHRKMLFLSLMTPPHQKRLAELMTEEWR

AAVTRWEKADEVVLFEEAKEILCRVACYWAGVPLKETEVKERAADFIDMVDAFGAVGPRH

WKGRRARPRAEEWIEVMIEDARAGLLKTTPGTALHEMAFHTQEDGSQLDSRMAAIELINV

LRPIVAISYFLVFSALALHEHPKYKEWLRSGNSRKREMFVQEVRRYYPFGPFLGALVKKD

FVWNNCEFKKGTSVLLDVYGTNHDPRLWDHPDEFRPERFAEREENPFDMIPQGGGHVEKG

HRCPGEGITIEVMKASLDFLVHQIEYDVPEQSLHYSLARMPSLPESGFVMSGIRRKS

>CYP102A36 *Bacillus subtilis* subsp. *subtilis* AG1839

MKETSPIPQPKTFGPLGNLPLIDKDKPTLSLIKLAEEQGPIFQIHTPAGTTIVVSGHELV

KEVCDEERFDKSIEGALEKVRAFSGDGLFTSWTHEPNWRKAHNILMPTFSQRAMKDYHEK

MVDIAVQLIQKWARLNPNEAVDVPGDMTRLTLDTIGLCGFNYRFNSYYRETPHPFINSMV

RALDEAMHQMQRLDVQDKLMVRTKRQFRYDIQTMFSLVDSIIAERRANGDQDEKDLLARM

LNVEDPETGEKLDDENIRFQIITFLIAGHETTSGLLSFATYFLLKHPDKLKKAYEEVDRV

LTDAAPTYKQVLELTYIRMILNESLRLWPTAPAFSLYPKEDTVIGGKFPITTNDRISVLI

PQLHRDRDAWGKDAEEFRPERFEHQDQVPHHAYKPFGNGQRACIGMQFALHEATLVLGMI

LKYFTLIDHENYELDIKQTLTLKPGDFHISVQSRHQEAIHADVQAAEKAAPDEQKEKTEA

KGASVIGLNNRPLLVLYGSDTGTAEGVARELADTASLHGVRTKTAPLNDRIGKLPKEGAV

VIVTSSYNGKPPSNAGQFVQWLQEIKPGELEGVHYAVFGCGDHNWASTYQYVPRFIDEQL

AEKGATRFSARGEGDVSGDFEGQLDEWKKSMWADAIKAFGLELNENADKERSTLSLQFVR

GLGESPLARSYEASHASIAENRELQSADSDRSTRHIEIALPPDVEYQEGDHLGVLPKNSQ

TNVSRILHRFGLKGTDQVTLSASGRSAGHLPLGRPVSLHDLLSYSVEVQEAATRAQIREL

ASFTVCPPHRRELEELSAEGVYQEQILKKRISMLDLLEKYEACDMPFERFLELLRPLKPR

YYSISSSPRVNPRQASITVGVVRGPAWSGRGEYRGVASNDLAERQAGDDVVMFIRTPESR

FQLPKDPETPIIMVGPGTGVAPFRGFLQARDVLKREGKTLGEAHLYFGCRNDRDFIYRDE

LERFEKDGIVTVHTAFSRKEGMPKTYVQHLMADQADTLISILDRGGRLYVCGDGSKMAPD

VEAALQKAYQAVHGTGEQEAQNWLRHLQDTGMYAKDVWAGI

>CYP102A48 *Bacillus subtilis* subsp. *subtilis* AG1839

MKQASAIPQPKTYGPLKNLPHLEKEQLSQSLWRIADELGPIFRFDFPGVSSVFVSGHNLV

AEVCDEKRFDKNLGKGLQKVREFGGDGLFTSWTHEPNWQKAHRILLPSFSQKAMKGYHSM

MLDIATQLIQKWSRLNPNEEIDVADDMTRLTLDTIGLCGFNYRFNSFYRDSQHPFITSML

RALKEAMNQSKRLGLQDKMMVKTKLQFQKDIEVMNSLVDRMIAERKANPDENIKDLLSLM

LYAKDPVTGETLDDENIRYQIITFLIAGHETTSGLLSFAIYCLLTHPEKLKKAQEEADRV

LTDDTPEYKQIQQLKYIRMVLNETLRLYPTAPAFSLYAKEDTVLGGEYPISKGQPVTVLI

PKLHRDQNAWGPDAEDFRPERFEDPSSIPHHAYKPFGNGQRACIGMQFALQEATMVLGLV

LKHFELINHTGYELKIKEALTIKPDDFKITVKPRKTAAINVQRKEQADIKAETKPKETKP

KHGTPLLVLFGSNLGTAEGIAGELAAQGRQMGFTAETAPLDDYIGKLPEEGAVVIVTASY

NGAPPDNAAGFVEWLKELEEGQLKGVSYAVFGCGNRSWASTYQRIPRLIDDMMKAKGASR

LTAIGEGDAADDFESHRESWENRFWKETMDAFDINEIAQKEDRPSLSITFLSEATETPVA

KAYGAFEGIVLENRELQTAASTRSTRHIELEIPAGKTYKEGDHIGILPKNSRELVQRVLS

RFGLQSNHVIKVSGSAHMAHLPMDRPIKVVDLLSSYVELQEPASRLQLRELASYTVCPPH

QKELEQLVSDDGIYKEQVLAKRLTMLDFLEDYPACEMPFERFLALLPSLKPRYYSISSSP

KVHANIVSMTVGVVKASAWSGRGEYRGVASNYLAELNTGDAAACFIRTPQSGFQMPNDPE

TPMIMVGPGTGIAPFRGFIQARSVLKKEGSTLGEALLYFGCRRPDHDDLYREELDQAEQD

GLVTIRRCYSRVENEPKGYVQHLLKQDTQKLMTLIEKGAHIYVCGDGSQMAPDVERTLRL

AYEAEKAASQEESAVWLQKLQDQRRYVKDVWTGM

>CYP107H1 *Bacillus subtilis* subsp. *subtilis* AG1839

MTIASSTASSEFLKNPYSFYDTLRAVHPIYKGSFLKYPGWYVTGYEETAAILKDARFKVR

TPLPESSTKYQDLSHVQNQMMLFQNQPDHRRLRTLASGAFTPRTTESYQPYIIETVHHLL

DQVQGKKKMEVISDFAFPLASFVIANIIGVPEEDREQLKEWAASLIQTIDFTRSRKALTE

GNIMAVQAMAYFKELIQKRKRHPQQDMISMLLKGREKDKLTEEEAASTCILLAIAGHETT

VNLISNSVLCLLQHPEQLLKLRENPDLIGTAVEECLRYESPTQMTARVASEDIDICGVTI

RQGEQVYLLLGAANRDPSIFTNPDVFDITRSPNPHLSFGHGHHVCLGSSLARLEAQIAIN

TLLQRMPSLNLADFEWRYRPLFGFRALEELPVTFE

>CYP107J1 *Bacillus subtilis* subsp. *subtilis* AG1839

MSSKEKKSVTILTESQLSSRAFKDEAYEFYKELRKSQALYPLSLGALGKGWLISRYDDAI

HLLKNEKLKKNYENVFTAKEKRPALLKNEETLTKHMLNSDPPDHNRLRTLVQKAFTHRMI

LQLEDKIQHIADSLLDKVQPNKFMNLVDDYAFPLPIIVISEMLGIPLEDRQKFRVWSQAI

IDFSDAPERLQENDHLLGEFVEYLESLVRKKRREPAGDLISALIQAESEGTQLSTEELYS

MIMLLIVAGHETTVNLITNMTYALMCHHDQLEKLRQQPDLMNSAIEEALRFHSPVELTTI

RWTAEPFILHGQEIKRKDVIIISLASANRDEKIFPNADIFDIERKNNRHIAFGHGNHFCL

GAQLARLEAKIAISTLLRRCPNIQLKGEKKQMKWKGNFLMRALEELPISF

>CYP107K1 *Bacillus subtilis* subsp. *subtilis* AG1839

MEKLMFHPHGKEFHHNPFSVLGRFREEEPIHRFELKRFGATYPAWLITRYDDCMAFLKDN

RITRDVKNVMNQEQIKMLNVSEDIDFVSDHMLAKDTPDHTRLRSLVHQAFTPRTIENLRG

SIEQIAEQLLDEMEKENKADIMKSFASPLPFIVISELMGIPKEDRSQFQIWTNAMVDTSE

GNRELTNQALREFKDYIAKLIHDRRIKPKDDLISKLVHAEENGSKLSEKELYSMLFLLVV

AGLETTVNLLGSGTLALLQHKKECEKLKQQPEMIATAVEELLRYTSPVVMMANRWAIEDF

TYKGHSIKRGDMIFIGIGSANRDPNFFENPEILNINRSPNRHISFGFGIHFCLGAPLARL

EGHIAFKALLKRFPDIELAVAPDDIQWRKNVFLRGLESLPVSLSK

>CYP152A1 *Bacillus subtilis* subsp. *subtilis* AG1839

MNEQIPHDKSLDNSLTLLKEGYLFIKNRTERYNSDLFQARLLGKNFICMTGAEAAKVFYD

TDRFQRQNALPKRVQKSLFGVNAIQGMDGSAHIHRKMLFLSLMTPPHQKRLAELMTEEWK

AAVTRWEKADEVVLFEEAKEILCRVACYWAGVPLKETEVKERADDFIDMVDAFGAVGPRH

WKGRRARPRAEEWIEVMIEDARAGLLKTTSGTALHEMAFHTQEDGSQLDSRMAAIELINV

LRPIVAISYFLVFSALALHEHPKYKEWLRSGNSREREMFVQEVRRYYPFGPFLGALVKKD

FVWNNCEFKKGTSVLLDLYGTNHDPRLWDHPDEFRPERFAEREENLFDMIPQGGGHAEKG

HRCPGEGITIEVMKASLDFLVHQIEYDVPEQSLHYSLARMPSLPESGFVMSGIRRKS

>CYP102A38 *Bacillus velezensis*

MLERVLFMKETGPIPQPKTFGPLGNLPLLDKDKPTMSLIKLANEQGPIFQLHTPAGAIIV

VSGHELVKEVCDEERFDKSIEGALEKVRAFSGDGLFTSWTHEPNWRKAHNILMPTFSQRA

MKDYHSMMTDIAVQLIQKWARLNPDEAVDVPADMTRLTLDTIGLCGFNYRFNSYYRETPH

PFINSMVRALDEAMHQMQRLDVQDKLMIRTKRQFHHDIQAMFSLVDSIIAERRSGGRDEK

DLLARMLNVEDPETGEKLDDKNIRFQIITFLIAGHETTSGLLSFAIYFLLKHPRVLDKAY

EEADRVLTDPVPSYKQVLDLTYIRMILQESLRLWPTAPAFSLYAKEDTVIGGKYPITPKD

RISVLIPQLHRDKDAWGDNAEEFYPERFEHPDQVPQHAYKPFGNGQRACIGMQFALHEAT

LVLGMILQHFTFIDHTDYELDIKQTLTIKPGDFHIRVRPRNKGAVAAALPAAEKAAGDVE

KEKRETKGASIIGLDNRPLLILYGSDTGTAEGVARELADTAGMHGVRTETAPLNDRIGKL

PKEGALLIITSSYNGKPPSNAGQFVQWLEEVKPGELEGVRYAVFGCGDHNWAATYQAVPR

LIDEKLAEKGAERFSSRGEGDVSGDFEGKLDEWKKSMWTDAMKAFGLKLNENAEKERSSL

GLQFVSGLGGSPLAKTYEAVYASVAENRELQAPESGRSTRHIEITLPKEAAYHEGDHLGV

LPVNSKEQVSRVLRRFNLNGNDQVLLTASGQSAAHLPLDRPVRLHDLLSSCVELQEAASR

AQIREMAAYTVCPPHKRELEDFLEEGVYQEQILTLRVSMLDLLEKYEACELPFERFLELL

RPLKPRYYSISSSPRKHPGQASITVGVVCGPARSGLGEYRGVASNYLADRGPEDGIVMFV

RTPETRFRLPEDPEKPIIMVGPGTGVAPFRGFLQARAALKKEGKELGEAHLYFGCRNDHD

FIYRDELEAYEKDGIVTLHTAFSRKEGVPKTYVQHLMAKDAGALISILGRGGHLYVCGDG

>CYP102A49 *Bacillus velezensis*

MKQLSAIPQPKTYGPLKNLPHLEKEKLSQSLWKIAEEYGPIFRFEFPSSVGVFVSGRELA

AEVCDEKRFDKNLSKALLKVREFGGDGLFTSWTHEKNWQKAHRILLPSFSQKAMKGYHSM

MLDIAMQLVQKWSRLNPNEEIDVAEDMTRLTLDTIGLCGFHYRFNSFYRDTQHPFITSML

RALQEAMRQSQRHSLQDKLMIKTRHQFQQDIEEMNSLVDRIIAERRENPDENLSDLLALM

LEAKDPVTGERLDDENIRYQIITFLIAGHETTSGLLSFAIYCLLKNKDKLKKAVQEAERV

LTGETPEYKQIQQLTYIRMVLNETLRLYPTAPAFSLYAKEDTVLGGKYPIEKGQPVTILT

PQLHRDKSAWGEDAESFRPERFSDPAAIPADAYKPFGNGQRACIGMQFALQEATMVLGLV

LKHFELIDHTDYELTIKEALTIKPGDFKIRVKNKDVSNHQPVQNHKAEGSGHKEETKEIP

SHGTPLLILYGSNLGTAEGIAEELADIGRSKGFSTETGPLDDYAGKLPVKGAVVIVTASY

NGAPPDNAAGFVKWMETLEDQELKGVSYAVFGCGDRNWAATYQRIPRLIDKVLEEKGAKR

LTSIGEGDNADDFEYSQEAWEDSFWKDIMKTFHIEAAPKQTNKSQLSIEYVSEAAETPIA

KTYKAFEAEVITNKELHTESSKRSVRHIELRLPETETYQEGDHLGVLPQNSGELISRVIR

RFGLDPNQHFKIKGRQLPHLPMDRPVNAPELLASYVELQEPATRAQLRELAAHTVCPPHQ

KELEHLYSDDAAYKENVLKKRMTMLDLLEDYPACELPFERFLELLPSLKARYYSISSSPK

AASGELSITVGVVTAPAWSGRGEYRGVASNYLAGLQNGDPAVCFIRSPQSGFALPENPKT

PLIMVGAGTGIAPFRGFIQARAAEKMSGNSLGEAHLYFGCRHPEEDDLYKDEFDDAEKNG

LVTVHRAYSRLDQDCKVYVQDVLLREAAQIIALLDQGGHLYICGDGSKMAPAVENVLLQA

YEKVHNTDSKVSLNWLEQLQAEGRYAKDVWAGM

>CYP107H4 *Bacillus velezensis*

MTAGLSIAHPSLHADSDFWNDPYPFYDKLRSIDPVYKGTVLKYPGWYVTGYKEAAAILKD

TRFKNRIPLPEASTKYQNLSHIQHDMLLFKNQSDHKRMRMLIGKEFTAKTAESLRPCIKE

TVHDLLDQVQIKKNADLVSEFAFPLASLIIAEILGVPKEERYQFRQWTADVIQAIDLTRS

RKTLVRASDTAGRLTSYFRDLIHKRKAHPQQDLISRFIMEEQLSKEEVLATCILLVIAGH

ETTVNLMCNGVFTLLKHPEQLSALRENPSLIETAVEECLRYDSPAQLTARTASEDCEING

KTIKKGEQVYILLGAANRDPSIFDQPHKMDIQRKPNPHLAFGKNAHFCIGSSLARIEAQI

AILTLFERMPKLRLAAHRLEYRKLIGFRSLKELPVVIG

>CYP107K3 *Bacillus velezensis*

MEKTMFHPHSPEFHENPFAVLSRFREQDPIHKFELQRFGGTFPAWLITRYDDCMAFLKDG

RITRDVKRVMPKELIAKLNVSEDIDFVSEHMLAKDPPDHSRLRSLVHQGFTPRMIEQLRT

GIEQITEELLDEMETKADPDIMRDFAAPLPFIVISELLGIPKEDRAKFQVWTNAMVDTSE

SGQDATNQALKEFKQYMKTLIEEKRKHPAEDLTSKLIYAEEDGQKLSESELYSMLFLLVV

AGLETTVNLLGSGTLALLLHKDQMEKIKRQPEDIQTAVEELLRYTSPVIMMANRWAIEDF

TYKDVSIKKGDMIFIGIGSANRDPEYFDDPDTLNIARTPNRHISFGFGIHFCLGAPLARM

EASIAFTALLKRFPNIELKGAPEDVTWRKNVFLRGLETLPVRF

>CYP109B7 *Bacillus velezensis*

MSKLRSTRQAIQRTLMKGKNGLDVYNPFPWYEKMRRESPIHFDEETKVWSVFLYDDVKKV

ISDKETFSSLMTDVKSSIAKSMLNMDPPKHTQIRSAVNRAFTPRVLKEWEPRIKDITDHL

LKQAKNKGRIDIVKDLSYPLPVMVISELLGVPSERMDQFKKWSDILVSMPKDASPEAAEK

NQQERDQCEAELAAFFAEIIESKRKKPGQDIISILIKEEEEGEKLTAEDLIPFCNLLLVA

GNETTTNLISNAVYSILETPGLYEELRQDPSLIAQTVEETLRFRAPAPFVRRTVRHDTEL

RGRRLKSGEIVLCYVASANRDENKFEKAGVFDIHRQSNPHLSFGFGVHFCLGAPLARLEA

EAALKGIVKTFSHLEPVGIEPIRNSVMYGLESLEAEINESEGEEKR

>CYP113L1 *Bacillus velezensis*

MTSLTKIRQQQPYKWYQTMRETSPVHYNEKEDCWEIFTYDEVKRVISDYSHFSSDHKYLS

ADKQEKMIRHINKDSLLKMDPPEHTVFRKLVNQPFMPKSVESLAPRIAAIADDLLQAVRS

KGRMDIIEDYAFPLPIIVIAELLGFPPKDRDIFKSWVDQSQNVKDEKKMNEVQKQMIGYF

MQFILQRRKQPQNDLISHLISADLDGEPLSDKQLIGFCGLLIVAGHVTTENVIGNSFLSL

KEFPHILPRLLENKALLPDFIEEVIRLRPSIQRVTRYTAVESEIGGKTIPAGEKVYAWIG

SANRDEKKFENADQIDLGRKPNQHLSFGQGSHYCLGAPLARLEAKIALSHFFEQMPAWRF

TEDQEPNLVPSPVFHGVDRLLVEF

>CYP102A38 *Bacillus velezensis* CAU B946

MKETGPIPQPKTFGPLGNLPLLDKDKPTMSLIKLANEQGPIFQLHTPAGAIIVVSGHELV

KEVCDEERFDKSIEGALEKVRAFAGDGLFTSWTHEPNWRKAHNILMPTFSQRAMKDYHSM

MTDIAVQLIQKWARLNPDEAVDVPADMTRLTLDTIGLCGFNYRFNSYYRETPHPFINSMV

RALDEAMHQMQRLDVQDKLMIRTKRQFHHDIQAMFSLVDSIIAERRSGGRDEKDLLARML

NVEDPETGEKLDDENIRFQIITFLIAGHETTSGLLSFAIYFLLKHPRVLEKAYEEADRVL

TDPVPSYKQVLELSYIRMILQESLRLWPTAPAFSLYAKEDTVIGGKYPITPKDRISVLIP

QLHREKDAWGDNAEEFYPERFEHPDQVPHHAYKPFGNGQRACIGMQFALHEATLVLGMIL

QHFTFIDHTDYELDIKQTLTIKPGDFHIRVRPRNKGAVAAALPAAEKAAEDVEKEKRETK

GASIIGLDNRPLLILYGSDTGTAEGVARELADTAGMHGVRTETAPLNDRIGKLPKEGALL

IITSSYNGKPPSNAGQFVQWLEEVKPGELEGVRYAVFGCGDHNWAATYQAVPRLIDEKLA

EKGAERFSARGEGDVSGDFEGKLDEWKKSMWTDAMKAFGLKLNENAEKERSALGLQFVSG

LGGSPLAQTYEAVYASVAENRELQAPESGRSTRHIEITLPKEAAYHEGDHLGVLPVNSKK

QVSRVLRRFNLNGNDQVLLTASGQSAAHLPLDRPVRLHDLLSSCVELQEAASRAQIREMA

AYTVCPPHKRELEDFLEEGVYQEQILTSRVSMLDLLEKYEACELPFERFLELLRPLKPRY

YSISSSPRKHPGQASITVGVVRGPARSGLGEYRGVASNYLADRRPEDGIVMFVRTPETRF

RLPEDPEKPIIMVGPGTGVAPFRGFLQARAALKKEGKELGEAHLYFGCRNDHDFIYRDEL

EAYEKDGIVTLHTAFSRKEGVPKTYVQHLMAKDAGALISILGRGGRLYVCGDGSKMAPDV

EATLQKAYQSVRETDERQAQEWLLDLQTKGIYAKDVWAGI

>CYP102A49 *Bacillus velezensis* CAU B946

MKQLSAIPQPKTYGPLKNLPHLEKEKLSQSLWKIAEEYGPIFRFEFPSSVGVFVSGRELA

AEVCDEKRFDKNLSKALLKVREFGGDGLFTSWTHEKNWQKAHRILLPSFSQKAMKGYHSM

MLDIAMQLVQKWSRLNPNEEIDVAEDMTRLTLDTIGLCGFHYRFNSFYRDTQHPFITSML

RALQEAMRQSQRHSLQDKLMIKTRHQFQQDIEVMNELVDRIIAERRENPDENLSDLLALM

LEAKDPVTGERLDDENIRYQIITFLIAGHETTSGLLSFAIYCLLKNKDKLKKAVQEAERV

LTGETPEYKQIQQLTYIRMVLNETLRLYPTAPAFSLYAKEDTVLGGKYPIAKGQPVTILT

PQLHRDKSAWGEDAELFRPERFSDPAAIPADAYKPFGNGQRACIGMQFALQEATMVLGLV

LKHFELIDHTDYELTIKEALTIKPGDFKIRVKNKDVSNHQPVQNHKAEGGGHKEETKEIP

SHGTPLLILYGSNLGTAEGIAEELADIGRSKGFSTETGPLNDYAGKLPVKGAVVIVTASY

NGAPPDNAAGFVKWMETLEDQELKGVSYAVFGCGDRNWAATYQRIPRLIDKVLEEKGAKR

LTSIGEGDNADDFEYSQEAWEDSFWKDIMKAFHIEAAPKQANKSQLSIEYVSEAAETPIA

KTYKAFEAEVITNKELHTESSKRSVRHIELRLPKTETYREGDHLGVLPQNSGELISRVIR

RFGLDPNQHFKIKGRQLPHLPMDRPVNAPELLASYVELQEPATRAQLRELAAHTVCPPHQ

KELEHLYSDDAAYKENVLKKRMTMLDLLEDYPACELPFERFLELLPSLKARYYSISSSPK

ANKRELSITVGVVTAPAWSGRGEYKGVASNYLAGLQNGDSAVCFIRSPQSGFALPENTKT

PLIMIGAGTGIAPFRGFIQARAAEKMSGNSLGEAHLYFGCRHPEEDDLYKDEFDHAEKNG

LVTVHRAYSRLDQDCKVYVQDVLLREAAQIIALLDQGGHLYICGDGSKMAPAVENVLLQA

YEKVHNTDSKVSSNWLEQLQAEGRYAKDVWAGV

>CYP107H4 *Bacillus velezensis* CAU B946

MTAGLSTAHPSLHADSDFWNDPYPFYEKLRAIDPVYKGTVLKHPGWYVTGYKEAAAILKD

TRFKNRVPFPEASTKYQNLSHIQHDMLLFKNQSDHKRMRMLIGKEFTAKTAESLRPCIKE

TVHDLLDQIQNKKNADLVSEFAFPLASLIIAEILGVPKEERYQFRQWTADVIQAIDLTRS

RKMLVRASDTAGRLTAYFRDLIHERKAHPQEDLISRFIMGEQLSKDEVLATCILLVIAGH

ETTVNLMSNGVFTLLKHPEQLSALRENPSLIETAVEECLRYDSPAQLTARTASEDCEING

KTIKKGEQVYILLGAANRDPSIFDQPHKMDIQRKPNPHLAFGKNAHFCIGSSLARIEAQI

AILTLFERMPKLRLAAHRLEYRKLIGFRSLKELPVVLG

>CYP107K3 *Bacillus velezensis* CAU B946

MEKTMFHPHSPEFHEDPFAVLSRFREQDPIHKFELQRFGGTFPAWLITRYDDCMAFLKDG

RITRDVKRVMPKELIAKLNVSEDIDFVSEHMLAKDPPDHSRLRSLVHQGFTPRMIEQLRT

GIEQITEELLNEMETKADPDIMRDFAAPLPFIVISELLGIPKEDRAKFQVWTNAMVDTSE

SGQDATNQALKEFKQYMKTLIEEKRKHPAEDLTSKLIYAEEDGQKLSESELYSMLFLLVV

AGLETTVNLLGSGTLALLLHKDQMEKIKRQPENIQTAVEELLRYTSPVIMMANRWAIEDF

TYKDVSIKKGDMIFIGIGSANRDPEYFDDPDTLNIARTPNRHISFGFGIHFCLGAPLARM

EASIAFTSLLKRFPNIELKGAHEDVTWRKNVFLRGLETLPVRF

>CYP109B7 *Bacillus velezensis* CAU B946

MSKLRSTRQAIQRTLMKGKNGLDVYNPFPWYEKMRRESPIYFDEETKVWSVFLYDDVKKV

ISDKETFSSRMTDVKSSIAKSMLNMDPPKHTQIRSAVNRAFTPRVLKEWEPRIKDITDHL

LKQAKNKGRIDIVKDLSYPLPVMVISELLGVPSERMDQFKKWSDILVSMPKDASPEAAEK

NQQERDQCEAELAAFFAGIIESKRKQPGQDIISILIKEEEEGEKLTPEDLIPFCNLLLVA

GNETTTNLISNAVYSILETPGLYEELRQDPSLIAQTVEETLRFRAPAPFVRRTVRHDTEL

RGRRLKSGEIVLCYVASANRDENKFEKAGVFDIHRQSNSHLSFGFGVHFCLGAPLARLEA

EAALKGIVKAFSHLEPVRIEPIRNSVMYGLESLEAEINENEGEEKR

>CYP113L1 *Bacillus velezensis* CAU B946

MTSLTKIRQQQPYKWYQTMRETSPVHYNEKEDCWEIFTYDEVKRVISDYSHFSSDHKYLS

ADKQEKMIRHINKDSLLKMDPPEHTVFRKLVNQPFMPKSVESLAPRIAAIADDLLQAVRS

KGRMDIIEDYAFPLPIIVIAELLGFPPKDRDIFKSWVDQSQNVKDEKKMNEVQKQMIGYF

MQFILQRRKQPQNDLISHLISADLDGEPLSDKQLIGFCGLLIVAGHVTTENVIGNSFLSL

KEFPHILPRLLENKALLPDFIEEVIRLRPSIQRVTRYTAVESEIGGKTIPAGEKVYAWIG

SANRDEKKFENADQIDLGRKPNQHLSFGQGSHYCLGAPLARLEAKIALSHFFEQMPAWRF

TEDQEPNLVPSPVFHGVDRLLVEF

>CYP102A38 *Bacillus velezensis* SQR9

MKETGPIPQPKTFGPLGNLPLLDKDKPTMSLIKLANEQGPIFQLHTPAGAIIVVSGHELV

KEVCDEERFDKSIEGALEKVRAFSGDGLFTSWTHEPNWRKAHNILMPTFSQRAMKDYHSM

MTDIAVQLIQKWARLNPDEAVDVPADMTRLTLDTIGLCGFNYRFNSYYRETPHPFINSMV

RALDEAMHQMQRLDVQDKLMIRTKRQFHHDIQAMFSLVDSIIAERRSGGRDEKDLLARML

NVEDPETGEKLDDENIRFQIITFLIAGHETTSGLLSFAIYFLLKHPRVLEKAYEEADRVL

TDPVPSYKQVLDLTYIRMILQESLRLWPTAPAFSLYAKEDTVIGGKYPITPKDRISVLIP

QLHRDKDAWGDNAEEFYPERFEHPDQVPHHAYKPFGNGQRACIGMQFALHEATLVLGMIL

QHFTFIDHTDYELDIKQTLTIKPGDFHIRVRPRNKEAVAAALPAAEKAAEDVEKEKRETK

GASIIGLDNRPLLILYGSDTGTAEGVARELADTAGMHGVRTETAPLNDRIGKLPKEGALL

IITSSYNGKPPSNAAQFVQWLEEVKPGELEGVRYAVFGCGDHNWAATYQAVPRLIDEKLA

EKGAERFSSRGEGDVSGDFEGKLDEWKKSMWTDAMKVFGLKLNENAEKERSALGLQFVSG

LGGSPLAQTYEAVYASVAENRELQAPESGRSTRHIEITLPKEAAYHEGDHLGVLPVNSKE

QVRRVLRRFNLNGNDQVLLTASGQSAAHLPLDRPVRLHDLLSSCVELQEAASRAQIREMA

AYTVCPPHKRELEDFLEEGVYQEQILTSRVSMLDLLEKYEACELPFERFLELLRPLKPRY

YSISSSPRKNPGQASITVGVVRGPARSGLGEYRGVASNYLADRGPEDGIVMFVRTPETRF

RLPEDPEKPIIMVGPGTGVAPFRGFLQARAALKKEGKELGEAHLYFGCRNDHDFIYRDEL

EAYEKDGIVTLHTAFSRKEGVPKTYVQHLMAKDAGALISILGRGGHLYVCGDGSKMAPDV

EATLQKAYQSVHETDERQAQEWLLDLQTKGIYAKDVWAGI

>CYP102A49 *Bacillus velezensis* SQR9

MKQLSAIPQPKTYGPLKNLPHLEKEKLSQSLWKIAEEYGPIFRFEFPSSVGVFVSGRELA

AEVCDEKRFDKNLSKALLKVREFGGDGLFTSWTHEKNWQKAHRILLPSFSQKAMKGYHSM

MLDIAMQLVQKWSRLNPNEEIDVAEDMTRLTLDTIGLCGFHYRFNSFYRDTQHPFITSML

RALQEAMRQSQRHSLQDKLMIKTRHQFQQDIEEMNSLVDRIIAERRENPDENLSDLLALM

LEAKDPVTGERLDDENIRYQIITFLIAGHETTSGLLSFAIYCLLKNKDKLKKAVQEAERV

LTGETPEYKQIQQLTYIRMVLNETLRLYPTAPAFSLYAKEDTVLGGKYPIAKGQPVTILT

PQLHRDKSAWGEDAESFRPERFSDPAAIPADAYKPFGNGQRACIGMQFALQEATMVLGLV

LKHFELIDHTDYELTIKEALTIKPGDFKIRVKNKDVSNHQPVQNHKAEGSGHKEETKEIP

SHGTPLLILYGSNLGTAEGIAEELAGIGRSKGFSTETGPLDDYAGKLPVKGAVVIVTASY

NGAPPDNAAGFVKWMETLEDQELKGVSYAVFGCGDRNWAATYQRVPRLIDKVLEEKGAKR

LTSIGEGDNADDFEYSQEAWEDSFWKDIMKAFHIEAAPKQTNKSQLSIEYVSEAAETPIA

KTYKAFEAEVITNKELHTESSKRSVRHIELRLPETETYQEGDHLGVLPQNSGELISRVIR

RFGLDPNQHFKIKGRQLPHLPMDRPVNAPELLASYVELQEPATRVQLRELAAHTVCPPHQ

KELEHLYSDDTAYKENVLKKRMTMLDLLENYPACELPFERFLELLPSLKARYYSISSSPK

AASGELSITVGVVTAPAWSGRGEYRGVASNYLAGLQNGDSAVCFIRSPQSGFALPENPKT

PLIMVGAGTGIAPFRGFIQARAAEKMSGNILGAAHLYFGCRHPEEDDLYKDEFDHAEKNG

LVTVHRAYSRLDQDCKVYVQDVLLREAEQIIALLDQGGHLYICGDGSKMAPAVENVLLQA

YEKVHNTDSKVSLEWLEQLQAEGRYAKDVWAGM

>CYP107K3 *Bacillus velezensis* SQR9

MEKTMFHPHSPEFHENPFAVLSRFREQDPIHKFELQRFGGTFPAWLITRYDDCMAFLKDG

RITRDVKRVMPKELIAKLNVSEDIDFVSEHMLAKDPPDHSRLRSLVHQGFTPRMIEQLRT

GIEQITEELLDEMETKADPDIMRDFAAPLPFIVISELLGIPKEDRAKFQVWTNAMVDTSE

SGQDATNQALKEFKQYMKTLIEEKRKHPGEDLTSKLIYAEEDGQKLSESELYSMLFLLVV

AGLETTVNLLGSGTLALLLHKDQMEKIKRQPEDIQTAVEELLRYTSPVIMMANRWAIEDF

TYKDVSIKKGDMIFIGIGSANRDPEYFDDPDTLNIARTPNRHISFGFGIHFCLGAPLARM

EASIAFTALLKRFPNIELKGAPEDVTWRKNVFLRGLETLPVRF

>CYP109B7 *Bacillus velezensis* SQR9

MSKLRSTRQAIQRTLMKGKNGLDVYNPFPWYEKMRRESPIHFDEETKVWSVFLYDDVKKV

ISDKETFSSLMTDVKSSIAKSMLNMDPPKHTQIRSAVNRAFTPRVLKEWEPRIKDITDHL

LKQAKNKGRIDIVKDLSYPLPVMVISELLGVPSERMDQFKKWSDILVSMPKDASPEAAEK

NQQERDRCEAELAAFFAEIIKSKRKQPGQDIISILIKEEEEGEKLTAEDLIPFCNLLLVA

GNETTTNLISNAVYSILETPGLYEELRQDPSLIAQTVEETLRFRAPAPFVRRTVRHDTEL

RGRRLKSGEIVLCYVASANRDENKFEKAGVFDIHRQSNPHLSFGFGVHFCLGAPLARLEA

EAALKGIVKAFSHLEPVRIEPIRNSVMYGLESLEAEINENEGEEKR

>CYP107H4 *Bacillus velezensis* SQR9

MTADLSIAHPSLHADSDFWNDPYPFYDKLRSIDPVYKGTVLKYPGWYVTGYKEAAAILKD

IRFKNRIPLPEASTKYQNLSHIQHDMLLFKNQSDHKRMRMLIGKEFTAKTAESLRPCIKE

TVHDLLDQVQIKKTADLVSEFAFPSASLIIAEILGVPKEERYQFRQWTADVIQAIDLTRS

RKTLVRASDTAGRLTSYFKDLIHKRKAHPQQDLISRFIMEEQLSKEEVLATCILLVIAGH

ETTVNLISNGVFTLLKHPEQLSALRENPSLIETAVEECLRYDSPAQLTARTASEDCEING

KTIKKGEQVYILLGAANRDPSIFDQPHKMDIQRKPNPHLAFGKNAHFCIGSSLARIEAQI

AILTLFERMPKLRLAAHRLEYRKLIGFRSLKELPVVIG

>CYP113L1 *Bacillus velezensis* SQR9

MTSLTKIRQQQPYKWYQTMRETSPVHYNEKEDCWEIFNYDEVKRVISDYSHFSSDHKYLS

ADKQEKMIRHINKDSLLKMDPPEHTVFRKLVNQPFMPKSVESLTPRIAAIADDLLQAVRS

KGRMDIIEDYAFPLPIIVIAELLGFPPKDRDIFKSWVDQSQNVKDEKKMNEVQKQMIGYF

MQFILQRRKQPQNDLISHLISADLDGEPLSDKQLIGFCGLLIVAGHVTTENVIGNSFLSL

KEFPHILPRLLENKALLPDFIEEVIRLRPSIQRVTRYTAVESEIGGKTIPAGEKVYAWIG

SANRDEKKFENADQIDLGRKPNQHLSFGQGSHYCLGAPLARLEAKIALSHFFEQMPAWRF

TEDQEPNLVPSPVFHGVDRLLVEF

>CYP102A38 *Bacillus amyloliquefaciens* CC178

MKETGPIPQPKTFGPLGNLPLLDKDKPTMSLIKLANEQGPIFQLHTPAGAIIVVSGHELV

KEVCDEERFDKSIEGALEKVRAFSGDGLFTSWTHEPNWRKAHHILMPTFSQRAMKDYHSM

MTDIAVQLIQKWARLNPDEAVDVPADMTRLTLDTIGLCGFNYRFNSYYRETPHPFINSMV

RALDEAMHQMQRLDVQDKLMIRTKRQFHHDIQAMFSLVDSIIAERRSGGRDEKDLLARML

NVEDPETGEKLDDENIRFQIITFLIAGHETTSGLLSFAIYFLLKHPRVLEKAYEEADRVL

TDPVPSYKQVLDLTYIRMILQESLRLWPTAPAFSLYAKEDTVIGGKYPITPKDRISVLIP

QLHRDKDAWGDNAEEFYPERFEHPDRVPHHAYKPFGNGQRACIGMQFALHEATLVLGMIL

QHFTFIDHTDYELDIKQTLTIKPGDFHIRVRPRNKEDVAAALPAAEKAAEDVGKEKRETK

GASIIGLDNRPLLILYGSDTGTAEGVARELADTAGMHGVRTETAPLNDRIGKLPKEGALL

IITSSYNGKPPSNAGQFVQWLEEVKPGELEGVRYAVFGCGDHNWAATYQAVPRLIDEKLA

EKGAERFSSRGEGDVSGDFEGKLDEWKKSMWTDAMKAFGLKLNENAEKERSALGLQFVSG

LGGSPLAQTYEAVYASVAENRELQAPESGRSTRHIEITLPKEAAYHEGDHLGVLPVNSKE

QVSRVLRRFNLNGNDQVLLTASGQSAAHLPLDRPVRLHDLLSSCVELQEAASRAQIREMA

AYTVCPPHKRELEDFLEEGVYQEQILTSRVSMLDLLEKYEACELPFERFLELLRPLKPRY

YSISSSPRKHPGQASITVGVVRGPARSGLGEYRGVASNYLADRGPEDGIVMFVRTPETRF

RLPEDPEKPIIMVGPGTGVAPFRGFLQARAALKKEGKELGEAHLYFGCRNDHDFIYRDEL

EAYEKDGIVTLHTAFSRKEGVPKTYVQHLMAKDAGALISILGRGGHLYVCGDGSKMAPDV

EATLQKAYQSVRETDERQAQEWLLDLQTKGIYAKDVWAGI

>CYP102A49 *Bacillus amyloliquefaciens* CC178

MKQLSAIPQPKTYGPLKNLPHLEKEKLSQSLWKIAEEYGPIFRFEFPSSVGVFVSGRELA

AEVCDEKRFDKNLSKALLKVREFGGDGLFTSWTHEKNWQKAHRILLPSFSQKAMKGYHSM

MLDIAMQLVQKWSRLNPNEEIDVAEDMTRLTLDTIGLCGFHYRFNSFYRDTQHPFITSML

RALQEAMRQSQRHSLQDKLMIKTRHQFQQDIEEMNSLVDRIIAERRENPDENLSDLLALM

LEAKDPVTGERLDDENIRYQIITFLIAGHETTSGLLSFAIYCLLKNKDKLKKAVQEAERV

LTGETPEYKQIQQLTYIRMVLNETLRLYPTAPAFSLYAKEDTVLGGKYPIAKGQPVTILT

PQLHRDKSAWGEDAELFRPERFSDPAAIPADAYKPFGNGQRACIGMQFALQEATMVLGLV

LKHFELIDHTDYELTIKEALTIKPGDFKIRVKNKDVSNHQPVQNHKAEGSGHKEETKEIP

SHGTPLLILYGSNLGTAEGIAEELADIGRSKGFSTESGPLDDYAGKLPVKGAVVIVTASY

NGAPPDNAAGFVKWMETLEDQELKGVSYAVFGCGDRNWAATYQRIPRLIDKVLEEKGAKR

LTSIGEGDNADDFEYSQEAWEDSFWKDIMKAFHIEAAPKQTNKSQLSIEYVSEAAETPIA

KTYKAFEAEVITNKELHTESSKRSVRHIELRLPETETYQEGDHLGVLPQNSGELISRVIR

RFGLDPNQHFKIKGRQLPHLPMDRPVNAPELLASYVELQEPATRAQLRELAAHTVCPPHQ

KELEHLYSDDAAYKENVLKNRMTMLDLLEDYPACELPFERFLELLPSLKARYYSISSSPK

ATSGELSITVGVVTAPAWSGRGEYRGVASNYLAGLQKGDSAVCFIRSPQSGFALPENPKT

PLIMVGAGTGIAPFRGFIQARAAEKMSGNSLGEAHLYFGCRHPEQDDLYKDEFDHAEKNG

LVTVHRAYSRLDQDCKVYVQDVLLREAAQIIALLDQGGHLYICGDGSKMAPAVENVLLQA

YEKVHNTDSKVSLNWLEQLQAEGRYAKDVWAGM

>CYP107K3 *Bacillus amyloliquefaciens* CC178

MEKTMFHPHSPEFHENPFAVLSRFREQDPIHKFELQRFGGTFPAWLITRYDDCMAFLKDG

RITRDVKRVMPKELIAKLNVSEDIDFVSEHMLAKDPPDHSRLRSLVHQGFTPRMIEQLRT

GIEQITEELLDEMETKADPDIMRDFAAPLPFIVISELLGIPKEDRAKFQVWTNAMVDTSE

SGQDATNQALKEFKQYMKTLIEEKRKHPGEDLTSKLIYAEEDGQKLSESELYSMLFLLVV

AGLETTVNLLGSGTLALLLHKDQMEKIKRQPENIQTAVEELLRYTSPVIMMANRWAIEDF

TYKDVSIKKGDMIFIGIGSANRDPEYFDDPDTLNIARTPNRHISFGFGIHFCLGAPLARM

EASIAFTALLKRFPNIELKGAAEDVTWRKNVFLRGLETLPVRF

>CYP107H4 *Bacillus amyloliquefaciens* CC178

MTADLSIAHPSLHADSGFWNDPYPFYDKLRSIDPVYKGTVLKYPGWYVTGYKEAAAILKD

IRFKNRIPLPEASTKYQNLSHIQHDMLLFKNQSDHKRMRMLIGKEFTAKTAESLRPCIKE

TVHDLLDQVQIKKTADLVSEFAFPLASLIIAEILGVPKEERYQFRQWTADVIQAIDLTRS

RKTLVRASDTAGRLTSYFRDLIHKREAHPQQDLISRFIMEEQLSKEEVLATCILLVIAGH

ETTVNLISNGVFTLLKHPEQLSALRENPSLIETAVEECLRYDSPAQLTARTASEDCEING

KTIKKGEQVYILLGAANRDPSIFDQPHKMDIQRKPNPHLAFGRNAHFCIGSSLARIEAQI

AILTLFERMPKLRLAAHRLEYRKLIGFRSLKELPVVIG

>CYP113L1 *Bacillus amyloliquefaciens* CC178

MTSLTKIRQQQPYKWYQTMRETSPVHYNEKEDCWEIFTYDEVKRVISDYSHFSSDHKYLS

ADKQEKMIRHINKDSLLKMDPPEHTVFRKLVNQPFMPKSVESLEPRIAAIADDLLQAVRS

KGRMDIIEDYAFPLPIIVIAELLGFPPKDRDIFKSWVDQSQNVKDEKKMNEVQKQMIGYF

MQFILQRRKQPQNDLISHLISADLDGEPLSDKQLIGFCGLLIVAGHVTTENVIGNSFLSL

KEFPHILPRLLENKALLPDFIEEVIRLRPSIQRVTRYTAVESEIGGKTIPAGEKVYAWIG

SANRDEKKFENADQIDLGRKPNQHLSFGQGSHYCLGAPLARLEAKIALSHFFEQMSAWRF

TEDQEPNLVPSPVFHGVDRLLVEF

>CYP102A4 *Bacillus anthracis* Ames *Ancestor*

MDKKVSAIPQPKTYGPLGNLPLIDKDKPTLSFIKLAEEYGPIFRMQTLSDTIIVVSGHEL

VAEVCDETRFDKSIEGALAKVRAFAGDGLFTSETQEPNWQKAHNILMPTFSQRAMKDYHA

MMVDIAVQLVQKWARLNPNENVDVPEDMTRLTLDTIGLCGFNYRFNSFYRETPHPFITSM

TRALDEAMHQLQRLDIEDKLMWRTKRQFQHDIQSMFSLVDNIIAERKSSENQEENDLLSR

MLNVQDPETGEKLDDENIRFQIITFLIAGHETTSGLLSFAIYFLLKNPDKLKKAYEEVDR

VLTDSTPTYQQVMKLKYIRMILNESLRLWPTAPAFSLYAKEDTVIGGKYPIKKGEDRISV

LIPQLHRDKDAWGDNVEEFQPERFEELDKVPHHAYKPFGNGQRACIGMQFALHEATLVMG

MLLQHFEFIDYEEYQLDVKQTLTLKPGDFKIRIVPRNQTISHTTVLAPTEEKLKNHEIKQ

QVQKTPSIIGADNLSLLVLYGSDTGVAEGIARELADTASLEGVQTEVAALNDRIGSLPKE

GAVLIVTSSYNGKPPSNAGQFVQWLEELKGDELKGVQYAVFGCGDHNWASTYQRIPRYID

EQMAQKGATRFSTRGEADASGDFEEQLEQWKQRMWSDAMKVFGLELNKNMEKERSTLSLQ

FVSRLGGSPLARTYEAVYASILENRELQSSSSERSTRHIEISLPEGATYKEGDHLGVLPI

NSEKNVNRILKRFGLNGKDQVILSASGRSVNHIPLDSPVRLYDLLSYSVEVQEAATRAQI

REMVTFTACPPHKKELESLLEDGVYQEQILKKRISMLDLLEKYEACEIRFEPFLELLPAL

KPRYYSISSSPLVAQDRLSITVGVVNAPAWSGEGTYEGVASNYLAQRHNKDEIICFIRTP

QSNFQLPENPETPIIMVGPGTGIAPFRGFLQARRVQKQKGMNVGEAHLYFGCRHPEKDYL

YRTELENDERDGLISLHTAFSRLEGQAKTYVQHVIKEDRIHLISLLDNGAHLYICGDGSK

MAPDVEDTLCQAYQEIHEVSEQEARNWLDRLQEEGRYGKDVWAGI

>CYP106B1 *Bacillus anthracis Ames Ancestor*

MASPENVILVHEISKLKTKEELWNPYEWYQFMRDNHPVHYDDEQDVWNVFLYDDVNRVLS

DYSLFSSRRERRQFAIPPLETRININSTDPPEHRNVRSIVSKAFTPRSLEQWKPRIQSIA

NELVKDIENCSEVDIVEQFAAPLPVTVISDLLGVPTTDRKKIKAWSDILFMPYSKEKFND

LDAEKGIALNEFKAYLLPIVQEKRYHLTDDIISDLIRAEYEGERLTDEEIVTFSLGLLAA

GNETTTNLIINSFYCFLVDSPATYKEVREKPKLISKAVEEVLRYRFPVTLARRITEDTNI

FGPLMKKDQMVVAWVSAANLDEKKFSQASKFNIHRIGNEKHLTFGKGPHFCLGAPLARLE

AEIALTTFINAFEKIALSPSFNIEQCILENEQTLKFLPIRLKPQ

>CYP107J2 *Bacillus anthracis Ames Ancestor*

MAMKNKVGIRIEDGINLASAQFKEDAYEIYKESRKVQPVLFVNKTELGAEWLITRYEDAL

PLLKDNRLKKDPANVFSQDTLNVFLTVDNSDYLTTHMLNSDPPNHNRLRSLVQKVFTPKM

IAQLEGRIQDIADDLLNEVERKGSLNLVDDYSFPLPIIVISEMLGIPKEDQAKFRIWSHA

VIAYPETPEEIKETEKQLSEFITYLQYLVDMKRKEPKEDLVSALILAESEGHKLSARELY

SMIMLLIVAGHETTVNLITNTVLALLENPNQLQLLKENPKLIDAAIEEGLRYYSPVEVTT

SRWADEPFQIHDQTIEKGDMVVIALAAANRDETVFENPEVFDITRENNRHIAFGHGSHFC

LGAPLARLEAKIAITTLFERMPELQIKGNREDIKWQGNYLMRSLEELPLTF

>CYP102A5 *Bacillus bombysepticus*

MEKKVSAIPQPKTYGPLGNLPLIDKDKPTLSFIKIAEEYGPIFQIQTLSDTIIVVSGHEL

VAEVCDETRFDKSIEGALAKVRAFAGDGLFTSETHEPNWKKAHNILMPTFSQRAMKDYHA

MMVDIAVQLVQKWARLNPNENVDVPEDMTRLTLDTIGLCGFNYRFNSFYRETPHPFITSM

TRALDEAMHQLQRLDIEDKLIWRTKRQFQHDIQSMFSLVDNIIAERKSSGNQEENDLLSR

MLNVPDPETGEKLDDENIRFQIITFLIAGHETTSGLLSFAIYFLLKNPDKLKKAYEEVDR

VLTDSTPTYQQVMKLKYIRMILNESLRLWPTAPAFSLYAKEDTVIGGKYPIKKGEDRISV

LIPQLHRDKDAWGDNVEEFQPERFEELDKVPNHAYKPFGNGQRACIGMQFALHEATLVMG

MLLQHFELIDYQNYQLEVKQTLTLKPGDFKIRILPRKQTISHPTVLAPTEDKLKNDEIKQ

HVQKTPSIIGADNLSLLVLYGSDTGVAEGIARELADTASLEGVQTEVVALNDRIGSLPKE

GAVLIVTSSYNGKPPSNAGQFVQWLEELKPDELKGVQYAVFGCGDHNWASTYQRIPRYID

EQMAQKGATRFSKRGEADASGDFEEQLEQWKQSMWSDAMKAFGLELNKNMEKERSTLSLQ

FVSRLGGSPLARTYEAVYASILENRELQSSSSDRSTRHIEVSLPEGATYKEGDHLGVLPV

NSEKNINRILKRFGLNGKDQVILSASGRSINHIPLDSPVSLLDLLSYSVEVQEAATRAQI

REMVTFTACPPHKKELEALLEEGVYHEQILKKRISMLDLLEKYEACEIRFERFLELLPAL

KPRYYSISSSPLVAQNRLSITVGVVNAPAWSGEGTYEGVASNYLAQRHNKDEIICFIRTP

QSNFELPKDPETPIIMVGPGTGIAPFRGFLQARRVQKQKGINLGEAHLYFGCRHPEKDYL

YRTELENDERDGLISLHTAFSRLEGHPKTYVQHLIKQDRINLISLLDNGAHLYICGDGSK

MAPDVEDTLCQAYQEIHEVSEQEARNWLDRVQDEGRYGKDVWAGI

>CYP106B4 *Bacillus bombysepticus*

MASPENVILVHEISKLKTKEELWNPYEWYRLMRDNHPVHYDEEQDVWNVFLYDDVNRVLS

DYRLFSSRRERRQFSIPPLETRININSTDPPEHRNVRSIVSKAFTPRSLEQWKPRIQAIA

DELVQHIEKCSEVNIVEQFAAPLPVTVISDLLGVPTTDRKKIKEWSDILFMPYSKEKFND

LDAEKGIALNEFKAYLLPIVQEKRYHLTDDIISDLIRAEYEGERLTDEEIVTFSLGLLAA

GNETTTNLIINSFYCFLVDSPGIYEELRKEPNLISKAIEEVLRYRFPVTLARRITEDTNI

FGSLMKKDQMIVAWVSAANLDEKKFSQASQFNVHRTGNEKHLTFGKGPHFCLGAPLARLE

AEIALTTFINAFEKIELSPSFCLEKCILENEQTLKYLPIRLKAK

>CYP107J3 *Bacillus bombysepticus*

MSMKNKVGLSIEDGINLASAQFKEDAYEIYKESRKKQPILFVNQVEIGKEWLITRYEDAL

PLLKDNRLKKDWTNVFSQDIKNMYLSVDNSDHLTTHMLNSDPPNHSRLRSLVQKAFTPKM

IAQLDKRIERIADELISDIERKGTLNLVDDYSFPLPIIVISEMLGIPKEDQAKFRIWSHA

VIASPETPEEIKETEKQLSEFITYLQYLVDIKRKEPKEDLVSALILAESEGHKLSARELY

SMIMLLIVAGHETTVNLITNTVLALLENPNQLQLLKDNPKLIDSAIEEGLRYYSPVEVTT

ARWAAEPFQIHDRTIEKGDMVVIALASANRDETVFENPEVFDITRENNRHIAFGHGSHFC

LGAPLARLEAKIAITTLFNRMPELQIKGNREEIKWQGNYLMRSLEELPLTF

>CYP107DY3 *Bacillus bombysepticus*

MKKLTFNDLNSPETMRNPIMFYKNLMEQQERFFRIDDFYGMGGAWVAFHYDDVVAILKDS

RFIKDLRKFTPPRDKQNPIEENTAVSKLFEWLMNMPNMLTVDPPDHTRLRRLVSKSFTPR

MIEDLRPRIQQIADELLDVVQEQGKMEIIADFAYPLPIIVISEMLGIPTNDRNQFRAWTQ

ELMNASVDPGQGTAVTATLEKFINYIEVLFNEKRLNPSDDLISALVQAKEQEDKLSKNEL

LSTIWLLIIAGHETTVNLISNGVLALLQHPEQMNLLGQNPSLLPSAVDELLRYAGPIMFS

SRFASEDVTIHGNRIRKGELVLLSLTAANIDPNIFPYPEELNISREENNHLAFGAGIHHC

LGAPLARLEGQIALGTLLKRLPNLRLAIEADQLIYNHSKIRSLAKLPVIF

>CYP134A6 *Bacillus bombysepticus*

MSNTIQTVNILTEEFQENPYKYFSYLRQNDPVHYEAEIDSYFISRYQDVRNILNDTETFT

TKSLAERAEPVMRGPVLAQMRGKEHVAKRKIVLRSFMGDALQKLMPLIKKNAEDLLFPHL

PNGKIDLINDFGRTFAVYVTMDMIGLDKKDHKKIGEWHSGVADFITSINQPPEAKKHSLW

CSEQLANYLEPIIKERRLNPQEDLISKLCSAKYEGIAMTDTDILALILNILLAATEPGDK

TLALLIYNLINQPKQLQDVLSDRSLVPLAIAETLRYNPPVQLIPRQLSKDTEISGIQLSK

GTTVFCMIGAANRDPNAFERPDEFNIYRPDLDIKKAFSGAARHLAFGSGIHNCVGAAFAK

SEIEIVVNVVLDNMKNIKLEEDFQYVEKGLYTRGPISMPILFDKLV

>CYP102A8 *Bacillus cereus* FRI-35

MDKKVSAIPQPKTYGPLGNLPLIDKDKPTLSFIKIAEEYGPIFRMQTLSDTIIVVSGHEL

VAEVCDETRFDKSIEGALAKVRAFAGDGLFTSETQEPNWKKAHNILMPTFSQRAMKDYHA

MMVDIAVQLVQKWARLNPNENVDVPEDMTRLTLDTIGLCGFNYRFNSFYRETPHPFITSM

TRALDEAMHQLQRLDIEDKLMWRTKRQFQHDIQSMFSLVDNIIAERKNSGNQEENDLLSR

MLHVQDPETGEKLDDENIRFQIITFLIAGHETTSGLLSFAIYFLLKNPDKLKKAYEEVDR

VLTDPTPTYQQVMKLKYIRMILNESLRLWPTAPAFSLYAKEDTVIGGKYPIKKGEDRISV

LIPQLHRDKDAWGDNVEEFQPERFEDLDKVPHHAYKPFGNGQRACIGMQFALHEATLVMG

MLLQHFEFIDYEDYQLDVKQTLTLKPGDFKIRIVPRNQTISHTTVLAPVEEKLKNHEIKQ

QVQKTPSIIGADNLSLLVLYGSDTGVAEGIARELADTASLEGVQTEVAALNDRIGSLPKE

GAVLIVTSSYNGKPPSNAGQFVQWLEELKPDELKGVQYAVFGCGDHNWASTYQRIPRYID

EQMAQKGATRFSTRGEADASGDFEEQLEQWKESMWSDAMKAFGLELNKNMEKERSTLSLQ

FVSRLGGSPLARTYEAVYASILENRELQSSSSERSTRHIEISLPEGATYKEGDHLGVLPI

NSEKNVNRILKRFGLNGKDQVILSASGRSVNHIPLDSPVRLYDLLSYSVEVQEAATRAQI

REMVTFTACPPHKKELESLLEDGVYHEQILKKRISMLDLLEKYEACEIRFERFLELLPAL

KPRYYSISSSPLIAQDRLSITVGVVNAPAWSGEGTYEGVASNYLAQRHNQDEIICFIRTP

QSNFQLPENPETPIIMVGPGTGIAPFRGFLQARRVQKQKGMNLGEAHLYFGCRHPEKDYL

YRTELENDERDGLISLHTAFSRLEGHPKTYVQHVIKEDRIHLISLLDNGAHLYICGDGSK

MAPDVEDTLCQAYQEIHEVSEQEARNWLDRLQDEGRYGKDVWAGI

>CYP106B1 *Bacillus cereus* FRI-35

MASPENVILVHEISKLKTKEELWNPYEWYQFMRDNHPVHYDEEQDVWNVFLYEDVNRVLS

DYRLFSSRRERRQFSIPPLETRININSTDPPEHRNVRSIVSKAFTPRSLEQWKPRIQAIA

NELVQHIGKYSEVNIVEEFAAPLPVTVISDLLGVPTTDRKKIKAWSDILFMPYSKEKFND

LDVEKGIALNEFKAYLLPIVQEKRYHLTDDIISDLIRAEYEGERLTDEEIVTFSLGLLAA

GNETTTNLIINSFYCFLVDSPGTYNELREEPTLISKAIEEVLRYRFPITLARRITEDTNI

FGPLMKKDQMVVAWVSAANLDEKKFSQASKFNIHRIGNEKHLTFGKGPHFCLGAPLARLE

AEIALTTFINAFEKIALSPSFNLEQCILENEQTLKFLPICLKTQ

>CYP107J3 *Bacillus cereus* FRI-35

MSMKNKVGLSIEDGINLASAQFKEDAYEIYKESRKKQPILFVNQVEIGKEWLITRYEDAL

PLLKDNRLKKDWKNVFSQDTKNMYLSVDNSDHLTTHMLNSDPPNHSRLRSLVQKAFTPKM

IAQLDGRIQRIADDLISDIERKGTLNLVDDYSFPLPIIVISEMLGIPKEDQAKFRIWSHA

VIATPETPEEIKETEKQLSEFITYLQYLVDVKRKEPKEELVSALILAESEGHKLSARELY

SMIMLLIVAGHETTVNLITNTVLALLENPNQLQLLKDNPKLIDSAIEEGLRYYSPVEVTT

ARWAAEPFQIHHQTIQKGDMVIIALASANRDETVFENPEIFDITRENNRHIAFGHGSHFC

LGAPLARLEAKIAITTLFNRMPELQIKGDRKDIKWQGNYLMRSLEELPLTF

>CYP107DY3 *Bacillus cereus* FRI-35

MKKLTFNDLNSPETMRNPIMFYKNLMEQKERFFHIDDFYGMGGAWVVFHYDDVVAILKDS

RFIKDLRKFTPPHYKQNPIEENTAVSKLFEWLMNMPNMLTVDPPDHTRLRRLVSKSFTPR

MIEDLRPRIQQIADELLDVVQEQRKMEIIADFAYPLPIIVISEMLGIPVTDRNQFRAWTQ

ELMKASVDPGQGTAVTATLEKFINYIEVLFNEKHLNPSDDLISALVQAKEQEDKLSKNEL

LSTIWLLIIAGHETTVNLISNGVLALLQHPEQMNLLRQDPSLLASAVDELLRYAGPIMFS

SRFASEDVKIHGNRIRKGELVLLSLTAANIDPNIFPYPEELNISREENNHLAFGAGIHQC

LGAPLARLEGQIALDTLLKRLPNLRLAIEADQLIYNHSKIRSLASLPVIF

>CYP109T4 *Bacillus cereus* FRI-35

MKTPYQSIDMIPTSKLIGKKALEDPYKPFAWYKEMREKEPICFNHQADMWNVFLYEDVKT

VLEDKEHFSNIMPEKKKSPFPQSILGMDPPKHTQIRSIVNRSFTPKSLREWEPRIQQITN

DILNQLSNRKTFDIVRELFYPLPVIVIAEMLGVSAKDMERFKKWSDIIVSSPSHDDPDYL

AEFFHTRLQAENELGDFFKEIIQLNREKSQKDANDIISLLVQSEAEKSISGKELVSFCKL

LLVAGNETTTNLLGNALYCFIEYPNVYEQLQQDISLIPKSIEEVLRYRSPVQRITRRVKK

EIQLKDQTLQVDQIISAWVGSANRDSHQFTDGDSFNIYRGRNPHLTFGHGIHFCLGAPLA

RLEAKIVLTELIKRYKSFSFIDENLPTPISNSSTIYGLSSFPVKSELLHIK

>CYP102A52 *Bacillus endophyticus*

MEKERTIPQPKTYGPLGSLPLMDKDKPMQTFMKIGQELGPIFQFQFPHALGTFVSGHDLV

AEVCDEEKFQKQLGVSLTSAREFAGDGLFTSYTEEPNWKKAHNILLPSFSQRAMKGYHHM

MLDIATQLVEKWERLNKEEEIDVPEDMTRLTLDTIGLCGFGYRFNSFYREGAHPFVDSMV

RALDESMNKMNRLPIQDKLMVRKKRQFQEDKMFMFELADEIIKERKKQGEVDGDDLLAHM

LNGKDPETGEKLDDENIRYQMITFLIAGHETTSGLLSFAIYYLLKNPRVLKKAYKEVDEV

LTGMPTYEDVRKLKYIRMILNEALRLWPTAPAFSLYAKEDIMLGGKYPIEKNQSVSVLLG

NLHRDKRVWGENVEEFRPERFQDEEKIPHHAYKPFGNGQRACIGQQFALHEATLVLGMIL

QRFNIVDHRNYELKVKETLTLKPDNFTIKVSTREREYAALNEHKSENKAAEKVEQDVIIE

EHNTPLLVLHGSNLGTAEGIANELAELGERYGFSTTIASLNSYSGKLPISGAVLIVSASY

NGKAPDNATEFVNWLETSKNDLSGVHYAVFGCGDRNWASTYQRIPRVIDEKMTKLGAERI

VDRGEGDASGDFELHLDEWKNQLTKKVKETFNVEETKEVAEKKGLNVTFVKENVQHPLVK

TYKAQEAEILLNKELQREESGRSTRHIEISLPNGVSYKEGDHLGILPQNSAELVNRVLKR

YGLDGSESIILEGDVYGLQHLPLNKPVRVRDVLTFNVELQEPVSRKALQELAEATVCPPH

KYELEALLGDHYSENVTNKRLTMLSLLEKYEACELSFERFLTLCPPLKPRYYSISSSPLK

SNQKASITVSVVEGKAWSGDGMYHGVASSYLAQCQAGEKVMMFINTPQSRFELPADESRP

LIMVGPGTGVAPFRGFLQARSLLKEKGKTLGEAHLYFGCRNEAHDFIYKDELEKAESDGI

VTLHTAFSRMESCDKTYVQHLIEQDAAEVISILVEKEGALYICGDGKSMAPAVESTLKKA

YQDIKHKSEEEAERFLLDLEEQGRFAKDVWAG

>CYP106A10*Bacillus endophyticus*

MEKEVIALKEIMKAKKRTDEFFPINWYKEKLESDPVYFHEETNTWNVFKYKHVKEVLSNY

KFFSSEGERTTISVGAKSKEGQTPDKVQLTSVDPPAHRKSRSLLSAAFTPRSLKEWEPRI

RQIVRNLLDNIEENSENDIVQVLTGPLPAVVMADLFGLSGTDQMLFKKWVDILFQPFDKN

KQEDIEAQKQKAAKEYYEYLYPYVAERRFNLSDDIISDLIRAEVDDEKFTDDEIVRITMM

ILGAGIETTSHMLANTFYALLYDDQSIYKELRSNIELVPNTVEEMLRYRFHMSRRDRTVK

QDNDLLGVPLKKGDVVIAWMSAANVDEEVFENAFSLDIHRSNNKKHLTFGSGAHFCLGAP

LARLEGQIALEEFVQRFSKIEPVEGFELEENLTDSATGQSLTHLPLKVYK

>CYP107H10 *Bacillus endophyticus*

MNTTNIESFSFNTSEFFQNPYPFYEEMRSINPIYKSTLLKYQGWYITGYEETLKVLKDSR

FHSRPPIPAFSRNYENIRRIQENMMLFTTPQHHRRLRSLVTKFFTPNIVNECRPFIEETV

DELIDQVSCRGTMEVVSDYAFPLASSVIAKILGVPVKDYSLFKEWTATFIQSIDFTRSPK

TLGNSRKATEEIIAYFKKIIREREQEPKEDLISKLIQEKQREEKLSYDELIATCVLLLIA

GHETTVNLISSSIFLLIQNPRQLEKLQDDPELITTAIEEFLRFESPAQLIARTASEDIII

NEAVIRKGEQVYLFLGAANRDSRKFRNSHQLEITRNPNPHLAFGHSRHFCLGSSLARLEA

KIAIERLLQNVQNLQAVDGNGHYRKIIGFKSLVELHVTFDSYPDENFI

>CYP107DE3 *Bacillus endophyticus*

MENSNQRLPEKEVLGLLSGKNGEDPFPLFAQLREIGPVISISSPLPGTNQQAWLVTRMEE

AMQLLKDHTHFTVDQSTIEGQVDIRKGNSDASAPPTFLTGKSMLSVDNPDHKRLRRLVSK

AFTPRYMESLRPRVQKIADELLDQVINKGEMDIVKDYAYPLPINVISDMLGVPQSDQGKI

HKWSSAIAHGLGLGRKEPGVAEQIKAFGEYIAKLVEDKRHHPSDDLISQLIAIEEEGDKL

SIEELLSMISLLIFAGHETTSNLIANGTLILLDRPEQLEKLKENADLVPSAVEELLRFNG

PATIAGPRFATEDIEFAGQQMSKGDMVIPILKSANRDELKFADSEDLDITREIKQHLAFG

HGIHSCLGAPLARIEGDVAFSTLLKRMPNLRLSVPRGNVNWEFTLSSQGLSSLPVAF

>CYP109U1 *Bacillus endophyticus*

MKQKTVGFFSRHTFSTSEENWNGRFEWYEKMRKASPITYNEEEKCWDIFLYEDIEAIIKN

KEIFSSQRPVQQGEPNILSLDPPRHTQLRSLVSKAFTPRELNLWKPRIEQITENLIDDMK

GKATFDLIKDLAYPLPVMVIADILGVPEEDMGDFKRWSDLLVAGPEDPTEEALQRLQKLR

TQSITEMDTYFEEIIKYKRTHPKEDIISILIDAEIEGQKLTDGEIVSFCRLLLAAGNETT

TNLIGNTMYGLLEEPSHYQELQQNPEYITLAIEEGLRYRSPVQALHRIAKEDKEIKGQNI

RKGDSVTLWIGSANRDENKFENANHFIINRKPNPHLSFGKGIHFCLGAPLARMEGQIAFE

QLTKKFSKICIPHNFELLPIQSAFVFGLKEFPLEGQEN

>CYP107H5 *Bacillus glycinifermentans*

MTTELDSVLGIDRDFWREPYPFYDKLRSIDPVYKGTVLKQPGWYVTGHNEAMTIFKDARF

KNRIPLPETSTKYEALKNVQQNMLLFKNDSDHKRMRTLVGKEFTPKAADVLRPFIKEAAD

ELLDQLQTGTTADIVSQFAFPLASLVIARILGVPKEERHQFRQWTANLIETIDFTRSRQT

LVKGSDTASRLTAYVRDLISKRKAVPQDDLITTFISAGKLTEEEVLATCVLLVIAGHETT

VNLISNGLLSLMKHPAQLGALKENPELITSAVEEFLRYESPTQLTARTASEDCGINGHVI

RKGEQVYILLGAANRDPNVFDHPHVLDIERHPNPHLAFGYGAHVCPGSSLARLEAQIAIQ

ALLERAPGLRLAGSAVQYRRLLGFRALASLPVILN

>CYP107J10 *Bacillus glycinifermentans*

MSSKHEKSSAVLSEIDLSSAAFKHEAYDFYKELRASRPLYPVSLGELGQGWLITRYDDAV

HLLKDARLKKDVENVFAENEKPVISKTKEPLTEHMLNSDPPDHTRLRSLVQKAFTPRMIL

QLEDRIQHIANSLLDNVQRNSMNLVADFSFPLPIIVISEMLGIPLEDRQKFKNWSQAIID

FTDTPESLDKIKQFAEYLQYLVHKKRNEPADDLISALIQAESEGTTLSTKELYSTIMLLI

VAGHETTVNLITNMIFALLNHPDQLETLQQNPNLIDSAIEEALRFHSPVELTTLRWAAEP

LTLHGQEVKRKDVIVISLASANRDEKVFPDADVFDIARKNNRHIAFGHGSHFCLGAPLAR

LEAKIAVPALLRRYPDVQIQGRREDIKWKGNFLMRSLEELPLCF

>CYP107JF3 *Bacillus glycinifermentans*

MSKIDFLNKEFIRNPFPVYSEVREQEPVYRFLLPSGHYAWIVTRYDDAVKVLSDSKFIAN

PLHLNEADDPAEPPHKKIISRNLLSVNPADHRRLRRLVQKAFTPRMVERLRGRIEEIANE

LLDNVQDKGEMNLIEDYAFPLPIIVICEMLGIPHEDQDTFRKWSDVIMEGVNNPHFEQQS

EEVMIAFVDYLRDLIARRRKELKEDLISDLISVEVEGDVLSEHEMYALVFVLIIAGHETT

VNLIGNGILALLHHPDQKAKLQNQPELIHSAIEEMLRYDGPAEVSNIRWATEDAKLGGKT

IRKGEMLFISFSSANRDPEKFADPDSFNITREANNHLAFGKGVHHCLGAPLARLEGEIAI

GTLLRRMPDLRLKTNADFLEWRPGMIIRGLKEIPLLF

>CYP109A5 *Bacillus glycinifermentans*

MTNSIQTSNRYANWIPMKEISSSKDRLFPFPIYNRLRQTSPVRFDDDRKCWDIFAYEDVQ

YVLKSPKLFSSKRGGDMQGKTMLTMDPPRHTSMRAIVNKAFTPKAVKQLESHIGEVTAFL

LDETRDRRSFDLVDDLAAPLPVIIIAELLGVPPEDRSMFKHYSDILVSGASDSSDEAVKL

MSKRRKEGNDYLKDYFKRIIKERKKSPKEDLISLLIQAEVEGEKLTEEELLGFCTLLLVA

GNETTTNLIANAVRYLTEDAHTQEAVRHDLSLVPHLVEETLRYYPPVQAIGRVAAQDTEI

RGVSIPKGSSVISWVASANRDETKFDDPESFRLDRKSNPHMSFGFGIHFCLGAPLARLEA

NIALSHLLRHASVERDDRVKLEAIQSPFVFGVRRLPVRLLY

>CYP109B4 *Bacillus glycinifermentans*

MSQTKQQNPIQKALINGKNRQDPYDPFPWYEKMRTESPIHYDEDSKVWSVFRYNDVKRVI

SDKDFFSNQFPQIGTGNTFAKTMISMDPPKHTRIRSIVNRAFTPRVMKEWEPRIRELTNE

LLADVRGREEIDLVQNFSYPLPVIVISELLGVPSVYKHHFKEWSDLLVSLPKSDRPEDVN

EWKNIRDQGEEELTAFFEKMIEEKRQNLGNDLISLLIKAEQEGDKLSPDELVPFCNLLLM

AGNETTTNLVSNAVYSILETPGAYDELAGHPELIPQAVEEAVRFRAPAPMIVRFVKQDTE

IRGVSLKKGEGVVAFLASANRDEAKFERAHEFDIHRHPNPHIGFGHGIHFCLGAPLARLE

AAIALEALLKQYSSMEKLAIVPMADSSMYGLKHFRLRVKSAGKTAPYSF

>CYP102A51 *Bacillus megaterium* DSM 319

MTIKEMPQPKTFGELKNLPLLNTDKPVQALMKIADELGEIFKFEAPGRVTRYLSSQRLIK

EACDESRFDKNLSQALKFVRDFTGDGLFTSWTHEKNWKKAHNILLPSFSQQAMKGYHAMM

VDIAVQLVQKWERLNADEHIEVPEDMTRLTLDTIGLCGFNYRFNSFYRDQPHPFITSMVR

ALDEAMNKLQRANPDDPAYDENKRQFQEDIKVMNDLVDKIIADRKASGEQSDDLLTHMLN

GKDPETGEPLDDENIRYQIITFLIAGHETTSGLLSFALYFLVKNPHVLQKAAEEAARVLV

DPVPSYKQVKQLKYVGMVLNEALRLWPTAPAFSLYAKEDTVLGGEYPLEKGDELMVLIPQ

LHRDKTIWGDDVEEFRPERFENPSAIPQHAFKPFGNGQRACIGQQFALHEATLVLGMVLK

HFDFEDHTNYELDIKETLTLKPEGFVVKAKSKKIPLGGIPSPSPEQSAKKVRKKAENAHN

TPLLVLYGSNMGTAEGTARDLADIAMSKGFAPQVATLDSHAGNLPREGAVLIVTASYNGH

PPDNAKQFVDWLDQASADEVKGVRYSVFGCGDKNWATTYQKVPAFIDETLAAKGAENIAD

RGEADASDDFEGTYEEWREHMWSDVAAYFNLDIENSEDNKSTLSLQFVDSAADMPLAKMH

GAFSTNVVASKELQQPGSARSTRHLEIELPKEASYQEGDHLGIIPRNYEGIVNRVTARFG

LDASQQIRLEAEEEKLAHLPLAKTVSVEELLQYVELQDPVTRTQLRAMAAKTVCPPHKVE

LEALLEKQAYKEQVLAKRLTMLELLEKYPACEMEFSEFIALLPSIRPRYYSISSSPRVDE

KQASITVSVVSGEAWSGYGEYKGIASNYLAELQEGDTITCFISTPQSEFTLPKDPETPLI

MVGPGTGVAPFRGFVQARKQLKEKGQSLGEAHLYFGCRSPHEDYLYQEELENAQNEGIIT

LHTAFSRVPNQPKTYVQHVMEQDGKKLIELLDQGAHFYICGDGSQMAPDVEATLMKSYAG

VHQVSEADARLWLQQLEEKGRYAKDVWAG

>CYP106A1 *Bacillus megaterium* DSM 319

MNKEVIPVTEIPKFQSRAEEFFPIQWYKEMLNNSPVYFHEETNTWNVFQYEHVKQVLSDY

EFFSSDGQRTTIFVGDNSKKKSTSPITNLTNLDPPDHRKARSLLAAAFTHRSLKNWEPRI

KQIAADLVEAIQKNPTINIVDDLSSPFPSLVIADLFGVPVKDRYQFKKWVDILFQPYDQE

RLEEIEQEKQRAGAEYFQYLYPIVIEKRSNLSDDIISDLIQAEFDGETFTDEEIVHATML

LLGAGVETTSHAIANMFYSFLYDDKSLYSELRNNRELAPKAVEEMLRYRFHISRRDRTVK

QDNELLGVKLKKGDVVIAWMSACNMDETMFENPFSVDIHRPTNKKHLTFGNGPHFCLGAP

LARLEMKIILEAFLEAFSHIEPFEDFELEPHLTASATGQSLTYLPMTVYR

>CYP109A2 *Bacillus megaterium* DSM 319

MNPKAVKRENRYANLIPMQEIKSVEQQLYPFDIYNSLRQEAPIRYDESRNCWDVFDYETV

KYILKNPSLFSSKRAMEERQESILMMDPPKHTKLRNLVNKAFTPRAIQHLEGHIEEIADY

LLDEVSSKEKFDIVEDFAGPLPIIVIAELLGVPIQDRALFKKYSDDLVSGAENNSDEAFA

KMMQKRNEGVIFLQGYFKEIIAERQQNKQEDLISLLLEAEIDGEHLTEEEVLGFCILLLV

AGNETTTNLITNGVRYMTEDVDVQNEVRRDISLVPNLVEETLRYYPPIQAIGRIAAEDVE

LGECKIKRGQQVISWAASANRDSAKFEWPDTFVVHRKTNPHVSFGFGIHFCLGAPLARME

GKIAFTKLLEKGGFSKVQNQSLKPIDSPFVFGVKKYEIAFNNA

>CYP109E1 *Bacillus megaterium* DSM 319

MKTERENGIVRQVNTIQTKEERFNPFSWYEEMRNTAPVQWDEERQVWDVFHYDGVKEVLE

QKNIFSSDRRPPQNQRQTALGTSLINIDPPKHAEMRALVNKAFTPKAMKAWEPKIARITN

ELLQEVEHLEDIDIVEHLSYPLPVMVIADILGVPIEDQRQFKDWSDIIVAGPSNNERETL

EKLQQEKMKANDELETYFYRIIEEKRTRPGDDIISVLLQAKEEGKQLTDEEIVGFSILLL

IAGNETTTNLISNTIYCLMEDKASFERLKREKELLPSGIEEVLRYRSPVQALHRIVKEDV

TLAGKKLKAGEHVVPWMGSAHRDAEYFEDPEVFKIDRKPNVHMAFGRGIHFCLGAPLARI

EAKIMLAELIDRYPQMDWSPSFELKPIESTFVYGLKELLIRKNV

>CYP1756A1 *Bacillus megaterium* DSM 319

MENLTQYGEIATFRVAHKRFYVTRDPQLIKDVVITNSKAFQKIKLTHMFKTLLGEEMLWT

DEALYMSPIQPSQLKQHLTYNKEAIAKIIEKHTETWEEGQLRTIVKDIRQIVVAVLLQLV

FGISIEEKDKIHYVQALMRKKEKLGKIYIRLPLHQPDSDEQLEQLLFERVQMRIQNKTGG

NDLLQYILNSYGEDSDEREIYEQLNSIFLSMYEMITHVCSWSIHLLSQNTREHLQLHKEI

QAYASGESLSTKNLTYMRKIIAESMRLYPPLWLFGRQAREDIQIDGYNIKKGEIMLISSY

MMHRHEDYFLEPSEFLPDRFEKGGSIDVPSYMYMPLGIEHQAERGMDYITEIVTIFLSEM

TKRFLFQLTKPESIAPMAGVMLNMKEELKVNVHKVHAQS

>CYP102A51 *Bacillus megaterium* WSH-002

MTIKEMPQPKTFGELKNLPLLNTDKPIQTLMKIADELGEIFKFEAPGRVTRYLSSQRLIK

EACDESRFDKNLSQALKFVRDFAGDGLFTSWTHEKNWKKAHNILLPSFSQQAMKGYHAMM

VDIAVQLIQKWERLNADEHIEVPEDMTRLTLDTIGLCGFNYRFNSFYRDQPHPFITSMVR

ALDEAMNKLQRANPDDPAYDENKRQFQEDIKVMNDLVDKIIADRKASGEQSDDLLTHMLN

GKDPETGEPLDDENIRYQIITFLIAGHETTSGLLSFALYFLVKNPHVLQKAAEEAARVLV

DPVPSYKQVKQLKYVGMVLNEALRLWPTAPAFSLYAKEDTVLGGEYPLEKGDELMVLIPQ

LHRDKTIWGDDVEEFRPERFENPSAIPQHAFKPFGNGQRACIGQQFALHEATLVLGMMLK

HFDFEDHTNYELDIKETLTLKPEGFVVKAKSKKIPLGGIPSLSTEQSAKKVRKKVENAHN

TPLLVLYGSNMGTAEGTARDLADIAMSKGFAPQVATLDSHAGNLPREGAVLIVTASYNGH

PPDNAKEFVDWLDQASADEVKGVRYSVFGCGDKNWATTYQKVPAFIDETLAAKGAENIAE

RGEADASDDFEGTYEEWREHMWSDVAAYFNLDIENSEDNKSTLSLQFVDSAADMPLAKMH

GAFSANVVASKELQQPGSERSTRHLEIELPKEASYQEGDHLGVIPRNYEGIVNRVATRFG

LDASQQIRLEAEEEKLAHLPLGKTVSVEELLQYVELQDPVTRTQLRAMAAKTVCPPHKVE

LEALLEKQTYKEQVLAKRLTMLELLEKYPACEMEFSEFIALLPSMRPRYYSISSSPRVDE

KQASITVSVVSGEAWSGYGEYKGIASNYLAELQEGDTITCFISTPQSGFALPKDSQTPII

MVGPGTGVAPFRGFVQARKQLKEQGQSLGEAHLYFGCRSPHEDYLYQEELENAQNEGIIT

LHTAFSRVPNQPKTYVQHVMEQDGTKLIELLDQGAHFYICGDGSQMAPDVEATLIKSYAD

VHEVSEADARLWLQQLEEKGRYAKDVWAG

>CYP106A1 *Bacillus megaterium* WSH-002

MNKEVIPVTEIPKFQSRTEEFFPIQWYKEMLNNSPVYFHEETNTWNVFQYDHVKQVLSNY

EFFSSDGQRTTIFVGDNSKKKSTSPITNLTNLDPPDHRKARSLLAAAFTPRSLKNWEPRI

KQIAADLVEAIQKNSTINIVEDLSSPFPSLVIADLFGVPVKDRYQFKKWVDILFQPYDQE

RLEEIQQEKQCAGAEYFQYLYPIVVEKRSNLSDDIISDLIQAEVDGETFTDEEIVHATML

LLGAGVETTSHAIANMFYSFLYDDKSLYSKLRNNRELAPKAVEEMLRYRFHISRRDRTVK

QDNELLGVKLKKGDVVIAWMSACNMDKNMFENPFSVDIHRPTNKKHLTFGNGPHFCLGAP

LARLEMKIILEAFLETFSHIEPFEDFELESHLTASATGQSLTYLPMTVYR

>CYP109A2 *Bacillus megaterium* WSH-002

MNPKAVKRENRYANLIPMQEITSVEQQLYPFDVYNSLRQEAPIRYDESRNCWDVFDYETV

KYILKNPSLFSSKRAMEERQESILMMDPPKHTKLRNLVNKAFTPRAIQHLEGHIEEIADY

LLDEVSSKKKFDIVEDFAGPLPIIVIAELLGVPIQDRALFKKYSDDLVSGAENNSDEAFA

KMMQKRNEGVLFLQGYFKEIIAQRQRKKQEDLISLLLEAEIDGEQLTEEEVLGFCILLLV

AGNETTTNLITNGVRYMTEDLDVQNEVRQDISLVPNLVEETLRYYPPIQAIGRIAAEDVE

LGECKIKKGQQVISWAASANRDSVKFERPDTFVVHRKTNPHVSFGFGIHFCLGAPLARME

GKIAFTKLLEKGEFSKVQNQSLKPIDSPFVFGVKKYEIAFNNA

>CYP109E1 *Bacillus megaterium* WSH-002

MKTERENGIVRQVNTIQSKEERFNPFSWYEEMRNSAPVQWDEERQVWDVFHYDGVKEVLE

QKNIFSSDRRPPQNQRQTALGTSLINIDPPKHAEMRALVNKAFTPKAMKAWEPKIARITH

ELLQEVEHLEDIDIVEHLSYPLPVMVIADILGVPIEDQRQFKDWSDIIVAGPSNNERETL

EKLQQDKMKANDELETYFYKIIEEKRTHPGADIISVLLQAKEEGKQLTDEEIVGFSILLL

IAGNETTTNLISNTIYCLMEDKASFERLKREKELLPSAIEEVLRYRSPVQALHRIVKEDV

VLAGKKLKAGEHVVPWMGSAHRDAQYFEDPDVFQIDRKPNIHMAFGRGIHFCLGAPLARI

EAKVMLAELIDRYPHMDWSPAFELKPIESTFVYGLKELLIRKHV

>CYP1756A1 *Bacillus megaterium* WSH-002

MITLPVIQGPSSYKLTGHLQKFRENPLGFLENLTQYGEIATFRVAHKRFYVTRDPQLIKD

VVITNNKAFQKIKLTHMFKTLLGEEMLWTDEALYMSPIQPSQIKQHLTYNKEAIAKIIEK

HTETWEEGQLRMIVKDIRQIVVAVLLQLVFGISIQDKDKIHYVQALMRKKEKLGKIYIRL

PLHQLDSDEQLEQLLFERVQMRVQNKTEGKDLLQYILNSCGEDIDEREIYQQLNSIFLSM

YEMITHVCSWSIYLLSQNTREHLQLHKEIQAYASGESLSTKNLTYMRKIIAESMRLYPPL

WLFGRQAREDIQIDGYSIKKGEIMLISPYMMHRHEDYFLEPSEFLPDRFEKGGSIDVPSY

MYMPLGIDHQAERGMDYITEIVTIFLSEMTKRFLFQLTKPESIAPMAGVMLNMKEELNVN

VHKVHTQS

>CYP102A7 *Bacillus paralicheniformis*

MNMLNGIPIPKTYGPLGNLPLLDKNKVSQSLWKIADEMGPIFQFKFADAIGIFVSSHELV

KEVSDESRFDKNMGKGLLKVREFSGDGLFTSWTKEPNWRKAHNILLPSFSQKAMKGYHPM

MQDIAVQLIQKWSRLNQDESIDVPDDMTRLTLDTIGLCGFNYRFNSFYREGQHPFIESMV

RGLSEAMRQTKRFPLQDKLMVQTKRQFDSDVESMFSLVDRIIADRKQAGGESGNDLLSLM

LHAKDPETGEKLDDENIRYQIITFLIAGHETTSGLLSFAIYLLLKHPDKLKKAYEEADRV

LTDPVPSYKQVQQLKYIRMILNESIRLWPTAPAFSLYAKDETVIGGKYLIPKGQSVTVLI

PKLHRDQSVWGEDAEGFRPERFEQMDSIPAHAYKPFGNGQRACIGMQFALHEATLVLGMI

LQYFDLEDHANYQLKIKESLTLKPDGFTIRVRPRKKEAMMVTPGAQPEENMRQEEKPSAP

AAENTHGTPLLVLYGSNLGTAEEVAKELAEEAREQGYRSRTAELDQYPGALPAEGAVIIV

TASYNGNPPDCAREFVNWLEHDQTGDLHGVKYAVFGCGNRSWASTYQRIPRLIDSALENR

GAQRLHKLGEGDAGDDFEGQFESWKNDLWPLLRTEFSLSEPDPNQTETDRQAISVEFVSA

PAAAPLAKAYQVFTAKISANRELQCEESGRSTRHIEISLPEGTAYQEGDHLGVLPQNSGV

LIERVFQRFGLNGDEQILISGRNQASHLPLERPVHVKDLFQHCVELQEPAARAQIRELAA

HTVCPPHQRELEDLLKDDVYKNQVLKKRLTMLDLLEQYPACELPFARFLALLPPLKPRYY

SISSSPQLNPRQTSITVSVVSGPALSGRGQYKGVASNYLAGLAPKDAISCFIREPQSGFR

LPEDPETPVIMVGPGTGIAPYRGFLQARRIQRDAGIKLGEAHLYFGCRRPDEDFLYRDEL

EQAEKDGIVHLHTAFSRLEGRPKTYVQDLLREDADMLIHLLNEGGRLYVCGDGSRMAPAV

EQALCEAYRIVQGASQEESESWLSGLLEEGRYAKDVWDGGVSQHDVNADSIART

>CYP107H7 *Bacillus paralicheniformis*

MTTELHEAGPRLKSDSDFWRDPYLFYDKLRSVHPVYKGTVLKHLGWYVTGYEEAAAILRD

AVFKNRVPLPEASTKYEQLHELQRNMMLFQNESGHKRMRTIVGKEFMPQKTASFRPVIEE

IVHELLDQLENKKMADMVSEFAFPLASLIIADMLGVPAEERYQFRQWTADLIQTIDLTRT

RKVLAKGGDTVAKLTAYFKDLIEKRKAHPSQDLISTFAGHEQLAEDEVLATCILLVIAGH

ETTVNLLTNGLFLLMNHPGQLSALKENPLLIESAVEECLRYESPTQLTARTASEDCEING

KIIKKGEHVYILLGAANRDPKIFQNPHVFDITRKPNPHLAFGAGAHVCLGSALARLEAQI

AIPALLERLPKLKLASTDIPFRRLIGFRSLAELPVILN

>CYP107J9 *Bacillus paralicheniformis*

MSSKQEESFAILTERELSSAAFKDEAYEFYKRLRASRPVYPVSMGDLGEGWLITRYEDAV

HILKDARMKKNYENVFTEEELENFSALENEEPLSKHMLNSDPPDHGRLRSLVQKAFTPRM

VLQLENRIQKIADSLLDQAEPNHSMNLVDDFAFPLPIIVISEMLGIPLEDRQKFRVWSQA

VIDFSDTPESLEEYKHKIGEFAEYLEYLVCKKRDEPAEDLVSALIQAESEGTTLSIEELY

STIMLLIVAGHETTVNLITNMTFALLNHPEQLEKLHQNADLIDSAIEEALRFHSPVELTT

LRWAAEPFTLHGQEMKRKDVIIISLASANRDDTVFPNADRFNIERKDNRHLAFGYGSHFC

LGAPLARLEAKIAIQTLLRRFEHIEIKGEREQIKWKGNFLMRALDELPLSF

>CYP109A6 *Bacillus paralicheniformis*

MANSNSLQSSKHYANWIPMKEISSSNDRLFPFPIYNRIRKTSPVRYDDERKCWDIFSYED

VQFVLKNPKLFSSKRGGNMEGKSILTMDPPKHTKMRAIVNKAFTPKAVKELEPHIEEVTA

FLFNEAKQKDLFDVVDDLAAPLPVIIIAELLGVPAEDRLMFKHYSDILVAGAEDRSAEAA

EQMYKRREEGNRFLADYFKNIIKQRKKEPKDDLISLLLRAEVDGKSLTEEELLNFCIILL

VAGNETTTNLIANSVRYLTEDKATQEAVRQDLSLVPVFVEETLRYYPPVQAIGRTAAEDL

DIKGVRIAKGSTVINWVASANRDELKFDDPDSFKLDRKSNPHMSFGFGIHFCLGAPLARL

EAKVALDYLLRHASVEKDSSEELEAIQSPFVFGVRHLPVRLSQK

>CYP134A5 *Bacillus paralicheniformis*

MNQSLKTFSVLSEQYHENPYQYFSYLRESDPVHYEESLDSYFISRYQDVRRVLQNQDVFT

TKSLAKRAEPVMRGPVLAQMKGKEHTAKRRIVLRRFIGESLDHLTPLIKENAQRLLAPHL

EKGRIDLVNDFGKTFAVCVTMDILGLDKNDHQTVRNWHSGVADFITSLNQAPEDREHSLK

CSEQLAEYLNPIIEERRKNPGHDLISILCTSEYEGVAMSDRDIRALILNILLAATEPADK

TLALMIYHLLHHPDQMNDVLEDRTLLPQAIAETLRYKPPVQLIPRQLSEDAVIGGVELKE

GTTVFCMIGAANRDPEAFEEPDEFNIHRTDLEVKSAFSGAARHLAFGSGIHNCVGAGFAK

TEIELVANIVLDQLKNIQLEEDFVYRETGLYTRGPVSLNIRFDTKH

>CYP102A36 *Bacillus sp*. JS

MKETSPIPQPKTFGPLGNLPLIDKDKPTLSLIKLAEEQGPIFQIHTPAGTTIVVSGHELV

KEVCDEGRFDKSIEGALEKVRAFSGDGLFTSWTHEPNWRKAHNILMPTFSQRAMKDYHEK

MVDIAVQLIQKWARLNPNEAVDVPGDMTRLTLDTIGLCGFNYRFNSYYRETPHPFINSMV

RALDEAMHQMQRLDFQDKLMVRTKRQFHHDIQTMFSLVDSIIAERRANGDQDEKDLLARM

LNVEDPETGEKLDDENIRFQIITFLIAGHETTSGLLSFAIYFLLKHPDKLKKAYEEVERV

LTDAAPTYKQVLELKYIRMILNESLRLWPTAPAFSLYPKEDTVIGGKFPITTKDRISVLI

PQLHRDRDAWGKDAEEFRPERFEHQDQVPHHAYKPFGNGQRACIGMQFALHEATLVLGMI

LKYFTLIDHENYELDIKQTLTLKPGDFRIKVQTRHQEAIHADIQATEKPGPDKQKEETET

KGASVIGLNNRPLLVLYGSDTGTAEGVARELADTASLHGVRTEVSPLNDQIGKLPKEGAV

VIVTSSYNGKPPSNARQFVQWLQEIKPGELEGVHYAVFGCGDHNWASTYQDVPRFIDEQL

AEKGATRFSARGEGDVSGDFEGQLDEWKKSMWADAIKAFGLELNENADKERSTLSLQFVR

GLGESPLARSYEAAHASIAENRELQSADSDRSTRHIEIALPPDVEYQEGDHLGVLPKNSP

TNVSRILHRFGLKGTDQVTLLASGRSAGHLPLGRPVSLNDLLSYSVEVQEAATRAQIREL

AAFTVCPPHKRELEELTAEGVYQEQILKKRISMLDLLEKYEACDMPFERFLELLRPLKPR

YYSISSSPRVNPGQASITVGVVRGPAWSGRGEYRGVASNDLAERQAGDDVVMFIRTPESR

FQLPKDPETPIIMVGPGTGVAPFRGFLQARDVLKREGKTLGEAHLYFGCRNDRDFIYRDE

LEQFEKDGVVTVHTAFSRKEGMPKTYVQHLMAEHAETLISILDRGGRLYVCGDGSKMAPD

VEAALQKAYQSVHGTGEQEAQNWLRHLQDTGMYAKDVWAGI

>CYP102A48 *Bacillus sp.* JS

MKQASAIPQPKTYGPLKNLPHLEKEQLSQSLWRLADELGPIFRFDFPGVSSVFVSGHNLV

TEVCDESRFDKNLGKGLLKVREFGGDGLFTSWTHEPNWQKAHRILLPSFSQKAMKGYHSM

MLDIATQLIQKWSRLNPNEEIDVADDMTRLTLDTIGLCGFNYRFNSFYRDSQHPFITSML

RALKEAMNQSKRLGLQDKMMVKTKLQFQKDIEVMNSLVDRMIAERKANPDENIKDLLSLM

LYAKDPVTGETLDDENIRYQIITFLIAGHETTSGLLSFAIYCLLTHPEKLKKAQEEADRV

LTDDTPEYKQIQQLKYIRMVLNETLRLYPTAPAFSLYAKEDTVLGGEYPISKGQPVTILI

PKLHRDQNAWGADAEDFRPERFEDPSSIPHHAYKPFGNGQRACIGMQFALQEATMVLGLV

LKHFDMLNHTGYKLKIKEALTIKPDDFKITVKPRKTAAINVQRREQADIKTETKPKETQP

KHGTPLLVLYGSNLGTAEGIAGELAAQGRQMGFTAETAPLDDYIGKLPEEGAVVIVTASY

NGAPPDNAAGFVEWLKELEEGRLKGVSYAVFGCGNRSWASTYQRIPRLIDDMMKAKGATR

LTAIGEGDAADDFESHRESWENRFWKETLDAFDINEIAQKEDRPSLSITFLSEATETPVA

KAYGAFEGIVLENRELQSADSTRSTRHIELEIPEGKTYKEGDHIGILPKNSRELVQRVLS

RFGLQSNHVIQISGSAHMAHLPMDRPIKVADLLSSYVELQEPASRLQLRELASYTVCPPH

QKELEQLVSDDGTYKEQVLAKRLTMLDLLEDYPACEMPFERFLALLPSLKPRYYSISSSP

KAHANIVSMTVGVVKASAWSGRGEYRGVASTYLAELNTGDAAACFIRTPQSGFQMPDDPD

TPMIMVGPGTGIAPFRGFIQARSVLNKEGSTLGEALLYFGCRRPDHDDLYREELDQAEQE

GLVTIRRCYSRVENESKGYVQHLLKQDSQKLMTLIEKGAHIYVCGDGSQMAPDVEKTLRW

AYEIEKGASQEESADWLQKLQDQKRYVKDVWTGN

>CYP107K1 *Bacillus sp.* JS

MEKLMFHPHGKEFHDNPFSVLGRFREEDPIHRFELKRFGGTYPAWLITRYDDCMAFLKDT

RITRDVKNVMSQEQIKMLNVSEDIDFVSDHMLAKDTPDHTRLRSLVHQAFTPRTIENLRG

SIEQISEQLLDEMEKEDKADIMTSFASPLPFIVISELMGIPKEDRTQFQIWTNAMVDTSE

GNRELTNQALREFKEYIAKLIHDRRIKPKDDLISKLVHAEENGSKLSEKELYSMLFLLVV

AGLETTVNLLGSGTLALLQHKKEREKLKQHPEMIATAVEELLRYTSPVVMMANRWAIEDF

TYKGHSIKRGDMIFIGIGSANRDPNFFENPEILNINRSPNRHISFGFGIHFCLGAPLARL

EGHIAFNALLKRFPDIELAMPDDIQWRKNVFLRGLESLPVSLSK

>CYP109B1 *Bacillus sp.* JS

MNVLNRRQALQRALLNGKNKQDAYHPFPWYESMRKDAPVSFDEENQVWSVFLYDDVKKVI

GDKELFSSYMPQQTSSIGNSIINMDPPRHTKIRSVVNKAFTPRVMKQWEPRIQEITDELI

QSFQGRSEFDLVHDFSYPLPVIVISELLGVPSEHMDQFKAWSDLLVSTPKDKSEEAEKAY

LEERDKCEKELAAFFAGIIEEKRNKPAQDIISILVEAEETGEKLSGEELIPFCTLLLVAG

NETTTNLISNAMYSILETPGVYEELRSHPELMSQAVEEALRFRAPAPVLRRIAKRDTEIG

GHLMKEGDMVLAFVASANRDEAKFDRPHMFDIRRHPNPHIAFGHGIHFCLGAPLARLEAN

IALTSLISAFPHMECLSITPIENSVIYGLKSFRVKM

>CYP152A1 *Bacillus sp*. JS

MNEQIPHDKSLDNSLTLLKEGYLFIKNRTERYNSDLFQARLLGKNFICMTGAEAAKVFYD

TNRFQRQNALPKRVQKSLFGVNAIQGMDGSAHIHRKMLFLSLMTPPHQKRLAELMTEEWK

AAVTRWEKADQVVLFEEAKEILCRVACYWAGVPLKETEVKERADDFIDMVDAFGAVGPRH

WKGRRARPRAEEWIEVMIEDVRAGLLKTPAGTALHEMAFHKQEDGSELDSRMASIELINV

LRPIVAISYFLVFSALALHEHPKYKEWLRSGSSREREMFVQEVRRYYPFGPFLGALVKKD

FVWNNCEFKKGTSVLLDVYGTNHDPRLWDHPDEFRPERFAEREENPFDLIPQGGGHAEKG

HRCPGEGITIEVMKASLDFLVNQIEYDVPEQSLHYSLARMPSLPESGFIMSGIRQKR

>CYP102A8 *Bacillus cereus* 03BB102

MDKKVSAIPQPKTYGPLGNLPLIDKDKPTLSFIKLAEEYGPIFRIQTLSDTIIVVSGHEL

VAEVCDETRFDKSIEGALAKVRAFAGDGLFTSETQEPNWQKAHNILMPTFSQRAMKDYHA

MMVDIAVQLVQKWARLNPNENVDVPEDMTRLTLDTIGLCGFNYRFNSFYRETPHPFITSM

TRALDEAMHQLQRLDIEDKLMWRTKRQFQHDIQSMFSLVDNIIAERKSSENQEENDLLSR

MLNVQDPETGEKLDDENIRFQIITFLIAGHETTSGLLSFAIYFLLKNPDKLKKAYEEVDR

VLTDPTPTYQQVMKLKYIRMILNESLRLWPTAPAFSLYAKEDTVIGGKYPIKKGEDRISV

LIPQLHRDKDAWGDNVEEFQPERFEELDRVPHHAYKPFGNGQRACIGMQFALHEATLVMG

MLLQHFEFIDYEDYQLDVKQTLTLKPGDFKIRIVPRNQTISHTTVLAPTEEKLKNHEIKQ

QVQKTPSIIGADNLSLLVLYGSDTGVAEGIARELADTASLEGVQTEVVALNDRIGSLPKE

GAVLIVTSSYNGKPPSNAGQFVQWLEELKPDELKGVQYAVFGCGDHNWASTYQRIPRYID

EQMAQKGATRFSKRGEADASGDFEEQLEQWKQRMWSDAMKAFGLELNKNMEKERSTLSLQ

FVSRLGGSPLARTYEAVYASILENRELQSSSSERSTRHIEISLPEGATYKEGDHLGVLPI

NSEKNVNRILKRFGLNGKDQVILSASGRSVNHIPLDSPVRLYDLLSYSVEVQEAATRAQI

REMVTFTVCPPHKKELESLLEEGVYQEQILKKRISMLDLLEKYEACEIRFERFLELLPAL

KPRYYSISSSPLVAQDRLSITVGVVNAPAWSGEGTYEGVASNYLAQRHNKEEIICFIRTP

QSNFQLPENPETPIIMVGPGTGIAPFRGFLQARRVQKQKGMNLGEAHLYFGCRHPEKDYL

YRTELENDEREGLISLHTAFSRLEGHPKTYVQHVIKEDRIHLISLLDNGAHLYICGDGSK

MAPDVEDTLCQAYQEIHQASEQEARNWLDRLQDEGRYGKDVWAGI

>CYP106B1 *Bacillus cereus* 03BB102

MDSPENVILVHEISKLKTKEELWNPYEWYQFMRDNHPVHYDDEQDVWNVFLYDDVNRVLS

DYRLFSSRRERRQFAIPPLETRININSTDPPEHRNVRSIVSKAFTPRSLEQWKPRIQSIA

NELVKDIENCSEVDIVEQFAAPLPVTVISDLLGVPTTDRKKIKTWSDILFMPYSKEKFND

LDAKKGIALNEFKAYLLPIVQEKRYYLTDDIISDLIRAEYEGERLTDEEIVTFSLGLLAA

GNETTTNLIINSFYCFLVDSPATYKELREKPKLISKAIEEVLRYRFPVTLARRITEDTNI

FGPLMKKDQMVVAWVSAANLDEKKFSQASKFNIHRIGNEKHLTFGKGPHFCLGAPLARLE

AEIALTTFINAFEKIALSPSFNIEQCILENEQTLKFLPIRLKPQ

>CYP107J2 *Bacillus cereus* 03BB102

MAMKNKVGLRIEDGINLASAQFKEDAYEIYKESRKVQPVLFVNKTELGAEWLITRYEDAL

PLLKDSRLKKDPANVFSQDTLNVFLTVDNGDHLTTHMLNSDPPNHNRLRSLVQKVFTPKM

IAQLEGRIQDIADDLLNEVERKGSLNLVDDYSFPLPIIVISEMLGIPKEDQAKFRIWSHA

VIAYPETPEEIKETEKQLSEFITYLQYLVDMKRKEPKEDLVSALILAESEGHKLSARELY

SMIMLLIVAGHETTVNLITNTVLALLENPNQLQLLKENPKLIDAAIEEGLRYYSPVEVTT

SRWADEPFQIHDQTIEKGDMVVIALASANRDETVFENPEVFDITRENNRHIAFGHGSHFC

LGAPLARLEAKIAITTLFERMPELQIKGNREDIKWQGNYLMRSLEELPLTF

>CYP109T5 *Bacillus cereus* 03BB102

MKTPYESIIMIPTNKLIGKKALEDPYDPFAWYKEMREEEPICFNPQADMWNVFLYDDVKR

VLEDKEYFSNIMPEKKKPPFPQSILGMDQPKHTQIRSIVNRSFTPKALEVWEPRIQEITE

DILTQLSNRKNFDIVQELFYPLPVIVIAEMLGVSTNDMDRFKNWSDIIVSSPSHDDPDYL

KEFFHTRLQAENELENFFEEILQLNRGNSNKDSNDIISLLVQSEVDEKISGKEIVSFCKL

LLVAGNETTTNLLGNLLYCLIEHPDVYKQIQQDTALIPKAIEEVLRYRSPAQRVVRRVKK

EMQLNGQTLQVDQIISAWVGSANRDSNYFKDADSFNIHRKRNPHLAFGHGIHFCLGARLA

RLEATIVLMELIKKYKSFSFINTNLPIPISNSSSVYGLKSFPVKSEMIQIK

>CYP102A8 *Bacillus cereus biovar anthracis* CI

MDKKVSAIPQPKTYGPLGNLPLIDKDKPTLSFIKLAEEYGPIFQIQTLSDTIIVVSGHEL

VAEVCDETRFDKSIEGALAKVRAFAGDGLFTSETDEPNWKKAHNILMPTFSQRAMKDYHA

MMVDIAVQLVQKWARLNPKENVDVPGDMTRLTLDTIGLCGFNYRFNSFYRETSHPFITSM

TRALDEAMHQLQRLDIEDKLMWRTKRQFQHDIQSMFSLVDNIIAERKSSGNQEENDLLSR

MLNVKDPETGEKLDDENIRFQIITFLIAGHETTSGLLSFAIYFLLKNPDKLKKAYEEVDR

VLTDPTPTYQQVMKLKYIRMILNESLRLWPTAPAFSLYAKEDTVIGGKYPIKKGEDRISV

LIPQLHRDKDAWGDNVEEFQPERFEELDKVPHHAYKPFGNGQRACIGMQFALHEATLVMG

MLLQHFEFIDYEDYQLDVKQTLTLKPGDFKIRIVPRNETISHTTVLAPTEEKLKNHETKQ

QVQKTPSIIGADNLSLLVLYGSDTGVAEGIARELADTASLEGVQTEVAALNDRIGSLPKE

GAVLIVTSSYNGKPPSNAGQFVQWLEELKPDELKGVQYAVFGCGDHNWASTYQRIPRYID

EQMAQKGATRFSKRGEADASGDFEEQLEQWKQSMWSDAMKAFGLELNKNMEKERSTLSLQ

FVSRLGGSPLARTYEAVYASILENRELQSSSSERSTRHIEISLPEGATYKEGDHLGVLPI

NSEKNVNRILKRFGLNGKDQVILSASGRSVNHIPLDSPVRLYDLLSYSVEVQEAATRAQI

REMVTFTVCPPHKKELESLLEEGVYQEQILKKRISMLDLLEKYEACEIRFERFLELLPAL

KPRYYSISSSPLVAQDRLSITVGVVNAPAWSGEGTYEGVASNYLAQRHNKDEIICFIRTP

QSNFQLSENPETPIIMVGPGTGIAPFRGFLQARRVQKQKGMKVGEAHLYFGCRHPEKDYL

YRTELENDEREGLISLHTAFSRLEGHPKTYVQHVIKEDRIHLISLLDNGAHLYICGDGSK

MAPDVEDTLCQAYQEIHQASEQEARNWLDRLQEEGRYGKDVWAGI

>CYP106B1 *Bacillus cereus biovar anthracis* CI

MASPENVILVHEISKLKTKEELWNPYEWYQFMRDNHPVHYDDEQDVWNVFLYDDVNRVLS

NYSLFSSRRERRQFAIPPLETRININSTDPPEHRNVRSIVSKAFTPRSLEQWKPRIQSIA

NELVKDIENCSEVDIVEQFAAPLPVTVISDLLGVPTTDRKKIKAWSDILFMPYSKEKFND

LDAEKGIALNEFKAYLLPIVQEKRYHLTDDIISDLIRAEYEGERLTDEEIVTFSLGLLAA

GNETTTNLIINSFYCFLVDSPATYKEVREKPKLISKAVEEVLRYRFPVTLARRITEDTNI

FGPLMKKDQMVVAWVSAANLDEKKFSQASKFNIHRIGNEKHLTFGKGPHFCLGAPLARLE

AEIALTTFINAFEKIALSPSFNIEQCILENEQTLKFLPIRLKAQ

>CYP107J2 *Bacillus cereus biovar anthracis* CI

MSMKNKVGLRIEDGINLASAQFKEDAYEIYKESRKMQPILFVNKTELGAEWLITRYEDAL

PLLKDNRLKKDPANVFSQDTLNVFLTVDNSDYLTTHMLNSDPPNHNRLRSLVQKAFTPKM

IAQLEGRIQHIADDLLNEVERKGSLNLVDDYSFPLPIIVISEMLGIPKEDQAKFRIWSHA

VIAYPETPEEIKETEKQLSEFITYLQYLVDMKRKEPKEDLVSALILAESEGHKLSARELY

SMIMLLIVAGHETTVNLITNTVLALLENPNQLRLLKENPKLIDAAIEEGLRYYSPVEVTT

SRWADEPFQIHDQTIEKGDMVVIALASANRDETVFENPEVFDITRENNRHIAFGHGSHFC

LGAPLARLEAKIAITTLFERMPELQIKGNREDIKWQGNYLMRSLEELPLTF

>CYP109T2 *Bacillus cereus biovar anthracis* CI

MKTPYQSVVMIPTNKLMGKKALEDPYKPFAWYKEMREKEPICFNHQADMWNVFLYEDVKT

VLEDKEYFSSIMPEKKKSPFPQSILGMDPPRHTQIRSIVNRSFTPKALREWEPRIQQITN

DILNQLSNRETFDIVRELFYPLPVIVIAEMLGVSAKDMERFKRWSDIIVSSPSHDDSDYL

EEFFNTRLQAENELGEFFEEIIQSNRGKSKKDSNNIISLLVQSEADKNISRKEIVPFCKL

LLVAGNETTTNLLGNALYCFIEHPNVYDQLQQDISLVPKAIEEVLRYRSPVQRIVRRVKK

EIQLKGQTLQVDQIISAWVGSANRDSHQFKDADSFNIYRKRNPHLTFGHGIHFCLGAPLA

RLEAKIVLTELIKRYKSFSFIDQDLPVPISNSSSIYGLHSFPVKSELN

>CYP102A8 *Bacillus cereus* E33L

MDKKVSAIPQPKTYGPLGNLPLIDKDKPTLSFIKIAEEYGPIFRMQTLSDAIIVVSGHEL

VAEVCDETRFDKSIEGALAKVRAFAGDGLFTSETDEPNWKKAHNILMPTFSQRAMKDYHA

MMVDIAVQLVQKWARLNPNENVDVPGDMTRLTLDTIGLCGFNYRFNSFYRETPHPFITSM

TRALDEAMHQLQRLDIEDKLMWRTKRQFQHDIQSMFSLVDNIIAERKSSGNQEENDLLSR

MLNVQDPETGEKLDDENIRFQIITFLIAGHETTSGLLSFTIYFLLKNPDKLKKAYEEVDR

VLTDPTPTYQQVMKLKYIRMILNESLRLWPTAPAFSLYAKEDTVIGGKYPIKKGEDRISV

LIPQLHRDKDAWGDNVEEFQPERFEELDKVPHHAYKPFGNGQRACIGMQFALHEATLVMG

MLLQHFEFIDYEDYQLGVKQTLTLKPGDFKIRIVPRNQTISHNTVLAPTEEKLKNHEIKQ

KVQKIPSIIGADNLSLLVLYGSDTGVAEGIARELADTASLEGVQTEVAALNDRIGSLPKE

GAVLIVTSSYNGKPPSNAGQFVQWLEELKGDELKGVQYAVFGCGDHNWASTYQRIPRYID

EQMAQKGATRFSTRGEADASGDFEEQLEQWKESMWSDAMKAFGLELNKNIEKERSTLSLQ

FVSRLGGSPLARTYEAVYASILENRELQSSSSERSTRHIEISLPEGATYKEGDHLGVLPI

NSEKNVNRILKRFGLNGKDQVILSASGRSVNHIPLDSPVRLYDLLSYSVEVQEAATRAQI

REMVTFTACPPHKKELESLLEDGVYHEQILKKRISMLDLLEKYEACEIRFERFLELLPAL

KPRYYSISSSPLVAQNRLSITVGVVNAPAWSGEGTYEGVASNYLAQLHNKDEIICFIRTP

QSNFQLPENPETPIIMVGPGTGIAPFRGFLQARRVQKQKGMKVGEAHLYFGCRHPEKDYL

YRTELENDERDGLISLHTAFSRLEGHPKTYVQHVIKQDRIHLISLLDNGAHFYICGDGSK

MAPDVEDTLCQAYQEIHEVSEQEARNWLDRLQEEGRYGKDVWAGI

>CYP106B1 *Bacillus cereus* E33L

MASPENVILVHEISKLKTKEELWNPYEWYQFMRDNHPVYYDEEQDVWNVFLYEDVNRVLS

DYRLFSSRRERRQFSIPPLETRININSTDPPEHRNVRSIVSKAFTPRSLEQWKPRIQAIA

DELVKDIEKFSEVDIVEQFAASLPVTVISDLLGVPTTDRKKIKAWSDILFMPYSKEKFND

LDAEKGIALNEFKAYLLPIVQEKRYLLTDDIISDLIRAEYEGERLTDEEIVIFSLGLLAA

GNETTTNLIINSFYCFLMDSPGTYKELRKEPKLISKAIEEVLRYRFPVTLARRITEDTNI

FGPLMKKDQMVVAWVSAANLDEKKFAQASKFNIHRIGNEKHLTFGKGAHFCLGAPLARLE

AEIALSTFINAFEKIELSSSFDLEKCILKNEQTLKFLPICLKTQ

>CYP107J2 *Bacillus cereus* E33L

MAMKNKVGLRIEDGINLASAQFKEDAYEIYKESRKVQPVLFVNKTELGAEWLITRYEDAL

PLLKDSRLKKDPANVFSQDTLNVFLTVDNGDHLTTHMLNSDPPNHNRLRSLVQKVFTPKM

IAQLEGRIQDITDDLLNEVERKGSLNLVDDYSFPLPIIVISEMLGIPKEDQAKFRIWSHA

VIAYPETPEEIKETEKQLSEFITYLQYLVDMKRKEPTEDLVSALILAESEGHKLSARELY

SMIMLLIVAGHETTVNLITNTVLALLENPNQLQLLKENPKLIDAAIEEGLRYYSPVEVTT

SRWADEPFQIHDQTIEKGDMVVIALASANRDETVFENPEVFDITRENNRHIAFGHGSHFC

LGAPLARLEAKIAITTLFERMPELQIKGNREDIKWQGNYLMRSLEELPLTF

>CYP109T2 *Bacillus cereus* E33L

MLYMKTPYQSVVMIPTNKLMGKKALEDPYKPFAWYKEMREKEPICFNHQADMWNVFLYED

VKTVLEDKEYFSNIMPEKKKSPFSQSILGMDPPKHTQIRSIVNRSFTPKALREWEPRIQQ

ITNDILNQLSNRKTFDIVRELFYPLPVIVIAEMLGVSAKDMERFKRWSDIIVSSPSHDDP

DYLVEFFNIRLQAENELGEFFEEIIQFNRGKSKQDLNNIISLLVQSEADENISGKEIVPF

CKLLLVAGNETTTNLLGNALYCFIEHPNVYDQLQQDISLIPKAIEEVLRYRSPVQRIVRR

VKKEIQLKGQTLQTDQIISAWVGSANRDSQQFKDADSFNIYRRRNPHLTFGHGIHFCLGA

PLARLEAKIVLTELIKRYKSFSFIDQNLPVPISNSSSIYGLHSFPVKSELN

>CYP102A8 *Bacillus cereus* FT9

MKDYHAMMVDIAVQLVQKWARLNPNENVDVPGDMTRLTLDTIGLCGFNYRFNSFYRETSH

PFITSMSRALDEAMHQLQRLDIEDKLMWRTKRQFQHDIQSMFSLVDNIIAERKSSGNQEE

NDLLSRMLNVQDPETGEKLDDENIRFQIITFLIAGHETTSGLLSFAIYFLLKNPDKLKKA

YEEVDRVLTDPTPTYQQVMKLKYIRMILNESLRLWPTAPAFSLYAKEDTVIGGKYPIKKG

EDRISVLIPQLHRDKDAWGDNVEEFQPERFEELDNVPHHAYKPFGNGQRACIGMQFALHE

ATLVMGMLLQHFEFIDYEDYQLDVKQTLTLKPGDFKIRIVPRNQTISHTTVLAPTEEKLK

NHETKQQVQKTPSIIGADNLSLLVLYGSDTGVAEGIARELADTASLEGVQTEVAALNDRI

GSLPKEGAVLIVTSSYNGKPPSNAGQFVQWLEELKPDELKGVQYAVFGCGDHNWASTYQR

IPRYIDEQMAQKGATRFSKRGEADASGDFEEQLEQWKQSMWSDAMKAFGLELNKNMEKER

STLSLQFVSRLGGSPLARTYEAVYASILENRELQSSSSERSTRHIEISLPEGATYKEGDH

LGVLPINSEKNVNRILKRFGLNGKDQVILSASGRSVNHIPLDSPVRLYDLLSYSVEVQEA

ATRAQIREMVTFTVCPPHKKELESLLEEGVYQEQILKKRISMLDLLEKYEACEIRFERFL

ELLPALKPRYYSISSSPLVAQDRLSITVGVVNAPAWSGEGTYEGVASNYLAQRHNKDEII

CFIRTPQSNFQLPENPETPIIMVGPGTGIAPFRGFLQARRVQKQKGMKVGEAHLYFGCRH

PEKDYLYRTELENDEREGLISLHTAFSRLEGHPKTYVQHVIKEDRIHLISLLDNGAHLYI

CGDGSKMAPDVEDTLCQAYQEIHEVSEQEARNWLDRVQEEGRYGKDVWAGI

>CYP106B1 *Bacillus cereus* FT9

MATPENVILVHEISKLKTKEELWNPYEWYQFMRDNHPVYYDEEQDVWNVFLYEDVNRVLS

DYRLFSSRRERRQFSIPPLETRININSTDPPEHRNVRSIVSKAFTPRSLEQWKPRIQAIA

NELVQHIGKYSEVNIVEEFAAPLPVTVISDLLGVPTTDRKKIKAWSDILFMPYSKEKFND

LDAEKGIALNEFKAYLLPIVQEKRYLLTDDIISDLIRAEYEGERLTDEEIVTFSLGVLAA

GNETTTNLIINSLYCFLVDSPGTYKELRKEPKLISKAIEEVLRYRFPVTLARRITEDTNI

FGPLMKKDQMVVAWVSAANLDEKKFAQASKFNIHRIGNEKHLTFGKGAHFCLGAPLARLE

AEIALSTFINAFEKIELSSSFDLEKCILENEQTLKFLPICLKTQ

>CYP107DY3 *Bacillus cereus* FT9

MKKLTFNDLNSPETMRNPIMFYKNLMEQKERFFHIDDFYGMGGAWVVFHYDDVVAILKDS

RFIKDLRKFTPPHYKQNPIEENTAVSKLFEWLMNMPNMLTVDPPDHTRLRRLVSKSFTPR

MIEDLRPRIQQIADELLDVVQEQRKMEIIADFAYPLPIIVISEMLGIPATDRNQFRAWTQ

ELMKASVDPGQGTTVTATLEKFINYIEILFNEKHLNPSDDLISALVQAKEQEDKLSKNEL

LSTIWLLIIAGHETTVNLISNGVLALLQHPEQMNLLRQDPSLLASAVDELLRYAGPIMFS

SRFASEDVTIHGNRIRKGELVLLSLTAANIDPNIFPYPEELNISREENNHLAFGAGIHQC

LGAPLARLEGQIALDTLLKRLPNLRLAIEADQLIYNHSKIRSLASLPVIF

>CYP109T4 *Bacillus cereus* FT9

MKTPYQSIDMIPTSKLIGKKALEDPYKPFAWYKEMREKEPICFNHQADMWNVFLYEDVKT

VLEDKEHFSNIMPEKKKSPFPQSILGMDPPKHTQIRSIVNRSFTPKSLREWEPRIQQITN

DILNQLSNRKTFDIVRELFYPLPVIVIAEMLGVSAKDMERFKKWSDIIVSSPSHDDPDYL

AEFFHTRLQAENELGDFFEEIIQLNREKSQKDANDIISLLVQSEAEKSISGKELVSFCKL

LLVAGNETTTNLLGNALYCFIEYPNVYEQLQQDISLIPKAIEEVLRYRSPVQRITRRVKK

EIQLKYQTLQVDQIISAWVGSANRDSHQFTDGDSFNIYRGRNPHLTFGHGIHFCLGAPLA

RLEAKIVLTELIKRYKSFSFIDENLPTPISNSSTIYGLSSFPVKSELLHIK

>CYP107J8 *Bacillus cytotoxicus*

MSVKNKVGRNIKDGIKLASPEFKEYAYEIYKESRKLQPILFVYKGELGTEWLITRYEDAL

SLLKDSRLKKNPENIFSQEKLKSLFSIENSDYLTKHMLNADPPDHNRLRALVQKAFTPKM

ISQLHNRIQHIADTLLDHVEQKRSFHFIRDFSFPLPIIVISEMLGIPKEDQAKFRIWSHA

VIDSPEIPEEKIENEEKLSEFITYLQYLVDLKRKEPKEDLISRLIQAESEGGQLSAAELY

SMIMLLIVAGHETTVNLMTNTVLALLENPDQLQLLKERPELIDSAIEEGLRYYSPVEVTT

ARWAAEPFHIHDKVIQKGDMVTISLASANRDETVFVNPDVFNITRENNRHIAFGHGGHFC

LGAPLARLEAKIAIATLLKRMPRLRIKGKREDIKWEGNYLMRSLKELPLTF

>CYP109T3 *Bacillus cytotoxicus*

MKTPYQSIDMIPTNKLIGKQALEEPYKPFVWYKEMREKEPICFNHQADMWNVFLYEDVKT

VLEDKEYFSNIMPEKKKSPFPQSILGMDPPKHTQMRSIVNRSFTPKALREWEPRIQQITN

DILNQLSNHKTFDIVRELFYPLPVIVIAEMLGVSAKDMERFKKWSDIIVSSPSHEDPDYL

KEFFHTRLQAENELGNFFEEIIQLNRGKAKKDSNDIISLLVQSEADINISSKELVAFCKL

LLVAGNETTTNLLGNALYCLIEHPNVYEQLQQDLSLIPKTIEEVLRYRSPVQRVVRRVKK

EIQLKGQTLQVDQIVSAWVGSANRDSYQFKDADSFNIYRRRNPHLTFGHGIHFCLGAPLA

RLEAKIVLTELIKRYKSFSFIDQNLPTPISNSSTIYGLSSFPVKSELLHIK

>CYP109T6 *Bacillus cytotoxicus*

MKKTYESIVMIPTNKLIEKKALENPYEPFAWYKEMREKEPICFNPQADMWNVFLYDDVKI

VLEDKEHFSKIMPEKKTLPFSKSILGMNPPKHTQIRSIVNRSFTPKVLEAWEPRIQKVTE

DILIQLSNRETFDIVQELFYPLPVIVTAEMLGVSANDMERFKKWSDSILSSPNHDDQDYL

TEFFHMRLQAENELGEFFEEILQIKRGNSKENSNDIISLLVQNEADNKISGEEIVPFCKL

LLVAGNETTTNLLGNALYCLIEHPNVYEQLQQDLSLIPKVIEEVLRYRSPAQRIVRRVKK

GMQLNGQTLKVNQIVSAWIGSANRDSQYFNDADSFNIHRPRNPHLGFGYGINFCLGTRLA

RLVATIVLTEIIKKYKSFSFIDHNLPIPISNSRSLYGLKTFPVKSEIIHIE

>CYP109X1 *Bacillus cytotoxicus*

MENFKMKNKGLGLVKPVKELILEQAKLDPFTWFKEMRNNTPIRYDEERGCWDVFNYNDVL

NILKDYNNFSSDRPEPVLVSSIIRMDPPRHTQMRGIISNAFTPRLIKDLEPRIQDIAKIL

INETLPNEEMEVIQDFSYALAIIVIADLLGVPSEDHYLFKKWSDIIAKGANDDSPNALRE

VIREKNEVREELNVYFSKIVSRRKENPKNDLITKLVEARIEGEGLTQIEILEFCHLLLVA

GNETTTNLIANLIRRIAEDDNLENQLRLNPHLIKNAIEETLRFYPPVLNTSRFAANDFNL

RGHQIKKGDQVILWIASANRDEKQFKNPDTFDINRVSIKHLTFGQSIHFCLGAPLARLEA

EIAIQTLLKMVRDIKFSNSKLNPIQSCLVYGCTALRIKFKVSE

>CYP102A42 *Bacillus simplex*

MENTTQFPQPKSYGPLGSLPIIDKDKPLQSYMKLARELGPVFQFQFPGRISTFVSSASLA

KEICDETRFDKKVGPSLQKVRAFGGDGLFTSETAEPNWKKAHNILLPSFSQQAMKGYHAK

MVDLATQLIQKWTRVNPADVIDVPEDMTRLTLDTIGLCGFNYRFNSFYRETTHPFVTSMV

RALDEAMSQTQRLGIQDKLMVKSKKQFREDIQYMFSLVDEMIAERKQNGDQGEDDLLSHM

LKGVDPETGVSLDDENIRFQIITFLIAGHETTSGLLSFAIYFLMNNRDKLKKAQQEVDEV

LGDDVPDYKQVKKLKYVRMVLNEVLRLWPTAPAFSVYAKEDTILDEKYTVKKGDVFTLLI

PELHRDPSVWGDDVESFIPERFENLDSIPYHAYKPFGNGQRACIGQQFALHEATLVLGMV

LQHFDLIDHEDYQLDVKETLTLKPDGLTMRVSPRKPAMSFTVASPEPKSADKGATASSIE

SAHGTPLLVLFGSNMGTAEGIARDLAETGKLQGFNARVAPLNDYTNRLPQEGAVLIVSAS

YNGNPTDNADDFVSWLKESHDATLDGVHYAVFGCGDRNWANTYQRIPIFIDERLEQKGAV

RLSETGYGDASDDFEGDYEKWTEALWPNLAETLNIEVNMNERLVSSITMNFVSNVSGTPL

ARTHHAFTSIVKRNLELQHVESGRSTRHIELTVPEGIYYKEGDHLGVLPQNPSELVERVL

SRFSLNGQDYVNLTGDSGKAAHLPTGKPIKLEELLSNHVEFQEPATRSQIRALATHTVCP

PHVKELENLLEDSTYKREILNKRMTMLDLTETYLACEIPFERFLALLPPLKARYYSISSS

PLHKEGEASITVSVVRGKALSGNGEYKGIASNYLAERSEGDKVACFINTPQSNFQLPEQT

EKPIIMIGPGTGIAPFRGFIQARRALKKKGETLGSAHLYFGCRNPEHDFLYQEELVQAEH

EGLVTLHTAFSRCPGQEKTYVQNRLAENAQDILPLLKEGGHLYICGDGSKMAPDVERTLI

DSYMHFYQTTKEEATEWLQSLEENGRYAKDVWAGA

>CYP106A2 *Bacillus simplex*

MMKEVIAIKEFTRFKTRTEEFSPYAWCKRMLENDPVSYHEGTDTWNVFKYEDVKRVLSDY

KHFSSVRKRTTISVGTDSGEGAVPEKIKITESDPPEHRKRRSLLAAAFTPRSLQNWEPRI

QEIADELIGQMDEETEIDIVQSLASPLPIIVMSDLMGVPSKDRLLFKKWVDILFLPFDKE

KQEEVNELKQVAAKEYYQYLYPIVVQKRLNPADDIISDLLKSEVDGEMFTDDEVVRTTML

ILGAGVETTSHLLANSFYSLLYDDVEVYQELHENLDLVPQAVEEMLRFRFNLIKLDRTVQ

EDNDLLGVELKKGENVVVWMSAANLDEAMFEDAFTLNIHRPNNKKHLTFGNGPHFCLGAP

LARLEAKIALTTFLKKFKHIEAVPTFQVEENLTDSATGQTLTSLPLKARRTH

>CYP109A9 *Bacillus simplex*

MTQEQKSTSAVEQYANLIPMREIDSNADQLFPFPIFNDLRQKSPVRYDESRSCWDVFRYE

DVHFILKNPKMFSSERGSGTLQGSILTMDPPRHTKMRNLVNKAFTPKAVKDLTNKIEEVT

LYLLDQVKEKGTMDLVHDVAGPLPVIIIAELLGIPTKDRDLFKTYSDVLVEGAKDNSSEA

FQRMTQKRKEGNEFLKEYFKKIIKERTNNPEEDLISLLIEAKIDGEKLTEEELLSFCVLL

LVAGNETTTNLITNAVRYMTEDKKIQEQARTNLPLIPKLVEETLRFYPPIQAIGRIAKEH

VKVGGKTIQKGEQVICWVASANRDEQKFAEPNRFTLERKLNPHLSFGFGIHFCLGAPLAR

LEAEVALVTLLSTFSKLEDVKGTKLEAIPSPFVFGVKSLPLKFTR

>CYP1221A8 *Bacillus simplex*

MSDTDFSHDSPKGPKGRLITGHSKEFISDTLGFLTHLAKEYGDVAKIRFGPFQNVYFISN

PDLIKQVLVTKQKSFLKSKDFHALKPLLGEGLLTSEKEIHMRQRRLIQPSFKKSHISNYA

QDMIDITMNYISDWKNREERIITEDMMSLALGIISKTMFSMNLKEGYDELGEPIEASMRI

AVKRMRTLLQLPLWVPTKNNREFKNSIQRLDTVIYNFIEKRRADAERHEDMLGILMDARD

DEDGLAMTNQQVRDELMTIFLAGHETTANVLSWTLYLLSQHPEVETKLFNEIDSVIGNRN

PTPGDYMKLTYTQNIINESMRIYPPGYIVSRKVEEDVVIGGYHFKKGDMLLLSQYVMHHK

PEYFDNPESFNPERFENNFVKTLPPFAYFPFGGGARVCIGNHFAMMEAVLVLACIAQRYR

IKLAPDHHEVKPLPSITLRPKRGLRMIVEDRKESFREPLIDD

>CYP102A36 *Bacillus subtilis* XF-1

MKETSPIPQPKTFGPLGNLPLIDKDKPTLSLIKLAEEQGPIFQIHTPAGTTIVVSGHELV

KEVCDEERFDKSIEGALEKVRAFSGDGLFTSWTHEPNWRKAHNILMPTFSQRAMKDYHEK

MVDIAVQLIQKWARLNPNEAVDVPGDMTRLTLDTIGLCGFNYRFNSYYRETPHPFINSMV

RALDEAMHQMQRLDVQDKLMVRTKRQFRHDIQTMFSLVDSIIAERRSNGDQDEKDLLARM

LNVEDPETGEKLDDENIRFQIITFLIAGHETTSGLLSFATYFLLKHPDKLKKAYEEVDRV

LTDAAPTYKQVLELTYIRMILNESLRLWPTAPAFSLYPKEDTVIGGKFPITTKDRISVLI

PQLHRDRDAWGKDAEEFRPERFEHQDQVPHHAYKPFGNGQRACIGMQFALHEATLVLGMI

LKYFTLIDHENYELDIKQTLTLKPGDFHIRVQSRNQEAIHADVQAAEKAVSDEQKEKTEA

KGASVIGLNNRPLLLLYGSDTGTAEGVARELADTASLHGVRTETAPLNDRIGKLPKEGAV

VIVTSSYNGKPPSNAGQFVQWLQEIKPGELEGVHYAVFGCGDHNWASTYQYVPRFIDEQL

AEKGATRFSARGEGDVSGDFEGQLDEWKKSMWADAIKAFGLELNENADKERSTLSLQFVR

GLGESPLARSYEASHASIAENRELQSADSDRSTRHIEIALPPDVEYQEGDHLGVLPKNSQ

TNVSRILHRFGLKGTDQVTLSASGRSAGHLPLGRPVSLHDLLSYSVEVQEAATRAQIREL

AAFTVCPPHRRELEELSAEGVYQEQILKKRISMLDLLEKYEACDMPFERFLELLRPLKPR

YYSISSSPRVNPRQASITVGVVRGPAWSGRGEYRGVASNDLAERKAGDDVVMFIRTPESR

FQLPEDPETPIIMVGPGTGVAPFRGFLQAREVLKREGKTLGEAHLYFGCRNDRDFIYRDE

LEQFEKDGIVTVHTAFSRKEGMPKTYVQHLMADHAETLISILDRGGRLYVCGDGSKMAPD

VEAALQKAYQSVHGTGEQEVQNWLRHLQDTGMYAKDVWAGI

>CYP102A48 *Bacillus subtilis* XF-1

MLMKQASAIPQPKTYGPLKNLPHLEKEQLSQSLWRIADELGPIFRFDFPGVSSVFVSGHN

LVAEVCDESRFDKNLGKGLQKVREFGGDGLFTSWTHEPNWQKAHRILLPSFSQKAMKGYH

SMMLDIATQLIQKWSRLNPNEEIDVADDMTRLTLDTIGLCGFNYRFNSFYRDSQHPFITS

MLRALKEAMNQSKRLGLQDKMMVKTKLQFQKDIEVMNSLVDRMIAERKANPDEDIKDLLS

LMLYAKDPVTGETLDDENIRYQIITFLIAGHETTSGLLSFAIYCLLTHPEKLKKAQEEAD

RVLTDDTPEYKQIQQLKYIRMVLNETLRLYPTAPAFSLYAKEDTVLGGEYPISKGQPVTV

LIPKLHRDQNAWGPDAEDFRPERFEDPSSIPHHAYKPFGNGQRACIGMQFALQEATMVLG

LVLKHFELINHTGYELKIKEALTIKPDDFKITVKPRKTAAINVQRKEQADIKAETKPKET

KPIHGTPLLVLYGSNLGTAEGIAGELAAQGRQMGFAAETAPLDDYIGKLPEEGAVVIVTA

SYNGAPPDNAAGFVEWLKELKEGQLKGVSYAVFGCGNRSWASTYQRIPRLIDDMMKAKGA

SRLTEIGEGDAADDFESHRESWENRFWKETMDAFDINEIAQKEDRPSLSITFLSETTETP

VAKAYGAFEGVVLENRELQTADSTRSTRHIELEIPAGKTYKEGDHIGIMPKNSRELVQRV

LSRFGLQSNHVIKVSGSAHMAHLPMDRPIKVVDLLSSYVELQEPASRLQLRELASYTVCP

PHQKELEQLVSDDGIYKEQVLAKRLTMLDFLEDYPACEMPFERFLALLPSLKPRYYSISS

SPKVHANIVSMTVGVVKASAWSGRGEYRGVASNYLAELNTGDAAACFIRTPQSGFQMPDE

SETPMIMVGPGTGIAPFRGFIQARSVLKKEGSTLGEALLYFGCRRPDHDDLYREELDQAE

QDGLVTIRRCYSRVENEPKEYVQHLLKQDTQKLMTLIEKGAHIYVCGDGSQMAPDVEKTL

RLAYEAEKGASQEESAEWLQKLQDQKRYVKDVWTGM

>CYP107K1 *Bacillus subtilis* XF-1

MQMEKLMFHPHGKEFHHNPFSVLGRFREEEPIHRFELKRFGATYPAWLITRYDDCMAFLK

DNRITRDVKNVMDQEQIKMLNVSEDIDFVSDHMLAKDTPDHTRLKSLVHQAFTPRTIENL

RGSIEQIAEQLLDEMEKENKADIMKSFASPLPFIVISELMGIPKEDRSQFQIWTNAMVDT

SEGNRELTNQALREFKDYIAKLIHDRRIKPKDDLISKLVHAEENGSKLSEKELYSMLFLL

VVAGLETTVNLLGSGTLALLQHKKECEKLKQHPEMIATAVEELLRYTSPVVMMANRWAIE

DFTYKGHSIKRGDMIFIGIGSANRDPNFFENPEILNINRSPNRHISFGFGIHFCLGAPLA

RLEGHIAFNALLKRFPDIELAVAPEDIQWRKNVFLRGLESLPVSLSK

>CYP109B1 *Bacillus subtilis* XF-1

MNVLNRRQALQRALLNGKNKQDAYHPFPWYESMRADAPVSFDEENQVWSVFLYDDVKKVV

GDKELFSSYMPQQTSSIGNSIINMDPPKHTKIRSVVNKAFTPRVMKQWEPRIQEITDELI

QKFQERSEFDLVHDFSYPLPVIVISELLGVPSKHMDQFKAWSDLLVSTPKDKSEEAEKAF

LEERDKCEEELAAFFAGIIEEKRNKPAQDIISILVEAEETGKKLSGEELIPFCTLLLVAG

NETTTNLISNAMYSILETPGVYEELRSHPELMPQAVEEALRFRAPAPVLRRIAKRDTKIG

GHLIKEGDMVLAFVASANRDEAKFDRPHMFDIRRHPNPHIAFGHGIHFCLGAPLARLEAN

IALTSLISAFPHMECVSITPIENSVIYGLKSFRVKM

>CYP102A5 *Bacillus thuringiensis* YBT-1518

MEKKVSAIPQPKTYGPLGNLPLIDKDKPTLSFIKIAEEYGPIFQIQTLSDTIIVVSGHEL

VAEVCDETRFDKSIEGALAKVRAFAGDGLFTSETHEPNWKKAHNILMPTFSQRAMKDYHA

MMVDLAVQLVQKWARLNPNENVDVPEDMTRLTLDTIGLCGFNYRFNSFYRETPHPFITSM

SRALDEAMHQLQRLDIEDKLMWRTKRQFQHDIQSMFSLVDNIIAERKSSGNQEENDLLSR

MLNVQDPETGEKLDDENIRFQIITFLIAGHETTSGLLSFSIYFLLKNPDKLKKAYEEVDR

VLTDPTPTYQQVMKLKYIRMILNESLRLWPTAPAFSLYAKEDTVIGGKYPIKKGEDRISV

LIPQLHRDKDAWGDNVEEFQPERFEELDKVPHHAYKPFGNGQRACIGMQFALHEATLVMG

MLLQHFELIDYQNYQLDVKQTLTLKPGDFKIRILPRKQTISHPTVLAPTEDKLKNDEIKQ

HVQKTPSIIGADNLSLLVLYGSDTGVAEGIARELADTASLEGVRTEVVALNDRIGSLPKE

GAVLIVTSSYNGKPPSNAGQFVQWLEELKPDELKGVQYAVFGCGDHNWASTYQRIPRYID

EQMAQKGATRFSKRGEADASGDFEEQLEQWKQSMWSDAMKAFGLELNKNMEKERSTLSLQ

FVSRLGGSPLARTYEAVYASILENRELQSSSSDRSTRHIEVSLPEGATYQEGDHLGVLPI

NSEKNVNRILKRFGLNGKDQVILSASGRSINHIPLDSPVSLLDLLSYSVEVQEAATRAQI

REMVTFTACPPHKKELEALLEEGVYHEQILKKRISMLDLLEKYEACEIRFERFLELLPAL

KPRYYSISSSPLVAQNRLSITVGVVNAPAWSGEGTYEGVASNYLAQRHNKDEIICFIRTP

QSNFELPKDPETPIIMVGPGTGIAPFRGFLQARRVQKQKGINLGQAHLYFGCRHPEKDYL

YRTELENDERDGLISLHTAFSRLEGHPKTYVQHLIKQDRINLISLLDNGAHLYICGDGSK

MAPDVEDTLCQAYQEIHEVSEQEARNWLDRVQDEGRYGKDVWAGI

>CYP106B4 *Bacillus thuringiensis* YBT-1518

MASPENVILVHEISKLKTKEELWNPYEWYRLMRDNHPVHYDEEQDVWNVFLYDDVNRVLS

DYRLFSSRRERRQFSIPPLETRININSTDPPEHRNVRSIVSKAFTPRSLGQWKTRIQAIA

DELVQHIEKCSEVNIVEQFAAPLPVTVISDLLGVPTTDRKKIKEWSDILFMPYSKEKFND

LDAEKGIALHEFKAYLLPIVQEKRYHLTDDIISDLIRAEYEGERLTDEEIVTFSLGLLAA

GNETTTNLIINSFYCFLVDSPGIYEELRKEPNLILKAIEEVLRYRFPVTLARRITEDTNI

FGPFMKKNQMIVAWVSAANLDEKKFSQASQFNVHRTGNEKHLTFGKGPHFCLGAALARLE

AEIALTTFINAFEKIELSPSFCLEKCILENEQTLKYLPIRLKAKEKS

>CYP107J3 *Bacillus thuringiensis* YBT-1518

MSMKNKVGLSIEDGINLASAQFKEDAYEIYKESRKKQPILFVNQVEIGKEWLITRYEDAL

PLLKDNRLKKDWTNVFSQDIKNMYLSVDNSDHLTTHMLNSDPPNHSRLRSLVQKAFTPKM

IAQLDGRIERIADDLISDIERKGTLNLVDDYSFPLPIIVISEMLGIPKEDQAKFRIWSHA

VIASPETPEEIKETEKQLSEFITYLQYLVDIKRKEPKEDLVSALILAESEGHKLSARELY

SMIMLLIVAGHETTVNLITNTVLALLENPNQLQLLKDNPKLIDSAIEEGLRYYSPVEVTT

ARWAAEPFQIHHQTIQKGDMVIIALASANRDETVFENPEIFDITRENNRHIAFGHGSHFC

LGAPLARLEAKIAITTLFNRMPELKIKGNREEIKWQGNYLMRSLEELPLTF

>CYP107DY4 *Bacillus thuringiensis* YBT-1518

MKKLTFKDLNSPETMRNPIMFYKNLIKQQERFFQIDDFYGMGGTWVVLHYDDVITILKDA

RFIKDLRKFTSPHDKQHPNSENTAASKLFDWLMNMPNMLTVDPPDHTRLRRLVSKAFTPR

MIEDLRPRIQQIADGLLVAVQEQGKMEIISDFAYPLPIIISEMLGIPVTDRNQFREWTQI

LMNASVNPSQGTAVTTTLEKFIQYIEVLLNEKRLNPDADLISKLVQTKDQEDKLSNNELL

STIWLLIIAGHETTVNLISNGVLALLQHPEQMNLLRENPSLISSTVDELLRYSGPVMFIS

RLASEDITIHGKRIRKGDLVLLSLTAANIDPQKFTYPETLNISREENNHLAFGAGIHHCL

GAPLARLEGQIALGTLLQRLPNLRLAIKPDQLNYNHSKIRSLVNLPVVF

>CYP102A38 *Bacillus velezensis* AS43.3

MKETGPIPQPKTFGPLGNLPLLDKDKPTMSLIKLANEQGPIFQLHTPAGAIIVVSGHELV

KEVCDEERFDKSIEGALEKVRAFSGDGLFTSWTHEPNWRKAHHILMPTFSQRAMKDYHSM

MTDIAVQLIQKWARLNPDEAVDVPADMTRLTLDTIGLCGFNYRFNSYYRETPHPFINSMV

RALDEAMHQMQRLDVQDKLMIRTKRQFHHDIQAMFSLVDSIIAERRSGGRDEKDLLARML

NVEDPETGEKLDDENIRYQIITFLIAGHETTSGLLSFAIYFLLKHPRVLEKAYEEADRVL

TDPVPSYKQVLDLTYIRMILQESLRLWPTAPAFSLYAKEDTVIGGKYPITPKDRISVLIP

QLHRDKDAWGDNAEEFYPERFEHPDRVPHHAYKPFGNGQRACIGMQFALHEATLVLGMIL

QHFTFIDHTDYELDIKQTLTIKPGDFHIRVRPRNKEDVAAALPAAEKAAEDVGKEKRETK

GASIIGLDNRPLLILYGSDTGTAEGVARELADTAGMHGVRTETAPLNDRIGKLPKEGALL

IITSSYNGKPPSNAGQFVQWLEEVKPGELEGVRYAVFGCGDHNWAATYQAVPRLIDEKLA

EKGAERFSSRGEGDVSGDFEGKLDEWKKSMWTDAMKAFGLKLNENAEKERSALGLQFVSG

LGGSPLAQTYEAVYASVAENRELQAPESGRSTRHIEITLPKEAAYHEGDHLGVLPVNSKE

QVSRVLRRFNLNGNDQVLLTASGQSAAHLPLDRPVRLHDLLSSCVELQEAASRAQIREMA

AYTVCPPHKRELEDFLEEGVYQEQILTSRVSMLDLLEKYEACELPFERFLELLRPLKPRY

YSISSSPRKHPGQASITVGVVRGPARSGLGEYRGVASNYLADRGPEDGIVMFVRTPETRF

RLPEDPEKPIIMVGPGTGVAPFRGFLQARAALKKEGKELGEAHLYFGCRNDHDFIYRDEL

EAYEKDGIVTLHTAFSRKEGVPKTYVQHLMAKDAGALISILGRGGHLYVCGDGSKMAPDV

EATLQKAYQSVRETDERQAQEWLLDLQTKGIYAKDVWAGI

>CYP102A49 *Bacillus velezensis* AS43.3

MKQLSAIPQPKTYGPLKNLPHLEKEKLSQSLWKIAEEYGPIFRFEFPSSVGVFVSGRELA

AEVCDEKRFDKNLSKALLKVREFGGDGLFTSWTHEKNWQKAHRILLPSFSQKAMKGYHSM

MLDIAMQLVQKWSRLNPNEEIDVAEDMTRLTLDTIGLCGFHYRFNSFYRDTQHPFITSML

RALQEAMRQSQRHSLQDKLMIKTRHQFQQDIEEMNSLVDRIIAERRENPDENLSDLLALM

LEAKDPVTGERLDDENIRYQIITFLIAGHETTSGLLSFAIYCLLKNKDKLKKAVQEAERV

LTGETPEYKKIQQLTYIRMVLNETLRLYPTAPAFSLYAKEDTVLGGKYPIAKGQPVTILT

PQLHRDKSAWGEDAESFRPERFSDPAAIPADAYKPFGNGQRACIGMQFALQEATMVLGLV

LKHFELIDHTDYELTIKEALTIKPGDFKIRVKNKDVSNHQPVQNHKAEGSGHKEETKEIP

SHGTPLLILYGSNLGTAEGIAEELADIGRSKGFSTETGPLDDYAGKLPVKGAVVIVTASY

NGAPPDNAAGFVKWMETLEDQELKGVSYAVFGCGDRNWAATYQRIPRLIDKVLEEKGAKR

LTSIGEGDNADDFEYSQEAWEDSFWKDIMKAFHIEAAPKQTNKSQLSIEYVSEAAETPIA

KTYKAFEAEVITNKELHTESSKRSVRHIELRLPETETYQEGDHLGVLPQNSGELISRVIR

RFGLDPNQHFKIKGRQLPHLPMDRPVNAPELLASYVELQEPATRAQLRELAAHTVCPPHQ

KELEHLYSDDAAYKENVLKKRMTMLDLLEDYPACELPFERFLELLPSLKARYYSISSSPK

ATSGELSITVGVVTAPAWSGRGEYRGVASNYLAGLQKGDSAVCFIRSPQSGFALPENPKT

PLIMVGAGTGIAPFRGFIQARAAEKMSGNSLGEAHLYFGCRHPEEDDLYKDEFDHAEKNG

LVTVHRAYSRLDQDCKVYVQDVLLREAAQIIALLDQGGHLYICGDGSKMAPAVENVLLQA

YEKVHNTDSKVSLEWLEQLQAEGRYAKDVWAGM

>CYP107K3 *Bacillus velezensis* AS43.3

MSFQKHEGGEIQMEKTMFHPHSPEFHENPFAVLSRFREQDPIHKFELQRFGGTFPAWLIT

RYDDCMAFLKDGRITRDVKRVMPKELIAKLNVSEDIDFVSEHMLAKDPPDHSRLRSLVHQ

GFTPRMIEQLRTGIEQITEELLDEMETKADPDIMRDFAAPLPFIVISELLGIPKEDRAKF

QVWTNAMVDTSESGQDATNQALKEFKQYMKTLIEEKRKHPGEDLTSKLIYAEEDGQKLSE

SELYSMLFLLVVAGLETTVNLLGSGTLALLLHKDQMEKIKRQPENIQTAVEELLRYTSPV

IMMANRWAIEDFTYKDVSIKKGDMIFIGIGSANRDPEYFDDPDTLNIARTPNRHISFGFG

IHFCLGAPLARMEASIAFTALLKRFPNIELKGAAEDVTWRKNVFLRGLKHCLSDFNIAPL

FFGGFFNVL

>CYP113L1 *Bacillus velezensis* AS43.3

MTSLTKIRQQQPYKWYQTMRETSPVHYNEKEDCWEIFTYDEVKRVISDYSHFSSDHKYLS

ADKQEKMIRHINKDSLLKMDPPEHTVFRKLVNQPFMPKSVETLAPRIAAIADDLLQTVRS

KDRMDIIEDYAFPLPIIVIAELLGFPPKDRDIFKSWVDQSQNVKDEKKMNEVQKQMIGYF

MQFILQRRKQPQNDLISHLISADLDGEPLSDKQLIGFCGLLIVAGHVTTENVIGNSFLSL

KEFPHILPRLLENKALLPDFIEEVIRLRPSIQRVTRYTAVESEIGGKTIPAGEKVYAWIG

SANRDEKKFENADQIDLGRKPNQHLSFGQGSHYCLGAPLARLEAKIALSHFFEQVPAWRF

TEDQEPNLVPSPVFHGVDRLLVEF

>CYP102A4 *Bacillus anthracis* A16

MDKKVSAIPQPKTYGPLGNLPLIDKDKPTLSFIKLAEEYGPIFRMQTLSDTIIVVSGHEL

VAEVCDETRFDKSIEGALAKVRAFAGDGLFTSETQEPNWQKAHNILMPTFSQRAMKDYHA

MMVDIAVQLVQKWARLNPNENVDVPEDMTRLTLDTIGLCGFNYRFNSFYRETPHPFITSM

TRALDEAMHQLQRLDIEDKLMWRTKRQFQHDIQSMFSLVDNIIAERKSSENQEENDLLSR

MLNVQDPETGEKLDDENIRFQIITFLIAGHETTSGLLSFAIYFLLKNPDKLKKAYEEVDR

VLTDSTPTYQQVMKLKYIRMILNESLRLWPTAPAFSLYAKEDTVIGGKYPIKKGEDRISV

LIPQLHRDKDAWGDNVEEFQPERFEELDKVPHHAYKPFGNGQRACIGMQFALHEATLVMG

MLLQHFEFIDYEEYQLDVKQTLTLKPGDFKIRIVPRNQTISHTTVLAPTEEKLKNHEIKQ

QVQKTPSIIGADNLSLLVLYGSDTGVAEGIARELADTASLEGVQTEVAALNDRIGSLPKE

GAVLIVTSSYNGKPPSNAGQFVQWLEELKGDELKGVQYAVFGCGDHNWASTYQRIPRYID

EQMAQKGATRFSTRGEADASGDFEEQLEQWKQRMWSDAMKVFGLELNKNMEKERSTLSLQ

FVSRLGGSPLARTYEAVYASILENRELQSSSSERSTRHIEISLPEGATYKEGDHLGVLPI

NSEKNVNRILKRFGLNGKDQVILSASGRSVNHIPLDSPVRLYDLLSYSVEVQEAATRAQI

REMVTFTACPPHKKELESLLEDGVYQEQILKKRISMLDLLEKYEACEIRFEPFLELLPAL

KPRYYSISSSPLVAQDRLSITVGVVNAPAWSGEGTYEGVASNYLAQRHNKDEIICFIRTP

QSNFQLPENPETPIIMVGPGTGIAPFRGFLQARRVQKQKGMNVGEAHLYFGCRHPEKDYL

YRTELENDERDGLISLHTAFSRLEGQAKTYVQHVIKEDRIHLISLLDNGAHLYICGDGSK

MAPDVEDTLCQAYQEIHEVSEQEARNWLDRLQEEGRYGKDVWAGI

>CYP106B1 *Bacillus anthracis* A16

MASPENVILVHEISKLKTKEELWNPYEWYQFMRDNHPVHYDDEQDVWNVFLYDDVNRVLS

DYSLFSSRRERRQFAIPPLETRININSTDPPEHRNVRSIVSKAFTPRSLEQWKPRIQSIA

NELVKDIENCSEVDIVEQFAAPLPVTVISDLLGVPTTDRKKIKAWSDILFMPYSKEKFND

LDAEKGIALNEFKAYLLPIVQEKRYHLTDDIISDLIRAEYEGERLTDEEIVTFSLGLLAA

GNETTTNLIINSFYCFLVDSPATYKEVREKPKLISKAVEEVLRYRFPVTLARRITEDTNI

FGPLMKKDQMVVAWVSAANLDEKKFSQASKFNIHRIGNEKHLTFGKGPHFCLGAPLARLE

AEIALTTFINAFEKIALSPSFNIEQCILENEQTLKFLPIRLKPQ

>CYP107J2 *Bacillus anthracis* A16

MAMKNKVGIRIEDGINLASAQFKEDAYEIYKESRKVQPVLFVNKTELGAEWLITRYEDAL

PLLKDNRLKKDPANVFSQDTLNVFLTVDNSDYLTTHMLNSDPPNHNRLRSLVQKVFTPKM

IAQLEGRIQDIADDLLNEVERKGSLNLVDDYSFPLPIIVISEMLGIPKEDQAKFRIWSHA

VIAYPETPEEIKETEKQLSEFITYLQYLVDMKRKEPKEDLVSALILAESEGHKLSARELY

SMIMLLIVAGHETTVNLITNTVLALLENPNQLQLLKENPKLIDAAIEEGLRYYSPVEVTT

SRWADEPFQIHDQTIEKGDMVVIALAAANRDETVFENPEVFDITRENNRHIAFGHGSHFC

LGAPLARLEAKIAITTLFERMPELQIKGNREDIKWQGNYLMRSLEELPLTF

>CYP102A4 *Bacillus anthracis* Ames

MDKKVSAIPQPKTYGPLGNLPLIDKDKPTLSFIKLAEEYGPIFRMQTLSDTIIVVSGHEL

VAEVCDETRFDKSIEGALAKVRAFAGDGLFTSETQEPNWQKAHNILMPTFSQRAMKDYHA

MMVDIAVQLVQKWARLNPNENVDVPEDMTRLTLDTIGLCGFNYRFNSFYRETPHPFITSM

TRALDEAMHQLQRLDIEDKLMWRTKRQFQHDIQSMFSLVDNIIAERKSSENQEENDLLSR

MLNVQDPETGEKLDDENIRFQIITFLIAGHETTSGLLSFAIYFLLKNPDKLKKAYEEVDR

VLTDSTPTYQQVMKLKYIRMILNESLRLWPTAPAFSLYAKEDTVIGGKYPIKKGEDRISV

LIPQLHRDKDAWGDNVEEFQPERFEELDKVPHHAYKPFGNGQRACIGMQFALHEATLVMG

MLLQHFEFIDYEEYQLDVKQTLTLKPGDFKIRIVPRNQTISHTTVLAPTEEKLKNHEIKQ

QVQKTPSIIGADNLSLLVLYGSDTGVAEGIARELADTASLEGVQTEVAALNDRIGSLPKE

GAVLIVTSSYNGKPPSNAGQFVQWLEELKGDELKGVQYAVFGCGDHNWASTYQRIPRYID

EQMAQKGATRFSTRGEADASGDFEEQLEQWKQRMWSDAMKVFGLELNKNMEKERSTLSLQ

FVSRLGGSPLARTYEAVYASILENRELQSSSSERSTRHIEISLPEGATYKEGDHLGVLPI

NSEKNVNRILKRFGLNGKDQVILSASGRSVNHIPLDSPVRLYDLLSYSVEVQEAATRAQI

REMVTFTACPPHKKELESLLEDGVYQEQILKKRISMLDLLEKYEACEIRFEPFLELLPAL

KPRYYSISSSPLVAQDRLSITVGVVNAPAWSGEGTYEGVASNYLAQRHNKDEIICFIRTP

QSNFQLPENPETPIIMVGPGTGIAPFRGFLQARRVQKQKGMNVGEAHLYFGCRHPEKDYL

YRTELENDERDGLISLHTAFSRLEGQAKTYVQHVIKEDRIHLISLLDNGAHLYICGDGSK

MAPDVEDTLCQAYQEIHEVSEQEARNWLDRLQEEGRYGKDVWAGI

>CYP106B1 *Bacillus anthracis Ames*

MASPENVILVHEISKLKTKEELWNPYEWYQFMRDNHPVHYDDEQDVWNVFLYDDVNRVLS

DYSLFSSRRERRQFAIPPLETRININSTDPPEHRNVRSIVSKAFTPRSLEQWKPRIQSIA

NELVKDIENCSEVDIVEQFAAPLPVTVISDLLGVPTTDRKKIKAWSDILFMPYSKEKFND

LDAEKGIALNEFKAYLLPIVQEKRYHLTDDIISDLIRAEYEGERLTDEEIVTFSLGLLAA

GNETTTNLIINSFYCFLVDSPATYKEVREKPKLISKAVEEVLRYRFPVTLARRITEDTNI

FGPLMKKDQMVVAWVSAANLDEKKFSQASKFNIHRIGNEKHLTFGKGPHFCLGAPLARLE

AEIALTTFINAFEKIALSPSFNIEQCILENEQTLKFLPIRLKPQ

>CYP107J2 *Bacillus anthracis Ames*

MAMKNKVGIRIEDGINLASAQFKEDAYEIYKESRKVQPVLFVNKTELGAEWLITRYEDAL

PLLKDNRLKKDPANVFSQDTLNVFLTVDNSDYLTTHMLNSDPPNHNRLRSLVQKVFTPKM

IAQLEGRIQDIADDLLNEVERKGSLNLVDDYSFPLPIIVISEMLGIPKEDQAKFRIWSHA

VIAYPETPEEIKETEKQLSEFITYLQYLVDMKRKEPKEDLVSALILAESEGHKLSARELY

SMIMLLIVAGHETTVNLITNTVLALLENPNQLQLLKENPKLIDAAIEEGLRYYSPVEVTT

SRWADEPFQIHDQTIEKGDMVVIALAAANRDETVFENPEVFDITRENNRHIAFGHGSHFC

LGAPLARLEAKIAITTLFERMPELQIKGNREDIKWQGNYLMRSLEELPLTF

>CYP102A4 *Bacillus anthracis* CDC 684

MDKKVSAIPQPKTYGPLGNLPLIDKDKPTLSFIKLAEEYGPIFRMQTLSDTIIVVSGHEL

VAEVCDETRFDKSIEGALAKVRAFAGDGLFTSETQEPNWQKAHNILMPTFSQRAMKDYHA

MMVDIAVQLVQKWARLNPNENVDVPEDMTRLTLDTIGLCGFNYRFNSFYRETPHPFITSM

TRALDEAMHQLQRLDIEDKLMWRTKRQFQHDIQSMFSLVDNIIAERKSSENQEENDLLSR

MLNVQDPETGEKLDDENIRFQIITFLIAGHETTSGLLSFAIYFLLKNPDKLKKAYEEVDR

VLTDSTPTYQQVMKLKYIRMILNESLRLWPTAPAFSLYAKEDTVIGGKYPIKKGEDRISV

LIPQLHRDKDAWGDNVEEFQPERFEELDKVPHHAYKPFGNGQRACIGMQFALHEATLVMG

MLLQHFEFIDYEEYQLDVKQTLTLKPGDFKIRIVPRNQTISHTTVLAPTEEKLKNHEIKQ

QVQKTPSIIGADNLSLLVLYGSDTGVAEGIARELADTASLEGVQTEVAALNDRIGSLPKE

GAVLIVTSSYNGKPPSNAGQFVQWLEELKGDELKGVQYAVFGCGDHNWASTYQRIPRYID

EQMAQKGATRFSTRGEADASGDFEEQLEQWKQRMWSDAMKVFGLELNKNMEKERSTLSLQ

FVSRLGGSPLARTYEAVYASILENRELQSSSSERSTRHIEISLPEGATYKEGDHLGVLPI

NSEKNVNRILKRFGLNGKDQVILSASGRSVNHIPLDSPVRLYDLLSYSVEVQEAATRAQI

REMVTFTACPPHKKELESLLEDGVYQEQILKKRISMLDLLEKYEACEIRFEPFLELLPAL

KPRYYSISSSPLVAQDRLSITVGVVNAPAWSGEGTYEGVASNYLAQRHNKDEIICFIRTP

QSNFQLPENPETPIIMVGPGTGIAPFRGFLQARRVQKQKGMNVGEAHLYFGCRHPEKDYL

YRTELENDERDGLISLHTAFSRLEGQAKTYVQHVIKEDRIHLISLLDNGAHLYICGDGSK

MAPDVEDTLCQAYQEIHEVSEQEARNWLDRLQEEGRYGKDVWAGI

>CYP106B1 *Bacillus anthracis* CDC 684

MASPENVILVHEISKLKTKEELWNPYEWYQFMRDNHPVHYDDEQDVWNVFLYDDVNRVLS

DYSLFSSRRERRQFAIPPLETRININSTDPPEHRNVRSIVSKAFTPRSLEQWKPRIQSIA

NELVKDIENCSEVDIVEQFAAPLPVTVISDLLGVPTTDRKKIKAWSDILFMPYSKEKFND

LDAEKGIALNEFKAYLLPIVQEKRYHLTDDIISDLIRAEYEGERLTDEEIVTFSLGLLAA

GNETTTNLIINSFYCFLVDSPATYKEVREKPKLISKAVEEVLRYRFPVTLARRITEDTNI

FGPLMKKDQMVVAWVSAANLDEKKFSQASKFNIHRIGNEKHLTFGKGPHFCLGAPLARLE

AEIALTTFINAFEKIALSPSFNIEQCILENEQTLKFLPIRLKPQ

>CYP107J2 *Bacillus anthracis* CDC 684

MAMKNKVGIRIEDGINLASAQFKEDAYEIYKESRKVQPVLFVNKTELGAEWLITRYEDAL

PLLKDNRLKKDPANVFSQDTLNVFLTVDNSDYLTTHMLNSDPPNHNRLRSLVQKVFTPKM

IAQLEGRIQDIADDLLNEVERKGSLNLVDDYSFPLPIIVISEMLGIPKEDQAKFRIWSHA

VIAYPETPEEIKETEKQLSEFITYLQYLVDMKRKEPKEDLVSALILAESEGHKLSARELY

SMIMLLIVAGHETTVNLITNTVLALLENPNQLQLLKENPKLIDAAIEEGLRYYSPVEVTT

SRWADEPFQIHDQTIEKGDMVVIALAAANRDETVFENPEVFDITRENNRHIAFGHGSHFC

LGAPLARLEAKIAITTLFERMPELQIKGNREDIKWQGNYLMRSLEELPLTF

>CYP102A4 *Bacillus anthracis* HYU01

MDKKVSAIPQPKTYGPLGNLPLIDKDKPTLSFIKLAEEYGPIFRMQTLSDTIIVVSGHEL

VAEVCDETRFDKSIEGALAKVRAFAGDGLFTSETQEPNWQKAHNILMPTFSQRAMKDYHA

MMVDIAVQLVQKWARLNPNENVDVPEDMTRLTLDTIGLCGFNYRFNSFYRETPHPFITSM

TRALDEAMHQLQRLDIEDKLMWRTKRQFQHDIQSMFSLVDNIIAERKSSENQEENDLLSR

MLNVQDPETGEKLDDENIRFQIITFLIAGHETTSGLLSFAIYFLLKNPDKLKKAYEEVDR

VLTDSTPTYQQVMKLKYIRMILNESLRLWPTAPAFSLYAKEDTVIGGKYPIKKGEDRISV

LIPQLHRDKDAWGDNVEEFQPERFEELDKVPHHAYKPFGNGQRACIGMQFALHEATLVMG

MLLQHFEFIDYEEYQLDVKQTLTLKPGDFKIRIVPRNQTISHTTVLAPTEEKLKNHEIKQ

QVQKTPSIIGADNLSLLVLYGSDTGVAEGIARELADTASLEGVQTEVAALNDRIGSLPKE

GAVLIVTSSYNGKPPSNAGQFVQWLEELKGDELKGVQYAVFGCGDHNWASTYQRIPRYID

EQMAQKGATRFSTRGEADASGDFEEQLEQWKQRMWSDAMKVFGLELNKNMEKERSTLSLQ

FVSRLGGSPLARTYEAVYASILENRELQSSSSERSTRHIEISLPEGATYKEGDHLGVLPI

NSEKNVNRILKRFGLNGKDQVILSASGRSVNHIPLDSPVRLYDLLSYSVEVQEAATRAQI

REMVTFTACPPHKKELESLLEDGVYQEQILKKRISMLDLLEKYEACEIRFEPFLELLPAL

KPRYYSISSSPLVAQDRLSITVGVVNAPAWSGEGTYEGVASNYLAQRHNKDEIICFIRTP

QSNFQLPENPETPIIMVGPGTGIAPFRGFLQARRVQKQKGMNVGEAHLYFGCRHPEKDYL

YRTELENDERDGLISLHTAFSRLEGQAKTYVQHVIKEDRIHLISLLDNGAHLYICGDGSK

MAPDVEDTLCQAYQEIHEVSEQEARNWLDRLQEEGRYGKDVWAGI

>CYP106B1 *Bacillus anthracis* HYU01

MASPENVILVHEISKLKTKEELWNPYEWYQFMRDNHPVHYDDEQDVWNVFLYDDVNRVLS

DYSLFSSRRERRQFAIPPLETRININSTDPPEHRNVRSIVSKAFTPRSLEQWKPRIQSIA

NELVKDIENCSEVDIVEQFAAPLPVTVISDLLGVPTTDRKKIKAWSDILFMPYSKEKFND

LDAEKGIALNEFKAYLLPIVQEKRYHLTDDIISDLIRAEYEGERLTDEEIVTFSLGLLAA

GNETTTNLIINSFYCFLVDSPATYKEVREKPKLISKAVEEVLRYRFPVTLARRITEDTNI

FGPLMKKDQMVVAWVSAANLDEKKFSQASKFNIHRIGNEKHLTFGKGPHFCLGAPLARLE

AEIALTTFINAFEKIALSPSFNIEQCILENEQTLKFLPIRLKPQ

>CYP107J2 *Bacillus anthracis* HYU01

MAMKNKVGIRIEDGINLASAQFKEDAYEIYKESRKVQPVLFVNKTELGAEWLITRYEDAL

PLLKDNRLKKDPANVFSQDTLNVFLTVDNSDYLTTHMLNSDPPNHNRLRSLVQKVFTPKM

IAQLEGRIQDIADDLLNEVERKGSLNLVDDYSFPLPIIVISEMLGIPKEDQAKFRIWSHA

VIAYPETPEEIKETEKQLSEFITYLQYLVDMKRKEPKEDLVSALILAESEGHKLSARELY

SMIMLLIVAGHETTVNLITNTVLALLENPNQLQLLKENPKLIDAAIEEGLRYYSPVEVTT

SRWADEPFQIHDQTIEKGDMVVIALAAANRDETVFENPEVFDITRENNRHIAFGHGSHFC

LGAPLARLEAKIAITTLFERMPELQIKGNREDIKWQGNYLMRSLEELPLTF

>CYP102A4 *Bacillus anthracis* *Sterne*

MDKKVSAIPQPKTYGPLGNLPLIDKDKPTLSFIKLAEEYGPIFRMQTLSDTIIVVSGHEL

VAEVCDETRFDKSIEGALAKVRAFAGDGLFTSETQEPNWQKAHNILMPTFSQRAMKDYHA

MMVDIAVQLVQKWARLNPNENVDVPEDMTRLTLDTIGLCGFNYRFNSFYRETPHPFITSM

TRALDEAMHQLQRLDIEDKLMWRTKRQFQHDIQSMFSLVDNIIAERKSSENQEENDLLSR

MLNVQDPETGEKLDDENIRFQIITFLIAGHETTSGLLSFAIYFLLKNPDKLKKAYEEVDR

VLTDSTPTYQQVMKLKYIRMILNESLRLWPTAPAFSLYAKEDTVIGGKYPIKKGEDRISV

LIPQLHRDKDAWGDNVEEFQPERFEELDKVPHHAYKPFGNGQRACIGMQFALHEATLVMG

MLLQHFEFIDYEEYQLDVKQTLTLKPGDFKIRIVPRNQTISHTTVLAPTEEKLKNHEIKQ

QVQKTPSIIGADNLSLLVLYGSDTGVAEGIARELADTASLEGVQTEVAALNDRIGSLPKE

GAVLIVTSSYNGKPPSNAGQFVQWLEELKGDELKGVQYAVFGCGDHNWASTYQRIPRYID

EQMAQKGATRFSTRGEADASGDFEEQLEQWKQRMWSDAMKVFGLELNKNMEKERSTLSLQ

FVSRLGGSPLARTYEAVYASILENRELQSSSSERSTRHIEISLPEGATYKEGDHLGVLPI

NSEKNVNRILKRFGLNGKDQVILSASGRSVNHIPLDSPVRLYDLLSYSVEVQEAATRAQI

REMVTFTACPPHKKELESLLEDGVYQEQILKKRISMLDLLEKYEACEIRFEPFLELLPAL

KPRYYSISSSPLVAQDRLSITVGVVNAPAWSGEGTYEGVASNYLAQRHNKDEIICFIRTP

QSNFQLPENPETPIIMVGPGTGIAPFRGFLQARRVQKQKGMNVGEAHLYFGCRHPEKDYL

YRTELENDERDGLISLHTAFSRLEGQAKTYVQHVIKEDRIHLISLLDNGAHLYICGDGSK

MAPDVEDTLCQAYQEIHEVSEQEARNWLDRLQEEGRYGKDVWAGI

>CYP106B1 *Bacillus anthracis Sterne*

MASPENVILVHEISKLKTKEELWNPYEWYQFMRDNHPVHYDDEQDVWNVFLYDDVNRVLS

DYSLFSSRRERRQFAIPPLETRININSTDPPEHRNVRSIVSKAFTPRSLEQWKPRIQSIA

NELVKDIENCSEVDIVEQFAAPLPVTVISDLLGVPTTDRKKIKAWSDILFMPYSKEKFND

LDAEKGIALNEFKAYLLPIVQEKRYHLTDDIISDLIRAEYEGERLTDEEIVTFSLGLLAA

GNETTTNLIINSFYCFLVDSPATYKEVREKPKLISKAVEEVLRYRFPVTLARRITEDTNI

FGPLMKKDQMVVAWVSAANLDEKKFSQASKFNIHRIGNEKHLTFGKGPHFCLGAPLARLE

AEIALTTFINAFEKIALSPSFNIEQCILENEQTLKFLPIRLKPQ

>CYP107J2 *Bacillus anthracis Sterne*

MAMKNKVGIRIEDGINLASAQFKEDAYEIYKESRKVQPVLFVNKTELGAEWLITRYEDAL

PLLKDNRLKKDPANVFSQDTLNVFLTVDNSDYLTTHMLNSDPPNHNRLRSLVQKVFTPKM

IAQLEGRIQDIADDLLNEVERKGSLNLVDDYSFPLPIIVISEMLGIPKEDQAKFRIWSHA

VIAYPETPEEIKETEKQLSEFITYLQYLVDMKRKEPKEDLVSALILAESEGHKLSARELY

SMIMLLIVAGHETTVNLITNTVLALLENPNQLQLLKENPKLIDAAIEEGLRYYSPVEVTT

SRWADEPFQIHDQTIEKGDMVVIALAAANRDETVFENPEVFDITRENNRHIAFGHGSHFC

LGAPLARLEAKIAITTLFERMPELQIKGNREDIKWQGNYLMRSLEELPLTF

>CYP102A4 *Bacillus anthracis* Vollum

MDKKVSAIPQPKTYGPLGNLPLIDKDKPTLSFIKLAEEYGPIFRMQTLSDTIIVVSGHEL

VAEVCDETRFDKSIEGALAKVRAFAGDGLFTSETQEPNWQKAHNILMPTFSQRAMKDYHA

MMVDIAVQLVQKWARLNPNENVDVPEDMTRLTLDTIGLCGFNYRFNSFYRETPHPFITSM

TRALDEAMHQLQRLDIEDKLMWRTKRQFQHDIQSMFSLVDNIIAERKSSENQEENDLLSR

MLNVQDPETGEKLDDENIRFQIITFLIAGHETTSGLLSFAIYFLLKNPDKLKKAYEEVDR

VLTDSTPTYQQVMKLKYIRMILNESLRLWPTAPAFSLYAKEDTVIGGKYPIKKGEDRISV

LIPQLHRDKDAWGDNVEEFQPERFEELDKVPHHAYKPFGNGQRACIGMQFALHEATLVMG

MLLQHFEFIDYEEYQLDVKQTLTLKPGDFKIRIVPRNQTISHTTVLAPTEEKLKNHEIKQ

QVQKTPSIIGADNLSLLVLYGSDTGVAEGIARELADTASLEGVQTEVAALNDRIGSLPKE

GAVLIVTSSYNGKPPSNAGQFVQWLEELKGDELKGVQYAVFGCGDHNWASTYQRIPRYID

EQMAQKGATRFSTRGEADASGDFEEQLEQWKQRMWSDAMKVFGLELNKNMEKERSTLSLQ

FVSRLGGSPLARTYEAVYASILENRELQSSSSERSTRHIEISLPEGATYKEGDHLGVLPI

NSEKNVNRILKRFGLNGKDQVILSASGRSVNHIPLDSPVRLYDLLSYSVEVQEAATRAQI

REMVTFTACPPHKKELESLLEDGVYQEQILKKRISMLDLLEKYEACEIRFEPFLELLPAL

KPRYYSISSSPLVAQDRLSITVGVVNAPAWSGEGTYEGVASNYLAQRHNKDEIICFIRTP

QSNFQLPENPETPIIMVGPGTGIAPFRGFLQARRVQKQKGMNVGEAHLYFGCRHPEKDYL

YRTELENDERDGLISLHTAFSRLEGQAKTYVQHVIKEDRIHLISLLDNGAHLYICGDGSK

MAPDVEDTLCQAYQEIHEVSEQEARNWLDRLQEEGRYGKDVWAGI

>CYP106B1 *Bacillus anthracis Vollum*

MASPENVILVHEISKLKTKEELWNPYEWYQFMRDNHPVHYDDEQDVWNVFLYDDVNRVLS

DYSLFSSRRERRQFAIPPLETRININSTDPPEHRNVRSIVSKAFTPRSLEQWKPRIQSIA

NELVKDIENCSEVDIVEQFAAPLPVTVISDLLGVPTTDRKKIKAWSDILFMPYSKEKFND

LDAEKGIALNEFKAYLLPIVQEKRYHLTDDIISDLIRAEYEGERLTDEEIVTFSLGLLAA

GNETTTNLIINSFYCFLVDSPATYKEVREKPKLISKAVEEVLRYRFPVTLARRITEDTNI

FGPLMKKDQMVVAWVSAANLDEKKFSQASKFNIHRIGNEKHLTFGKGPHFCLGAPLARLE

AEIALTTFINAFEKIALSPSFNIEQCILENEQTLKFLPIRLKPQ

>CYP107J2 *Bacillus anthracis Vollum*

MAMKNKVGIRIEDGINLASAQFKEDAYEIYKESRKVQPVLFVNKTELGAEWLITRYEDAL

PLLKDNRLKKDPANVFSQDTLNVFLTVDNSDYLTTHMLNSDPPNHNRLRSLVQKVFTPKM

IAQLEGRIQDIADDLLNEVERKGSLNLVDDYSFPLPIIVISEMLGIPKEDQAKFRIWSHA

VIAYPETPEEIKETEKQLSEFITYLQYLVDMKRKEPKEDLVSALILAESEGHKLSARELY

SMIMLLIVAGHETTVNLITNTVLALLENPNQLQLLKENPKLIDAAIEEGLRYYSPVEVTT

SRWADEPFQIHDQTIEKGDMVVIALAAANRDETVFENPEVFDITRENNRHIAFGHGSHFC

LGAPLARLEAKIAITTLFERMPELQIKGNREDIKWQGNYLMRSLEELPLTF

>CYP102A5 *Bacillus cereus* ATCC 14579

MEKKVSAIPQPKTYGPLGNLPLIDKDKPTLSFIKIAEEYGPIFQIQTLSDTIIVVSGHEL

VAEVCDETRFDKSIEGALAKVRAFAGDGLFTSETHEPNWKKAHNILMPTFSQRAMKDYHA

MMVDIAVQLVQKWARLNPNENVDVPEDMTRLTLDTIGLCGFNYRFNSFYRETPHPFITSM

TRALDEAMHQLQRLDIEDKLMWRTKRQFQHDIQSMFSLVDNIIAERKSSGDQEENDLLSR

MLNVPDPETGEKLDDENIRFQIITFLIAGHETTSGLLSFAIYFLLKNPDKLKKAYEEVDR

VLTDPTPTYQQVMKLKYMRMILNESLRLWPTAPAFSLYAKEDTVIGGKYPIKKGEDRISV

LIPQLHRDKDAWGDNVEEFQPERFEELDKVPHHAYKPFGNGQRACIGMQFALHEATLVMG

MLLQHFELIDYQNYQLDVKQTLTLKPGDFKIRILPRKQTISHPTVLAPTEDKLKNDEIKQ

HVQKTPSIIGADNLSLLVLYGSDTGVAEGIARELADTASLEGVQTEVVALNDRIGSLPKE

GAVLIVTSSYNGKPPSNAGQFVQWLEELKPDELKGVQYAVFGCGDHNWASTYQRIPRYID

EQMAQKGATRFSKRGEADASGDFEEQLEQWKQNMWSDAMKAFGLELNKNMEKERSTLSLQ

FVSRLGGSPLARTYEAVYASILENRELQSSSSDRSTRHIEVSLPEGATYKEGDHLGVLPV

NSEKNINRILKRFGLNGKDQVILSASGRSINHIPLDSPVSLLALLSYSVEVQEAATRAQI

REMVTFTACPPHKKELEALLEEGVYHEQILKKRISMLDLLEKYEACEIRFERFLELLPAL

KPRYYSISSSPLVAHNRLSITVGVVNAPAWSGEGTYEGVASNYLAQRHNKDEIICFIRTP

QSNFELPKDPETPIIMVGPGTGIAPFRGFLQARRVQKQKGMNLGQAHLYFGCRHPEKDYL

YRTELENDERDGLISLHTAFSRLEGHPKTYVQHLIKQDRINLISLLDNGAHLYICGDGSK

MAPDVEDTLCQAYQEIHEVSEQEARNWLDRVQDEGRYGKDVWAGI

>CYP107J3 *Bacillus cereus* ATCC 14579

MKNKVGLSIEDGINLASAQFKEDAYEIYKESRKKQPILFVNQVEIGKEWLITRYEDALPL

LKDNRLKKDWTNVFSQDIKNMYLSVDNSDHLTTHMLNSDPPNHSRLRSLVQKAFTPKMIA

QLDGRIQRIADDLISDIERKGTLNLVDDYSFPLPIIVISEMLGIPKEDQAKFRIWSHAVI

ASPETPEEIKETEKQLSEFITYLQYLVDIKRKEPKEDLVSALILAESEGHKLSARELYSM

IMLLIVAGHETTVNLITNTVLALLENPNQLQLLKDNPKLIDSAIEEGLRYYSPVEVTTAR

WAAEPFQIHHQTIQKGDMVIIALASANRDETVFENPEIFDITRENNRHIAFGHGSHFCLG

APLARLEAKIAITTLFNRMPELQIKGNREEIKWQGNYLMRSLEELPLTF

>CYP107J4P *Bacillus cereus* ATCC 14579

MKEPQLQQHLEKFIQYIEALVNEKRLNPDADLISELVQTKEQEDKLSNNELLSTIWLLII

AGHETTVNLISNGLLALLQHPEQMNLIRENPSLIPSAVDELLRHSGPVMFISRLASEDMT

IHGKRIPKGDLVLLSLTAANIDPQKFTYPETLNISREENNHLAFGAGIHHCLGAPLARLE

GQIALGTLLQRLPNLRLAIKPDQLNYNHSKIRSLVNLPVVF

>CYP102A5 *Bacillus cereus* B4264

MVAEVCDETRFDKSIEGALAKVRAFAGDGLFTSETHEPNWKKAHNILMPTFSQRAMKDYH

AMMVDIAVQLVQKWARLNPNENVDVPEDMTRLTLDTIGLCGFNYRFNSFYRETPHPFITS

MTRALDEAMHQLQRLDIEDKLMWRTKRQFQHDIQSMFSLVDNIIAERKSSGDQEENDLLS

RMLNVKDPETGEKLDDENIRFQIITFLIAGHETTSGLLSFAIYFLLKNPDKLKKAYEEVD

RVLTDPTPTYEQVMKLKYIRMILNESLRLWPTAPAFSLYAKEDTVIGGKYPIKKGEDRIS

VLIPQLHRDKDAWGDNVEEFQPERFEELDKVPHHAYKPFGNGQRACIGMQFALHEATLVM

GMLLQHFELIDYQNYQLDVKQTLTLKPGDFKIRILPRKQTISHPTVLAPTEDKLKNDEIK

QHVQKTPSIIGADNLSLLVLYGSDTGVAEGIARELADTASLEGVRTEVVALNDQIGSLPK

EGAVLIVTSSYNGKPPSNAGQFVQWLEELKPDELKGVQYAVFGCGDHNWASTYQRIPRYI

DEQMAQKGATRFSKRGEADASGDFEEQLEQWKQSMWSDAMKAFGLELNKNMEKERSTLSL

QFVSRLGGSPLARTYEAVYASILENRELQSSSSDRSTRHIEVSLPEGATYKEGDHLGVLP

VNSEKNINRILKRFGLNGKDQVILSASGRSINHIPLDSPVSLLDLLSYSVEVQEAATRAQ

IREMVTFTACPPHKKELEALLEEGVYHEQILKKRISMLDLLEKYEACEIRFERFLELLPA

LKPRYYSISSSPLVAHNRLSITVGVVNAPAWSGEGTYEGVASNYLAQRHNKDEIICFIRT

PQSNFELPKDPETPIIMVGPGTGIAPFRGFLQARRVQKQKGMNLGQAHLYFGCRHPEKDY

LYRTELENDERDGLISLHTAFSRLEGHPKTYVQHLIKQDRINLISLLDNGAHLYICGDGS

KMAPDVEDTLCQAYQEIHEVSEQEARNWLDRVQDEGRYGKDVWAGI

>CYP106B4 *Bacillus cereus* B4264

MASPENVILVHEISKLKTKEELWNPYEWYRLMRDNHPVHYDEEQDVWNVFLYDDVNRVLS

DYRLFSSRRERRQFSIPPLETRININSTDPPEHRNVRSIVSKAFTPRSLEQWKPRIQAIA

DELVQHIEKCSEVNIVEQFAAPLPVTVISDLLGVPTTDRKKIKEWSDILFMPYSKEKFND

LNAEKGIALNEFKAYLLPIVQEKRYHLTDDIISDLIRAEYEGERLTDEEIVTFSLGLLAA

GNETTTNLIINSFYCFLVDSPGIYEELRKEPNLISKAIEEVLRYRFPVTLARRITEDTNI

FGPLMKKDQMIVAWVSAANLDEKKFSQASQFNVHRTGNEKHLTFGKGPHFCLGAPLARLE

AEIALTTFINTFEKIELSPSFCLEKCILENEQTLKYLPIRLKAK

>CYP107J3 *Bacillus cereus* B4264

MSMKNKVGLSIEDGINLASAQFKEDAYEIYKESRKKQPILFVNQVEIGKEWLITRYEDAL

PLLKDNRLKKDWTNVFSQDIKNMYLSVDNSDHLTTHMLNSDPPNHSRLRSLVQKAFTPKM

IAQLDKRIEKIADDLISDIERKGTLNLVDDYSFPLPIIVISEMLGIPKEDQAKFRIWSHA

VIASPETPEEIKETEKQLSEFITYLQYLVDIKRKEPKEDLVSALILAESEGHKLSARELY

SMIMLLIVAGHETTVNLITNTVLALLENPNQLQLLKDNPKLIDSAIEEGLRYYSPVEVTT

ARWAAEPFQIHDRTIEKGDMVVIALASANRDETVFENPEVFDITRENNRHIAFGHGSHFC

LGAPLARLEAKIAITTLFNRMPELQIKGNREEIKWQGNYLMRSLEELPLTF

>CYP102A5 *Bacillus cereus* G9842

MEKKVSAIPQPKTYGPLGNLPLIDKDKPTLSFIKLAEEYGPIFRIQTLSDAIIVVSGHEL

VAEVCDETRFDKSIEGALAKVRAFAGDGLFTSETHEPNWKKAHNILMPTFSQRAMKDYHA

MMVDLAVQLVQKWARLNPNEDVDVPEDMTRLTLDTIGLCGFNYRFNSFYRETPHPFITSM

SRALDEAMHQLQRLDIEDKLMWRTKRQFQHDIQSMFSLVDNIIAERKSSGDQEENDLLSR

MLNVQDPETGEKLDDENIRFQIITFLIAGHETTSGLLSFAIYFLLKNPDKLKKAYEEVDR

ILTDPTPTYQQVMKLKYIRMILNESLRLWPTAPAFSLYAKEDTVIGGKYPIKKGEDRISV

LIPQLHRDKDAWGDNVEEFQPERFEELDKVPHHAYKPFGNGQRACIGMQFALHEATLVMG

MLLQHFELIDYQNYQLDVKQTLTLKPGDFKIRILPRKQTISHPTVLAPTEDKLKNHEIKQ

HVQKTPSIIGADNLSLLVLYGSDTGVAEGIARELADTASLEGVQTEVVALNDRIGSLPKE

GAVLIVTSSYNGKPPSNAGQFVQWLEELKPDELKGVQYAVFGCGDHNWASTYQRIPRYID

EQMAQKGATRFSKRGEADASGDFEEQLEQWKQGMWSDAMKAFGLEFNKNMEKERSTLSLQ

FVSRLGGSPLARTYEAVYATILENRELQSSSSDRSTRHIEVSLPEGATYQEGDHLGVLPI

NSEKNVNRILKRFGLNGKDQVILSASGRSINHIPLDSPVSLLDLLSYSVEVQEAATRAQI

REMVTFTACPPHKKELEALLEEGVYHEQILKKRISMLDLLEKYEACEIRFERFLELLPAL

KPRYYSISSSPLVAQNRLSITVGVVNAPAWSGEGTYEGVASNYLAQRHNKDEIICFIRTP

QSNFELPKDPETPIIMVGPGTGVAPFRGFLQARRVQKQKGINLGQAHLYFGCRHPEKDYL

YRTELENDERDGLISLHTAFSRLEGHPKTYVQHLIKQDSINLISLLDNGAHLYICGDGSK

MAPDVEDTLCQAYQEIHEVSEQEARNWLDRVQDEGRYGKDVWAGI

>CYP106B4 *Bacillus cereus* G9842

MASPENVILVHEISKLKTKEELWNPYEWYRLMRDNHPVHYDEEQDVWNVFLYDDVNRVLS

DYRLFSSRRERRQFSIPPLETRININSTDPPEHRNVRSIVSKAFTPRSLEQWKPRIQAIA

DELVQHIEKYSEVNIVEQFAALLPVTVISDLLGVPTTDRKKIKEWSDILFMPYSKEKFND

LDAEKEIALNEFKAYLLPIVQEKRYHLTDDIISDLIRAEYEGERLTDEEIVTFSLGLLAA

GNETTTNLIINSFYCFLVDSPGIYEDLRKTPSLISKAIEEVLRYRFPVTLARRITEDTNI

FGPLMKKDQMIVAWVSAANLDEKKFSHPSQFNVHRIGNEKHLTFGKGPHFCLGAPLARLE

AEIALTTFINAFEKIELSPSFCLEKCILENEQTLKYLPIRLKAK

>CYP107J2 *Bacillus cereus* G9842

MSMKNKVGLKIEEGINLASAQFKEDAYEIYKESRKMQPILFVNKTELGAEWLITRYEDAL

PLLKDSRLKKDPANVFSQDTLNVFLTVDNSDYLTTHMLNSDPPNHNRLRSLVQKAFTPKM

ITQLEGRIQDIADDLLNEVERKGSLNLVDDYSFPLPIIVISEMLGIPKEDQAKFRIWSHA

VIAYPETPEEIKETEKQLSEFITYLQYLVDIKRKEPKEDLVSALILAESEGHKLSARELY

SMIMLLIVAGHETTVNLITNTVLALLENPDQLQLLKENPKLIDAAIEEGLRYYSPVEVTT

SRWADEPFQIHDQTIEKGDMVVIALASANRDETVFENPEVFDITRENNRHIAFGHGSHFC

LGAPLARLEAKIAITTLFNRMPELQIKGNREEIKWQGNYLMRSLEELPLTF

>CYP102A8 *Bacillus cereus* NC7401

MDKKVSAIPQPKTYGPLGNLPLIDKDKPTLSFIKLAEEYGPIFQIQTLSDTIIVVSGHEL

VAEVCDETRFDKSIEGALAKVRAFAGDGLFTSETDEPNWKKAHNILMPTFSQRAMKDYHA

MMVDIAVQLVQKWARLNPNENVDVPGDMTRLTLDTIGLCGFNYRFNSFYRETSHPFITSM

TRALDEAMHQLQRLDIEDKLMWRTKRQFQHDIQSMFSLVDNIIAERKSSGNQEENDLLSR

MLHVQDPETGEKLDDENIRFQIITFLIAGHETTSGLLSFAIYFLLKNPDKLEKAYEEVDR

VLTDPTPTYQQVMKLKYIRMILNESLRLWPTAPAFSLYAKEDTVIGGKYPIKKGEDRISV

LIPQLHRDKDAWGDNVEEFQPERFEELDKVPHHAYKPFGNGQRACIGMQFALHEATLVMG

MLLQHFEFIDYEDYQLDVKQTLTLKPGDFKIRIVPRNETISHNTVLAPTEEKLKNDETKQ

QVQKTPSIIGADNLSLLVLYGSDTGVAEGIARELADTASLEGVQTEVAALNDRIGSLPKE

GAVLIVTSSYNGKPPSNAGQFVQWLEELKPDELKGVQYAVFGCGDHNWASTYQRIPRYID

EQMAQKGATRFSKRGEADASGDFEEQLEQWKQSMWSDAMKSFGLELNKNMEKERSTLSLQ

FVSRLGGSPLARTYEAVYASILENRELQSSSSERSTRHIEISLPEGATYKEGDHLGVLPI

NSEKNVNRILRRFGLNGKDQVILSASGRSVNHIPLDSPVRLYDLLSYSVEVQEAATRAQI

REMATFTVCPPHKKELESLLEEGVYQEQILKKRISMLDLLEKYEACEIRFERFLELLPAL

KPRYYSISSSPLVAQDRLSITVGVVNAPAWSGEGTYEGVASNYLAQRHNKDEIICFIRTP

QSNFQLPENLETPIIMVGPGTGIAPFRGFLQARRVQKQKGMKVGEAHLYFGCRHPEKDYL

YRTELENDEREGLISLHTAFSRLEGHPKTYVQHVIKEDRIHLISLLDNGAHLYICGDGSK

MAPDVEDTLCQAYQEIHEVSEQEARNWLDRLQDEGRYGKDVWAGI

>CYP107J2 *Bacillus cereus* NC7401

MSMKNKVGLRIENGINLASAQFKEDAYEIYKESRKMQPILFVNKTELGAEWLITRYEDAL

PLLKDNRLKKDPANVFSQDTLNVFLTVDNSDYLTTHMLNSDPPNHNRLRSLVQKAFTPKM

IAQLEGRIQHIADDLLNEVERKDSLNLVDDYSFPLPIIVISEMLGIPKEDQAKFRIWSHA

VIAYPETPEEIKETEKQLSEFITYLQYLVDMKRKEPKEDLVSALILAESEGHKLNARELY

SMIMLLIVAGHETTVNLITNTVLALLENPDQLQLLKENPKLIDAAIEEGLRYYSPVEVTT

SRWADEPFQIHDQTIEKGDMVVIALASANRDETVFENPEVYDITRENNRHIAFGHGSHFC

LGAPLAKLEAKIAITTLFNRMPKLQIKGDREEIKWQGNYLMRSLEELPLTF

>CYP109T2 *Bacillus cereus* NC7401

MKTPYQSVVMIPTNKLMGKKALEDPYKPFAWYKEMREKEPICFNHQADMWNVFLYEDVKT

VLEDKEYFSSIMPEKKKSPFPQSILGMDPPKHTQIRSIVNRSFTPKALREWEPRIQQITN

DILNQLSNRETFDIVRELFYPLPVIVIAEMLGVSAKDMERFKRWSDIIVSSPSHDDSDYL

EEFFNTRLQAENELGEFFEEIIQSNRGKSKKDSNNIISLLVQSEADKNISGKEIVPFCKL

LLVAGNETTTNLLGNALYCFIEHPNVYDQLQQDVSLVPKAIEEVLRYRSPVQRIVRRVKK

EIQLKGQTLQVDQIISAWVGSANRDSHQFKDVDSFNIYRRRNPHLTFGHGIHFCLGAPLA

RLEAKIVLTELIKRYKSFSFIDQDLPVPISNSSSIYGLHSFPVKSELN

>CYP102A8 *Bacillus cereus* Q1

MDKKVSAIPQPKTYGPLGNLPLIDKDKPTLSFIKLAEEYGPIFQIQTLSDTIIVVSGHEL

VVEVCDETRFDKSIEGALAKVRAFAGDGLFTSETDEPNWKKAHNILMPTFSQRAMKDYHA

MMVDIAVQLVQKWARLNPNENVDVPGDMTRLTLDTIGLCGFNYRFNSFYRETSHPFITSM

TRALDEAMHQLQRLDIEDKLMWRTKRQFQHDIQSMFSLVDNIIAERKSSGNQEENDLLSR

MLHVQDPETGEKLDDENIRFQIITFLIAGHETTSGLLSFAIYFLLKNPDKLKKAYEEVDR

VLTDPTPTYQQVMKLKYIRMILNESLRLWPTAPAFSLYAKEDTVIGGKYPIKKGEDRISV

LIPQLHRDKDAWGDNVEEFQPERFEELDKVPHHAYKPFGNGQRACIGMQFALHEATLVMG

MLLQHFEFIDYEDYQLDVKQTLTLKPGDFKIRIVPRNQTISHTTVLAPTEEKLKNHETKQ

QVQKTPSIIGADNLSLLVLYGSDTGVAEGIARELADTASLEGVQTEVAALNDRIGSLPKE

GAVLIVTSSYNGKPPSNAGQFVQWLEELKPDELKGVQYAVFGCGDHNWASTYQRIPRYID

EQMAQKGATRFSKRGEADASGDFEEQLEQWKQSMWSDAMKSFGLELNKNMEKERSTLSLQ

FVSRLGGSPLARTYEAVYASILENRELQSSSSERSTRHIEISLPEGATYKEGDHLGVLPI

NSEKNVNRILKRFGLNGKDQVILSASGRSVNHIPLDSPVRLYDLLSYSVEVQEAATRAQI

REMVTFTVCPPHKKELESLLEEGVYQEQILKKRISMLDLLEKYEACEIRFERFLELLPAL

KPRYYSISSSPLVAQDRLSITVGVVNAPAWSGEGTYEGVASNYLAQRHNKDEIICFIRTP

QSNFQLPENPETPIIMVGPGTGIAPFRGFLQARRVQKQKGMKVGEAHLYFGCRHPEKDYL

YRTELENDEREGLISLHTAFSRLEGHPKTYVQHVIKEDRIHLISLLDNGAHLYICGDGSK

MAPDVEDTLCQAYQEIHEVSEQEARNWLDRLQDEGRYGKDVWAGI

>CYP107J2 *Bacillus cereus* Q1

MKNKVGLRIENGINLASAQFKEDAYEIYKESRKMQPILFVNKTELGAEWLITRYEDALPL

LKDNRLKKDPANVFSQDTLNVFLTVDNSDYLTTHMLNSDPPNHNRLRSLVQKAFTPKMIA

QLEGRIQHIADDLLNEVERKDSLNLVDDYSFPLPIIVISEMLGIPKEDQAKFRIWSHAVI

AYPETPEEIKETEKQLSEFITYLQYLVDMKRKEPKEDLVSALILAESEGHKLNARELYSM

IMLLIVAGHETTVNLITNTVLALLENPDQLQLLKENPKLIDAAIEEGLRYYSPVEVTTSR

WADEPFQIHDQTIEKGDMVVIALASANRDETVFENPEVYDITRENNRHIAFGHGSHFCLG

APLAKLEAKIAITTLFNRMPKLQIKGDREEIKWQGNYLMRSLEELPLTF

>CYP107DY4 *Bacillus cereus* Q1

MVKRHVISDHSIVIMIRRILVKKLTFKDLNSPETMRNPIMFYENLIKQQERFFQIDDFYG

MGGTWVALHYDDVITILKDSRFIKDLRKFTSPHDKQHPNSGNTAASKMFDWLMNMPNMLT

VDPPDHTRLRRLVSKAFTPRMIEDLRPRIQQIADELLVAVQEHGKMDIISDFAYPLPIIV

ISEMLGIPATDRNQFREWTQILMNVSVNPSQGNAVTTTLEKFIQYIEVLLNEKRLNPAAD

LISELVQTKEQEDRLSNNELLSTIWLLIIAGHETTVNLISNGVLSLLQHPEQMNLLRENP

SLIPSAVDELLRYSGPVMFISRLASEDMTIHGKRIRKGDLVLLSLTAANIDPQKFSYPEE

LNISREENNHLAFGAGIHHCLGAPLARLEGQIALGTLLQRLPNLCLAIKPDQLNYNHSKI

RSLVNLPVVF

>CYP107H11(BkAM31D_02760 K16593)*Bacillus krulwichiae*

MKTEFEDLTQNPYPFYEEIRAQSPIHWGEFFGNRGWYITGYEEASMILKDLRFQNRMPLP

ETTKWFERLKEIQKQMVLYQNPPDHRKIRRLVNERFTPKVLERYVFMIEETAEQLFSHIK

NDKKLDFIADFAFPLASTVIARLLGVPERDKDLFRRWALTLIDTIDLNRSRKSLVKGNEC

IVEMVHYFKSLILNSKRNRKNDLISVLVEEQNNDKLTEDEVLSTCILLLIAGHETTVNLI

SNAIYCLATNQEQQIKLREEPLLLKTAIEEVLRYESPTQLTARIASTDVIINQTQIKKGD

HIYLFLGACNRDPKNFPNANTLDFAREPNPHLSFGAGIHFCLGSELAKIETKAALRTILK

NTNSIQIDSKQLEWRNLTGFRALKELPVVFN

>CYP223G1(BkAM31D_08685)*Bacillus krulwichiaebkw*

MSKLNESKCPFNYGNLESYENELFKEVKIDKRGDHDFDPKAPESFTSAHDLYKEMRSKCP

VAHSNEWGGFWALSKYEDVVGVLKDYETYTTSVQNVVPKVAFTGRRPPLHFDPPEHTVYR

RLINQFFTKKKMLKLEPIIQRDTEELVKALMEKDEVEIAADYSHQLPSLVFAQFFNLPKS

LSKKIKEVSTAYVKAIMEFDNQEKVKGLSLQLYDIARTVIDSRKENPMDPEEDFTSALLN

TKQEGEYLTDDMVLGCVRAMLVAGMIAPSVLIASIFVHLAKHKDIQEQLRNDLSLIPAAT

EEYLRLLTPYRGMARTAKQDVVIGGQLIKKDEPIALNYASANRDDEVFPDGDTFILNRPN

INKHIVFGEGPHKCPASPLARMMVRFAIEEALKRTKDIDLIGEIKMTVWAEWGVLEAPMK

LTPVK

>CYP223G2(BkAM31D_08710)*Bacillus krulwichiaebkw*

MNNQNNTSQQKCPFHQGTIDLSLDSQDKLDERLTNDFDPTKKETFTSAHEEYKELRQRCP

VAYSEAYDGFWALLKYEDIVNVLKDPNTYVTSVQNVVPKVSTTGRRPPLHLDPPEHTPYR

RTLDPFFTDEKMNEIEPVIRKTTIDLLQPFINSGGGDICTEFSHQLPGYVFAQFFNLPTE

LGMKIREVTKLYVKALNEVDKPTIQEKSLELYDIARMIIQMRKEAPLDPTTDITSAFLAR

KYKGEPLPDELILGTIRQLIVVGMIAPVVFIGSMSVHLAENQDVQELLRNDPSLIPAAIE

EYLRLFTPYRGFARTSKHDVEIRGRKIKKDEPIAVVFASANRDEEVFPNADKFILNRPNI

KKHVAFGMGPHQCPGAPLARLILNITLKELLVRTKKLELDGEVIMTRFPEWGPVSVPLKV

VN

>CYP107H9 *Bacillus pseudofirmus*

MNTVSQTKAKQIRLKRDDLFKDPYGLYKRLRERGPIYKGSILKQPGWFVTGYKETAYILK

DSSFFTRIPLPEATEKYSHLKQIQSKMMLYMNKEDHRKLRLLVNKGFTPKQIAGFRPLIE

EVVDSLLDELIVKEEFDVVSEFAFPLTSLVIATILGVPEEDRDRFRAWAALLLQSIDFSR

SHKTLEASDQLAEELSEYFSSLIAQKRAEPGDDLISMLIKENDCTENELISTFILLVIAG

HETTVNLISNTIYTFLKHPEQWKMLNENPTLAGSAIEEVLRFESPTQLTARVAMKDTEVA

GKHITCGEQLYLMLGAANRDPDVFDEPDEFLITRKKVPHLSFGLGAHFCLGSTLARLEAQ

IALSRFSKRVHTYNQTKTEIKWRPLTGFRALEKLRITVQTSN

>CYP152A14 *Bacillus pseudofirmus*

MKTSQANIPSEKGLDHSLSLLKDGYLFIHKRAQDFQSDIFKTRLMGEEAICLRGEDGAKV

FYDNDKFKREGAAPKRVQKTLFGEDAIQSMDGEAHRHRKQLFMSLMSRERLQELNEITRA

QWLLYIKKWETQKKIVLFDEMEAMLTKVACEWAGVPLEEKEVEKRTKDFGDMIDAFGAVG

VRHARGRSARKRTEAWITSLIEEVRSEQRSPRESTALYQMAWHKELDGTLMDAKMAAIEL

INVIRPTVAVGRFITFGAVGLHEFPKEREKLMQDQDGTYSHLFTQEVRRYYPFAPFTGAR

VKKDFTWNSHQFKENTLVLLDIYGTNRHPKLWESPNEFRPERFKGWEGSPFSFIPQGGGD

EHKGHRCAGEWITIELMKISLSQLAQNITYDVPKQDLTYDLSRMPSIPKSRFIIQNVRRQ

QQ

>CYP1221A1 *Bacillus pseudofirmus*

MTNRFQRRKPIPGPKEHWLKGSLQAFTSDPLRFLSNQAETFGPVSSFRFGPFQEVYFVND

PDLIKEILVTKQKAFIKSRDIQMLKAVVGEGLLTNEKESHLKQRRLIQPAFKKTHIHQYA

QDMIETTNAFIKGWKGEEERNIAADMMNIALGIITKTMFGMEMGQGADVIEQPMEAVMKL

GIKRMRSLSPLPLWVPTQANRQLKKAVKELDDVLFSIISKRRLEDNQSEDLLGMLMKARD

EENGAVMSDQQLRDELMTIFLAGHETTANLLAWTLYLLSEHPSADERLYAEIKEVTNGEA

LLPEHYTKLTYTQNVISESMRLYPPAYVIGRQVEEDIEIGPYLFNKGAMVLISQYVMHRN

ASFYHEPNIFKPERFDHNFLKTLPPFAYFPFGGGPRVCIGNHFAMMEATLALAAIAQNYK

FTLTSSQQKVTPQPLITLRPKGGLMMKVEKRK

>CYP102A30 *Bacillus pumilus* MTCC B6033

MQQTSIIPKPKTYGPLKNIPHIKKGELSQTFWRLADELGPIFQFEFSKATSIFVSSHELA

KEVFDERRFDKFIGSSLNKVRTFSGDGLFTSWTEEPNWRKAHHILMPAFSQQAMKGYHEM

MLDIATQLVQKWQRTGRDEEIEVAEDMTKLTLDTIGLCGFDFRFNSFYKENQHPFIESML

NGLNEAMDQASRLPVADKLMIKRRKEFEQDVDFMKQLVDDIIQERKKQDKTGNDLLSLML

HAKDPETGERLSDENIRYQIITFLIAGHETTSGLLSFAIYFLLKNPDKLKKAVQEADNVL

QGGLPTFKQVQKLSYTRMVLNEALRLWPTAPTFSLYAKEDTVIGGKYPIKKNQSVSVLLP

KIHRDQAVWGEDAEEFKPERFMHPEKIPQHAYKPFGNGQRACIGMQFALHEATMVLAMVL

HNLELIDHTSYELDLKESLTIKPNDFKIKVRPRKQQFFMAPPKEEPTKSTASAEAKIASH

GTPLLVLYGSNLGTAKQMANEFAEDGKAKGFDVTTAPLDDYTRKLPESGAVLIVTASYNG

HPPDHAKQFVDWVTQDEEQDLSNVTFAVFGCGDRNWASTYQRIPRLIDEALERKGAKRVT

DIGEGDAGGDMDEDKETFQKTVFDELAKEFNLTLQEKRQEKPNLSIAYTNELVERPVAKT

YGAFSAVVLKNQELQSEKSTRQTRHIELQLPEGKHYKEGDHIGIVPKNSATLVQRVTDRF

KLDPKQHMILSSEKEASHLPLNQPIQVGELLASHVELQEPVTRTQLRDLAKYTVCPPHRI

ELEQMAGEIYQEAILKKRVTMLDLLEQYEACELPFAHFLALLPGLKPRYYSISSSPKVDE

KRLSITVAVVKGKSWSGRGEYAGVASNYLCGLQEGDEVACFLHEAQAGFGLPPSPEIPMI

MIGPGTGIAPFRGFIQARETWQNEGKPLGEADLYFGCRHPHEDDLYYDEMQLAEQKGVVT

IHRAYSRYEEQKVYVQHFIKNDGAKLIELLDKGAYLYICGDGKVMAPDVEATLIELYQTE

KQCAKETAEQWLTSLANDNRYVKDVWS

>CYP109A10 *Bacillus pumilus* MTCC B6033

MKVKENYVNVIPMKEIRSTDDLLFPFPIYNELRSQGDIRYDETRKCWDLFRYADIQSVLK

QPKVFSSQRGRSTSKTSILTMDPPKHTKMRALVNKAFTPKAIKQIEEKIKDLTHDLLHQV

KDQRTFDIVQDLAAPLPVMIIAELLGAEMKDRELIKKHSDALVAGAKDDSKEAVQAVVDM

QKQAEKELSAYFAHLIQKRKETPADDLISLLIQAEIDGERLSENELLGFCILLLVAGNET

TTNLITNAVRLLTEQQHIAESVRQDLSLIPQLTEETLRYYPPVQAIGRVAAEDVQIAGSH

IEKGDYIISWVASANRDERKFEDPDTFRLDRKSNPHLSFGFGIHFCLGAPLARLEAHLAL

EILLRTFREITCAADQLKPIQSTFVFGVKEFPVQVTRF

>CYP109B5 *Bacillus pumilus* MTCC B6033

MMETTTPSAVQKALLRGKNKQDPYHPFDWYAKMRQESPVHFDEHSQTWSVFTYEEAKRVT

IDKDTFSSQPPQDHRKHSLMKTMVMMDPPKHTRIRSIVSKAFTPRVMKLWEPRIQELMDD

LIAQIEGKEEIDLVQDISYPLPVIVIAELLGVPTEHKQSFKEWSDILVSMPKSEHEKDVV

EWQKTRDQSEADMMAFFADIIEEKRQNLSDDLISLLIQAEEDGDKLSADELIPFCNLLLL

AGNETTTNLISNMIFSLLENPGSYEALAQSPDLIPRAVEEAVRFRAPAPTIVRYVTEDTE

LGGKVLKKGDSVIVFLASANRDERQFPNAHEYDIHRHPNPHIGFGHGIHFCLGAPLARLE

ACTAIKAIQSRYESLELLSYVPMTSSGMYGLKALKLRVTPRS

>CYP102A15 *Bacillus pumilus* SAFR-032

MQQTSIIPKPKTYGPFKNIPHIKKGELSQTFWRLADELGPIFQFEFSKATSIFVSSHELA

KEVFDESRFDKFIGSSLNKVRTFSGDGLFTSWTEEPNWRKAHHILMPAFSQQAMKGYHEM

MLDIATQLVQKWQRTGRDEEIEVAEDMTKLTLDTIGLCGFDFRFNSFYKENQHPFIESML

NGLNEAMEQASRLPVADKLMIKRRKKFEENVDFMKTLVDDIIQERRKQDKTGNDLLSLML

HAKDPETGERLSDENIRYQIITFLIAGHETTSGLLSFAIYFLLKNPEKLKKAVQEADDVL

QGELPTFKQVQKLTYTRMVLNEALRLWPTAPTFSLYAKEDTIIGGKYSIKKNQSVSVLLP

KLHRDQAVWGGDAEEFKPERFLHPEKIPQHAYKPFGNGQRACIGMQFALHEATMVLAMVL

HNMELIDHTSYELNLKESLTIKPNDFKIKVRPRKQQLFMAPPKEETKKSTTIEESKVKSH

GTPLLVLYGSNLGTAQQIANELAEDGKAKGFDMTTAPLDDYARQLPDKGAVLIVTASYNG

HPPDHAKTFVDWVTQDKEKDLTNVTFAVFGCGDRNWASTYQRIPRLIDEALESKGAKRVA

DLGEGDAGGDMDEDKETFQKIVFEQLAKEFQLTFQEKGKETPKLSVAYTNELVERPVAKT

YGAFSAVVLKNEELQSQKSERKTRHIELRLPEGKKYKEGDHIGIVPKNRDVLVQRVIDRF

NLDPKQHIKLSSEKEANHLPLGQPIQIRELLASHVELQEPATRTQLRELASYTVCPPHRV

ELEQMAGEAYQEAILKKRVTMLDLLDQYEACEMPFAHFLALLPGLKPRYYSISSSPKIDE

KRVSITVAVVKGKAWSGRGEYAGVASNYLCDLQKGEEVACFLHEAQAGFQLPPSSETPMI

MIGPGTGIAPFRGFVQAREVWQKEGKRLGEAHLYFGCRHPHEDDLYFEEMQLAAQKGVVH

IRRAYSRHKDQKVYVQHLLKEDGGMLIKLLDEGAYLYVCGDGKVMAPDVESTLIDLYQHE

KQCSKEDAENWLTTLANNNRYVKDVWS

>CYP109A8 *Bacillus pumilus* SAFR-032

MKVKENYINVIPMKEIRSTDDLLFPFPIYNELRAQSDIRYDETRKCWDLFRYADIQSVLK

QPKVFSSQRGRSTTKTSILTMDPPKHTKMRALVNKAFTPKAIKQLEDNIRDLTHDLLQQV

KDQRTFDIVQDLAAPLPVMIIAELLGAEVKDRAFIKKHSDALVAGAKDESKEAIQAVVDM

QKRAEEELSAYFTHLIQKRKESPTDDLISLLIQAEIDGERLTEDELLGFCILLLVAGNET

TTNLITNAVRLLTEQPHIAEAVRQDPSLIPQLTEETLRYYPPVQAIGRIAAEDVEIAGSK

IKKGDYLISWVASANRDEQKFDDPDTFRLDRKSNPHMSFGFGIHFCLGAPLARLESNIAL

EVLLQTFQDITCVADQLKPIQSTFVFGVREFPVQVTRF

>CYP109B6 *Bacillus pumilus* SAFR-032

METTSPSAVQKTLLRGKNKQDPYHPFDWYANMRKTSPVHFDEASRTWSVFTYEEARRVTI

DKDTFSSQPPKNQRKHSLMKTMVMMDPPNHTRIRSIVSKAFTPRVMKLWEPRIHELMDEL

MAQLEGKKEIDLVQDISYPLPVIVIAELLGVPSEHKQSFKEWSDILVSMPKSESEKDVAE

WQKTRDKGEADMMAFFADTIEKKRHNLGDDLISLIIQAEENGDKLSADELIPFCNLLLLA

GNETTTNLISNMMFSLLEQPGAYEALAQSPELIPRAVEEAVRFRAPAPAIVRYVTKDTEL

GEKVLKRGDNVIVFLASANRDERQFSNAHEYDIHRHPNPHIGFGHGIHFCLGAPLARLEA

CTAIKIFIERYEALELLSYVPMTSSSMYGLKELKLRVTPRS

>CYP102A29 *Bacillus pumilus* SH-B9

MHQSSIIPKPKTYGPFKNIPHIKKGELSQTFWRLADELGPIFQFEFSNATSIFVSSHELV

KEVCDESRFDKFIGTSLNKVRAFAGDGLFTSWTEEPNWRKAHQILMPAFSQQAMKGYHEM

MLDIATQLVQKWQRTGRDEEIEVAEDMTKLTLDTIGLCGFDFRFNSFYKENQHPFIESML

KGLNEAMEQSSRLPIADKLMIKRRKEFDQHVDFMSQLVDDIIQERKKQDKTGDDLLSLML

HAEDPETGERLSDENIRYQIITFLIAGHETTSGLLSFAIYFLLKNPEKLKKAIQEADEVL

QGGLPTFKQVQKLSYTRMVLNEALRLWPTAPTFSLYAKEDTVIGGKYPIQKNQSVSVLLP

KLHRDPAVWGEDSEDFKPERFMHPEKIPQHAYKPFGNGQRACIGMQFALHEATMVLAMVL

HNLELIDHTSYELDLKESLTIKPNDFKIKVLPRKQQFFMAPPKEEPKKSTKSSDSKVASH

GTSLLVLYGSNLGTAKKIANELVEDGKAKGFDVTTASLDDFTRRLPDTGAVLIVTASYNG

HPPDNAKQFVDWLTQDEEQDLSNVTFAVFGCGDRNWASTYQRIPRLIDEALERKGAKRVT

DIGEGDAGGDMDEDKETFQKTVFEQLAKEFKLTFQEKGKEKPNLSVAYTNELVERPVAKI

YDAFSAVVLKNEELQSEKSTRQTRHIELKLPAGKVYKEGDHIGIVPKNSDALVQRVTHRF

KLDPEQHIKLSSEKEASHLPLDQPIPIKELLASHVELQEPATRTQLRELAAHTVCPPHRV

ELEQMAGEAYQDAILKKRVTMLDLLDQYEACELPFVHFLALLPGLKPRYYSISSSPKVEE

EKVSITVAVVKGKAWNGRGEYAGVASTYLCGLKEGEEVACFLHEAQAGFQLPPSSELPMI

MIGPGTGIAPFRGFVQAREVWQKEGKPLGEAHLYFGCRHPHEDDLYFEEMQLAAQKGVVH

IHRAYSRYNEQKIYVQHLLKEDGGTLIKLLDQGAYLYVCGDGKVMAPDVEATLIDLYQQE

KQCSKEVAENWLTTLANNNRYVKDVWS

>CYP109A8 *Bacillus pumilus* SH-B9

MKVKENYINVIPMKEIRSTDDLLFPFPIYNELRAESDIRYDETRKCWDLFRYADIQSVLK

QPKVFSSQRGRSTTKTSILTMDPPKHTKMRALVNKAFTPKAIKQLEDNIKDLTHDLLQQV

KDQRTFDIVEDLAAPLPVMIIAELLGAEIKDRAFIKKHSDALVAGAKDESKEAIQAVVDM

QKRAEEELSAYFAHLIQKRKEAPADDLISLLIQAEIDGERLTENELLGFCILLLVAGNET

TTNLITNAVRLLTEQPHIAEAVRQDPSLIPQLTEETLRYYPPVQAIGRIAAEDVEIAGSK

IKKGDYLINWVASANRDEQKFDDPDTFRLNRKSNPHMSFGFGIHFCLGAPLARLESNIAL

EVLLQSYQDITCAADQLKPIQSTFVFGVKEFPVQITRF

>CYP109B6 *Bacillus pumilus* SH-B9

METTTPSAVQKTLLRGKNKQDPYHPFDWYANMRQTSPVHFDEASRTWSVFTYEEAKRVTI

DKDTFSSQPPKNQRKHSLMKTMVMMDPPNHTRIRSIVSKAFTPRVMKLWEPRIQELMDEL

MAQIEGKKEIDLVQDISYPLPVIVIAELLGVPSEHKQSFKEWSDILVSMPKSENEEDVAE

WQKTRDKGEADMMAFFADTIEKKRHNLGDDLISLIIQAEENGEKLSADELIPFCNLLLLA

GNETTTNLISNMIFSLLEQPGAYEALSQSPELIPRAVEEAVRFRAPAPAIVRYVTKDTEL

GGKVLKKGDNVIVFLASANRDERQFSNAHEYDIHRHPNPHIGFGHGIHFCLGAPLARLEA

CTAIKFLIERYEALELLSYVPMTSSSMYGLKELKLRVTPRS

>CYP102A8 *Bacillus thuringiensis* Al Hakam

MLFERVFLMDKKVSAIPQPKTYGPLGNLPLIDKDKPTLSFIKLAEEYGPIFRIQTLSDTI

IVVSGHELVAEVCDETRFDKSIEGALAKVRAFAGDGLFTSETQEPNWQKAHNILMPTFSQ

RAMKDYHAMMVDIAVQLVQKWARLNPNENVDVPEDMTRLTLDTIGLCGFNYRFNSFYRET

PHPFITSMTRALDEAMHQLQRLDIEDKLMWRTKRQFQHDIQSMFSLVDNIIAERKSSENQ

EENDLLSRMLNVQDPETGEKLDDENIRFQIITFLIAGHETTSGLLSFAIYFLLKNPDKLK

KAYEEVDRVLTDPTPTYQQVMKLKYIRMILNESLRLWPTAPAFSLYAKEDTVIGGKYPIK

KGEDRISVLIPQLHRDKDAWGDNVEEFQPERFEELDKVPHHAYKPFGNGQRACIGMQFAL

HEATLVMGMLLQHFEFIDYEDYQLDVKQTLTLKPGDFKIRIVPRNQTISHTTVLAPTEEK

LKNHEIKQQVQKTPSIIGADNLSLLVLYGSDTGVAEGIARELADTASLEGVQTEVVALND

RIGSLPKEGAVLIVTSSYNGKPPSNAGQFVQWLEELKPDELKGVQYAVFGCGDHNWASTY

QRIPRYIDEQMAQKGATRFSTRGEADASGDFEEQLEQWKQRMWSDAMKAFGLELNKNMEK

ERSTLSLQFVSRLGGSPLARTYEAVYASILENRELQSSSSERSTRHIEISLPEGATYKEG

DHLGVLPINSEKNVNRILKRFGLNGKDQVILSASGRSVNHIPLDSPVRLYDLLSYSVEVQ

EAATRAQIREMVAFTACPPHKKELESLLEDGIYHEQILKKRISMLDLLEKYEACEIRFER

FLELLPALKPRYYSISSSPLVAQDRLSITVGVVNAPAWSGEGTYEGVASNYLAQRHNKDE

IICFIRTPQSNFQLPENPETPIIMVGPGTGIAPFRGFLQARRVQKQKGMKVGEAHLYFGC

RHPEKDYLYRTELENDEREGLISLHTAFSRLEGHPKTYVQHVIKEDRIHLISLLDNGAHL

YICGDGSKMAPDVEDTLCQAYQEIHQASEQEARNWLDRLQDEGRYGKDVWAGI

>CYP106B1 *Bacillus thuringiensis* Al Hakam

MDSPENVILVHEISKLKTKEELWNPYEWYQFMRDNHPVHYDDEQDVWNVFLYDDVNRVLS

DYRLFSSRRERRQFAIPPLETRININSTDPPEHRNVRSIVSKAFTPRSLEQWKPRIQSIA

NELVKDIENCSEVDIVEQFAAPLPVTVISDLLGVPTTDRKKIKTWSDILFMPYSKEKFND

LDAKKGIALNEFKAYLLPIVQEKRYYLTDDIISDLIRAEYEGERLTDEEIVTFSLGLLAA

GNETTTNLIINSFYCFLVDSPATYKELREKPKLISKAIEEVLRYRFPVTLARRITEDTNI

FGPLMKKDQMVVAWVSAANLDEKKFSQASKFNIHRIGNEKHLTFGKGPHFCLGAPLARLE

AEIALTTFINAFEKIALSPSFNIEQCILENEQTLKFLPIRLKPQ

>CYP107J2 *Bacillus thuringiensis Al Hakam*

MAMKNKVGLRIEDGINLASAQFKEDAYEIYKESRKVQPVLFVNKTELGAEWLITRYEDAL

PLLKDSRLKKDPANVFSQDTLNVFLTVDNGDHLTTHMLNSDPPNHNRLRSLVQKVFTPKM

IAQLEGRIQDIADDLLNEVERKGSLNLVDDYSFPLPIIVISEMLGIPKEDQAKFRIWSHA

VIAYPETPEEIKETEKQLSEFITYLQYLVDMKRKEPKEDLVSALILAESEGHKLSARELY

SMIMLLIVAGHETTVNLITNTVLALLENPNQLQLLKENPKLIDAAIEEGLRYYSPVEVTT

SRWADEPFQIHDQTIEKGDMVVIALASANRDETVFENPEVFDITRENNRHIAFGHGSHFC

LGAPLARLEAKIAITTLFERMPELQIKGNREDIKWQGNYLMRSLEELPLTF

>CYP102A5 *Bacillus thuringiensis* Bt407

MEKKVSAIPQPKTYGPLGNLPLIDKDKPTLSFIKIAEEYGPIFQIQTLSDTIIVVSGHEL

VAEVCDETRFDKSIEGALAKVRAFAGDGLFTSETHEPNWKKAHNILMPTFSQRAMKDYHA

MMVDIAVQLVQKWARLNPNENVDVPGDMTRLTLDTIGLCGFNYRFNSFYRETPHPFITSM

TRALDEAMHQLQRLDIEDKLMWRTKRQFQHDIQSMFSLVDNIIAERKSNGNQEENDLLSR

MLNVPDPETGEKLDDENIRFQIITFLIAGHETTSGLLSFAIYFLLKNPDKLKKAYEEVDR

VLTDPTPTYQQVMKLKYIRMILNESLRLWPTAPAFSLYAKEDTVIGGKYPIKKGEDRISV

LIPQLHRDKDAWGDNVEEFQPERFEELDKVPHHAYKPFGNGQRACIGMQFALHEATLVMG

MLLQHFELIDYQNYQLDVKQTLTLKPGDFKIRILPRKQTISHPTVLTPTEDKLKNDEIKQ

HVQKPPSIIGADNLSLLVLYGSDTGVAEGIARELADTASLEGVRTEVVALNDRIGSLPKE

GAVLIVTSSYNGKPPSNAGQFVQWLEELKPDELKGVQYAVFGCGDHNWASTYQRIPRYID

EQMAQKGATRFSKRGEADASGDFEEQLEQWKQNMWSDAMKAFGLELNKNIEKERSTLSLQ

FVSRLGGSPLARTYEAVYASILENRELQSPSSDRSTRHIEVSLPEGATYKEGDHLGVLPV

NSEKNINRILKRFGLNGKDQVILSASGRSINHIPLDSPVSLLDLLSYSVEVQEAATRAQI

REMVTFTACPPHKKELEALLEEGVYHEQILKKRISMLDLLEKYEACEIRFERFLELLPAL

KPRYYSISSSPLVAHNRLSITVGVVNAPAWSGEGTYEGVASNYLAQRHNKDEIICFIRTP

QSNFELPKDPETPIIMVGPGTGIAPFRGFLQARRVQKQKGINLGQAHLYFGCRHPEKDYL

YRTELENDERDGLISLHTAFSRLEGHPKTYVQHLIKQDRINLISLLDNGAHLYICGDGSR

MAPDVEDTLCQAYQEIHEVSEQEARNWLDRVQDEGRYGKDVWAGI

>CYP106B4 *Bacillus thuringiensis* Bt407

MASPENVILVHEISKLKTKEELWNPYEWYRLMRDNHPVHYDEEQDVWNVFLYDDVNRVLS

DYRLFSSRRERRQFSIPPLETRININSTDPPEHRNVRSIVSKAFTPRSLEQWKPRIQAIA

DELVQHIEKCSEVNIVEQFAAPLPVTVISDLLGVPTTDRKKIKEWSDILFMPYSKEKFND

LDAEKGIALHEFKAYLLPIVQEKRYHLTDDIISDLIRAEYEGERLTDEEIVTFSLGLLAA

GNETTTNLIINSFYCFLVDSPGIYEELRKEPNLILKAIEEVLRYRFPVTLARRITEDTNI

FGPFMKKNQMIVAWVSAANLDEKKFSQASQFNVHRTGNEKHLTFGKGPHFCLGAPLARLE

AEIALTTFINAFEKIELSPSFCLEKCILENEQTLKYLPIRLKAK

>CYP107J3 *Bacillus thuringiensis* Bt407

MSMKNKVGLSIEDGINLASAQFKEDAYEIYKESRKKQPILFVNQVEIGKEWLITRYEDAL

PLLKDNRLKKDWTNVFSQDIKNMYLSVDNSDHLTTHMLNSDPPNHSRLRSLVQKAFTPKM

IAQLDGRIERIADDLISDIERKGTLNLVDDYSFPLPIIVISEMLGIPKEDQAKFRIWSHA

VIASPETPEEIKETEKQLSEFITYLQYLVDIKRKEPKEDLVSALILAESEGHKLSARELY

SMIMLLIVAGHETTVNLITNTVLALLENPNQLQLLKDNPKLIDSAIEEGLRYYSPVEVTT

ARWAAEPFQIHHQTIQKGDMVIIALASANRDETVFENPEIFDITRENNRHIAFGHGSHFC

LGAPLARLEAKIAITTLFNRMPELKIKGNREEIKWQGNYLMRSLEELPLTF

>CYP102A8*Bacillus thuringiensis* HD1011

MDKKVSAIPQPKTYGPLGNLPLIDKDKPTLSFIKLAEEYGPIFRMQTLSDTIIVVSGHEL

VAEVCDETRFDKSIEGALAKVRAFAGDGLFTSETQEPNWQKAHNILMPTFSQRAMKDYHA

MMVDIAVQLVQKWARLNPNENVDVPEDMTRLTLDTIGLCGFNYRFNSFYRETPHPFITSM

TRALDEAMHQLQRLDIEDKLMWRTKRQFQHDIQSMFSLVDNIIAERKSSENQEENDLLSR

MLNVQDPETGEKLDDENIRFQIITFLIAGHETTSGLLSFAIYFLLKNPDKLKKAYEEVDR

VLTDPTPTYQQVMKLKYIRMILNESLRLWPTAPAFSLYAKEDTVIGGKYPIKKGEDRISV

LIPQLHRDKDAWGDNVEEFQPERFEELDRVPHHAYKPFGNGQRACIGMQFALHEATLVMG

MLLQHFEFIDYEDYQLDVKQTLTLKPGDFKIRIVPRNQTISHTTVLAPTEEKLKKHEIKQ

QVQKTPSIIGADNLSLLVLYGSDTGVAEGIARELADTASLEGVQTEVVALNDRIGSLPKE

GAVLIVTSSYNGKPPSNAGQFVQWLEELKPDELKGVQYAVFGCGDHNWASTYQRIPRYID

EQMAQKGATRFSTRGEADASGDFEEQLEQWKQRMWSDAMKAFGLELNKNMEKERSTLSLQ

FVSRLGGSPLARTYEAVYASILENRELQSSSSERSTRHIEISLPEGATYKEGDHLGVLPI

NSEKNVNRILKRFGLNGKDQVILSASGRSVNHIPLDSPVRLYDLLSYSVEVQEAATRAQI

REMVTFTACPPHKKELESLLEDGVYQEQILKKRISMLDLLEKYEACEIRFERFLELLPAL

KPRYYSISSSPLVAQDRLSITVGVVNAPAWSGEGTYEGVASNYLAQRHNKDEIICFIRTP

QSNFQLPENPETPIIMVGPGTGIAPFRGFLQARRVQKQKGMNVGEAHLYFGCRHPEKDYL

YRTELENDERDGLISLHTAFSRLEGHPKTYVQHVIKEDRIHLISLLDNGAHLYICGDGSK

MAPDVEDTLCQAYQEIHEVSEQEARNWLDRVQEEGRYGKDVWAGI

>CYP106B1 *Bacillus thuringiensis* HD1011

MASPENVILVHEISKLKTKEELWNPYEWYQFMRDNHPVHYDDEQDVWNVFLYDDVNRVLS

DYSLFSSRRERRQFAIPPLETRININSTDPPEHRNVRSIVSKAFTPRSLEQWKPRIQSIA

NELVKDIENCSEVDIVEQFAAPLPVTVISDLLGVPTTDRKKIKAWSDILFMPYSKEKFND

LDAEKGIALNEFKAYLLPIVQEKRYHLTDDIISDLIRAEYEGERLTDEEIVTFSLGLLAA

GNETTTNLIINSFYCFLVDSPATYKEVREKPKLISKAVEEVLRYRFPVTLARRITEDTNI

FGPLMKKDQMVVAWVSAANLDEKKFSQASKFNIHRIGNEKHLTFGKGPHFCLGAPLARLE

AEIALTTFINAFEKIALSPSFNIEQCILENQQTLKFLPIRLKPQ

>CYP107J2 *Bacillus thuringiensis* HD1011

MAMKNKVGLRIEDGINLASAQFKEDAYEIYKESRKVQPVLFVNKTELGAEWLITRYEDAL

PLLKDNRLKKDPANVFSQDTLNVFLTVDNSDYLTTHMLNSDPPNHNRLRSLVQKVFTPKM

IAQLEGRIQDIADDLLNEVERKGSLNLVDDYSFPLPIIVISEMLGIPKEDQAKFRIWSHA

VIAYPETPEEIKETEKQLSEFITYLQYLVDMKRKEPKEDLVSALILAESEGHKLSARELY

SMIMLLIVAGHETTVNLITNTVLALLENPNQLQLLKENPKLIDAAIEEGLRYYSPVEVTT

SRWADEPFQIHDQTIEKGDMVVIALASANRDETVFENPEVFDITRENNRHIAFGHGSHFC

LGAPLARLEAKIAITTLFERMPELQIKGNREDIKWQGNYLMRSLEELPLTF

>CYP102A5 *Bacillus thuringiensis* HD-789

MEKKVSAIPQPKTYGPLGNLPLIDKDKPTLSFIKLAEEYGPIFRIQTLSDAIIVVSGHEL

VAEVCDETRFDKSIEGALAKVRAFAGDGLFTSETHEPNWKKAHNILMPTFSQRAMKDYHA

MMVDLAVQLVQKWARLNPNEDVDVPEDMTRLTLDTIGLCGFNYRFNSFYRETPHPFITSM

SRALDEAMHQLQRLDIEDKLMWRTKRQFQHDIQSMFSLVDNIIAERKSSGDQEENDLLSR

MLNVQDPETGEKLDDENIRFQIITFLIAGHETTSGLLSFAIYFLLKNPDKLKKAYEEVDR

ILTDPTPTYQQVMKLKYIRMILNESLRLWPTAPAFSLYAKEDTVIGGKYPIKKGEDRISV

LIPQLHRDKDAWGDNVEEFQPERFEELDKVPHHAYKPFGNGQRACIGMQFALHEATLVMG

MLLQHFELIDYQNYQLDVKQTLTLKPGDFKIRILPRKQTISHPTVLAPTEDKLKNHEIKQ

HVQKTPSIIGADNLSLLVLYGSDTGVAEGIARELADTASLEGVQTEVVALNDRIGSLPKE

GAVLIVTSSYNGKPPSNAGQFVQWLEELKPDELKGVQYAVFGCGDHNWASTYQRIPRYID

EQMAQKGATRFSKRGEADASGDFEEQLEQWKQGMWSDAMKAFGLEFNKNMEKERSTLSLQ

FVSRLGGSPLARTYEAVYATILENRELQSSSSDRSTRHIEVSLPEGATYQEGDHLGVLPI

NSEKNVNRILKRFGLNGKDQVILSASGRSINHIPLDSPVSLLDLLSYSVEVQEAATRAQI

REMVTFTACPPHKKELEALLEEGVYHEQILKKRISMLDLLEKYKACEIRFERFLELLPAL

KPRYYSISSSPLVAQNRLSITVGVVNAPAWSGEGTYEGVASNYLAQRHNKDEIICFIRTP

QSNFELPKDPETPIIMVGPGTGVAPFRGFLQARRVQKQKGINLGQAHLYFGCRHPEKDYL

YRTELENDERDGLISLHTAFSRLEGHPKTYVQHLIKQDSINLISLLDNGAHLYICGDGSK

MAPDVEDTLCQAYQEIHEVSEQEARNWLDRVQDEGRYGKDVWAGI

>CYP106B4 *Bacillus thuringiensis* HD-789

MASPENVILVHEISKLKTKEELWNPYEWYRLMRDNHPVHYDEEQDVWNVFLYDDVNRVLS

DYRLFSSRRERRQFSIPPLETRININSTDPPEHRNARSIVSKAFTPRSLEQWKPRIQAIA

DELVQHIEKYSEVNIVEQFAAPLPVTVISDLLGVPTTDRKKIKEWSDILFMPYSKEKFND

LDAEKGIALNEFKAYLLPIVQEKRYHLTDDIISDLIRAEYEGERLTDEEIVTFSLGLLAA

GNETTTNLIINSFYCFLVDSPGIYEDLRKKPSLISKAIEEVLRYRFPVTLARRITEDTNI

FGPLMKKDQMIVAWVSAANLDEKKFSHPSQFNVHRIGNEKHLTFGKGPHFCLGAPLARLE

AEIALTTFINAFEKIELSPSFCLEKCILENEQTLKYLPIRLKAK

>CYP107J2 *Bacillus thuringiensis* HD-789

MSMKNKVGLKIEEGINLASAQFKEDAYEIYKESRKMQPILFVNKTELGAEWLITRYEDAL

PLLKDSRLKKDPANVFSQDTLNVFLTVDNSDYLTTHMLNSDPPNHNRLRSLVQKAFTPKM

ITQLEGRIQDIAGDLLNEVERKGSLNLVDDYSFPLPIIVISEMLGIPKEDQAKFRIWSHA

VIAYPETPEEIKETEKQLSEFITYLQYLVDIKRKEPKEDLVSALILAESEGHKLSARELY

SMIMLLIVAGHETTVNLITNTVLALLENPDQLQLLKENPKLIDAAIEEGLRYYSPVEVTT

SRWADEPFQIHDQTIEKGDMVVIALASANRDETVFENPEVFDITRENNRHIAFGHGSHFC

LGAPLARLEAKIAITTLFNRMPELQIKGNREEIKWQGNYLMRSLEELPLTF

>CYP102A5 *Bacillus thuringiensis* MC28

MLFERVFLMDKKVSAIPQPKTYGPLGNLPLIDKDKPTLSFIKIAEEYGPIFQIQTLSDTI

IVVSGHELVAEVCDETRFDKSIDGALAKVRAFAGDGLFTSETDEPNWKKAHNILMPTFSQ

RAMKDYHGMMVDIAVQLVQKWARLNPNENVDVPEDMTRLTLDTIGLCGFNYRFNSFYRET

SHPFITSMSRALDEAMHQLQRLDIEDKLMWRTKRQFQHDIQSMFSLVDNIIAERKSNGNQ

EENDLLARMLNVQDPETGEKLDDENIRFQIITFLIAGHETTSGLLSFAIYFLLKNPDKLK

KAYEEVDRVLTDPTPTYQQVMKLKYIRMILNESLRLWPTAPAFSLYAKEDTVIGGKYPIK

KGEDRISVLIPQLHRDKDAWGDNVEEFQPERFEEPDKVPHHAYKPFGNGQRACIGMQFAL

HEATLVMGMLLQHFELIDYQNYQLDVKQTLTLKPGDFKIRILARNQTISHPTVLAPIEEK

QKDHEIKQQVQKTPSIIGADNLSLLVLYGSDTGVAEGIARELADTASLEGVQTEVVALNE

RIGSLPKEGAVLIVTSSYNGKPPSNAGQFVQWLEELKSDELKGVQYAVFGCGDHNWASTY

QRIPRYIDEQMAQKGATRFSKRGEADASGDFEEQLEQWKQSMWSDAMRAFGLELNKNMEK

ERSTLSLQFVSRLGGSPLARTYEAVYASILENRELQSSSSDRSTRHIEVSLPEGATYQEG

DHLGVLPINSEKNVNRILKRFGLNGKDQVILSASGRSVNHIPLDSPVSLFDLISYSVEVQ

EAATRAQIREMVTFTACPPHKKELESLLEEGVYHERILKKRISMLDLLEKYEACEIRFER

FLELLPALKPRYYSISSSPLVAQDRLSITVGVVNAPAWSGEGTYEGVASNYLAQRHNKDE

IICFIRTPQSNFQLPENPETPIIMVGPGTGIAPFRGFLQARRVQKQKGINLGQAHLYFGC

RHPEKDYLYRTELENDERDGLISLHTAFSRLEGHPKTYVQHLIKQDRINLISLLDNGAHL

YICGDGSKMAPDVEDTLCQAYEEIHEVSEQEARNWLDHLQHEGRYGKDVWTGI

>CYP106B5 *Bacillus thuringiensis* MC28

MDSPENVILVHEISKLKTKEELWNPYGWYQFMRDNHPVHYDEEQDVWNVFLYEDVNRVLS

DYRLFSSRRERRQFSIPPLETRININSTDPPEHRNVRSIVSKAFTPRSLEQWKPRIKAIA

DELVKNIKKYDEVNIVNQFAAPLPVTVISDLLGVPTTDRKQIKEWSDILFMPYSKEKFND

LDAEKGIALNEFNAYLLPIVQEKRYHLTDDIISDLIRAEYEGERLTDEEIVTFSLGLLAA

GNETTTNLIINSFYCFLVDSPGIYQELREEPKLVAKAIEEVLRYRFPVTLARRITEDTNI

FGPLMKKDQMIVAWVSAANLDENKFSHASKYNLHRIGNEKHLTFGKGPHFCLGAPLARLE

AEIALSTFINAFEKIELSPSFNLERCILKNKQTLKYLPIRLKTQ

>CYP107J3 *Bacillus thuringiensis* MC28

MSMKNKVGLRIEDGINLASAQFKKDAYEIYKESRKVQPILFVNQIEIGKEWLITRYEDAL

PLLKDNRLKKDQANVFPQDTKNMYLSVDNSDHLTTHMLNSDPPNHSRLRSLVQKAFTPKM

ISQLDGRIQRIADDLISEIERKGTLNLVDDYSFPLPIIVISEMLGIPKEDQAKFRIWSHA

VIASPETPEEIKETEKQLSEFITYLQYIVDVKRKNPKEDLVSALILAENEGQKLSARELY

SMIMLLIVAGHETTVNLITNTVLALLENPKQLQLLKDNPKLIDSAIEEGLRYYSPVEVTT

ARWAAEPFQIHDQTIQKGDMVIIALASANRDETVFENPEVFDIMRENNRHIAFGHGSHFC

LGAPLARLEAKIAITTLFKRMPELQIKGDREDIKWQGNYLMRSLEELPLTF

>CYP102A5 *Bacillus thuringiensis serovar chinensis* CT-43

MEKKVSAIPQPKTYGPLGNLPLIDKDKPTLSFIKIAEEYGPIFQIQTLSDTIIVVSGHEL

VAEVCDETRFDKSIEGALAKVRAFAGDGLFTSETHEPNWKKAHNILMPTFSQRAMKDYHA

MMVDIAVQLVQKWARLNPNENVDVPGDMTRLTLDTIGLCGFNYRFNSFYRETPHPFITSM

TRALDEAMHQLQRLDIEDKLMWRTKRQFQHDIQSMFSLVDNIIAERKSNGNQEENDLLSR

MLNVPDPETGEKLDDENIRFQIITFLIAGHETTSGLLSFAIYFLLKNPDKLKKAYEEVDR

VLTDPTPTYQQVMKLKYIRMILNESLRLWPTAPAFSLYAKEDTVIGGKYPIKKGEDRISV

LIPQLHRDKDAWGDNVEEFQPERFEELDKVPHHAYKPFGNGQRACIGMQFALHEATLVMG

MLLQHFELIDYQNYQLDVKQTLTLKPGDFKIRILPRKQTISHPTVLTPTEDKLKNDEIKQ

HVQKPPSIIGADNLSLLVLYGSDTGVAEGIARELADTASLEGVRTEVVALNDRIGSLPKE

GAVLIVTSSYNGKPPSNAGQFVQWLEELKPDELKGVQYAVFGCGDHNWASTYQRIPRYID

EQMAQKGATRFSKRGEADASGDFEEQLEQWKQNMWSDAMKAFGLELNKNIEKERSTLSLQ

FVSRLGGSPLARTYEAVYASILENRELQSPSSDRSTRHIEVSLPEGATYKEGDHLGVLPV

NSEKNINRILKRFGLNGKDQVILSASGRSINHIPLDSPVSLLDLLSYSVEVQEAATRAQI

REMVTFTACPPHKKELEALLEEGVYHEQILKKRISMLDLLEKYEACEIRFERFLELLPAL

KPRYYSISSSPLVAHNRLSITVGVVNAPAWSGEGTYEGVASNYLAQRHNKDEIICFIRTP

QSNFELPKDPETPIIMVGPGTGIAPFRGFLQARRVQKQKGINLGQAHLYFGCRHPEKDYL

YRTELENDERDGLISLHTAFSRLEGHPKTYVQHLIKQDRINLISLLDNGAHLYICGDGSR

MAPDVEDTLCQAYQEIHEVSEQEARNWLDRVQDEGRYGKDVWAGI

>CYP106B4 *Bacillus thuringiensis serovar* *chinensis* CT-43

MASPENVILVHEISKLKTKEELWNPYEWYRLMRDNHPVHYDEEQDVWNVFLYDDVNRVLS

DYRLFSSRRERRQFSIPPLETRININSTDPPEHRNVRSIVSKAFTPRSLEQWKPRIQAIA

DELVQHIEKCSEVNIVEQFAAPLPVTVISDLLGVPTTDRKKIKEWSDILFMPYSKEKFND

LDAEKGIALHEFKAYLLPIVQEKRYHLTDDIISDLIRAEYEGERLTDEEIVTFSLGLLAA

GNETTTNLIINSFYCFLVDSPGIYEELRKEPNLILKAIEEVLRYRFPVTLARRITEDTNI

FGPFMKKNQMIVAWVSAANLDEKKFSQASQFNVHRTGNEKHLTFGKGPHFCLGAPLARLE

AEIALTTFINAFEKIELSPSFCLEKCILENEQTLKYLPIRLKAK

>CYP107J3 *Bacillus thuringiensis serovar* *chinensis* CT-43

MSMKNKVGLSIEDGINLASAQFKEDAYEIYKESRKKQPILFVNQVEIGKEWLITRYEDAL

PLLKDNRLKKDWTNVFSQDIKNMYLSVDNSDHLTTHMLNSDPPNHSRLRSLVQKAFTPKM

IAQLDGRIERIADDLISDIERKGTLNLVDDYSFPLPIIVISEMLGIPKEDQAKFRIWSHA

VIASPETPEEIKETEKQLSEFITYLQYLVDIKRKEPKEDLVSALILAESEGHKLSARELY

SMIMLLIVAGHETTVNLITNTVLALLENPNQLQLLKDNPKLIDSAIEEGLRYYSPVEVTT

ARWAAEPFQIHHQTIQKGDMVIIALASANRDETVFENPEIFDITRENNRHIAFGHGSHFC

LGAPLARLEAKIAITTLFNRMPELKIKGNREEIKWQGNYLMRSLEELPLTF

>CYP102A8 *Bacillus thuringiensis serovar finitimus* YBT-020

MDKKVSAIPQPKTYGPLGNLPLIDKDKPTLSFIKLAEEYGPIFQIQTLSDTIIVVSGHEL

VAEVCDETRFDKSIEGALAKVRAFAGDGLFTSETHEPNWKKAHNILMPTFSQRAMKDYHA

MMVDIAVQLVQKWARLNPNENVDVPGDMTRLTLDTIGLCGFNYRFNSFYRETSHPFITSM

TRALDEAMHQLQRLDIEDKLMWRTKRQFQHDIQSMFSLVDNIIAERKSSGNQEENDLLSR

MLHVQDPETGEKLDDENIRFQIITFLIAGHETTSGLLSFAIYFLLKNPDKLKKAYEEVDR

VLTDPTPTYQQVMKLKYIRMILNESLRLWPTAPAFSLYAKEDTVIGGKYPIKKGEDRISV

LIPQLHRDKDAWGDNVEEFQPERFEELDKVPHHAYKPFGNGQRACIGMQFALHEATLVMG

MLLQHFEFIDYEDYKLDVKQTLTLKPGDFKIRIVPRNQTISHTTVLAPTEEKLKNHETKQ

QVQKTPSIIGADNLSLLVLYGSDTGVAEGIARELADTASLEGVQTEVAALNDRIGSLPKE

GAVLIVTSSYNGKPPSNAGQFVQWLEELKPDELKGVQYAVFGCGDHNWASTYQRIPRYID

EQMAQKGATRFSKRGEADASGDFEEQLEQWKQSMWSDAMKAFGLELNKNMEKERSTLSLQ

FVSRLGGSPLARTYEAVYASILENRELQSSSSERSTRHIEISLPEGATYKEGDHLGVLPI

NSEKNVNRILKRFGLNGKDQVILSASGRSVNHIPLDSPVRLFDLLSYSVEVQEAATRAQI

REMVTFTVCPPHKKELESLLEDGVYHEQILKKRISMLDLLEKYEACEIRFERFLELLPAL

KPRYYSISSSPLVAQDRLSITVGVVNAPAWSGEGTYEGVASNYLAQRHNKDEIICFIRTP

QSNFQLPENPETPIIMVGPGTGIAPFRGFLQARRVQKQKGMNLGEAHLYFGCRHPEKDYL

YRTELENDEREGLISLHTAFSRLEGHPKTYVQHVIKEDRIHLIALLDNGAHLYICGDGSK

MAPDVEDTLCEAYQEIHEVSEQEARNWLERVQDEGRYGKDVWAGI

>CYP107J2 *Bacillus thuringiensis serovar finitimus* YBT-020

MSMKNKVGLRIEDGINLASAQFKEDAYEIYKESRKMQPILFVNKTELGAEWLITRYEDAL

PLLKDNRLKKDPANVFSQDTLNVFLTVDNSDYLTTHMLNSDPPNHNRLRSLVQKAFTPKM

IAQLEGRIRDIADDLLNEVERKGSLNLVDDYSFPLPIIVISEMLGIPKEDQAKFRIWSHA

VIAYPETPEEIKETEKQLSEFITYLQYLVDVKRQEPKEDLVSALILAESEGHKLSARELY

SMIMLLIVAGHETTVNLITNTVLALLENPDQLQLLKENPKLIDVAIEEGLRYYSPVEVTT

SRWADEPFQIHDRTIEKGDMVVIALASANRDETVFENPEVFDITRENNRHIAFGHGSHFC

LGAPLARLEAKIAITTLFNRMPDLQIKGSREEIKWQGNYLMRSLEELPLTF

>CYP109T5 *Bacillus thuringiensis serovar finitimus* YBT-020

MKTPYESIIMIPTNKLIGKKALEDPYDPFAWYKEMREEEPICFNPQVDMWNVFLYDDVKR

VLEDKEYFSNIMPEKKKPPFPQSILGMDQPKHTQIRSIVNRSFTPKALEVWEPRIQEITE

HILTQLSNRENFDIVHELFYPLPVIVIAEMLGVSTNDMDRFKNWSDIIVSSPSHDDPDYL

KEFFHTRLQAENELENFFEEILQLNRGNANKDSNDIISLLVQSEVDEKISGKEIVSFCKL

LLVAGNETTTNLLGNLLYCLIEHPDVYKQIQQDTALIPKAIEEVLRYRSPAQRVVRRVKK

EMQLNGQTLQVDQIISAWVGSANRDSNYFKDADSFNIHRRRNPHLAFGHGIHFCLGARLA

RLEARIVLTELIKKYKSFSFIDTNLPIPISNSSSVYGLKSFPVKSEMIQIK

>CYP102A5 *Bacillus thuringiensis serovar kurstaki* HD-1

MEKKVSAIPQPKTYGPLGNLPLIDKDKPTLSFIKIAEEYGPIFQIQTLSDTIIVVSGHEL

VAEVCDETRFDKSIEGALAKVRAFAGDGLFTSETHEPNWKKAHNILMPTFSQRAMKDYHA

MMVDIAVQLVQKWARLNPNENVDVPEDMTRLTLDTIGLCGFNYRFNSFYRETPHPFITSM

TRALDEAMHQLQRLDIEDKLMWRTKRQFQHDIQSMFSLVDNIIAERKSSGDQEENDLLSR

MLNVQDPETGEKLDDENIRFQIITFLIAGHETTSGLLSFAIYFLLKNPDKLKKAYEEVDR

VLTDPTPTYQQVMKLKYIRMILNESLRLWPTAPAFSLYAKEDTVIGGKYPIKKGEDRISV

LIPQLHRDKDAWGDNVEEFQPERFEELDKVPHHAYKPFGNGQRACIGMQFALHEATLVMG

MLLQHFELIDYQNYQLDVKQTLTLKPGDFKIRILPRKQTISHPTALAPTEDKLKNDEIKQ

HVQKTPSIIGADNLSLLVLYGSDTGVAEGIARELADTASLEGVRTEVVALNDRIGSLPKE

GAVLIVTSSYNGKPPSNAGQFVQWLEELKTDELKGVQYAVFGCGDHNWASTYQRIPRYID

EQMAQKGATRFSKRGEADASGDFEEQLEQWKQSMWSDAMKAFGLELNKNMEKERSTLSLQ

FVSRLGGSPLARTYEAVYASILENRELQSSSSDRSTRHIEVSLPEGATYKEGDHLGVLPV

NSEKNINRILRRFGLNGKDQVILSASGRSINHIPLDSPVSLLDLLSYSVEVQEAATRAQI

REMVTFTACPPHKKELEALLEEGVYHEQILKKRISMLDLLEKYEACEIRFERFLELLPAL

KPRYYSISSSPLVVQNRLSITVGVVNAPAWSGEGTYEGVASNYLAQRHNKDEIICFIRTP

QSNFELPKDPETPIIMVGPGTGIAPFRGFLQARRVQKQKGINLGEAHLYFGCRHPEKDYL

YRTELENDERDGLISLHTAFSRLEGHPKTYVQHLIKQDRINLISLLDNGAHLYICGDGSK

MAPDVEDTLCQAYQEIHEVSEQEARNWLDRVQDEGRYGKDVWAGI

>CYP106B4 *Bacillus thuringiensis serovar kurstaki* HD-1

MASPENVILVHEISKLKTKEELWNPYEWYRLMRDNHPVHYDEEQDVWNVFLYDDVNRVLS

DYRLFSSRRERRQFSIPPLETRININSTDPPEHRNVRSIVSKAFTPRSLEQWKPRIQAIA

DELVQHIEKCSEVNIVEQFAAPLPVTVISDLLGVPTTDRKKIKEWSDILFMPYSKEKFND

LDAEKGIALNEFKAYLLPIVQEKRYHLTDDIISDLIRAEYEDERLTDEEIVTFSLGLLAA

GNETTTNLIINSFYCFLVDSPGIYEELRKEPNLISKAIEEVLRYRFPVTLARRITEDTNI

FGSLMKKDQMIVAWVSAANLDEKKFSQASQFNVHRTGNEKHLTFGKGPHFCLGAPLARLE

AEIALTTFINAFEKIELSPSFCLEKCILENEQTLKYLPIRLKAK

>CYP102A5 *Bacillus thuringiensis serovar kurstaki* YBT-1520

MEKKVSAIPQPKTYGPLGNLPLIDKDKPTLSFIKIAEEYGPIFQIQTLSDTIIVVSGHEL

VAEVCDETRFDKSIEGALAKVRAFAGDGLFTSETHEPNWKKAHNILMPTFSQRAMKDYHA

MMVDIAVQLVQKWARLNPNENVDVPEDMTRLTLDTIGLCGFNYRFNSFYRETPHPFITSM

TRALDEAMHQLQRLDIEDKLMWRTKRQFQHDIQSMFSLVDNIIAERKSSGDQEENDLLSR

MLNVQDPETGEKLDDENIRFQIITFLIAGHETTSGLLSFAIYFLLKNPDKLKKAYEEVDR

VLTDPTPTYQQVMKLKYIRMILNESLRLWPTAPAFSLYAKEDTVIGGKYPIKKGEDRISV

LIPQLHRDKDAWGDNVEEFQPERFEELDKVPHHAYKPFGNGQRACIGMQFALHEATLVMG

MLLQHFELIDYQNYQLDVKQTLTLKPGDFKIRILPRKQTISHPTALAPTEDKLKNDEIKQ

HVQKTPSIIGADNLSLLVLYGSDTGVAEGIARELADTASLEGVRTEVVALNDRIGSLPKE

GAVLIVTSSYNGKPPSNAGQFVQWLEELKTDELKGVQYAVFGCGDHNWASTYQRIPRYID

EQMAQKGATRFSKRGEADASGDFEEQLEQWKQSMWSDAMKAFGLELNKNMEKERSTLSLQ

FVSRLGGSPLARTYEAVYASILENRELQSSSSDRSTRHIEVSLPEGATYKEGDHLGVLPV

NSEKNINRILRRFGLNGKDQVILSASGRSINHIPLDSPVSLLDLLSYSVEVQEAATRAQI

REMVTFTACPPHKKELEALLEEGVYHEQILKKRISMLDLLEKYEACEIRFERFLELLPAL

KPRYYSISSSPLVVQNRLSITVGVVNAPAWSGEGTYEGVASNYLAQRHNKDEIICFIRTP

QSNFELPKDPETPIIMVGPGTGIAPFRGFLQARRVQKQKGINLGEAHLYFGCRHPEKDYL

YRTELENDERDGLISLHTAFSRLEGHPKTYVQHLIKQDRINLISLLDNGAHLYICGDGSK

MAPDVEDTLCQAYQEIHEVSEQEARNWLDRVQDEGRYGKDVWAGI

>CYP106B4 *Bacillus thuringiensis serovar kurstaki* YBT-1520

MASPENVILVHEISKLKTKEELWNPYEWYRLMRDNHPVHYDEEQDVWNVFLYDDVNRVLS

DYRLFSSRRERRQFSIPPLETRININSTDPPEHRNVRSIVSKAFTPRSLEQWKPRIQAIA

DELVQHIEKCSEVNIVEQFAAPLPVTVISDLLGVPTTDRKKIKEWSDILFMPYSKEKFND

LDAEKGIALNEFKAYLLPIVQEKRYHLTDDIISDLIRAEYEDERLTDEEIVTFSLGLLAA

GNETTTNLIINSFYCFLVDSPGIYEELRKEPNLISKAIEEVLRYRFPVTLARRITEDTNI

FGSLMKKDQMIVAWVSAANLDEKKFSQASQFNVHRTGNEKHLTFGKGPHFCLGAPLARLE

AEIALTTFINAFEKIELSPSFCLEKCILENEQTLKYLPIRLKAK

>CYP107J3 *Bacillus thuringiensis serovar kurstaki* YBT-1520

MSMKNKVGLSIEDGINLASAQFKEDAYEIYKESRKKQPILFVNQVEIGKEWLITRYEDAL

PLLKDNRLKKDWTNVFSQDIKNMYLSVDNSDHLTTHMLNSDPPNHSRLRSLVQKAFTPKM

IAQLDKRIEKIADDLISDIERKGTLNLVDDYSFPLPIIVISEMLGIPKEDQAKFRIWSHA

VIASPETPEEIKETEKQLSEFITYLQYLVDIKRKEPKEDLVSALILAESEGHKLSARELY

SMIMLLIVAGHETTVNLITNTVLALLENPNQLQLLKDNPKLIDSAIEEGLRYYSPVEVTT

ARWAAEPFQIHDRTIEKGDMVVIALASANRDETVFENPEVFDITRENNRHIAFGHGSHFC

LGAPLARLEAKIAITTLFNRMPELQIKGNREEIKWQGNYLMRSLEELPLTF

>CYP102A5 *Bacillus thuringiensis serovar thuringiensis* IS5056

MEKKVSAIPQPKTYGPLGNLPLIDKDKPTLSFIKIAEEYGPIFQIQTLSDTIIVVSGHEL

VAEVCDETRFDKSIEGALAKVRAFAGDGLFTSETHEPNWKKAHNILMPTFSQRAMKDYHA

MMVDIAVQLVQKWARLNPNENVDVPGDMTRLTLDTIGLCGFNYRFNSFYRETPHPFITSM

TRALDEAMHQLQRLDIEDKLMWRTKRQFQHDIQSMFSLVDNIIAERKSNGNQEENDLLSR

MLNVPDPETGEKLDDENIRFQIITFLIAGHETTSGLLSFAIYFLLKNPDKLKKAYEEVDR

VLTDPTPTYQQVMKLKYIRMILNESLRLWPTAPAFSLYAKEDTVIGGKYPIKKGEDRISV

LIPQLHRDKDAWGDNVEEFQPERFEELDKVPHHAYKPFGNGQRACIGMQFALHEATLVMG

MLLQHFELIDYQNYQLDVKQTLTLKPGDFKIRILPRKQTISHPTVLTPTEDKLKNDEIKQ

HVQKPPSIIGADNLSLLVLYGSDTGVAEGIARELADTASLEGVRTEVVALNDRIGSLPKE

GAVLIVTSSYNGKPPSNAGQFVQWLEELKPDELKGVQYAVFGCGDHNWASTYQRIPRYID

EQMAQKGATRFSKRGEADASGDFEEQLEQWKQNMWSDAMKAFGLELNKNIEKERSTLSLQ

FVSRLGGSPLARTYEAVYASILENRELQSPSSDRSTRHIEVSLPEGATYKEGDHLGVLPV

NSEKNINRILKRFGLNGKDQVILSASGRSINHIPLDSPVSLLDLLSYSVEVQEAATRAQI

REMVTFTACPPHKKELEALLEEGVYHEQILKKRISMLDLLEKYEACEIRFERFLELLPAL

KPRYYSISSSPLVAHNRLSITVGVVNAPAWSGEGTYEGVASNYLAQRHNKDEIICFIRTP

QSNFELPKDPETPIIMVGPGTGIAPFRGFLQARRVQKQKGINLGQAHLYFGCRHPEKDYL

YRTELENDERDGLISLHTAFSRLEGHPKTYVQHLIKQDRINLISLLDNGAHLYICGDGSR

MAPDVEDTLCQAYQEIHEVSEQEARNWLDRVQDEGRYGKDVWAGI

>CYP106B4 *Bacillus thuringiensis serovar thuringiensis* IS5056

MASPENVILVHEISKLKTKEELWNPYEWYRLMRDNHPVHYDEEQDVWNVFLYDDVNRVLS

DYRLFSSRRERRQFSIPPLETRININSTDPPEHRNVRSIVSKAFTPRSLEQWKPRIQAIA

DELVQHIEKCSEVNIVEQFAAPLPVTVISDLLGVPTTDRKKIKEWSDILFMPYSKEKFND

LDAEKGIALHEFKAYLLPIVQEKRYHLTDDIISDLIRAEYEGERLTDEEIVTFSLGLLAA

GNETTTNLIINSFYCFLVDSPGIYEELRKEPNLILKAIEEVLRYRFPVTLARRITEDTNI

FGPFMKKNQMIVAWVSAANLDEKKFSQASQFNVHRTGNEKHLTFGKGPHFCLGAPLARLE

AEIALTTFINAFEKIELSPSFCLEKCILENEQTLKYLPIRLKAK

>CYP107J3 *Bacillus thuringiensis serovar thuringiensis* IS5056

MSMKNKVGLSIEDGINLASAQFKEDAYEIYKESRKKQPILFVNQVEIGKEWLITRYEDAL

PLLKDNRLKKDWTNVFSQDIKNMYLSVDNSDHLTTHMLNSDPPNHSRLRSLVQKAFTPKM

IAQLDGRIERIADDLISDIERKGTLNLVDDYSFPLPIIVISEMLGIPKEDQAKFRIWSHA

VIASPETPEEIKETEKQLSEFITYLQYLVDIKRKEPKEDLVSALILAESEGHKLSARELY

SMIMLLIVAGHETTVNLITNTVLALLENPNQLQLLKDNPKLIDSAIEEGLRYYSPVEVTT

ARWAAEPFQIHHQTIQKGDMVIIALASANRDETVFENPEIFDITRENNRHIAFGHGSHFC

LGAPLARLEAKIAITTLFNRMPELKIKGNREEIKWQGNYLMRSLEELPLTF

>CYP102A38 *Bacillus velezensis* FZB42

MKETGPIPQPKTFGPLGNLPLLDKDKPTMSLIKLANEQGPIFQLHTPAGAIIVVSGHELV

KEVCDEERFDKSIEGALEKVRAFSGDGLFTSWTHEPNWRKAHHILMPTFSQRAMKDYHSM

MTDIAVQLIQKWARLNPDEAVDVPADMTRLTLDTIGLCGFNYRFNSYYRETPHPFINSMV

RALDEAMHQMQRLDVQDKLMIRTKRQFHHDIQAMFSLVDSIIAERRSGGRDEKDLLARML

NVEDPETGEKLDDENIRFQIITFLIAGHETTSGLLSFAIYFLLKHPRVLEKAYEEADRVL

TDPVPSYKQVLDLTYIRMILQESLRLWPTAPAFSLYAKEDTVIGGKYPITPKDRISVLIP

QLHRDKDAWGDNAEEFYPERFEHPDRVPHHAYKPFGNGQRACIGMQFALHEATLVLGMIL

QHFTFIDHTDYELDIKQTLTIKPGDFHIRVRPRNKEDVAAALPAAEKAAEDVGKEKRETK

GASIIGLDNRPLLILYGSDTGTAEGVARELADTAGMHGVRTETAPLNDRIGKLPKEGALL

IITSSYNGKPPSNAGQFVQWLEEVKPGELEGVRYAVFGCGDHNWAATYQAVPRLIDEKLA

EKGAERFSSRGEGDVSGDFEGKLDEWKKSMWTDAMKAFGLKLNENAEKERSALGLQFVSG

LGGSPLAQTYEAVYASVAENRELQAPESGRSTRHIEITLPKEAAYHEGDHLGVLPVNSKE

QVSRVLRRFNLNGNDQVLLTASGQSAAHLPLDRPVRLHDLLSSCVELQEAASRAQIREMA

AYTVCPPHKRELEDFLEEGVYQEQILTSRVSMLDLLEKYEACELPFERFLELLRPLKPRY

YSISSSPRKHPGQASITVGVVRGPARSGLGEYRGVASNYLADRGPEDGIVMFVRTPETRF

RLPEDPEKPIIMVGPGTGVAPFRGFLQARAALKKEGKELGEAHLYFGCRNDHDFIYRDEL

EAYEKDGIVTLHTAFSRKEGVPKTYVQHLMAKDAGALISILGRGGHLYVCGDGSKMAPDV

EATLQKAYQSVRETDERQAQEWLLDLQTKGIYAKDVWAGI

>CYP102A49 *Bacillus velezensis* FZB42

MKQLSAIPQPKTYGPLKNLPHLEKEKLSQSLWKIAEEYGPIFRFEFPSSVGVFVSGRELA

AEVCDEKRFDKNLSKALLKVREFGGDGLFTSWTHEKNWQKAHRILLPSFSQKAMKGYHSM

MLDIAMQLVQKWSRLNPNEEIDVAEDMTRLTLDTIGLCGFHYRFNSFYRDTQHPFITSML

RALQEAMRQSQRHSLQDKLMIKTRHQFQQDIEEMNSLVDRIIAERRENPDENLSDLLALM

LEAKDPVTGERLDDENIRYQIITFLIAGHETTSGLLSFAIYCLLKNKDKLKKAVQEAERV

LTGETPEYKQIQQLTYIRMVLNETLRLYPTAPAFSLYAKEDTVLGGKYPIAKGQPVTILT

PQLHRDKSAWGEDAELFRPERFSDPAAIPADAYKPFGNGQRACIGMQFALQEATMVLGLV

LKHFELIDHTDYELTIKEALTIKPGDFKIRVKNKDVSNHQPVQNHKAEGSGHKEETKEIP

SHGTPLLILYGSNLGTAEGIAEELADIGRSKGFSTESGPLDDYAGKLPVKGAVVIVTASY

NGAPPDNAAGFVKWMETLEDQELKGVSYAVFGCGDRNWAATYQRIPRLIDKVLEEKGAKR

LTSIGEGDNADDFEYSQEAWEDSFWKDIMKAFHIEAAPKQTNKSQLSIEYVSEAAETPIA

KTYKAFEAEVITNKELHTESSKRSVRHIELRLPETETYQEGDHLGVLPQNSGELISRVIR

RFGLDPNQHFKIKGRQLPHLPMDRPVNAPELLASYVELQEPATRAQLRELAAHTVCPPHQ

KELEHLYSDDAAYKENVLKNRMTMLDLLEDYPACELPFERFLELLPSLKARYYSISSSPK

ATSGELSITVGVVTAPAWSGRGEYRGVASNYLAGLQKGDSAVCFIRSPQSGFALPENPKT

PLIMVGAGTGIAPFRGFIQARAAEKMSGNSLGEAHLYFGCRHPEQDDLYKDEFDHAEKNG

LVTVHRAYSRLDQDCKVYVQDVLLREAAQIIALLDQGGHLYICGDGSKMAPAVENVLLQA

YEKVHNTDSKVSLNWLEQLQAEGRYAKDVWAGM

>CYP107K3 *Bacillus velezensis* FZB42

MEKTMFHPHSPEFHENPFAVLSRFREQDPIHKFELQRFGGTFPAWLITRYDDCMAFLKDG

RITRDVKRVMPKELIAKLNVSEDIDFVSEHMLAKDPPDHSRLRSLVHQGFTPRMIEQLRT

GIEQITEELLDEMETKADPDIMRDFAAPLPFIVISELLGIPKEDRAKFQVWTNAMVDTSE

SGQDATNQALKEFKQYMKTLIEEKRKHPGEDLTSKLIYAEEDGQKLSESELYSMLFLLVV

AGLETTVNLLGSGTLALLLHKDQMEKIKRQPENIQTAVEELLRYTSPVIMMANRWAIEDF

TYKDVSIKKGDMIFIGIGSANRDPEYFDDPDTLNIARTPNRHISFGFGIHFCLGAPLARM

EASIAFTALLKRFPNIELKGAAEDVTWRKNVFLRGLETLPVRF

>CYP1179A4 *Bacillus xiamenensis* MVAGSFYLGFFICPRLMRRESCHISTNLKNESKILYVKTEGMMLKLKTEQSVEQLSLYSN

EYFERRYDIYKQLRNEAPVCWNEEMKSWFITRHADMTTNLPGDRFISSQVIPLKMNNLEK

GEDQHFKDIIDIIKTWMVYHDRPDHTELRGYMNRAFLVNEIELITPEIKKIVATIIDRVL

ESHTESFDFVEEIAHPIPAMVLCKMLGIPGTEVDKFIKWSDDIALFMQNFVVSHVPSKEI

SEQVRGSVKEIYAYLSNAIAERRKEKKNDLLSRLISETPGVNGELTDDQLIAQTMHLIFG

GHKIPQFVLSNSLHLLFKNPEILKMLKKDMSLLPKVLDETMRLEGPIQYIVRHASSDIEL

HGKQIRKNDSVYFFLASAGRDERVFEHPDKMDITRKGFRHMAFGGGYHTCIAAAFARAEI

MEILKEVISRCPDIQPLYDLENPEWTSNPTFTGILHMEVKVKRN

>CYP109A10(BK049_17760)*Bacillus xiamenensis*

MKVKKNYVNVIPMKEIRSTDDLLFPFPIYNELRSQGDIRYDERRKCWDLFRYADIQSVLK

HPKVFSSQRGRSTSKTSILTMDPPKHTKMRALVNKAFTPKAIKQLEDQIKDLTHDLLNQV

KDQRTFDLVQDLAAPLPVMIIAELLGAEVEDRELIKKHSDALVAGAKDDSKEAIQAVVEM

QKQAEEELSEYFAHLIQKRKETPADDLISLLIQAEIDGEHLTENELLGFCILLLVAGNET

TTNLITNAVRLLTEQPHIAESVRQDPSLIPQLTEETLRYYPPVQAIGRIAAEDVEIAGSH

IKKGDYLINWVASANRDERKFEDPDTFRLDRKSNPHMSFGFGIHFCLGAPLARLESHIAL

EILLNTFQEITCAADKLKPIQSTFVFGVKEFPVQVTRF

>CYP102A30(BK049_18240 K14338)*Bacillus xiamenensis*

MHQTSIIPKPKTYGPLKNIPLLKKGELSQTFWRLADDLGPIFQFEFTGQTSIFVSSHELV

SEVCDESRFDKYIGISLDKARAFAGDGLFTSWTEEPNWRKAHQILMPAFSQQAMKGYHEM

MLDIASQLVQKWQRTGRDEEIEVAEDMTKLTLDTIGLCGFDFRFNSFYKENQHPFIESML

KGLNEAMDQSSRLPIADKLMIKRRKEFEQNVDFMKQLVDDIIRERKTQDQTGDDLLSLML

NAKDPETGERLSDENIRYQIITFLIAGHETTSGLLSFAIYFLLKNPDKLKKAVQEADDVL

QDGLPSFKQMQKLSYTRMVLNEALRLWPTAPSFSLYAKEDTVIGGKYPIAKNQSVSVLLP

KLHRDQAVWGEDAEEFKPERFMHPETIPQHAYKPFGNGQRACIGMQFALHEATMVLAMVL

HNLELIDHTSYELDLKESLTIKPKDFKIKVRPRKQQFFMAPPKEEPKKSTASAASKVASH

GTPLLILYGSNLGTAKQMANEFAEDGQAKGFDVTTAPLDDYTGKLPESGAVLIVTASYNG

HPPDDAKQFVDWVTQDEEQDLSNVTFAVFGCGDRNWASTYQRIPRLIDEALERKGAKRLT

NIGEGDAGGDMDEDKEIFQKTVFEDLAKEFDLTLQEKSQESPDLSVVYTNELVERPVAKT

YGAFSAVVLKNRELQSEQSARQTRHIELQLPEGKPYKEGDHIGIVPKNSAALVQRVTNRF

KLDPQQHIMLSSAKEASHLPLNQPIQIRELLASHVELQEPATRTQLRELAKYTVCPPHRI

ELEQMAGEIYQEAVLKKRVTMLDLIEQYEACELPFHHFLALLPGLKPRYYSISSSPKVDE

KRLSITVAVVKGKAWSGRGEYAGVASNYLCGLQEGEEVACFLHQAQGGFELPPSPEIPMI

MIGPGTGIAPFRGFIQARGEWHNEGKQLGEAHLYFGCRHPHEDDLYYDEMQLAAQKGLVT

IHRAYSRYEEHKVYVQHLIKKDGVKLIEWLDQGAYLYICGDGKVMAPDVEAALIDLYMTV

KQCEKDTAEQWLTSLEKDNRYVKDVWS

>CYP109B5 *Bacillus altitudinis*

METTTPSAVQKALLRGKNKQDPYHPFDWYAKMRQESPIHFDEHSQTWSVFTYEEAKRVTI

DKDTFSSQPPQDHRKHSLMKTMVMMDPPKHTRIRSIVSKAFTPRVMKLWEPRIQELMDDL

IAQIEGKEEIDLVQDISYPLPVIVIAELLGVPTEHKQSFKEWSDILVSMPKSEEERDVVE

WQKTRDQGEADMMAFFADIIEEKRQNLSDDLISLLIQAEENGDKLSADELIPFCNLLLLA

GNETTTNLISNMIFSLLENPGSYEALAQSPDLIPRAVEEAVRFRAPAPTIVRYVTEDTEL

GGKVLKKGDSVIVFLASANRDERQFPNAHEYDIHRHPNPHIGFGHGIHFCLGAPLARLEA

CTAIKAIQSRYASLELLSYVPMTSSGMYGLKALKLRVTPRC

>CYP1179A4 *Bacillus altitudinis*

MYVKTEGMMLKMKTEQSVEQLSLYSNEYFERRYDIYKQLRNEAPVCWNEEMKSWFITRHA

DMTTNLPGDRFISSQVIPLKMNNLEKGEDQHFKDIIDIIKTWMVYHDRPDHTELRGYMNR

AFLVNEIELITPEIKKIVATIIDRVLEGHTESFDFVEEIAHPIPAMVLCKMLGIPGAEVD

KFIKWSDDIALFMQNFVVSHVPSKEISEQVRGSVKEIYAYLSNAIAERRKEKKNDLLSRL

ISETPGVNGELTDDQLIAQTMHLIFGGHKIPQFVLSNSLHLLFKNPEILNMLKKDMSLLP

KVLDETMRLEGPIQYIVRHASADIELHGKQIKKNDSVYFFLASAGRDERVFAHPDQMDIT

RKGFRHMAFGGGYHTCIAAAFARAEIMEILKEVISRCPDIQPLYDLENPEWTSNPTFTGI

LHMEVKVNGN

>CYP102A4 Bacillus anthracis H9401

MLFERVFVMDKKVSAIPQPKTYGPLGNLPLIDKDKPTLSFIKLAEEYGPIFRMQTLSDTI

IVVSGHELVAEVCDETRFDKSIEGALAKVRAFAGDGLFTSETQEPNWQKAHNILMPTFSQ

RAMKDYHAMMVDIAVQLVQKWARLNPNENVDVPEDMTRLTLDTIGLCGFNYRFNSFYRET

PHPFITSMTRALDEAMHQLQRLDIEDKLMWRTKRQFQHDIQSMFSLVDNIIAERKSSENQ

EENDLLSRMLNVQDPETGEKLDDENIRFQIITFLIAGHETTSGLLSFAIYFLLKNPDKLK

KAYEEVDRVLTDSTPTYQQVMKLKYIRMILNESLRLWPTAPAFSLYAKEDTVIGGKYPIK

KGEDRISVLIPQLHRDKDAWGDNVEEFQPERFEELDKVPHHAYKPFGNGQRACIGMQFAL

HEATLVMGMLLQHFEFIDYEEYQLDVKQTLTLKPGDFKIRIVPRNQTISHTTVLAPTEEK

LKNHEIKQQVQKTPSIIGADNLSLLVLYGSDTGVAEGIARELADTASLEGVQTEVAALND

RIGSLPKEGAVLIVTSSYNGKPPSNAGQFVQWLEELKGDELKGVQYAVFGCGDHNWASTY

QRIPRYIDEQMAQKGATRFSTRGEADASGDFEEQLEQWKQRMWSDAMKVFGLELNKNMEK

ERSTLSLQFVSRLGGSPLARTYEAVYASILENRELQSSSSERSTRHIEISLPEGATYKEG

DHLGVLPINSEKNVNRILKRFGLNGKDQVILSASGRSVNHIPLDSPVRLYDLLSYSVEVQ

EAATRAQIREMVTFTACPPHKKELESLLEDGVYQEQILKKRISMLDLLEKYEACEIRFEP

FLELLPALKPRYYSISSSPLVAQDRLSITVGVVNAPAWSGEGTYEGVASNYLAQRHNKDE

IICFIRTPQSNFQLPENPETPIIMVGPGTGIAPFRGFLQARRVQKQKGMNVGEAHLYFGC

RHPEKDYLYRTELENDERDGLISLHTAFSRLEGQAKTYVQHVIKEDRIHLISLLDNGAHL

YICGDGSKMAPDVEDTLCQAYQEIHEVSEQEARNWLDRLQEEGRYGKDVWAGI

>CYP107J2*Bacillus anthracis* H9401

MAMKNKVGIRIEDGINLASAQFKEDAYEIYKESRKVQPVLFVNKTELGAEWLITRYEDAL

PLLKDNRLKKDPANVFSQDTLNVFLTVDNSDYLTTHMLNSDPPNHNRLRSLVQKVFTPKM

IAQLEGRIQDIADDLLNEVERKGSLNLVDDYSFPLPIIVISEMLGIPKEDQAKFRIWSHA

VIAYPETPEEIKETEKQLSEFITYLQYLVDMKRKEPKEDLVSALILAESEGHKLSARELY

SMIMLLIVAGHETTVNLITNTVLALLENPNQLQLLKENPKLIDAAIEEGLRYYSPVEVTT

SRWADEPFQIHDQTIEKGDMVVIALAAANRDETVFENPEVFDITRENNRHIAFGHGSHFC

LGAPLARLEAKIAITTLFERMPELQIKGNREDIKWQGNYLMRSLEELPLTF

>CYP102A4 *Bacillus anthracis* SVA11

MLFERVFVMDKKVSAIPQPKTYGPLGNLPLIDKDKPTLSFIKLAEEYGPIFRMQTLSDTI

IVVSGHELVAEVCDETRFDKSIEGALAKVRAFAGDGLFTSETQEPNWQKAHNILMPTFSQ

RAMKDYHAMMVDIAVQLVQKWARLNPNENVDVPEDMTRLTLDTIGLCGFNYRFNSFYRET

PHPFITSMTRALDEAMHQLQRLDIEDKLMWRTKRQFQHDIQSMFSLVDNIIAERKSSENQ

EENDLLSRMLNVQDPETGEKLDDENIRFQIITFLIAGHETTSGLLSFAIYFLLKNPDKLK

KAYEEVDRVLTDSTPTYQQVMKLKYIRMILNESLRLWPTAPAFSLYAKEDTVIGGKYPIK

KGEDRISVLIPQLHRDKDAWGDNVEEFQPERFEELDKVPHHAYKPFGNGQRACIGMQFAL

HEATLVMGMLLQHFEFIDYEEYQLDVKQTLTLKPGDFKIRIVPRNQTISHTTVLAPTEEK

LKNHEIKQQVQKTPSIIGADNLSLLVLYGSDTGVAEGIARELADTASLEGVQTEVAALND

RIGSLPKEGAVLIVTSSYNGKPPSNAGQFVQWLEELKGDELKGVQYAVFGCGDHNWASTY

QRIPRYIDEQMAQKGATRFSTRGEADASGDFEEQLEQWKQRMWSDAMKVFGLELNKNMEK

ERSTLSLQFVSRLGGSPLARTYEAVYASILENRELQSSSSERSTRHIEISLPEGATYKEG

DHLGVLPINSEKNVNRILKRFGLNGKDQVILSASGRSVNHIPLDSPVRLYDLLSYSVEVQ

EAATRAQIREMVTFTACPPHKKELESLLEDGVYQEQILKKRISMLDLLEKYEACEIRFEP

FLELLPALKPRYYSISSSPLVAQDRLSITVGVVNAPAWSGEGTYEGVASNYLAQRHNKDE

IICFIRTPQSNFQLPENPETPIIMVGPGTGIAPFRGFLQARRVQKQKGMNVGEAHLYFGC

RHPEKDYLYRTELENDERDGLISLHTAFSRLEGQAKTYVQHVIKEDRIHLISLLDNGAHL

YICGDGSKMAPDVEDTLCQAYQEIHEVSEQEARNWLDRLQEEGRYGKDVWAGI

>CYP107J2 *Bacillus anthracis* SVA11

MAMKNKVGIRIEDGINLASAQFKEDAYEIYKESRKVQPVLFVNKTELGAEWLITRYEDAL

PLLKDNRLKKDPANVFSQDTLNVFLTVDNSDYLTTHMLNSDPPNHNRLRSLVQKVFTPKM

IAQLEGRIQDIADDLLNEVERKGSLNLVDDYSFPLPIIVISEMLGIPKEDQAKFRIWSHA

VIAYPETPEEIKETEKQLSEFITYLQYLVDMKRKEPKEDLVSALILAESEGHKLSARELY

SMIMLLIVAGHETTVNLITNTVLALLENPNQLQLLKENPKLIDAAIEEGLRYYSPVEVTT

SRWADEPFQIHDQTIEKGDMVVIALAAANRDETVFENPEVFDITRENNRHIAFGHGSHFC

LGAPLARLEAKIAITTLFERMPELQIKGNREDIKWQGNYLMRSLEELPLTF

>CYP107DF8 *Bacillus infantis*

MFNGNHQDNPEAYRIFSKEFIQNPYPGYRKLREEQPVYKTVMPDGQTGWVITKYEDAVAA

LKDKRFIKDFSKLYGGQMDHESIFTENMLFSDPPDHKRLRGLVQKAFTPRMIENMRGRIK

EIADQLLDRMENKEEITLIDDYAFPLPIIVISEILGVPTEDQDKFRIWSNSLIEGSNGEN

WNEIQQHMNEFVKYLGEWFAFLRDNPRDDLISQLINAEEGGDKLTEKELYGVVSLLIIAG

HETTVNLIGNGILSLLENPDQLKLLREKPELIHNAVEELLRYNGPVEYSTSRWAIEDVEF

QGQTIRKGELVIAALNSANRDDEKFADPDVLDITREKSPHLAFGKGIHLCLGAPLARLEG

EIAILALLERFPEIGLNASPDELEWRPGMIVRGVKELPLRVR

>CYP152A11 *Bacillus infantis*

MAMNGQIPHDKSLDNSLTLMKEGYEFIPNRKERYGTELFEARLLGQKVICMSGQEAARLF

YDTDRFKRNGAAPKRIQKTLFGENAIQTMDGDAHIHRKLLFMSLMTPPHQKRLAGMVMNE

WKAAAAKWQEDGREVVLMEEAKVILCRTACAWAGVPLKQEDEKERAEEFADMVDAFGAVG

PRHWKGRHARGSSEDWIRGLIEDVRDGKLAAEEGTALYEMAFHKNLDGTHLDSQMAAVEL

INVLRPIVAISTFIAFSALALNGHPRYKEMLQTRSGSELEMFVQEVRRFYPFGPFLGAIV

RKDFIWNEAEFKEGMLVLLDIYGTNHDPLLWEEPYEFRPERFENWDGSLFDLIPQGGGDA

SKGHRCPGEGITIEVMKASLDFLVNEIDYSVPENQDLSFSLSRMPTFPESGFIMSNIKKK

>CYP102A29 *Bacillus sp*. WP8

MKQTSIIPKPKTYGPFKNIPHIKKGELSQSFWRLADELGPIFQFEFSNATSIFVSSHELV

KEVFDESRFDKFIGTSLSKVRAFAGDGLFTSWTEEPNWRKAHQILMPAFSQQAMKGYHEM

MLDIATQLVQKWQRTGRDEEIEVAEDMTKLTLDTIGLCGFDFRFNSFYKENQHPFIESML

KGLNEAMEQSSRLPIADKLMIKRRKEFDQHVDFMKQLVDDIIQERRKQDKTGDDLLSLML

HAEDPETGERLSDENIRYQIITFLIAGHETTSGLLSFAIYFLLKNPEKLKKAVQEADEVL

QGGLPTFKQVQKLSYTRMVLNEALRLWPTAPTFSLYAKEDTVIGGKYPIHKNQSVSVLLP

KLHRDPAVWGEDAEEFKPERFTHPEKIPQHAYKPFGNGQRACIGMQFALHEATMVLAMVL

HNLELIDHTSYELDLKESLTIKPNDFKIKVRPRKQQFFMAPPKEEPKKSTKSGESKVPSH

GTPLLVLYGSNLGTAKQIANELAEDGKAKGFDVTTAPLDEYTRQLPDTGAVLIVTASYNG

YPPDNAKQFVDWLAQDEEQDLSNVTFVVFGCGDRNWASTYQRIPRLIDEALERKGAKRVA

DLGEGDAGGDMDEDKEIFQKTVFEQLAKEFKLTFQEKGKEKPNLSVAYTNELVERPVAKI

YDAFSAVVLKNEELQSEKSTRQTRHIELKLPAGKAYKEGDHIGIVPKNSDALVERVTRRF

KLNPEQHIKLSSEKEASHLPLEQPIPIKELLASHVELQEPATRTQLRELAAHTVCPPHRV

ELEQMAGEAYQEAILKKRVTMLDLLDQYEACELPFVHFLALLPGLKPRYYSISSSPKVDE

EKVSITVAVVKDKAWSGRGEYAGVASTYLCGLKEGEEVACFLHEAQDGFQLPPSSEVPMI

MIGPGTGIAPFRGFVQAREVWQKEGKQLGEAHLYFGCRHPEEDDLYFDEMQLAAQNGVIH

IHRAYSRYDEQKVYVQHLLKEEGGMLIELLDQGAYLYVCGDGKVMAPDVEATLIDLYQNE

KQCSKETAENWLTALANDNRYVKDVWS

>CYP109A8 *Bacillus sp*. WP8

MKVKENYVNVIPMKEIRSTDDLLFPFPIYNELRAESDIRFDETRKCWDLFRYADIQSVLK

QPKVFSSQRGRSTSKTSILTMDPPKHTKMRALVNKAFTPKAIKQLEDKIKDLTHDLLNQV

KDQRTFDIVQDLAAPLPVMIIAELLGAEVQDRELIKKHSDALVAGAKDESKEAIQAVVDM

QKRAEEELSVYFAQLIKKRKETPADDLISLLIQAEIDGERLTENELLGFCILLLVAGNET

TTNLITNAVRLLTEQPHIAESVRQDPSLIPQLTEETLRYYPPVQAIGRIAAEDVEIAGAR

IEKGDYLIGWVASANRDELKFEDPDTFRLDRKSNPHMSFGFGIHFCLGAPLARLESNIAL

DVLLHTFQDITCAADQLKPIQSTFVFGVKEFPVQVTRF

>CYP102A5 *Bacillus thuringiensis serovar kurstaki* HD73

MEKKVSAIPQPKTYGPLGNLPLIDKDKPTLSFIKIAEEYGPIFQIQTLSDTIIVVSGHEL

VAEVCDETRFDKSIEGALAKVRAFAGDGLFTSETHEPNWKKAHNILMPTFSQRAMKDYHA

MMVDIAVQLVQKWARLNPNENVDVPEDMTRLTLDTIGLCGFNYRFNSFYRETPHPFITSM

TRALDEAMHQLQRLDIEDKLMWRTKRQFQHDIQSMFSLVDNIIAERKSSGDQEENDLLSR

MLNVQDPETGEKLDDENIRFQIITFLIAGHETTSGLLSFAIYFLLKNPDKLKKAYEEVDR

VLTDPTPTYQQVMKLKYIRMILNESLRLWPTAPAFSLYAKEDTVIGGKYPIKKGEDRISV

LIPQLHRDKDAWGDNVEEFQPERFEELDKVPHHAYKPFGNGQRACIGMQFALHEATLVMG

MLLQHFELIDYQNYQLDVKQTLTLKPGDFKIRILPRKQTISHPTALAPTEDKLKNDEIKQ

HVQKTPSIIGADNLSLLVLYGSDTGVAEGIARELADTASLEGVRTEVVALNDRIGSLPKE

GAVLIVTSSYNGKPPSNAGQFVQWLEELKTDELKGVQYAVFGCGDHNWASTYQRIPRYID

EQMAQKGATRFSKRGEADASGDFEEQLEQWKQSMWSDAMKAFGLELNKNMEKERSTLSLQ

FVSRLGGSPLARTYEAVYASILENRELQSSSSDRSTRHIEVSLPEGATYKEGDHLGVLPV

NSEKNINRILRRFGLNGKDQVILSASGRSINHIPLDSPVSLLDLLSYSVEVQEAATRAQI

REMVTFTACPPHKKELEALLEEGVYHEQILKKRISMLDLLEKYEACEIRFERFLELLPAL

KPRYYSISSSPLVVQNRLSITVGVVNAPAWSGEGTYEGVASNYLAQRHNKDEIICFIRTP

QSNFELPKDPETPIIMVGPGTGIAPFRGFLQARRVQKQKGINLGEAHLYFGCRHPEKDYL

YRTELENDERDGLISLHTAFSRLEGHPKTYVQHLIKQDRINLISLLDNGAHLYICGDGSK

MAPDVEDTLCQAYQEIHEVSEQEARNWLDRVQDEGRYGKDVWAGI

>CYP106B4 *Bacillus thuringiensis serovar kurstaki* HD73

MASPENVILVHEISKLKTKEELWNPYEWYRLMRDNHPVHYDEEQDVWNVFLYDDVNRVLS

DYRLFSSRRERRQFSIPPLETRININSTDPPEHRNVRSIVSKAFTPRSLEQWKPRIQAIA

DELVQHIEKCSEVNIVEQFAAPLPVTVISDLLGVPTTDRKKIKEWSDILFMPYSKEKFND

LDAEKGIALNEFKAYLLPIVQEKRYHLTDDIISDLIRAEYEDERLTDEEIVTFSLGLLAA

GNETTTNLIINSFYCFLVDSPGIYEELRKEPNLISKAIEEVLRYRFPVTLARRITEDTNI

FGSLMKKDQMIVAWVSAANLDEKNFHKPLNLMYIEQEMKSI

>CYP102A4 *Bacillus anthracis* A0248

MDKKVSAIPQPKTYGPLGNLPLIDKDKPTLSFIKLAEEYGPIFRMQTLSDTIIVVSGHEL

VAEVCDETRFDKSIEGALAKVRAFAGDGLFTSETQEPNWQKAHNILMPTFSQRAMKDYHA

MMVDIAVQLVQKWARLNPNENVDVPEDMTRLTLDTIGLCGFNYRFNSFYRETPHPFITSM

TRALDEAMHQLQRLDIEDKLMWRTKRQFQHDIQSMFSLVDNIIAERKSSENQEENDLLSR

MLNVQDPETGEKLDDENIRFQIITFLIAGHETTSGLLSFAIYFLLKNPDKLKKAYEEVDR

VLTDSTPTYQQVMKLKYIRMILNESLRLWPTAPAFSLYAKEDTVIGGKYPIKKGEDRISV

LIPQLHRDKDAWGDNVEEFQPERFEELDKVPHHAYKPFGNGQRACIGMQFALHEATLVMG

MLLQHFEFIDYEEYQLDVKQTLTLKPGDFKIRIVPRNQTISHTTVLAPTEEKLKNHEIKQ

QVQKTPSIIGADNLSLLVLYGSDTGVAEGIARELADTASLEGVQTEVAALNDRIGSLPKE

GAVLIVTSSYNGKPPSNAGQFVQWLEELKGDELKGVQYAVFGCGDHNWASTYQRIPRYID

EQMAQKGATRFSTRGEADASGDFEEQLEQWKQRMWSDAMKVFGLELNKNMEKERSTLSLQ

FVSRLGGSPLARTYEAVYASILENRELQSSSSERSTRHIEISLPEGATYKEGDHLGVLPI

NSEKNVNRILKRFGLNGKDQVILSASGRSVNHIPLDSPVRLYDLLSYSVEVQEAATRAQI

REMVTFTACPPHKKELESLLEDGVYQEQILKKRISMLDLLEKYEACEIRFEPFLELLPAL

KPRYYSISSSPLVAQDRLSITVGVVNAPAWSGEGTYEGVASNYLAQRHNKDEIICFIRTP

QSNFQLPENPETPIIMVGPGTGIAPFRGFLQARRVQKQKGMNVGEAHLYFGCRHPEKDYL

YRTELENDERDGLISLHTAFSRLEGQAKTYVQHVIKEDRIHLISLLDNGAHLYICGDGSK

MAPDVEDTLCQAYQEIHEVSEQEARNWLDRLQEEGRYGKDVWAGI

>CYP1221A2 *Bacillus cellulosilyticus*

MNNRRNSNYPPGPKEKWLTGSLRAFQSSPLKFLTSLSEKYGTVSKFRLGPFQDVYLVNDP

DLIKEILVSKQQSFIKSRDIQSLKSIVGNGLLTSEKGFHLKQRRMIQPAFKKTHITTYAQ

DMIDTTNKYISRWSSRAERLVSDDMMDIALGIISKTMFSMEFEEGASVIGEPMEETMRTA

VRRMRSILPLPLWIPVKQNRKYKQAIKELDNVLFRLIKERKETEVEHEDLLGVLMRAKDE

TDGLSMEDNQLRDELMTIFLAGHETTANALTWTLYLLSQHRKIQDKLFKEIASITRDGPV

KPEHFGRLTYAQHVISESLRLYPPAYVIGRQAAEDTEINGYRIKKGDMILMSQYVMQRNR

KYYEDPHTFIPERFENDFIKTIPEYAYFPFGGGPRVCIGNHFAFMEAVLVLACLSKQFKF

TSPHEPQKIKPQPLITLRPKYGLTLLTTKRR

>CYP102A8*Bacillus cereus* F837/76

MDKKVSAIPQPKTYGPLGNLPLIDKDKPTLSFIKLAEEYGPIFRIQTLSDTIIVVSGHEL

VAEVCDETRFDKSIEGALAKVRAFAGDGLFTSETQEPNWQKAHNILMPTFSQRAMKDYHA

MMVDIAVQLVQKWARLNPNENVDVPEDMTRLTLDTIGLCGFNYRFNSFYRETPHPFITSM

TRALDEAMHQLQRLDIEDKLMWRTKRQFQHDIQSMFSLVDNIIAERKSSENQEENDLLSR

MLNVQDPETGEKLDDENIRFQIITFLIAGHETTSGLLSFAIYFLLKNPDKLKKAYEEVDR

VLTDPTPTYQQVMKLKYIRMILNESLRLWPTAPAFSLYAKEDTVIGGKYPIKKGEDRISV

LIPQLHRDKDAWGDNVEEFQPERFEELDKVPHHAYKPFGNGQRACIGMQFALHEATLVMG

MLLQHFEFIDYEDYQLDVKQTLTLKPGDFKIRIVPRNQTISHTTVLAPTEEKLKNHEIKQ

QVQKTPSIIGADNLSLLVLYGSDTGVAEGIARELADTASLEGVQTEVVALNDRIGSLPKE

GAVLIVTSSYNGKPPSNAGQFVQWLEELKPDELKGVQYAVFGCGDHNWASTYQRIPRYID

EQMAQKGATRFSTRGEADASGDFEEQLEQWKQRMWSDAMKAFGLELNKNMEKERSTLSLQ

FVSRLGGSPLARTYEAVYASILENRELQSSSSERSTRHIEISLPEGATYKEGDHLGVLPI

NSEKNVNRILKRFGLNGKDQVILSASGRSVNHIPLDSPVRLYDLLSYSVEVQEAATRAQI

REMVAFTACPPHKKELESLLEDGIYHEQILKKRISMLDLLEKYEACEIRFERFLELLPAL

KPRYYSISSSPLVAQDRLSITVGVVNAPAWSGEGTYEGVASNYLAQRHNKDEIICFIRTP

QSNFQLPENPETPIIMVGPGTGIAPFRGFLQARRVQKQKGMKVGEAHLYFGCRHPEKDYL

YRTELENDEREGLISLHTAFSRLEGHPKTYVQHVIKEDRIHLISLLDNGAHLYICGDGSK

MAPDVEDTLCQAYQEIHQASEQEARNWLDRLQDEGRYGKDVWAGI

>CYP107CB1 *Bacillus clausii*

MKPTSSEPIDLFSDQFHQQPYTYYKDIREQTGFAKVMLPYGIPAWMAFHYDVAEAVLKDE

RFIKDARTVFPDEVSDEQMLPISKSMLFVDPPDHKRLRGLIQKGFTPKRISRLKGRIDAI

AMEQARRIKQKKRFDLVEEYAFPIPIIVICELLGIPDSDRDKFQYWSKLIVDLDNDGYGE

SSTVQEGMNDFLAYLQALIHARRQDPREDLLSDLIRAEEDGDRLTTNELYGVVMLLIVAG

HETTVNLIANGMLALLMHPDQLALLKNDDQLIPQAVEELLRYNSPVEFSTDRWARESFSF

MGKDIKKGDFVIVSLASANHDEALVEHPDKLDITREKSPHLSFGKGIHYCLGAPLARLEA

ESAIRVLLEECPDIRLGAEPAELAWRQSLIIRGLENLPVETG

>CYP152A13 *Bacillus coagulans* 36D1

MAGQIPNEKTIDDTIDLMKEGYLYIKNRTDDYHANLFETRLLGQKAVCISGEEAVKMFYD

PEKFKRNGAVPKRVQKTLFGENAIQTLDGKEHLHRKALFLSLMGPDAQQHLAQMVRAAWE

ARIPEWEQKDRIVLFDEAKLLLCRCACKWAGVPLREEEAEKRAQEFIDMVYAFGAVGPRH

WKGRTARNSTEDWIRTVIEDVRAGKIEAKDGSPLKEMAFYEDAQGRRFNAQMAAVELINV

IRPVTAIATFITFAALALFEHPRARDWVQNGDKQHIDWFTREVRRFYPFGPFLGAKVRKS

FTWNGYPFDEGLLVLLDMYGTNRDPAIWGDPDVFSPERFRDWDGSLYNFIPQGGADPAKG

HRCPGEGITQEIIEASLDFLVNAIDYDVPAQDLSVPLNKMPTLPESGFILQHVKKKTPAS

>CYP107CB3 *Bacillus flexus*

MNIKSLDLFSQKFHQNPYEYYEQIRLHEPFAKVKLYTEAPDSWMAFTYEAAEAVLKDERF

IKDMRTVFPGEMTDENVPPISQTMLFADPPNHRRLRSLVQSGFTPKKIMSLSGRIEEIAR

EQAKRMKEHETVDFIETYAFPIPIIVICELLGVPQEDQLDFQRWSNVLVDINEASQYDEV

LMEFMMYLEKLIHNRRQSPQNDLLSHLIQAEEDGDKLTATELYGVVMLLIVAGHETTVNL

ISNGLLALLTHPDQLALLKNDPSLISKAIDEFLRYNGPVEFSTDRWARESFTFMGQQVQK

GDHVIVSLASTGRDPEVFSHPEKLDITREKNPHLAFGKGIHYCLGAPLARLEGEIAIRVL

LEEFPNVGLVANLSDLEWRQSFIIRGLKELPIRLA

>CYP197A1 *Bacillus halodurans*

MPTNTMPTGPKGNPVLGNTIEFGKDPLQFITRCSQEYGEIVRLRFERERDTFLLNDPKHI

QYVFMNKGGEFSKGYQQDPIMGLVFGNGLLTSEGSFWLRQRRLSQPAFHPKRIADYADTM

VGYCERMLNTWMDNDTRDINDEMMQLTMAIATKTLFDLDLHKGDTQEASRSLDTVMTAFN

EQMTNVFRHVLHLIGLGKLVPPVSRELREAVESLDKMIYSIIEERRKHPGDRGDLLSMLI

STYDEDDGSYMTDRQLRDEIITLFLAGHETTANTLSWAFYLLSQHPHVEEKLYQEVSQVL

GNRPATLEDMPKLSYAEHVIKETLRVQPTVWLISRRAEKDVTLGDYHISAGSEIMISQWG

MHRNPRYFNDPLTFLPERWDNNDNKPSKYVYFPFGGGPRVCIGERFALMEATLIMATIVR

EFRMELVDELPIKMEPSITLRPKHGVTMKLRKR

>CYP152N3(B4U37_03195)*Bacillus horikoshii*

MPKEEGLDHSLKLLSEGYQFIMNRRHMFHSNIFETTLLAEKAICLTGKEAAEIFYDNEKF

KRKGAAPKRLQKTLFGEGGVQAMDGEAHHHRKAMFMSLMTKKSLKEITVLTRKAWKNAAL

TWEREDEIVLYIEAKKILTQVACEWTGVPLSEDKVEKFAVQLGDMYESAAAIGLKHWKGR

RSRSSVESWLEDLVEEVREEKLEVPIDKALYQFSMHRDLNGELLDKEIVAVELLNLLRPI

VAISVYVCFSALAVHQYPEEAAKLHKGETASLQRFVQEVRRFYPFFPFTAARVKEDFTWN

SYKFEKDTLTLLDLYGTNHHPEDWENPDRFEPDRFKTWDKSPFDFIPQGGGEYDIGHRCA

GEWLTVDIMNVSLDFLVNDLEYDIPEQDLSFSMSQIPSLPESKMVMRVKGTGTATHSH

>CYP107CB2 *Bacillus lehensis*

MSIVLNKKEFHQEPYEFYKEIRPHDAFAKVKLVSGIQDSWVAFTYEAAEAVLKDERFVKN

PRAVFPDVSEHELMPITHSMLFADPPDHRRLRSLVQRGFTPKMIQRLQGRIEEIAKIQVE

QMKGKETVDLIADYAFPIPIIVICELLGVPPEDRLDFQRWSNSMVEINDDPSFYEQVEAH

MKEFQLYIEQLLAEKRIHPQDDLLSELIRAEEDGDKLSVQELYGAIMLMIVAGHETTVNL

IANGMLALFTHPEQLKKLKESPSLIDGAIEEILRFNGPVEFSTDRYAKESFTFMGKQLQK

GDHVLVSLASADHDPAVFSEPDKLVITREKSPHLAFGKGIHYCLGAPLARLEGKIAIQTL

LNTFPEIQINTALANLEWRQSFVIRGLKEPAGEIELMFEKHVPSLKAQVF

>CYP109B *Bacillus methanolicus*

MENRTMEVRLIPSSYLRGKIDPYDPFLWYEEMRKQTPVYYNDKAGMWNVFLYKDVKRVLE

DKEFFSNVIPQRGKSSLSQSIIAMDPPKHTDIRSIVSRSFTPKVMREWESRIHEIASELL

NSVQGQSEFDIVNDFSYPLPVIVIAEMLGVPSSEMKLFKDWSDTLVSSPENDHPEHLAKF

KETRNRTEAELSAFFEQIIEEKRAHPGNDLISILIKAS

>CYP106A6 *Bacillus oceanisediminis*

MLKEVIPVNEITNFKSRAEEFFPIEWYKDMLHHHPVYYHEQTNTWNVFTYEGVKQVLGNY

EFFSSAGPRTTIFVGANDKHEKASPLTNLTLVDPPDHRKGRSLLAAAFTPRSLKNWEPRI

KQIAEELVENIQDNNEINIVEALAAPLPSMVIADLFGVPIQDRAQFKEWVDILFQPYDKE

RLEDIELQKQNAAKEYFQYLYPIVVQKRSNLSDDIISDLIQAEVDGEKFTDNEIVQVTML

LLGAGVETTSHAIANTFYSLLYDDESLYGELRNDLELVPNAVEEMLRYRFHMSRRDRTVK

KDNNLLGVELKEGDVVIAWMSACNMDHRMFEDPFSINIHRPNNKKHLTFGNGPHFCLGAP

LARLEMKIALETFVKKFSSIEPVEGFELEKNLTASATGQSLTNLPMNVYK

>CYP109A7 *Bacillus smithii*

MATAKKTSYETKRYANLIPMKEIQSVQDQLFPFTIYNTLRKKTPVRYDPIRECWDVFRYE

DVHFILKNPALFSSNRGIGDKRNSILIMDPPKHTKMRNLINKTFTPKAVNELSQRIQDVT

TSLLDQAKEKETLEMIRDFAAPLPVIIIAEILGVPAKDRELFKNYSDILVSGAEDDSDEA

FNMMMKRRREGAKFLNDYFKEIIQERQRSPKDDLISLLLAAEVDGERLKEEELLKFCILL

LVAGNETTTNLIVNAVRYMVEDTNTQETVRKNFSLVPNLIEETLRFYPPIQAIGRTATAD

VEIGGHTIRKGSQVISWVAAANRDEQKFEQPDRFMLERHPNPHLGFGYGIHFCLGAPLAR

LEAKVALSVLLTTFSKLELAKHTELEPIQSPFVFGVKSFPIQFSL

>CYP152A15 *Bacillus sp*. OxB-1

MQRPIERGLDHSLNLLTEGYSFIRNRTESFGAPLFETRLMGKKMICMTGKEAVQLFYDEN

RFQRHGAIPKRIQKSLFGVGGVQTMDGELHAVRKQLFLSLMTEEKLAELRSSTSAQWRLW

AEAQNGREVVLFDETEKILCRVACLWAGVPLKEADVGGRAYDFGAMVDAFGGVGPRYQEG

KNARKRAEQWIEQIIEKYRSGAIESEEGKAIQAVATHRDADGRLLDTHSAAVELINVLRP

VVAVARFIIFGALALHDFPEYKDKLKECEEEFLEMFAQEVRRFYPFAPMLGAKTRYDFKW

NGYRFAKGETVLLDLYGTNHDPELWENPDTFLPERFADRKEDLYDFVPQGGGDPNSGHRC

PGEKATVEIMKASFRFLVEELDYEVIDDQDLTISHVRMPTLPKSRFIIRVK

>CYP102A8 *Bacillus toyonensis*

MDKKVSAIPQPKTYGPLGNLPLIDKDKPTLSFIKIAEEYGPIFQIQTLSDTIIVVSGHEL

VAEVCDETRFDKSIDGALAKVRAFAGDGLFTSETDEPNWKKAHNILMPTFSQRAMKDYHG

MMVDIAVQLVQKWARLNPNENVDVPEDMTRLTLDTIGLCGFNYRFNSFYRETSHPFITSM

SRALDEAMHQLQRLDIEDKLMWRTKRQFQHDIQSMFSLVDNIIAERKSNGNQEENDLLAR

MLNVQDPETGEKLDDENIRFQIITFLIAGHETTSGLLSFAIYFLLKNPDKLKKAYEEVDR

VLTDPTPTYQQVMKLKYIRMILNESLRLWPTAPAFSLYAKEDTVIGGKYPIKKGEDRISV

LIPQLHRDKDAWGDNVEEFQPERFEEPDKVPHHAYKPFGNGQRACIGMQFALHEATLVMG

MLLQHFELIDYQNYQLDVKQTLTLKPGDFKIRILARNQTISHPTVLAPIEEKQKDHEIKQ

QVQKTPSIIGADNLSLLVLYGSDTGVAEGIARELADTASLEGVQTEVVALNERIGSLPKE

GAVLIVTSSYNGKPPSNAGQFVQWLEELKPDELKGVQYAVFGCGDHNWASTYQRIPRYID

EQMAQKGATRFSTRGEADASGDFEEQLEQWKQSMWSDAMRAFGLELNKNMEKERSTLSLQ

FVSRLGGSPLARTYEAVYASILENRELQSSSSDRNTRHIEVSLPEGATYQEGDHLGVLPI

NSEKNVNRILKRFGLNGKDQVILSASGRSVNHIPLDSPVSLFDLISYSVEVQEAATRAQI

REMVTFTACPPHKKELESLLEEGVYHERILKKRISMLDLLEKYEACEIRFERFLELLPAL

KPRYYSISSSPLVAQDRLSITVGVVNAPAWSGEGTYEGVASNYLAQRHNKDEIICFIRTP

QSNFQLPENPETPIIMVGPGTGIAPFRGFLQARRVQKQKGINLGQAHLYFGCRHPEKDYL

YRTELENDERDGLISLHTAFSRLEGHPKTYVQHLIKQDRINLISLLDNGAHLYICGDGSK

MAPDVEDTLCQAYEEIHEVSEQEARNWLDYLQHEGRYGKDVWTGI
