# Supplementary figures and images for: Comparative Analyses of Cytochrome P450s and Those Associated with Secondary Metabolism in Bacillus Species
Source: Int J Mol Sci. 2018 Nov 16;19(11):3623. doi: 10.3390/ijms19113623 (PMC6275058; doi:10.3390/ijms19113623)

# Taxonomy

- CYP152
- CYP102
- CYP134
- CYP113
- CYP106
- CYP109
- CYP107

0.1

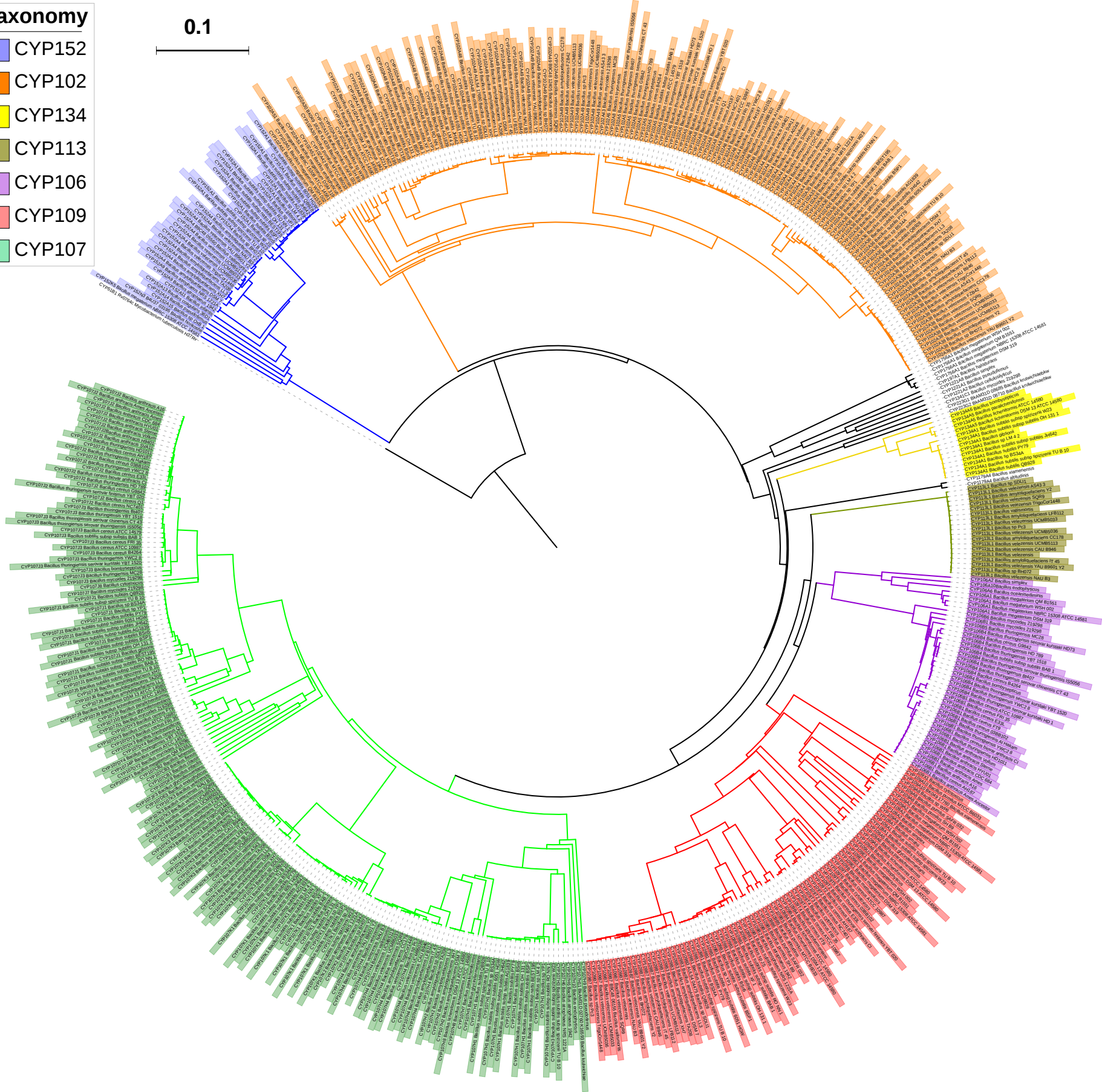

Supplement: Supplementary file 1 [file ijms-19-03623-s001.zip › Supplementary files/Figure S1.pdf]
